# Anion‐Bridged Dual Hydrogen Bond Enabled Concerted Addition of Phenol to Glycal

**DOI:** 10.1002/advs.202308513

**Published:** 2024-01-15

**Authors:** Qinbo Jiao, Zhenbo Guo, Mingwen Zheng, Wentao Lin, Yujie Liao, Weitao Yan, Tianfei Liu, Chunfa Xu

**Affiliations:** ^1^ Institute of Pharmaceutical Science and Technology College of Chemistry Fuzhou University Fuzhou 350108 China; ^2^ State Key Laboratory of Elemento‐organic Chemistry College of Chemistry Nankai University Weijin Road No. 94 Tianjin 300071 China; ^3^ Haihe Laboratory of Sustainable Chemical Transformations Tianjin 300192 China; ^4^ Key Laboratory of Organofluorine Chemistry Shanghai Institute of Organic Chemistry Chinese Academy of Sciences Shanghai 200032 China

**Keywords:** concerted addition, glycosylation, hydrogen bond, organocatalysis, phenolic glycoside

## Abstract

A noncovalent organocatalytic concerted addition of phenol to glycal is developed for the stereoselective and regioselective construction of biologically important phenolic 2‐deoxyglycosides, featuring wide substrate tolerance. The method relies on an anion‐bridged dual hydrogen bond interaction which is experimentally proved by Nuclear Magnetic Resonance (NMR), Ultraviolet and visible (UV–vis), and fluorescence analysis. Experimental evidence including kinetic analysis, Kinetic Isotope Effect (KIE) studies, linear free energy relationship, Hammett plot, and density functional theory (DFT) calculations is provided for a concerted mechanism where a high‐energy oxocarbenium ion is not formed. In addition, the potential utility of this method is further demonstrated by the synthesis of biologically active glycosylated flavones. The benchmarking studies demonstrate significant advances in this newly developed method compared to previous approaches.

## Graphical abstract

A concerted addition of phenol to glycal promoted by anion‐bridged dual hydrogen bond interaction is disclosed, featuring excellent stereoselectivity and broad substrate tolerance. The unprecedented concerted addition pathway is confirmed by kinetic analysis, KIE studies, Hammett plot and DFT calculation. The synthesis of biologically active glycosylated flavones and benchmarking studies demonstrate the significant advance of this method.

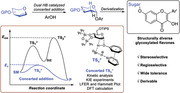

## 1 Introduction

In recent years, noncovalent catalysis has gained increasing attention because of its excellent and regulated reactivity, insensitivity to moisture and oxygen, and low toxicity, which has a tremendous impact on the synthesis of pharmaceutical intermediates.^[^ ^1^ ^]^ Due to the fact that carbohydrate molecules are at the forefront of drug discovery and biochemistry, recent research has focused on developing noncovalent strategies for stereoselective glycosylation to target these structurally diverse molecules.^[^ ^2^ ^]^

**Figure 1:**
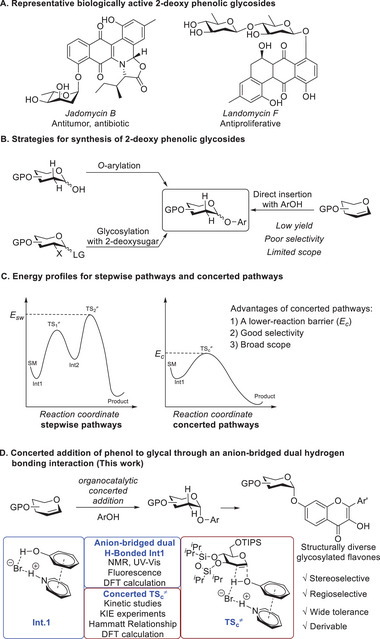
Graphic summary of essential and synthesis of phenolic 2‐deoxyglycoside, energy profiles for stepwise and concerted pathways in this work.

The phenolic 2‐deoxyglycosides make up a large group of carbohydrates that occur in many natural products, exhibiting extensive and unique biological properties such as antibiotics, antibacterial, anticancer, and enzyme inhibitory properties (**Figure** **1**A).^[^ ^3^ ^]^ As a result, the synthesis of these interesting phenolic glycosides has drawn considerable attention from the synthetic community as well as from medicinal chemists.^[^ ^4^ ^]^ However, there are some inherent challenges associated with the stereoselective construction of phenolic 2‐deoxyglycosides. First, due to their electron‐withdrawing properties of aromatic rings, phenols are considered to be more difficult substrates to glycosylate than their analogous alcohols. The second challenge is that phenols are amphipathic nucleophiles, thus, they are prone to converting to *C*‐glycosides through rearrangement, leading to lower yields of the targeted *O*‐glycosides. The third challenge that hinders stereoselective synthesis is the absence of substituents at C2, which is not able to afford neighboring group participation. Furthermore, glycosidic bonds of related products are considerably labile in acidic media. To date, three strategies have been employed to construct phenolic 2‐deoxyglycosides, which involve arylation of anomeric hydroxyl groups,^[^ ^5^ ^]^ nucleophilic substitution of glycosyl donors containing leaving groups at anomeric center,^[^ ^2^, ^6^ ^]^ and direct addition of phenol to glycals.^[^ ^7^ ^]^ (Figure 1B). While the former two strategies were capable of producing the desired compounds, the major drawback of these strategies is the need to synthesize the glycosyl donor in multiple steps, which involves introducing and removing auxiliary functional groups such as halo and chalcogen atoms, making them time‐consuming and producing substantial reagent waste. Virtually, most of those glycosyl donors are derived from more accessible glycals. Thus, direct catalytic and stereoselective addition of phenolic nucleophiles to 1,2‐glycals is the most straightforward and practical method. In recent decades, great efforts have been made with the aim to achieve efficient transformations by using various catalysts such as HPPh_3_Br,^[^ ^7d^ ^]^ boronic acid,^[^ ^7e^ ^]^ or transition metal salts.^[^ ^7f^ ^]^ Although these progresses have been made, the existing methods either suffer from low yields and poor selectivity or have a very limited sugar scope. Further, it has been reported that altering the sugar moiety of glycoconjugates can have a significant impact on their biological activity.^[^ ^8^ ^]^ As a result, the development of broadly applicable, efficient, and stereoselective protocols for phenolic 2‐deoxyglycosides is still in high demand.

In recent years, an interesting activation mode through forming a catalyst‐acceptor complex, which is capable of activating the glycosyl donor and regulating the selectivity, was proposed by Schmidt's group^[^ ^9^ ^]^ originally, and largely extended in noncovalent catalytic glycosylation by Schmidt,^[^ ^2e^ ^]^ Jacobsen,^[^ ^2j–n^ ^]^ Pedersen.^[^ ^2v^ ^]^ It was suggested that a conformationally constrained donor–catalyst–acceptor intermediate was formed which governed the displacement of anomeric leaving group by glycosyl acceptor in an intramolecular S_N_2‐like manner. To the best of our knowledge, the reported 2‐deoxyglycosylations of phenol with glycal were reported to proceed in a stepwise fashion with the formation of the highly energetic oxocarbenium ions as intermediates, resulting in less efficient glycosylation.^[^ ^7^ ^]^ With the continuing interest in carbohydrate^[^ ^10^ ^]^ and inspired by the abovementioned elegant works,^[^ ^2e,j–n,v^ ^]^ we envision that an unprecedented concerted addition of phenol to glycal via catalyst–acceptor activation by noncovalent organocatalysis would uncover the limitations in the production of 2‐deoxy phenolic glycosides. This specific concerted process might significantly lower the reaction energy barrier which could rule out the pathways going through high‐energy intermediates, leading to a broadly applicable and stereoselective glycosylation (Figure 1C).^[^ ^11^ ^]^ In this study, we report a pyridinium bromide‐catalyzed concerted 2‐deoxyglycosylation of phenols via an anion‐bridged dual hydrogen‐bonded reactive intermediate (**Int.1** in Figure 1D).

## 2 Results and Discussion

Our initial proposal was to use pyridinium salt^[^ ^12^ ^]^ which is easily accessible and structure‐regulated as the organocatalyst since it has an anion‐bridged hydrogen bonding structure^[^ ^13^ ^]^ and can form *π*–*π* stacking.^[^ ^14^ ^]^ The strength of the O─H bond of phenol can be attenuated as a result, which would facilitate the addition of phenol of glycal. Taking this into account, we chose triacetyl‐D‐glucal (**1a**) and 2‐naphthol (**2a**) as model substrates and pyridinium bromide **A** as the catalyst to test the glycosylation. Unfortunately, the reaction suffered from a serious Ferrier rearrangement and provided only trace amounts of the targeted addition product. Because the reactivities of glycals are very dependent on the protecting groups, we turned to use the highly armed silylated glucal **1b** ^[^ ^15^ ^]^ as the model to further optimize the conditions. In the following experiments, a series of pyridinium salts were screened by varying the counter anion and the substituents on the pyridine moiety (**Table** **1**, entries 1–8). Based on the results, it was found that the anion had a strong influence on the catalyst's behavior (Table 1, entries 1–5). Regarding conversion and yield, bromide catalyst **A** was an excellent choice. Other catalysts either had a low reaction rate or produced significant side products. The introduction of functional groups with different electron properties did not improve the transformation. Solvents with a nonpolar structure, such as toluene, were found to be the most suitable, whereas solvents with a higher polarity had detrimental effects on glycosylation (Table 1, entries 9–11). Changes in temperature did not give better results (Table 1, entries 12–13). It appears that the control experiments without catalyst or using the ethylated congener **I** significantly indicated the importance of the **N**─**H** substructure (Table 1, entries 14–15). Meantime, some of the commonly used Lewis acids or Brönsted acids for glycosylation were assessed and it was found that no product was formed along with a significant composition of glycal. While Schreiner's catalyst (1,3‐bis[3,5‐bis(trifluoromethyl)phenyl]thiourea) which was active in the glycosylation of alcohol^[^ ^2d^ ^]^ presented no reactivity. To facilitate further transformation, the reaction was conducted with 1.5 equivalents of **2a**, resulting in 91% isolated 2‐deoxy phenolic glycosides. The structure of compound **3a** was determined by NMR analysis. The *J*‐value of the equatorial proton and axial proton at the C2 position is 13.6, 5.3, 1.3 Hz, and 13.5, 11.4, and 3.6 Hz respectively. Therefore, the anomeric proton was suggested to be located at the equatorial orientation. A further 1D NOE spectrum was recorded with the irradiation at the position of the anomeric proton, and a signal of the proton at the C4 position was observed, demonstrating the α configuration.

**Table 1:** Reaction Optimization.

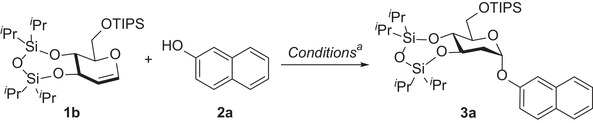					

Entries	Catalyst	Solvent	Temp [/°C]	Conversion [%]	Yield [%]
1	**A**	Toluene	70	78	78
2	**B**	Toluene	70	66	63
3	**C**	Toluene	70	86	79
4	**D**	Toluene	70	38	36
5	**E**	Toluene	70	82	68
6	**F**	Toluene	70	38	37
7	**G**	Toluene	70	100	76
8	**H**	Toluene	70	91	63
9	**A**	ClCH_2_CH_2_Cl	70	83	68
10	**A**	THF	70	63	15
11	**A**	DMSO	70	37	0
12	**A**	Toluene	RT	15	12
13	**A**	Toluene	90	81	75
14	**none**	Toluene	70	0	0
15	**I**	Toluene	70	0	0
16	**Et_3_N.HCl**	Toluene	70	0	0
17	**TfOH**	Toluene	70	100	0
18	**BF_3_.Et_2_O**	Toluene	70	100	0
19	**TsOH.H_2_O**	Toluene	70	100	0
20	**TMSOTf**	Toluene	70	100	0
21	**Schreiner's catalyst**	Toluene	70	0	0
22c	**A**	Toluene	70	92	91
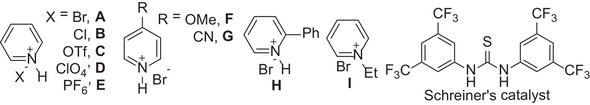					

^a)^ **1b** (0.05 mmol, 1 equiv.), **2a** (0.05 mmol, 1 equiv.), catalyst (20 mol%), temperature, solvent (0.05 m), 12 h, nitrogen; Conversions and yields were determined by crude ^1^H NMR spectra analysis using 1,3,5‐trimethoxybenzene as an internal standard based on the glycal **1b**;

^b)^ 1 mol%. *^c^* **2a** (1.5 equiv.) was used.

**Figure 2:**
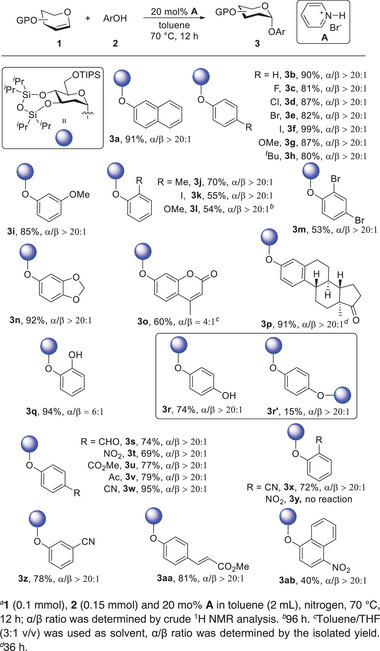
Phenol scope for D‐glucal. *a* denotes 1 (0.1 mmol), **2** (0.15 mmol) and 20 mol% **A** in toluene (2 mL), nitrogen, 70 °C, 12 h; α/β ratio was determined by crude ^1^H NMR analysis. b denotes 96 h. c denotes Toluene/THF (3:1 v/v) was used as solvent, α/β ratio was determined by the isolated yield. d denotes 36 h.

Following the establishment of optimal glycosylation conditions, we examined the generality of this phenolic glycosylation protocol (**Figure** **2**). The results clearly demonstrated the utility and robustness of the catalyst pyridinium bromide **A**. Initially, a variety of electron‐rich phenols bearing different functional groups were examined. In the majority of cases, the corresponding products were obtained in good to excellent yields, accompanied by high stereoselectivities (**3a, g–i**). Previous protocols reported lower yields for the substrates containing a substituent in the ortho‐position.^[^ ^4a,g^ ^]^ We were delighted to discover that these challenging aglycones tolerated our method well, yielding the corresponding products in satisfactory yields (**3j–m**). In addition, some bioactive phenolic compounds including sesamol, 4‐methylcoumarin and estrone were also confirmed successfully (**3n–p**). Dihydroxylphenol‐derived glycosides were found to exhibit biological activities.^[^ ^16^ ^]^ Therefore, we sought to investigate the reaction profiles of them. As shown in Figure 2, pyrocatechol reacted in a regioselective manner, only affording monoglycosylation product (**3q**). In contrast, both hydroxyl groups of the hydroquinone presented the reactivity for glycosylation, but the monoglycosylation product still predominated (**3r, 3r′**). Then we moved to explore the glycosylation reactions with the notably electron‐deficient phenols which were less reactive in acidic media. Pleasingly, the phenols with various electron‐withdrawing groups including aldehyde, nitro, ester, ketone, and cyano proved to be active in this protocol along with excellent stereoselectivities (**3s–x, z‐ab**). It was noteworthy that if these functional groups except cyano were placed in the vicinal position of the hydroxyl group, the substrates could not provide any products. It is possible that this difference was caused by intramolecular hydrogen bonding, resulting in attenuated phenol reactivity.^[^ ^17^ ^]^

**Figure 3:**
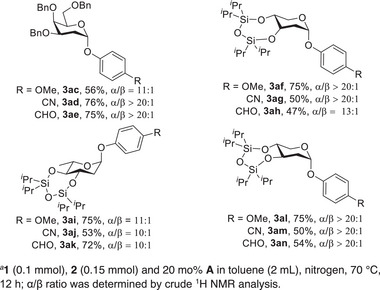
Investigation of sugar scope. a **1** (0.1 mmol), **2** (0.15 mmol) and 20 mol% **A** in toluene (2 mL), nitrogen, 70 °C, 12 h; α/β ratio was determined by crude ^1^H NMR analysis. a denotes 1. (0.1 mmol), **4** (0.15 mmol) and 20 mol% **A** in toluene (2 mL), nitrogen, 70 °C, 12 h; α/β ratio was determined by crude ^1^H NMR analysis; for synthesis of flavonoid glycoside: **5** (0.1 mmol, 1 equiv.), ArCHO (1.2 equiv.), NaH (6 equiv.), H_2_O_2_ (18 equiv.). b denotes 3,4,6‐triacetylgalctal was used as substrate.

**Figure 4:**
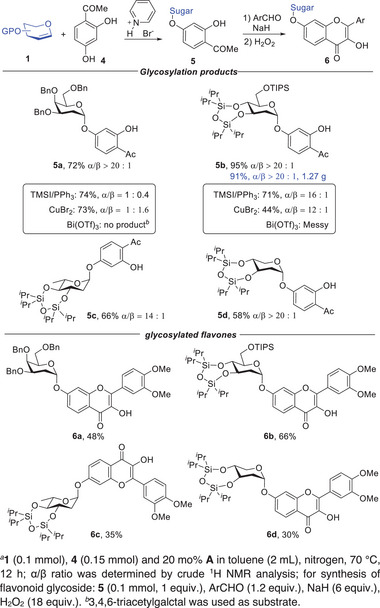
Further derivatization to access flavonoid glycosides and benchmarking studies for 2,4‐dihydroxyacetophenone (**4**).

Our success with glucal encouraged us to investigate the reactions of other sugar donors. Fortunately, this protocol was also adaptable to substrates with a variety of structures. As illustrated in **Figure** **3**, benzylated galactal, silylated arabinal, rhamnal, and xylal were readily converted into the desired 2‐deoxyphenolic glycosides. Likewise, the phenols with different electron properties were compatible with other glycals. In order to demonstrate the synthetic potential of this methodology, we performed a series of glycosylation reactions with aglycone **4‐producing** intermediates **5** that could be used to construct the flavonoid glycosides. On one hand, glycosylated flavones might be an effective method to enhance their bioavailability and pharmaceutical activity.^[^ ^3a^ ^]^ The reaction, on the other hand, could provide structurally diverse flavonoid glycosides that would greatly expand the chemical space and accelerate the discovery of new drugs. As depicted in **Figure** **4**, in the first step, the glycosylation of 2,4‐dihydroxylacetophenone proceeded well in a regioselective and stereoselective manner, affording a series of monoglycosylation products **5**. With these intermediates **5** in hand, we were curious about the transformation to form the glycosylated flavones **6**. As expected, treatment of **5** with veratraldehyde (3,4‐dimethoxybenzaldehyde) under basic conditions and followed by a cyclization process in the presence of H_2_O_2_, the flavonoid glycosides **6** could be obtained smoothly. This would open new avenues for the diverse synthesis of unnatural glycosylated flavones. Moreover, benchmarking studies with the useful and challenging aglycone **4** between this newly developed method and another conventional catalysis including TMSI/PPh_3_,^[^ ^7c^ ^]^ CuBr_2_,^[^ ^7i^ ^]^ and Bi(OTf)_3_^[^ ^7g^ ^]^ were conducted. As shown in Figure 4 (Framed), the results unambiguously revealed that the performance of pyridinium bromide **A** was significantly superior to that of other catalysts. It was noteworthy that this protocol was easily scaled up without losing the selectivity. Notably, the gram‐scale synthesis of **5b** was successfully executed.

**Figure 5:**
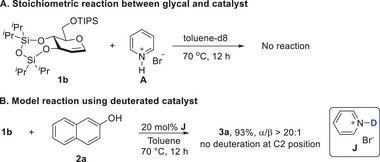
Control experiments reveal no interaction between catalyst and glycal.

We were encouraged by the excellent results to further recognize the intrinsic nature of broad tolerance and high stereoselectivity. We then turned our attention to studying the mechanism of pyridinium bromide‐catalyzed glycosylation in order to accomplish this goal. As a first step, a control experiment using stoichiometric amount catalyst **A** and glycal **1b** in deuterated toluene at 70 °C was conducted to determine whether the proton in the N─H bond of pyridinium cation could activate the glycosyl donor (**Figure** **5** **A**). The chemical shifts of **1b** in the presence or absence of **A** don't change at all (See Figure S1, Supporting Information), which strongly suggests that the catalyst is not directly protonating the glycal to give the oxocarbenium cation, in accordance with previous reports.^[^ ^12a^ ^]^ In addition, another control reaction was performed with the deuterated catalyst **J**, and the product **3a** was isolated in 93% yield, no deuteration at the C2 position was determined by ^1^H NMR spectrum and HRMS analysis (Figure 5B; Figure S2, Supporting Information). This also supports that the catalyst should not protonate the glycal.

**Figure 6:**
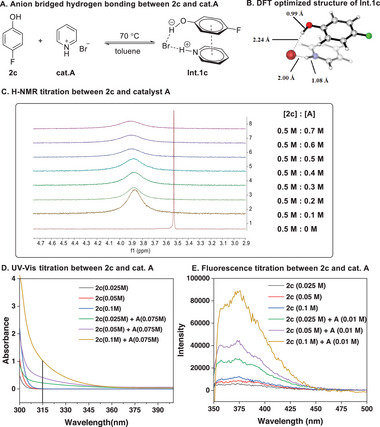
In situ characterization of the anion‐bridged H‐bonded intermediates **Int.1c** generated from **2c** and **cat.A**, and its DFT optimized structure.

In stark contrast, the ^1^H peak of OH in **2c** shifts downfield when it is mixed with **A**, and the chemical shift and the peak width are significantly dependent on the concentration of **A** (**Figure** **6** **C**; Figure S3, Supporting Information). These results reveal that there should be an intermediate **Int.1c** exists in equilibrium between phenol **2c** and catalyst **A** (Figure 6A). Subsequently, UV–vis spectra of the sole **2c** and the mixture of **2c** and **A** at various concentrations clearly showed a new species is formed (Figure 6D), and its absorbance at 315 nm is linearly dependent on the catalyst's concentration (Figure S4, Supporting Information). Additionally, the notable enhancement of fluorescence of the intermediate **Int.1c** generated from **2c** and **A** was observed, further affirming the existence of **Int.1c** in toluene (Figure 6E). Moreover, as previously shown in Entry 15 of Table 1, 1‐ethylpyridinium bromide **I** has no activity to catalyze the reaction. The computational results completely support the existence of intermediate **Int.1c**, which is bonded by the anion‐bridged dual hydrogen bonds, and also benefited from the *π*–*π* stacking between the aromatic rings of **2c** and **A** (Figure 6B; Table S11, Supporting Information).

**Figure 7:**
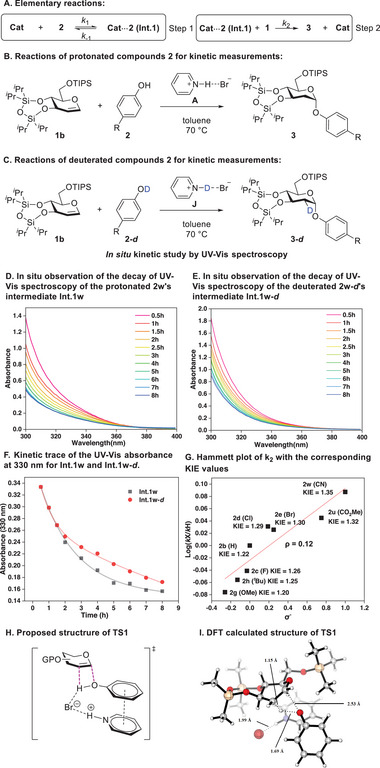
Kinetic analysis for the reactions between protonated/deuterated phenol **2/2‐*d*** and **1b**, Hammett plot and the proposed transition state.

**Scheme 1:**
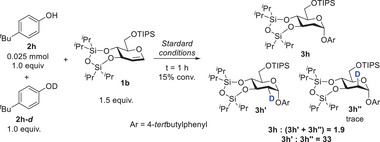
Competition reactions between **2** **h** and **2h‐*d***.

We can follow the kinetics of the reaction by monitoring the absorbance of the intermediate **Int.1** through UV–vis spectroscopy since the starting materials, catalysts, and products appear silent within the range of observation of the UV–vis spectrum. As the reaction progressed, the absorbance of **Int.1** decreased gradually (see **Figure** **7**B,D). This allows us to propose the reaction mechanism in Figure 7A, which includes two steps: **step 1** is a rapid equilibrium step where catalyst **A** and phenol **2** form hydrogen‐bonded intermediate **Int.1**; step **2** is the glycosylation of **Int.1** with glycal **1** to form the product **3**, with the kinetic order being determined to be second order (See SI for details). The observation of **Int.1** during the reaction course implies that the glycosylation step (step 2) is the rate‐determining step. Our DFT computational methods were used to determine the equilibrium constants (*K_eq_*) and Gibbs free energy changes of the first steps between the catalyst **A** and the phenolic substrates (**2b–e**, **2g–h**, **2u** and **2w**), as shown in Table S7 and S13 (Supporting Information). By deriving the kinetic equations, we were able to establish the correlation between the absorbance change rate of intermediate **Int.1** and the second‐order rate constant of the second‐step reaction, as well as to solve the rate constant value (see Table S7, Supporting Information). In addition, the deuterated phenolic substrates (**2‐*d***) were prepared. Using these deuterated reagents, we determined the rate constants of the second step of the deuterated reactions through UV–vis spectroscopy (Figure 7C,E, see in Table S7, Supporting Information). The KIE values of these second‐step reactions are stable between 1.20 and 1.35 for all phenolic substrates (see Table S9, Supporting Information). Furthermore, we conducted a competition experiment using one‐to‐one ratios of deuterated and non‐deuterated phenolic substrates (**Scheme** **1** and Figure S10, Supporting Information). According to our findings, the isotope effect of the overall reaction (**3h**:(**3h′**+**3h″**)) is 1.9. This value is consistent with the measured KIE of the reaction's second step (Figure 7A,G). Nevertheless, it is surprising that the ratio between the products deuterium‐labeled in the equatorial bond (**3h″**) and the axial bond (**3h″**) is 33:1. Due to such selectivity, we can infer that concerted addition has taken place. In light of our research experience with other reaction mechanisms,^[^ ^18^ ^]^ we calculated the theoretical equilibrium isotope effect EIE of the reaction when it occurs in a step‐by‐step manner, which should be 0.99 (see Table S10 and Figure S9, Supporting Information). As this predicted EIE value is not consistent with our experimentally measured KIE values, we can rule out the possibility of step‐by‐step addition. Based on the measured *k_2_*, we drew the Hammett plot^[^ ^19^ ^]^ of the second step, resulting in a slope ρ of 0.12 (Figure 7G), indicating that the reaction should not undergo a large charge change during the entire mechanism, and probably pass through a concerted transition state such as **TS1** (Figure 7H,I).

**Figure 8:**
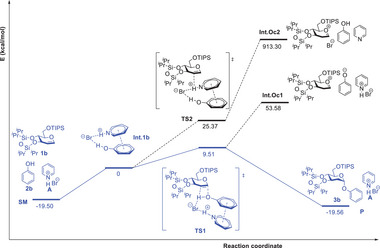
DFT‐calculated reaction coordinate of the Cat. A catalyzed addition of phenol 2b to glycal 1b.

In an effort to get more insights into the mechanism of the reaction, density functional theory (DFT) calculations were performed by the B3LYP in conjunction with the 6–31+G(d,p) basis set in toluene solution with the SMD continuum solvation models, and **Figure** **8** shows the reaction coordinate diagram (see details in Tables S11 and S12, Supporting Information). In toluene solution, our catalyst and substrates only require an uphill of 19.50 kcal mol^−1^ to generate the active intermediate **Int.1b**. Then, it takes only 9.51 kcal mol^−1^ uphill to go through the transition state **TS1** of the concerted addition from intermediate **Int.1b**, while the proton on the Br∙∙∙HO hydrogen bond transfers to the C2 position of the glycal, forming a C─H bond synchronously with a C─O bond at the C1 position to form a stable product (−19.56 kcal mol^−1^ relative to **Int.1b**). An alternative pathway via **TS1** to form the oxocarbenium cation intermediate **Int.Oc1** is excluded due to its unreasonable high energy (53.58 kcal mol^−1^ uphill). There is also a proposed pathway that undergoes a transition state **TS2** with higher energy (25.37 kcal mol^−1^ uphill relative to **Int.1b**), in which the proton in the Br∙∙∙HN hydrogen bond of **Int.1b** transfers to the C2 position. Nevertheless, the subsequent formation of the oxocarbenium cation intermediate **Int.Oc2** also requires unreasonable energy uphill (913.30 kcal mol^−1^), which makes it impossible to be considered. The exclusion of such a step‐wise pathway is consistent with our previous experiments which excluded the protonation of the glycal **1b** by **A** (Figure 5A). As a result, based on the computational results, we conclude that the reaction involves the rapid formation of intermediate **Int.1b** and the formation of the product **P** via one transition state **TS1**. This confirmed mechanism pathway is consistent with our experimental evidence, including the characterization of the active intermediate, the determination of the rate constants with their KIE values of the second step, and the analysis of the linear free energy relationship from the Hammett plot of the reaction.

## 3 Conclusion

In summary, we have developed a concerted pyridinium catalysis for broadly applicable, stereoselective and regioselective 2‐deoxyglycosylation of phenols with easily accessible glycal. This approach is strongly relied on an anion‐bridged dual hydrogen bond interaction which is fully confirmed by NMR, UV–vis, fluorescence analysis and further verified by DFT calculations. The real‐time kinetic monitoring, KIE experiments, free energy relationship and DFT calculation collectively support the unprecedentedly concerted addition process. As a result, this method tolerates a wide range of glycosyl donors and glycosyl acceptors, leading to structurally diverse phenolic glycosides. Hexoses such as glucal, galactal, rhamnal as well as the less‐explored pentoses xylal, arabinal are demonstrated as glycosyl donors efficiently. Not only the electron‐rich phenols, but also the challenging electron‐deficient substrates work well with this protocol. Besides, a successful transformation to furnish the unnatural bioactive flavonoid glycosides involving a glycosylation of 2,4‐dihydroxyacetophenone as the key step is achieved. Benchmarking studies display the robustness of the pyridinium catalysis in both yield and selectivity in comparison to the previously known reports. This novel process of the pyridinium catalysis would open a new venue for efficient and selective 2‐deoxyglycosylation with glycal. Further application of pyridinium catalysis and biological investigation of the obtained products are currently undergoing in our laboratory.

## Conflict of Interest

The authors declare no conflict of interest.

## Supporting information

Supporting Information

## Data Availability

The data that support the findings of this study are available in the supplementary material of this article.
