# Supplementary material for: Anion‐Bridged Dual Hydrogen Bond Enabled Concerted Addition of Phenol to Glycal
Source: Adv Sci (Weinh). 2024 Jan 15;11(11):2308513. doi: 10.1002/advs.202308513 (PMC10953558; doi:10.1002/advs.202308513)
Supplement: Supplementary file 1 — Supporting Information [file ADVS-11-2308513-s001.pdf]

ADVANCED SCIENCE
Open Access

Supporting Information

for *Adv. Sci.*, DOI 10.1002/advs.202308513

Anion-Bridged Dual Hydrogen Bond Enabled Concerted Addition of Phenol to Glycal

Qinbo Jiao, Zhenbo Guo, Mingwen Zheng, Wentao Lin, Yujie Liao, Weitao Yan, Tianfei Liu and Chunfa Xu**

Supporting Information for

Anion-Bridged Dual Hydrogen Bond Enabled Concerted Addition of Phenol to Glycal

Qinbo Jiao, Zhenbo Guo, Mingwen Zheng, Wentao Lin, Yujie Liao, Weitao Yan, Tianfei Liu* and Chunfa Xu*

E-mail: xucf@fzu.edu.cn;

E-mail: tianfeiliu@nankai.edu.cn

Table of Contents

General information……………………………………………………..………………….....3
General procedure for optimization…………………………………………………………...4
General procedure for synthesis of pyridine salts……………………………………………..4
General procedure for synthesis of *O*-glycals……………………………………………..……..4
General procedure for preparation of flavonoid glycosides…….……………………………..….4
General procedure for preparation of deuterated phenols……………………………………...5
NMR data …………..…………………………………………………………………..…......7
Structure determination………………………………………………………………………34
Benchmarking studies………………………………………………………………………..36
Reactions with flavonoid substrate and alcohols……………………………………………..37
Gram-scale reaction…………………………………………………………………………38
Mechanistic studies…………………………………………………………………………..39
Investigation of the interaction between catalyst and glycal………………………………………39
Investigation of the interaction between catalyst and phenol………………………………………40
Determination of the order of the reaction ………………………………………………………43
Chemical kinetics………………………………………………………………………...44
UV-Vis titration of catalyst and phenol mixture in toluene…………………………………………46
Kinetic monitoring…………………………………………………………………………47
Hammett plot………………………………………………………………………………50
Theoretical equilibrium isotope effect……………………………………………………...52
Competition experiment……………………………………………………………………53
DFT calculation……………………………………………………………………………54
Reference……………………………………………………………………………..…...87
NMR spectra…………………………………………………...………………….…...88

General information

Unless otherwise stated, all reactions were set up under inert atmosphere (N_2) utilizing glassware that were oven-dried and cooled under nitrogen atmosphere. Silica Gel Flash Column Chromatography was performed silica gel (particle size 300-400 mesh). Starting materials were purchased from commercial suppliers (Energy Chemical, Bidepharm, Tansoole) and used directly without further purifications unless otherwise stated. All solvents were dried according to standard procedures or purchased from commercial suppliers. Toluene and Et_2O were obtained by distillation from Na/benzophenone and further degassed by freezing-pump-thaw under argon. All samples for mechanistic studies were weighed on OHAUS semi-micro electronic balance PX125DZH. During the titration, the valve type gas-tight microliter syringes produced by Shanghai GaoGe was used, and the volume have been calibrated by the weight of the standard pure solvent using OHAUS balance inside glovebox. Reactions were monitored using thin-layer chromatography (TLC). Visualization of the developed plates were performed under UV light (254 nm) or H_2SO_4-EtOH (10% H_2SO_4 v/v).

^1H NMR, ^{19}F NMR and ^{13}C NMR spectra were recorded on Bruker AVIII 400 spectrometer and JEOL JNM-EAC600 spectrometer. ^1H NMR and ^{13}C NMR chemical shifts were reported in parts per million (ppm) downfield from tetramethylsilane and ^{19}F NMR chemical shifts were determined relative to $PhOCF_3$ as the external standard and low field is positive. Coupling constants (*J*) are reported in Hertz (Hz). The residual solvent peak was used as an internal reference: ^1H NMR ($CDCl_3$ δ 7.26 ppm), ^{13}C NMR ($CDCl_3$ δ 77.16 ppm), ^1H NMR (DMSO-d_6 δ 2.50 ppm), ^{13}C NMR (DMSO-d_6 δ 39.50 ppm), The following abbreviations were used to explain the multiplicities: s = singlet, d = doublet, t = triplet, q = quartet, m = multiplet, br = broad. IR spectra were recorded on Nicolet iS50 spectrometer. HRMS data was recorded on HRMS Exactive Plus instrument. Melting point was measured on SGW X-4A instrument. The UV-Vis spectra and kinetic measurements were recorded by Agilent Cary 60. NMR spectra were recorded on Bruker AV400. Fluorescence spectroscopy was recorded on Edinburgh Instruments FLS920.

General procedure for reaction optimization (Procedure A)

In a glove box filled with nitrogen, to an oven-dried 10 mL tube equipped with a stirring bar were added D-glucal **1b** (0.05 mmol, 1.0 equiv.), 2-naphthol **2a** (0.05 mmol, 1.0 equiv.), catalyst, solvent (1 mL). The tube was sealed with a Teflon screw cap and the mixture was stirred at an indicated temperature. Then the solvent was removed under reduced pressure, and the yield was determined by ^{1}H NMR spectroscopy in $CDCl_3$ with 1,3,5-trimethoxybenzene as an internal standard.

General procedure for synthesis of pyridinium salts (Procedure B)

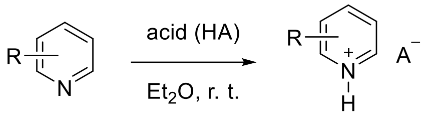

To a round-bottom flask equipped with a stirring bar was added an ethyl ether solution of pyridine, then the corresponding acid was added dropwise. A white precipitation was observed. When the pyridine was consumed completely, the precipitation was filtered, and washed with ethyl ether three times. Afterwards, the solid was dried under vacuum to afford the targeted product.

General procedure for synthesis of *O*-glycal (Procedure C)

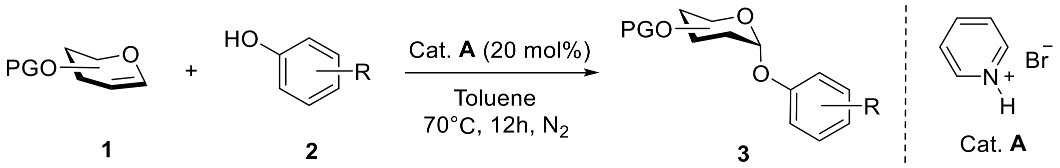

In a glove box filled with nitrogen, to an oven-dried 10 mL tube equipped with a stirring bar were added sugar **1** (0.1 mmol, 1.0 equiv.), phenol **2** (0.15 mmol, 1.5 equiv.), catalyst, solvent (2 mL). The tube was sealed with a Teflon screw cap and the mixture was stirred at 70°C. Upon completion, solvent was removed with rotary evaporator and the residue was purified by silica gel chromatography (EtOAc/petroleum ether) to afford the product **3**.

General procedure for synthesis of flavonoid glycosides (Procedure D)

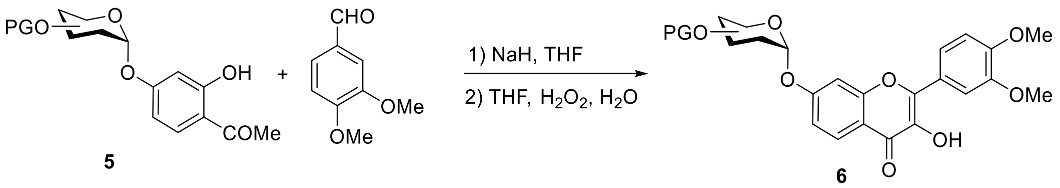

In a glove box filled with nitrogen, to an oven-dried 10 mL tube equipped with a stirring bar were added NaH (0.6 mmol, 6.0 equiv.), THF (2 mL). Glycoside **5** (0.1 mmol, 1.0 equiv.) and veratraldehyde (0.12 mmol, 1.2 equiv.) were dissolved in THF (1 mL), and then added to the NaH mixture dropwise. The resulting mixture was stirred at room temperature for 12 h. After that, H_2O (1 mL) was added to quench reaction. Subsequently, H_2O_2 (30% w/w in H_2O, 1.8 mmol, 18.0 equiv.) was added, and the mixture was stirred at room temperature for additional 24 h. Upon completion, EtOAc was added to dilute the solution and HCl (1 M) was used to adjust the pH to 4. The organic phase was separated and dried over Na_2SO_4, filtered, and evaporated under vacuum. The residue was purified by basified silica gel (deactivated by NEt_3) column chromatography to give the targeted product.

General procedure for preparation of deuterated phenols (Procedure E)

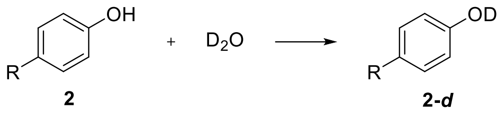

In the glovebox, phenol (1.5 mmol) was added into a Schlenk tube. Then, deuterated water (2 mL) was added to the tube and stirred at room temperature for 1 h under Ar. After that, the solvent was removed. The procedure was manipulated for another two times to afford the deuterated phenol.

Deuteration ratio: **2b-*d*** (98%D), **2c-*d*** (97.5%D), **2d-*d*** (96.5%D), **2e-*d*** (98%D), **2g-*d*** (99%D), **2h-*d*** (97.5%D), **2u-*d*** (97.5%D), **2w-*d*** (98%D).

Table S1: Effect of protecting group

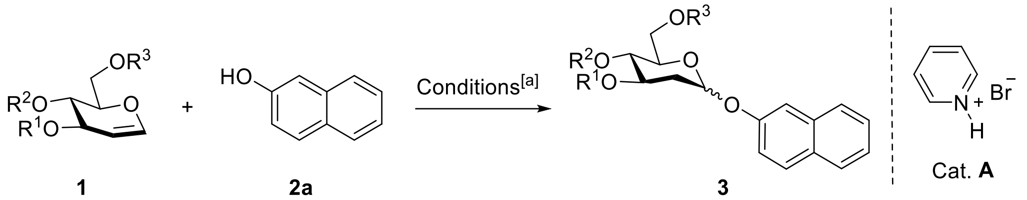

Entry	R^1	R^2	R^3	Yield (%)
1	Ac	Ac	Ac	N.R.
2	TIPS	^{i}Pr$_2$SiOSi^{i}Pr$_2$		78
3	TBS	TBS	TBS	Complex
4	Bn	Bn	Bn	Complex

[a]**1** (0.05 mmol), **2a** (0.05 mmol), 20 mol% catalyst **A** in toluene (0.05 M), 12 h, nitrogen, 70 °C; Yields were determined by crude ^{1}H NMR spectra analysis using 1,3,5-trimethoxybenzene as an internal standard. N.R. = no reaction; **1a**: $R^1 = R^2 = R^3$ = Ac, **1b**: R^1 = TIPS (Triisopropylsilyl), R^2, R^3 = ^{i}Pr$_2$SiOSi^{i}Pr$_2$, **1c**: $R^1 = R^2 = R^3$ = TBS, **1d**: $R^1 = R^2 = R^3$ = Bn.

Table S2: Conditions optimization

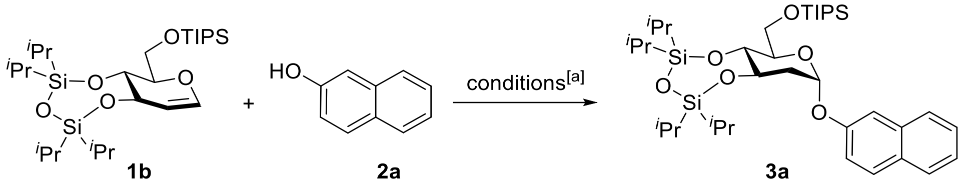

Entry	Catalyst	Solvent	Temp./°C	Conversion (%)	Yield (%)
1	**A**	toluene	70	78	78
2	**B**	toluene	70	66	63
3	**C**	toluene	70	86	79
4	**D**	toluene	70	38	36
5	**E**	toluene	70	82	68
6	**F**	toluene	70	38	37
7	**G**	toluene	70	100	76
8	**H**	toluene	70	91	63
9	**A**	$ClCH_2CH_2Cl$	70	83	68
10	**A**	THF	70	63	15
11	**A**	DMSO	70	37	0
12	**A**	toluene	RT	15	12
13	**A**	toluene	90	81	75
14	**none**	toluene	70	0	0
15	**I**	toluene	70	0	0
16	**$Et_3N·HCl$**	toluene	70	0	0
17[b]	**TfOH**	toluene	70	100	0
18	**$BF_3·Et_2O$**	toluene	70	100	0
19	**$TsOH·H_2O$**	toluene	70	100	0
20	**TMSOTf**	toluene	70	100	0
21	**Schreiner's catalyst**	toluene	70	0	0
22[c]	**A**	toluene	70	92	91

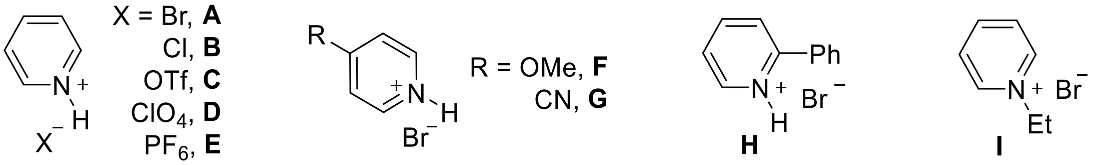

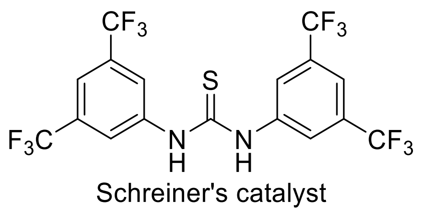

Schreiner's catalyst

[a]**1b** (0.05 mmol, 1 equiv.), **2a** (0.05 mmol, 1 equiv.), catalyst (20 mol%), temperature, solvent (0.05 M), 12 h, nitrogen; Conversions and yields were determined by crude ^1H NMR spectra analysis using 1,3,5-trimethoxybenzene as an internal standard based on the glycal **1b**. [b]1 mol%. [c]**2a** (1.5 equiv.) was used.

Experimental data for pyridinium salts

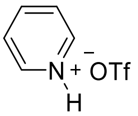

Cat. **C**

pyridin-1-ium trifluoromethanesulfonate[1]

Cat. **C** was synthesized according to the general procedure B, to a round-bottom flask equipped with a solution of pyridine (200 μL, 2.5 mmol) in ethyl ether (10 mL), HOTf (221 μL, 2.5 mmol) was added dropwise. The formation of precipitation was observed. When pyridine was consumed completely, the precipitation was filtered and washed with ethyl ether three times. Then the residue was dried under reduced pressure to afford the product as a white solid (135 mg, 59%). The final pyridinium salt was kept in nitrogen atmosphere.

^{1}H NMR (400 MHz, DMSO-*d_6*) δ 8.93 (d, *J* = 6.0 Hz, 2H), 8.61 (t, *J* = 8.0 Hz, 1H), 8.12-8.04 (m, 2H) ppm. **^{19}F NMR** (376 MHz DMSO-*d_6*) δ -77.73 (s, 3F) ppm.

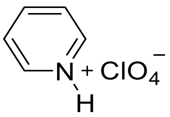

Cat. **D**

pyridin-1-ium perchlorate[2]

Cat. **D** was synthesized according to the general procedure B, to a round-bottom flask equipped with a solution of pyridine (200 μL, 2.5 mmol) in ethyl ether (10 mL), $HClO_4$ was added dropwise. The formation of precipitation was observed. When pyridine was consumed completely, the precipitation was filtered and washed with ethyl ether three times. Then the residue was dried under reduced pressure to afford the product as a white solid (158 mg, 88%). The final pyridinium salt was kept in nitrogen atmosphere.

^{1}H NMR (400 MHz, DMSO-*d_6*) δ 8.92 (d, *J* = 5.6 Hz, 2H), 8.60 (t, *J* = 8.4 Hz, 1H), 8.07 (t, *J* = 6.8 Hz, 2H) ppm.

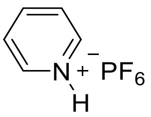

Cat. **E**

pyridin-1-ium hexafluorophosphate [3]

Cat. **E** was synthesized according to the general procedure B, to a round-bottom flask equipped with a solution of pyridine (200 μL, 2.5 mmol) in ethyl ether (10 mL), HPF_6 was added dropwise. The formation of precipitation was observed. When pyridine was consumed completely, the precipitation was filtered and washed with ethyl ether three times. Then the residue was dried under reduced pressure afford the product as a white solid (189 mg, 84%). The final pyridinium salt was kept in nitrogen atmosphere.

^{1}H NMR (400 MHz, DMSO-*d_6*) δ 8.90 (d, *J* = 5.2 Hz, 2H), 8.54 (tt, *J* = 7.6, 1.6 Hz, 1H), 8.02 (dd, *J* = 6.8, 1.2 Hz, 2H) ppm. **^{19}F NMR** (376 MHz DMSO-*d_6*) δ -70.11 (d, *J* = 709.9 Hz) ppm.

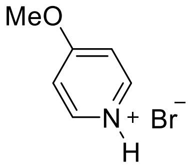

Cat. **F**

4-methoxypyridin-1-ium bromide[3]

Cat. **F** was synthesized according to the general procedure B, to a round-bottom flask equipped with a solution of 4-methoxypyridine (254 μL, 2.5 mmol) in ethyl ether (10 mL), HBr (40 wt% in water) was added dropwise. The formation of precipitation was observed. When pyridine was consumed completely, the precipitation was filtered and washed with ethyl ether three times. Then the residue was dried under reduced pressure to afford the product as a white solid (146 mg, 77%). The final pyridinium salt was kept in nitrogen atmosphere.

^{1}H NMR (400 MHz, DMSO-d_6) δ 8.78 (d, J = 7.2 Hz, 2H), 7.57 (d, J = 7.6 Hz, 2H), 4.09 (s, 3H) ppm.
^{13}C NMR (101 MHz, DMSO-d_6) δ 171.43, 143.34, 112.73, 57.80 ppm.

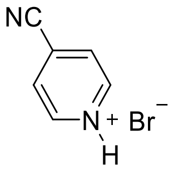

Cat. **G**

4-cyanopyridin-1-ium bromide

Cat. **G** was synthesized according to the general procedure B, to a round-bottom flask equipped with a solution of 4-cyanopyridine (260 mg, 2.5 mmol) in ethyl ether (10 mL), HBr (40 wt. % in water) was added dropwise. The formation of precipitation was observed. When pyridine was consumed completely, the precipitation was filtered and washed with ethyl ether three times. Then the residue was dried under reduced pressure to afford the product as a white solid (158 mg, 85%). The final pyridinium salt was kept in nitrogen atmosphere.

^{1}H NMR (400 MHz, DMSO-d_6) δ 8.89 (d, J = 5.2 Hz, 2H), 7.95 (d, J = 4.0 Hz, 2H) ppm. **^{13}C NMR** (101 MHz, DMSO-d_6) δ 150.09, 126.16, 120.38, 116.62 ppm. **ESI-HRMS:** Calculated for $C_6H_5N_2$ $(M-Br^-)^+$: 105.0447, Found: 105.0449.

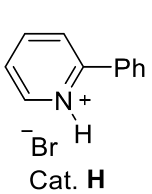

Cat. **H**

2-phenylpyridin-1-ium bromide

Cat. **H** was synthesized according to the general procedure B, to a round-bottom flask equipped with a solution of 2-phenylpyridine (357 μL, 2.5 mmol) in ethyl ether (10 mL), HBr (33 wt.% in acetic acid) was added dropwise. The formation of precipitation was observed. When pyridine was consumed completely, the precipitation was filtered and washed with ethyl ether three times. Then the residue was dried under reduced pressure to afford the product as a white solid (107 mg, 45%). The final pyridinium

salt was kept in nitrogen atmosphere.

^{1}H NMR (400 MHz, DMSO-d_6) δ 8.78 (d, J = 3.6 Hz, 1H), 8.23 (td, J = 8.0, 2.0 Hz, 1H), 8.17 (d, J = 8.0 Hz, 1H), 8.07 - 8.03 (m, 2H), 7.68 - 7.63 (m, 1H), 7.62 - 7.52 (m, 3H) ppm. **^{13}C NMR** (101 MHz, DMSO-d_6) δ 154.12, 146.60, 141.28, 135.47, 130.39, 129.12, 127.34, 123.91, 122.63 ppm. **ESI-HRMS:** Calculated for $C_{11}H_{10}N$ $(M\text{-}Br^-)^+$: 156.0808, Found: 156.0801.

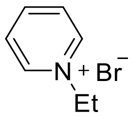

Cat. **I**

1-ethylpyridin-1-ium bromide[4]

A round-bottom flask charged with pyridine (80 µL, 1.0 mmol) and ethyl bromide (82 µL, 1.1 mmol) was added acetonitrile (10 mL). The mixture was stirred at reflux for 48 h under an argon atmosphere. Upon completion, the reaction mixture was allowed to cool to room temperature, the precipitate was filtered, and washed with diethyl ether, then dried in vacuo to afford the product as a gray solid (112 mg, 60%).

^{1}H NMR (400 MHz, DMSO-d_6) δ 9.20 - 9.11 (m, 2H), 8.61 (td, J = 8.0, 1.6 Hz, 1H), 8.17 (t, J = 6.8 Hz, 2H), 4.70-4.61 (m, 2H), 1.54 (t, J = 7.2 Hz, 3H) ppm. ^{13}C NMR (101 MHz, DMSO-d_6) δ 145.37, 144.54, 128.03, 56.25, 16.32.

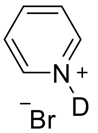

Cat. **J**

Cat. **J** was synthesized according to the following procedure. In the glovebox, Py·HBr (1.5 mmol) was added into a Schlenk tube. Then, deuterated water (2 mL) was added to the tube and stirred at room temperature for 1 h under Ar. After that, the solvent was removed. The procedure was manipulated for another two times to afford the deuterated compound (82.7 mg, 45%).

^{1}H NMR (400 MHz, DMSO-d_6): 8.93-8.91(m, 2H), 8.57-8.53 (m, 1H), 8.05-8.03 (m, 2H).

Experimental data for phenolic *O*-glycosides

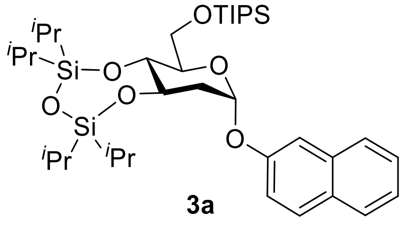

3a

(5a*R*,6*R*,8*R*,9a*R*)-2,2,4,4-tetraisopropyl-8-(naphthalen-2-yloxy)-6-(((triisopropylsilyl)oxy)methyl)tetrahydro-6*H*-pyrano[3,4-*f*][1,3,5,2,4]trioxadisilepine

3a was synthesized according to the general procedure C and isolated by column chromatography on silica gel using petroleum ether/ethyl acetate as the eluent, giving the titled product as a colorless syrup (63 mg, 91% yield).

¹H NMR (600 MHz, $CDCl_3$) δ 7.73 (d, *J* = 8.1 Hz, 1H), 7.70 (d, *J* = 8.9 Hz, 1H), 7.67 (d, *J* = 7.7 Hz, 1H), 7.49 (d, *J* = 2.4 Hz, 1H), 7.41 - 7.38 (m, 1H), 7.33 - 7.30 (m, 1H), 7.25 - 7.21 (m, 1H), 5.73 (d, *J* = 2.3 Hz, 1H), 4.26 (ddd, *J* = 11.3, 7.9, 5.2 Hz, 1H), 3.95 (dd, *J* = 11.0, 1.4 Hz, 1H), 3.87 - 3.81 (m, 1H), 3.68 - 3.64 (m, 2H), 2.33 (ddd, *J* = 13.6, 5.3, 1.3 Hz, 1H), 1.88 (ddd, *J* = 13.5, 11.4, 3.6 Hz, 1H), 1.14 - 0.91 (m, 49H) ppm. **¹³C NMR** (101 MHz, $CDCl_3$) δ 154.80, 134.63, 129.63, 129.24, 127.65, 127.31, 126.24, 124.01, 119.34, 110.80, 96.19, 74.37, 74.14, 71.75, 63.01, 38.41, 18.05, 17.82, 17.63, 17.61, 17.57, 17.51, 17.48, 17.43, 13.21, 13.11, 12.55, 12.44, 12.11 ppm. **IR (thin film, cm^{-1})**: 2941, 2865, 2323, 2167, 2505, 1980, 1465, 1387, 1254, 1111, 983, 883, 798, 698. **ESI-HRMS:** Calculated for $C_{37}H_{64}O_6NaSi_3$ $(M+Na)^+$: 711.3903, Found: 711.3884. **$[\alpha]_D^{25}$** = +123.7 (c = 0.32, $CHCl_3$).

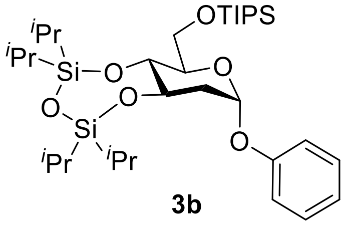

3b

(5a*R*,6*R*,8*R*,9a*R*)-2,2,4,4-tetraisopropyl-8-phenoxy-6-(((triisopropylsilyl)oxy)methyl)tetrahydro-6*H*-pyrano[3,4-*f*][1,3,5,2,4]trioxadisilepine

3b was synthesized according to the general procedure C and isolated by column chromatography on silica gel using petroleum ether/ethyl acetate as the eluent, giving the titled product as a colorless syrup (58 mg, 90% yield).

¹H NMR (400 MHz, $CDCl_3$) δ 7.24 (t, *J* = 7.6 Hz, 2H), 7.12 (d, *J* = 8.0 Hz, 2H), 6.97 (t, *J* = 7.2 Hz, 1H), 5.57 (d, *J* = 3.2 Hz, 1H), 4.27 - 4.19 (m, 1H), 3.99 (d, *J* = 10.8 Hz, 1H), 3.82 (dd, *J* = 10.8, 6.0 Hz, 1H), 3.73 - 3.66 (m, 1H), 3.60 (t, *J* = 8.4 Hz, 1H), 2.28 (dd, *J* = 13.6, 5.2 Hz, 1H), 1.89 - 1.79 (m, 1H), 1.16 - 0.95 (m, 49H) ppm. **¹³C NMR** (101 MHz, $CDCl_3$) δ 157.19, 129.42, 121.99, 117.05, 96.30, 74.55, 74.04, 71.66, 38.45, 18.10, 18.07, 17.79, 17.60, 17.56, 17.48, 17.46, 17.42, 13.18, 13.07, 12.52, 12.40, 12.11 ppm. **IR (thin film, cm^{-1})**: 2943, 2866, 2323, 2184, 2164, 2050, 1994, 1109, 985, 884. **ESI-HRMS:** Calculated for $C_{33}H_{62}O_6NaSi_3$ $(M+Na)^+$: 661.3746, Found: 661.3727. **$[\alpha]_D^{25}$** = +55.3 (c = 0.28, $CHCl_3$).

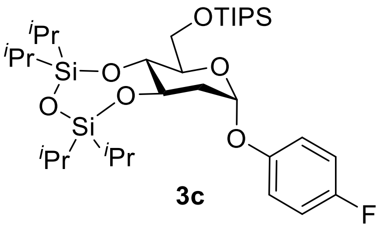

3c

(5a*R*,6*R*,8*R*,9a*R*)-8-(4-fluorophenoxy)-2,2,4,4-tetraisopropyl-6-(((triisopropylsilyl)oxy)methyl)tetrahydro-6*H*-pyrano[3,4-*f*][1,3,5,2,4]trioxadisilepine

3c was synthesized according to the general procedure C and isolated by column chromatography on silica gel using petroleum ether/ethyl acetate as the eluent, giving the titled product as a colorless syrup (53 mg, 81% yield).

¹H NMR (400 MHz, $CDCl_3$) δ 7.11 - 7.08 (m, 2H), 6.92 (t, *J* = 8.4 Hz, 2H), 5.48 (d, *J* = 3.2 Hz, 1H), 4.24 - 4.18 (m, 1H), 4.04 (d, *J* = 10.4 Hz, 1H), 3.83 - 3.79 (m, 1H), 3.74 - 3.70 (m, 1H), 3.55 (t, *J* = 8.8 Hz, 1H), 2.28 (dd, *J* = 13.6, 5.2 Hz, 1H), 1.88 - 1.78 (m, 1H), 1.15 - 0.99 (m, 49H) ppm. **¹³C NMR** (101 MHz, $CDCl_3$) δ 158.22 (d, *J* = 240.4 Hz), 153.30 (d, *J* = 2.0 Hz), 118.54 (d, *J* = 8.1 Hz), 115.75 (d, *J* =

23.2 Hz), 97.03, 74.69, 74.18, 71.56, 63.51, 38.41, 18.08, 18.06, 17.77, 17.57, 17.54, 17.46, 17.44, 17.40, 13.14, 13.06, 12.48, 12.38, 12.07 ppm. **^{19}F NMR** (376 MHz CDCl$_3$) δ -122.52 (m, 1F) ppm. **IR (thin film, cm^{-1})**: 2943, 2866, 2050, 1980, 1505, 1190, 1108, 985, 884, 829. **ESI-HRMS:** Calculated for $C_{33}H_{61}O_6FNaSi_3$ (M+Na)$^+$: 679.3652, Found: 679.3631. **[α]$_D$^{25}** = +94.6 (c = 0.28, CHCl$_3$).

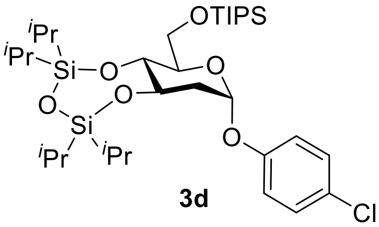

3d

(5a*R*,6*R*,8*R*,9a*R*)-8-(4-chlorophenoxy)-2,2,4,4-tetraisopropyl-6-(((triisopropylsilyl)oxy)methyl)tetrahydro-6*H*-pyrano[3,4-*f*][1,3,5,2,4]trioxadisilepine

3d was synthesized according to the general procedure C and isolated by column chromatography on silica gel using petroleum ether/ethyl acetate as the eluent, giving the titled product as a colorless syrup (59 mg, 87% yield).

^{1}H NMR (400 MHz, CDCl$_3$) δ 7.20 (d, *J* = 8.4 Hz, 2H), 7.06 (d, *J* = 8.4 Hz, 2H), 5.53 (s, 1H), 4.24 - 4.19 (m, 1H), 4.01 (d, *J* = 10.4 Hz, 1H), 3.83 - 3.79 (m, 1H), 3.68 - 3.64 (m, 1H), 3.60 - 3.58 (m, 1H), 2.29 (dd, *J* = 13.6, 5.6 Hz, 1H), 1.84 (t, *J* = 10.0 Hz, 1H), 1.13-1.03 (m, 49H) ppm. **^{13}C NMR** (101 MHz, CDCl$_3$) δ 155.70, 129.33, 127.02, 118.45, 96.53, 74.59, 74.29, 71.58, 38.32, 18.08, 18.06, 17.78, 17.58, 17.54, 17.47, 17.44, 17.41, 13.18, 13.09, 12.53, 12.44, 12.12. ppm. **IR (thin film, cm^{-1})**: 2943, 2866, 1489, 1463, 1231, 1193, 1108, 985, 884, 823, 699. **ESI-HRMS:** Calculated for $C_{33}H_{61}O_6ClNaSi_3$ (M+Na)$^+$: 695.3357, Found: 695.3334. **[α]$_D$^{25}** = +90.4 (c = 0.43, CHCl$_3$).

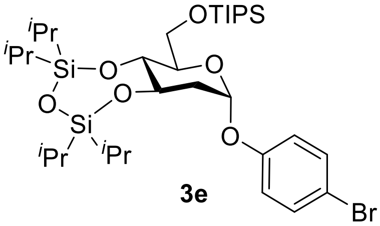

3e

(5a*R*,6*R*,8*R*,9a*R*)-8-(4-bromophenoxy)-2,2,4,4-tetraisopropyl-6-(((triisopropylsilyl)oxy)methyl)tetrahydro-6*H*-pyrano[3,4-*f*][1,3,5,2,4]trioxadisilepine

3e was synthesized according to the general procedure C and isolated by column chromatography on silica gel using petroleum ether/ethyl acetate as the eluent, giving the titled product as a colorless syrup (59 mg, 82% yield).

^{1}H NMR (400 MHz, CDCl$_3$) δ 7.34 (d, *J* = 8.8 Hz, 2H), 7.01 (d, *J* = 8.8 Hz, 2H), 5.53 (d, *J* = 3.6 Hz, 1H), 4.23 - 4.17 (m, 1H), 4.00 (d, *J* = 10.8 Hz, 1H), 3.82 - 3.78 (m, 1H), 3.66 - 3.63 (m, 1H), 3.60 - 3.55 (m, 1H), 2.28 (dd, *J* = 13.6, 5.2 Hz, 1H), 1.87 - 1.80 (m, 1H), 1.14 - 0.99 (m, 49H) ppm. **^{13}C NMR** (101 MHz, CDCl$_3$) δ 156.16, 132.28, 118.89, 114.40, 96.39, 74.53, 74.27, 71.55, 63.30, 38.27, 18.07, 18.05, 17.77, 17.58, 17.53, 17.47, 17.44, 17.40, 13.16, 13.07, 12.50, 12.41, 12.10. ppm. **IR (thin film, cm^{-1})**: 2943, 2866, 2323, 2161, 2050, 1979, 1486, 1463, 1231, 1193, 1110, 985, 884, 822, 700. **ESI-HRMS:** Calculated for $C_{33}H_{61}O_6BrNaSi_3$ (M+Na)$^+$: 739.2852, Found: 739.2830. **[α]$_D$^{25}** = +87.1 (c = 0.49, CHCl$_3$).

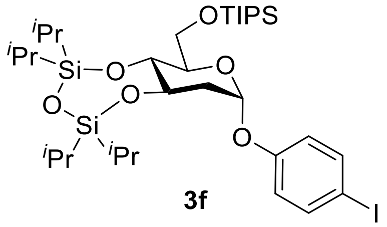

3f

(5a*R*,6*R*,8*R*,9a*R*)-8-(4-iodophenoxy)-2,2,4,4-tetraisopropyl-6-(((triisopropylsilyl)oxy)methyl)tetrahydro-6*H*-pyrano[3,4-*f*][1,3,5,2,4]trioxadisilepine

3f was synthesized according to the general procedure C and isolated by column chromatography on silica gel using petroleum ether/ethyl acetate as the eluent, giving the titled product as a colorless syrup (76 mg, 99% yield).

^{1}H NMR (400 MHz, $CDCl_3$) δ 7.52 (d, *J* = 8.4 Hz, 2H), 6.90 (d, *J* = 8.0 Hz, 2H), 5.54 (d, *J* = 3.6 Hz, 1H), 4.23 - 4.17 (m, 1H), 3.99 (d, *J* = 10.8 Hz, 1H), 3.82 - 3.78 (m, 1H), 3.67 - 3.54 (m, 2H), 2.27 (dd, *J* = 13.6, 5.2 Hz, 1H), 1.89 - 1.79 (m, 1H), 1.12 - 1.02 (m, 49H) ppm. **^{13}C NMR** (101 MHz, $CDCl_3$) δ 156.91, 138.29, 119.37, 96.22, 84.53, 74.51, 74.29, 71.57, 63.27, 38.26, 18.08, 18.06, 17.78, 17.58, 17.53, 17.47, 17.44, 17.41, 13.17, 13.08, 12.52, 12.43, 12.12 ppm. **IR (thin film, cm^{-1})**: 2942, 2866, 2323, 2162, 2050, 1979, 1585, 1483, 1463, 1383, 1295, 1231, 1193, 1108, 1047, 984, 883, 819, 800, 699. **ESI-HRMS:** Calculated for $C_{33}H_{61}O_6INaSi_3$ $(M+Na)^+$: 787.2713, Found: 787.2690. **$[\alpha]_D^{25}$** = +228.8 (c = 0.18, $CHCl_3$).

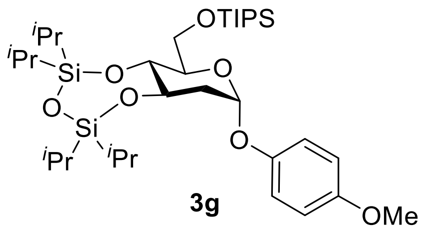

3g

(5a*R*,6*R*,8*R*,9a*R*)-2,2,4,4-tetraisopropyl-8-(4-methoxyphenoxy)-6-(((triisopropylsilyl)oxy)methyl)tetrahydro-6*H*-pyrano[3,4-*f*][1,3,5,2,4]trioxadisilepine

3g was synthesized according to the general procedure **C** and isolated by column chromatography on silica gel using petroleum ether/ethyl acetate as the eluent, giving the titled product as a colorless syrup (58 mg, 87% yield).

^{1}H NMR (400 MHz, $CDCl_3$) δ 7.08 (d, *J* = 8.8 Hz, 2H), 6.79 (d, *J* = 8.4 Hz, 2H), 5.44 (s, 1H), 4.26 - 4.19 (m, 1H), 4.04 (d, *J* = 10.4 Hz, 1H), 3.86 - 3.79 (m, 1H), 3.76 (s, 3H), 3.74 (s, 1H), 3.57 (t, *J* = 8.8 Hz, 1H), 2.28 (dd, *J* = 13.6, 5.6 Hz, 1H), 1.87 - 1.77 (m, 1H), 1.15 - 1.00 (m, 49H) ppm. **^{13}C NMR** (101 MHz, $CDCl_3$) δ 154.92, 151.38, 118.52, 114.57, 97.23, 74.79, 74.05, 71.68, 63.55, 55.79, 38.57, 18.12, 18.10, 17.78, 17.59, 17.55, 17.48, 17.46, 17.42, 13.19, 13.09, 12.54, 12.45, 12.15 ppm. **IR (thin film, cm^{-1})**: 2943, 2866, 2323, 2161, 2049, 1979, 1507, 1464, 1383, 1225, 1192, 1107, 1046, 986, 885, 826, 700. **ESI-HRMS:** Calculated for $C_{34}H_{64}O_7NaSi_3$ $(M+Na)^+$: 691.3852, Found: 691.3826. **$[\alpha]_D^{25}$** = +80.8 (c = 0.48, $CHCl_3$).

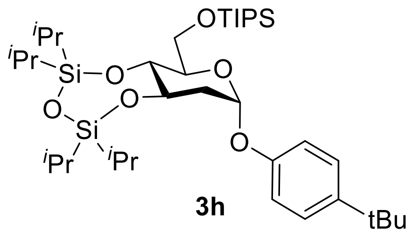

3h

(5a*R*,6*R*,8*R*)-8-(4-(tert-butyl)phenoxy)-2,2,4,4-tetraisopropyl-6-(((triisopropylsilyl)oxy)methyl)tetrahydro-6*H*-pyrano[3,4-f][1,3,5,2,4]trioxadisilepine

3h was synthesized according to the general procedure C and isolated by column chromatography on silica gel using petroleum ether/ethyl acetate as the eluent, giving the titled product as a colorless syrup (56 mg, 80%).

^{1}H NMR (500 MHz, $CDCl_3$) δ 7.28-7.25 (m, 2H), 7.08-7.04 (m, 2H), 5.56 (d, *J* = 2.3 Hz, 1H), 4.24 (ddd, *J* = 11.3, 8.2, 5.2 Hz, 1H), 4.02 (dd, *J* = 10.8, 2.0 Hz, 1H), 3.83 (dd, *J* = 10.8, 6.0 Hz, 1H), 3.72 (ddd, *J* = 9.7, 5.9, 1.9 Hz, 1H), 3.59 (dd, *J* = 9.5, 8.2 Hz, 1H), 2.28 (ddd, *J* = 13.3, 5.3, 1.3 Hz, 1H), 1.83 (ddd, *J* = 13.4, 11.4, 3.6 Hz, 1H), 1.30 (s, 9H), 1.21-0.88 (m, 49H). **^{13}C NMR** (126 MHz, $CDCl_3$) δ 154.87, 144.52, 126.19, 116.38, 96.24, 74.67, 73.95, 71.70, 63.40, 38.48, 34.22, 31.62, 18.10, 18.09, 17.80, 17.59, 17.56, 17.49, 17.46, 17.41, 13.17, 13.07, 12.52, 12.40, 12.10. **ESI-HRMS:** Calculated for $C_{37}H_{70}O_6Si_3$ $(M+H)^+$: 695.4553, Found: 695.4555. **$[\alpha]_D^{25}$** = +198.4 (c = 1, $CHCl_3$)

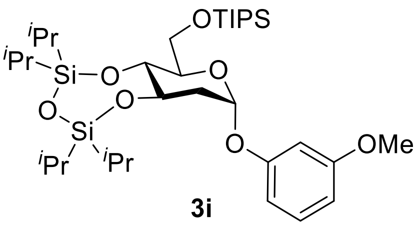

3i

(5a*R*,6*R*,8*R*,9a*R*)-2,2,4,4-tetraisopropyl-8-(3-methoxyphenoxy)-6-(((triisopropylsilyl)oxy)methyl)tetrahydro-6*H*-pyrano[3,4-*f*][1,3,5,2,4]trioxadisilepine

3i was synthesized according to the general procedure C and isolated by column chromatography on silica gel using petroleum ether/ethyl acetate as the eluent, giving the titled product as a colorless syrup (57 mg, 85% yield).

^{1}H NMR (400 MHz, $CDCl_3$) δ 7.14 (t, *J* = 8.4 Hz, 1H), 6.74 (d, *J* = 8.4 Hz, 1H), 6.66 (s, 1H), 6.54 (d, *J* = 8.0 Hz, 1H), 5.59 (s, 1H), 4.27 - 4.19 (m, 1H), 3.98 (d, *J* = 10.8 Hz, 1H), 3.85 (d, *J* = 11.6 Hz, 1H), 3.78 (s, 3H), 3.70 - 3.61 (m, 2H), 2.28 (dd, *J* = 13.6, 5.2 Hz, 1H), 1.88 - 1.78 (m, 1H), 1.16 - 0.99 (m, 49H) ppm. **^{13}C NMR** (101 MHz, $CDCl_3$) δ 160.81, 158.34, 129.88, 109.15, 107.79, 102.92, 96.19, 74.42, 74.13, 71.70, 63.13, 55.36, 38.41, 18.10, 18.07, 17.80, 17.63, 17.61, 17.56, 17.49, 17.46, 17.43, 13.22, 13.10, 12.57, 12.47, 12.16 ppm. **IR (thin film, cm^{-1})**: 2942, 2866, 2322, 2161, 2049, 1980, 1594, 1491, 1463, 1383, 1259, 1190, 1154, 1116, 1047, 985, 883, 798, 686. **ESI-HRMS:** Calculated for $C_{34}H_{64}O_7NaSi_3$ $(M+Na)^+$: 691.3852, Found: 691.3828. **$[\alpha]_D^{25}$** = +75.4 (c = 0.48, $CHCl_3$).

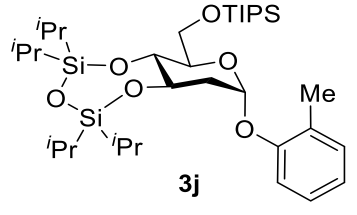

3j

(5a*R*,6*R*,8*R*,9a*R*)-2,2,4,4-tetraisopropyl-8-(o-tolyloxy)-6-(((triisopropylsilyl)oxy)methyl)tetrahydro-6*H*-pyrano[3,4-*f*][1,3,5,2,4]trioxadisilepine

3j was synthesized according to the general procedure C and isolated by column chromatography on silica gel using petroleum ether/ethyl acetate as the eluent, giving the titled product as a colorless syrup (46 mg, 70% yield).

^{1}H NMR (400 MHz, $CDCl_3$) δ 7.21 (d, *J* = 8.0 Hz, 1H), 7.13 - 7.05 (m, 2H), 6.88 (t, *J* = 7.2 Hz, 1H), 5.59 (s, 1H), 4.34 - 4.26 (m, 1H), 3.98 (d, *J* = 10.8 Hz, 1H), 3.84 (dd, *J* = 10.8, 4.8 Hz, 1H), 3.70 - 3.60 (m, 2H), 2.30 (dd, *J* = 13.6, 5.2 Hz, 1H), 2.21 (s, 3H), 1.90 - 1.81 (m, 1H), 1.18 - 0.92 (m, 49H) ppm. **^{13}C NMR** (101 MHz, $CDCl_3$) δ 155.17, 130.60, 127.24, 127.03, 121.67, 114.89, 96.03, 74.48, 74.29, 71.74, 63.23, 38.58, 18.11, 18.07, 17.72, 17.60, 17.56, 17.54, 17.44, 17.41, 16.26, 13.24, 13.09, 12.60, 12.42, 12.14 ppm. **IR (thin film, cm^{-1})**: 2942, 2866, 2323, 2187, 2161, 2050, 1979, 1492, 1463, 1240, 1188, 1109, 985, 885, 750, 699. **ESI-HRMS:** Calculated for $C_{34}H_{64}O_6NaSi_3$ $(M+Na)^+$: 675.3903, Found: 675.3884. **$[\alpha]_D^{25}$** = +51.3 (c = 0.29, $CHCl_3$).

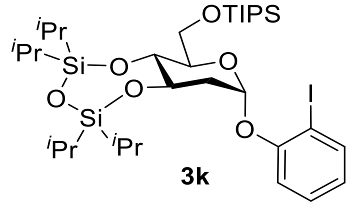

3k

(5a*R*,6*R*,8*R*,9a*R*)-8-(2-iodophenoxy)-2,2,4,4-tetraisopropyl-6-(((triisopropylsilyl)oxy)methyl)tetrahydro-6*H*-pyrano[3,4-*f*][1,3,5,2,4]trioxadisilepine

3k was synthesized according to the general procedure C and isolated by column chromatography on silica gel using petroleum ether/ethyl acetate as the eluent, giving the titled product as a colorless syrup (42 mg, 55% yield).

^{1}H NMR (400 MHz, $CDCl_3$) δ 7.74 (d, *J* = 7.6 Hz, 1H), 7.24 - 7.19 (m, 2H), 6.75 - 6.70 (m, 1H), 5.65 (d, *J* = 2.8 Hz, 1H), 4.43 (ddd, *J* = 11.2, 7.6, 4.8 Hz, 1H), 3.99 (dd, *J* = 11.2, 2.0 Hz, 1H), 3.81 (dd, *J* = 10.8, 5.6 Hz, 1H), 3.70 - 3.58 (m, 2H), 2.41 (dd, *J* = 13.2, 5.2 Hz, 1H), 1.90 - 1.80 (m, 1H), 1.18 - 0.9 (m, 49H) ppm. **^{13}C NMR** (101 MHz, $CDCl_3$) δ 155.49, 139.20, 129.59, 123.74, 115.70, 96.65, 87.60, 74.73, 74.51, 71.42, 63.29, 38.19, 18.09, 18.06, 17.96, 17.59, 17.57, 17.55, 17.52, 17.47, 17.43, 17.41, 13.22, 13.04, 12.52, 12.39, 12.08 ppm. **IR (thin film, cm^{-1})**: 2942, 2866, 2323, 2188, 2161, 2049, 2033, 1979, 1470, 1383, 1241, 1192, 1045, 984, 885, 748, 702. **ESI-HRMS:** Calculated for $C_{33}H_{61}O_6INaSi_3$ $(M+Na)^+$: 787.2713, Found: 787.2689. **$[\alpha]_D^{25}$** = +43.5 (c = 0.40, $CHCl_3$).

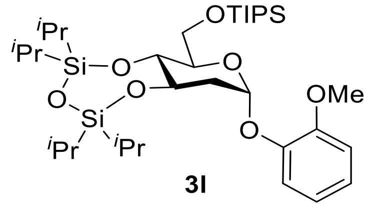

3l

(5a*R*,6*R*,8*R*,9a*R*)-2,2,4,4-tetraisopropyl-8-(2-methoxyphenoxy)-6-(((triisopropylsilyl)oxy)methyl)tetrahydro-6*H*-pyrano[3,4-*f*][1,3,5,2,4]trioxadisilepine

3l was synthesized according to the general procedure C with a reaction time of 96 h and isolated by column chromatography on silica gel using petroleum ether/ethyl acetate as the eluent, giving the titled product as a colorless syrup (36 mg, 54% yield).

^{1}H NMR (400 MHz, $CDCl_3$) δ 7.25 (dd, *J* = 8.0, 2.0 Hz, 1H), 7.00 - 6.94 (m, 1H), 6.90 - 6.82 (m, 2H), 5.53 (d, *J* = 3.3 Hz, 1H), 4.31 (ddd, *J* = 11.6, 8.4, 5.2 Hz, 1H), 4.04 (d, *J* = 8.8 Hz, 1H), 3.88 - 3.80 (m, 5H), 3.58 (t, *J* = 8.8 Hz, 1H), 2.44 - 2.37 (m, 1H), 1.83 (ddd, *J* = 13.6, 11.6, 3.6 Hz, 1H), 1.16 - 0.97 (m, 49H) ppm. **^{13}C NMR** (101 MHz, $CDCl_3$) δ 150.57, 146.60, 123.08, 121.30, 119.32, 112.92, 97.61, 74.74, 74.36, 71.61, 63.50, 56.32, 38.42, 18.12, 18.09, 17.78, 17.59, 17.55, 17.50, 17.46, 17.43, 13.18, 13.06, 12.54, 12.44, 12.11 ppm. **IR (thin film, cm^{-1})**: 2943, 2866, 2323, 2168, 2050, 1979, 1503, 1463, 1255, 1111, 985, 885, 699. **ESI-HRMS:** Calculated for $C_{34}H_{64}O_7NaSi_3$ $(M+Na)^+$: 691.3852, Found: 691.3828. **$[\alpha]_D^{25}$** = +80.4 (c = 0.23, $CHCl_3$).

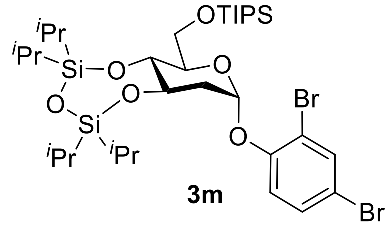

3m

(5a*R*,6*R*,8*R*,9a*R*)-8-(2,4-dibromophenoxy)-2,2,4,4-tetraisopropyl-6-(((triisopropylsilyl)oxy)methyl)tetrahydro-6*H*-pyrano[3,4-*f*][1,3,5,2,4]trioxadisilepine

3m was synthesized according to the general procedure C and isolated by column chromatography on silica gel using petroleum ether/ethyl acetate as the eluent, giving the titled product as a colorless syrup (42 mg, 53% yield).

^{1}H NMR (400 MHz, $CDCl_3$) δ 7.65 (d, *J* = 2.4 Hz, 1H), 7.30 (dd, *J* = 8.8, 2.4 Hz, 1H), 7.17 (d, *J* = 8.8 Hz, 1H), 5.60 (d, *J* = 3.2 Hz, 1H), 4.34 (ddd, *J* = 11.2, 8.0, 5.2 Hz, 1H), 4.00 (dd, *J* = 10.8, 2.0 Hz, 1H), 3.79 (dd, *J* = 10.8, 6.0 Hz, 1H), 3.67 - 3.61 (m, 1H), 3.60 - 3.54 (m, 1H), 2.39 (ddd, *J* = 13.2, 5.2, 1.2 Hz, 1H), 1.86 (ddd, *J* = 13.9, 11.2, 3.2 Hz, 1H), 1.17 - 0.94 (m, 49H) ppm. **^{13}C NMR** (101 MHz, $CDCl_3$) δ 152.54, 135.35, 131.40, 118.23, 114.53, 114.14, 96.92, 74.96, 74.45, 71.27, 63.31, 38.04, 18.06, 18.04, 17.76, 17.56, 17.52, 17.44, 17.43, 17.41, 17.39, 13.19, 13.03, 12.52, 12.38, 12.06 ppm. **IR (thin film, cm^{-1})**: 2943, 2866, 2049, 1981, 1470, 1383, 1241, 1192, 1110, 1043, 984, 827, 759, 699. **ESI-HRMS:** Calculated for $C_{33}H_{60}Br_2O_6NaSi_3$ $(M+Na)^+$: 819.1936, Found: 819.1917. **$[\alpha]_D^{25}$** = +60.1 (c = 0.63, $CHCl_3$).

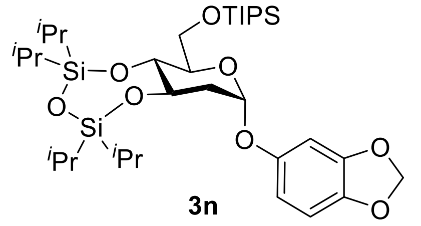

3n

(5a*R*,6*R*,8*R*,9a*R*)-8-(benzo[*d*][1,3]dioxol-5-yloxy)-2,2,4,4-tetraisopropyl-6-(((triisopropylsilyl)oxy)methyl)tetrahydro-6*H*-pyrano[3,4-*f*][1,3,5,2,4]trioxadisilepine

3n was synthesized according to the general procedure C and isolated by column chromatography on silica gel using petroleum ether/ethyl acetate as the eluent, giving the titled product as a white solid (63 mg, 92% yield).

^{1}H NMR (400 MHz, $CDCl_3$) δ 6.72 (s, 1H), 6.68 - 6.64 (m, 1H), 6.62 - 6.57 (m, 1H), 5.90 (s, 2H), 5.43 (s, 1H), 4.24 - 4.16 (m, 1H), 4.05 (d, J = 10.8 Hz, 1H), 3.86 - 3.78 (m, 1H), 3.73 (t, J = 8.8 Hz, 1H), 3.56 (t, J = 8.4 Hz, 1H), 2.27 (dd, J = 13.6, 5.2 Hz, 1H), 1.86 - 1.78 (m, 1H), 1.16 - 0.99 (m, 49H) ppm. **^{13}C NMR** (101 MHz, $CDCl_3$) δ 152.51, 148.09, 142.65, 109.49, 108.03, 101.24, 100.40, 97.36, 74.73, 74.17, 71.63, 63.51, 38.50, 18.10, 18.08, 17.78, 17.59, 17.54, 17.47, 17.45, 17.42, 13.18, 13.08, 12.53, 12.45, 12.13 ppm. **IR (thin film, cm^{-1})**: 2942, 2866, 2049, 1502, 1485, 1463, 1382, 1258, 1178, 1108, 1042, 833, 795, 699. **ESI-HRMS:** Calculated for $C_{34}H_{62}O_8NaSi_3$ $(M+Na)^+$: 705.3645, Found: 705.3622. **$[\alpha]_D^{25}$** = +68.4 (c = 0.25, $CHCl_3$).

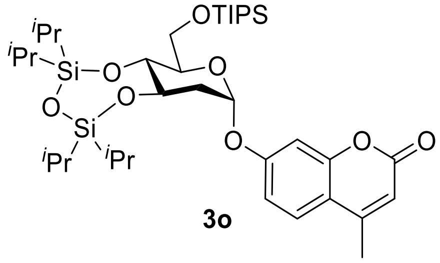

3o

4-methyl-7-(((5a*R*,6*R*,8*R*,9a*R*)-2,2,4,4-tetraisopropyl-6-(((triisopropylsilyl)oxy)methyl)tetrahydro-6*H*-pyrano[3,4-*f*][1,3,5,2,4]trioxadisilepin-8-yl)oxy)-2*H*-chromen-2-one

3o was synthesized according to the general procedure C by using toluene/THF (3:1 v/v) as the solvent and isolated by column chromatography on silica gel using petroleum ether/ethyl acetate as the eluent, giving the titled product as a white solid (43 mg, 60% yield).

^{1}H NMR (400 MHz, $CDCl_3$) δ 7.47 (d, J = 9.2 Hz, 1H), 7.08 - 7.03 (m, 2H), 6.15 (s, 1H), 5.68 (d, J = 3.6 Hz, 1H), 4.25 - 4.17 (m, 1H), 3.97 (d, J = 10.8 Hz, 1H), 3.79 (dd, J = 11.2, 4.4 Hz, 1H), 3.60 (d, J = 4.8 Hz, 2H), 2.39 (s, 3H), 2.32 (dd, J = 13.6, 5.6 Hz, 1H), 1.93 - 1.84 (m, 1H), 1.18 - 0.91 (m, 49H) ppm. **^{13}C NMR** (101 MHz, $CDCl_3$) δ 161.41, 159.72, 155.05, 152.44, 125.46, 114.60, 113.66, 112.62, 104.59, 96.13, 74.64, 74.31, 71.46, 63.11, 37.98, 18.79, 18.00, 17.98, 17.78, 17.56, 17.52, 17.46, 17.42, 17.39, 13.13, 13.06, 12.47, 12.39, 12.02 ppm. **IR (thin film, cm^{-1})**: 2926, 2866, 2323, 2186, 2165, 2049, 2033, 1992, 1735, 1613, 1463, 1387, 1260, 1115, 1067, 988, 844, 855, 798, 701. **ESI-HRMS:** Calculated for $C_{37}H_{64}O_8NaSi_3$ $(M+Na)^+$: 743.3801, Found: 743.3779. **$[\alpha]_D^{25}$** = +60.4 (c = 0.24, $CHCl_3$).

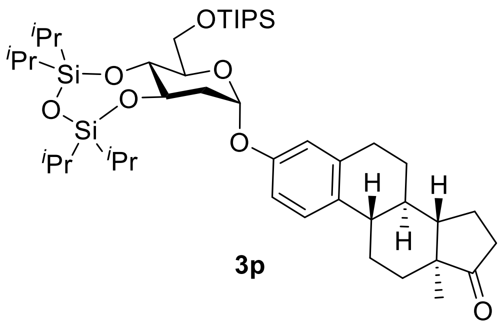

3p

(8*R*,9*S*,13*S*,14*S*)-13-methyl-3-(((5a*R*,6*R*,8*R*,9a*R*)-2,2,4,4-tetraisopropyl-6-(((triisopropylsilyl)oxy)methyl)tetrahydro-6*H*-pyrano[3,4-*f*][1,3,5,2,4]trioxadisilepin-8-yl)oxy)-6,7,8,9,11,12,13,14,15,16-decahydro-17*H*-cyclopenta[*a*]phenanthren-17-one

3p was synthesized according to the general procedure C with a reaction time of 36 h and isolated by column chromatography on silica gel using petroleum ether/ethyl acetate as the eluent, giving the titled product as a colorless syrup (74 mg, 91% yield).

^{1}H NMR (400 MHz, $CDCl_3$) δ 7.16 (d, *J* = 8.4 Hz, 1H), 6.91 (dd, *J* = 8.4, 2.4 Hz, 1H), 6.83 (d, *J* = 2.8 Hz, 1H), 5.57 (d, *J* = 3.2 Hz, 1H), 4.26 - 4.17 (m, 1H), 3.96 (d, *J* = 10.8 Hz, 1H), 3.87 - 3.80 (m, 1H), 3.68 - 3.59 (m, 2H), 2.91 - 2.79 (m, 2H), 2.50 (dd, *J* = 18.8, 8.8 Hz, 1H), 2.43 - 2.36 (m, 1H), 2.29 - 2.20 (m, 2H), 2.20 - 1.90 (m, 4H), 1.87 - 1.76 (m, 1H), 1.69 - 1.46 (m, 6H), 1.19 - 0.91 (m, 52H) ppm. **^{13}C NMR** (101 MHz, $CDCl_3$) δ 155.04, 137.64, 133.16, 126.32, 116.61, 114.42, 95.89, 74.35, 73.92, 71.70, 63.03, 50.59, 48.16, 44.18, 38.47, 38.37, 36.03, 31.73, 29.71, 26.72, 26.02, 21.74, 18.13, 18.10, 17.80, 17.60, 17.59, 17.55, 17.49, 17.46, 17.42, 13.99, 13.18, 13.06, 12.52, 12.40, 12.13 ppm.**IR (thin film, cm^{-1})**: 2932, 2865, 2286, 2162, 2049, 1743, 1497, 1463, 1383, 1246, 1109, 986, 884, 818, 702. **ESI-HRMS:** Calculated for $C_{45}H_{78}O_7Si_3Na$ $(M+Na)^+$: 837.4948, Found: 837.4924. **$[\alpha]_D^{25}$** = +101.7 (c = 0.29, $CHCl_3$).

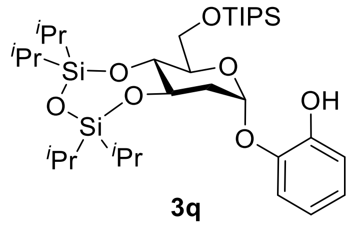

3q

2-(((5a*R*,6*R*,8*R*,9a*R*)-2,2,4,4-tetraisopropyl-6-(((triisopropylsilyl)oxy)methyl)tetrahydro-6*H*-pyrano[3,4-*f*][1,3,5,2,4]trioxadisilepin-8-yl)oxy)phenol

3q was synthesized according to the general procedure C and isolated by column chromatography on silica gel using petroleum ether/ethyl acetate as the eluent, giving the titled product as a white solid (62 mg, 94% yield).

^{1}H NMR (400 MHz, $CDCl_3$) δ 7.29 - 7.24 (m, 1H), 6.93 (d, *J* = 4.0 Hz, 2H), 6.81 - 6.74 (m, 1H), 5.89 (s, 1H), 5.50 (d, *J* = 3.5 Hz, 1H), 4.22 (ddd, *J* = 11.6, 8.4, 5.2 Hz, 1H), 4.07 (d, *J* = 10.0 Hz, 1H), 3.90 - 3.78 (m, 2H), 3.61 (t, *J* = 8.4 Hz, 1H), 2.34 (dd, *J* = 14.0, 5.6 Hz, 1H), 1.90 (ddd, *J* = 14.4, 11.6, 3.6 Hz, 1H), 1.15 - 0.99 (m, 49H) ppm. **^{13}C NMR** (101 MHz, $CDCl_3$) δ 146.49, 144.64, 123.58, 120.53, 117.46, 115.59, 98.57, 74.65, 74.41, 71.44, 63.30, 38.57, 18.10, 18.08, 17.79, 17.58, 17.56, 17.50, 17.45, 17.42, 17.39, 17.38, 13.14, 13.07, 12.49, 12.37, 12.09 ppm. **IR (thin film, cm^{-1})**: 2943, 2866, 2638, 2323, 2285, 2185, 2049, 1979, 1498, 1464, 1381, 1261, 1189, 1106, 1047, 986, 855, 798, 747, 700. **ESI-HRMS:** Calculated for $C_{33}H_{62}O_7NaSi_3$ $(M+Na)^+$: 677.3696, Found: 677.3674. **$[\alpha]_D^{25}$** = +43.1 (c = 0.16, $CHCl_3$).

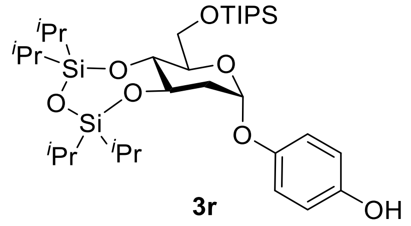

4-(((5a*R*,6*R*,8*R*,9a*R*)-2,2,4,4-tetraisopropyl-6-(((triisopropylsilyl)oxy)methyl)tetrahydro-6*H*-pyrano[3,4-*f*][1,3,5,2,4]trioxadisilepin-8-yl)oxy)phenol

3r was synthesized according to the general procedure C and isolated by column chromatography on silica gel using petroleum ether/ethyl acetate as the eluent, giving the titled product as a colorless syrup (48 mg, 74% yield).

^{1}H NMR (400 MHz, $CDCl_3$) δ 7.02 (d, *J* = 8.8 Hz, 2H), 6.71 (d, *J* = 8.4 Hz, 2H), 5.41 (s, 1H), 4.61 (s, 1H), 4.25 - 4.16 (m, 1H), 4.04 (d, *J* = 10.4 Hz, 1H), 3.86 - 3.72 (m, 2H), 3.55 (t, *J* = 8.8 Hz, 1H), 2.27 (dd, *J* = 13.2, 5.3 Hz, 1H), 1.85 - 1.76 (m, 1H), 1.13 - 0.99 (m, 49H) ppm. **^{13}C NMR** (101 MHz, $CDCl_3$) δ 151.44, 150.71, 118.82, 115.91, 97.36, 74.77, 74.05, 71.63, 63.57, 38.54, 18.11, 18.09, 17.77, 17.58, 17.54, 17.45, 17.41, 13.16, 13.06, 12.51, 12.41, 12.12 ppm. **IR (thin film, cm^{-1})**: 2942, 2866, 2648, 2323, 2288, 2163, 2050, 1979, 1508, 1462, 1190, 1109, 986, 885, 791, 692. **ESI-HRMS:** Calculated for $C_{33}H_{62}O_7NaSi_3$ $(M+Na)^+$: 677.3696, Found: 677.3677. **$[\alpha]_D^{25}$** = +58.8 (c = 0.18, $CHCl_3$).

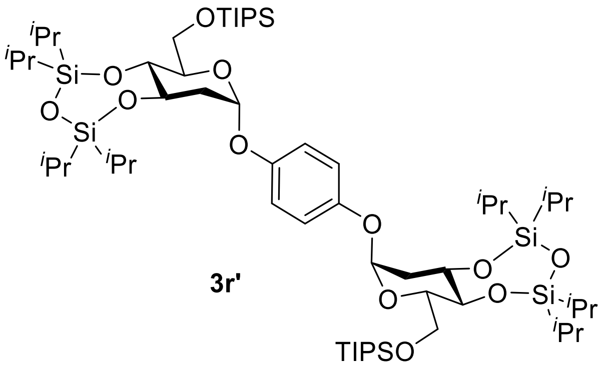

1,4-bis(((5a*R*,6*R*,8*R*,9a*R*)-2,2,4,4-tetraisopropyl-6-(((triisopropylsilyl)oxy)methyl)tetrahydro-6*H*-pyrano[3,4-*f*][1,3,5,2,4]trioxadisilepin-8-yl)oxy)benzene

3r' was synthesized according to the general procedure C and isolated by column chromatography on silica gel using petroleum ether/ethyl acetate as the eluent, giving the titled product as a colorless syrup (18 mg, 15% yield).

^{1}H NMR (400 MHz, $CDCl_3$) δ 6.99 (s, 4H), 5.46 (d, *J* = 3.6 Hz, 2H), 4.21 (ddd, *J* = 11.2, 8.0, 5.2 Hz, 2H), 3.97 (d, *J* = 10.8 Hz, 2H), 3.83 (dd, *J* = 10.8, 5.2 Hz, 2H), 3.71 - 3.65 (m, 2H), 3.61 (t, *J* = 8.8 Hz, 2H), 2.26 (dd, *J* = 13.2, 5.2 Hz, 2H), 1.85 - 1.76 (m, 2H), 1.15 - 0.96 (m, 98H) ppm. **^{13}C NMR** (101 MHz, $CDCl_3$) δ 152.15, 117.74, 96.78, 74.50, 73.92, 71.68, 63.22, 38.49, 18.12, 18.08, 17.79, 17.61, 17.56, 17.49, 17.46, 17.42, 13.19, 13.08, 12.54, 12.43, 12.13 ppm. **IR (thin film, cm^{-1})**: 2942, 2866, 2323, 2160, 2050, 1979, 1504, 1463, 1188, 1109, 986, 885, 823, 701. **ESI-HRMS:** Calculated for $C_{60}H_{118}O_{12}NaSi_6$ $(M+Na)^+$: 1221.7131, Found: 1221.7112. **$[\alpha]_D^{25}$** = +65.5 (c = 0.27, $CHCl_3$).

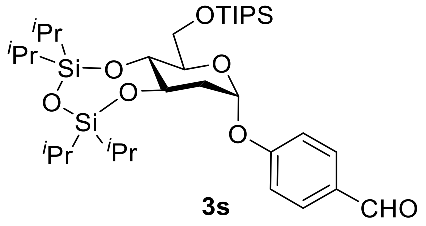

3s

4-(((5a*R*,6*R*,8*R*,9a*R*)-2,2,4,4-tetraisopropyl-6-(((triisopropylsilyl)oxy)methyl)tetrahydro-6*H*-pyrano[3,4-*f*][1,3,5,2,4]trioxadisilepin-8-yl)oxy)benzaldehyde

3s was synthesized according to the general procedure C and isolated by column chromatography on silica gel using petroleum ether/ethyl acetate as the eluent, giving the titled product as a colorless syrup (49 mg, 74% yield).

^{1}H NMR (400 MHz, $CDCl_3$) δ 9.89 (s, 1H), 7.80 (d, *J* = 8.4 Hz, 2H), 7.23 (d, *J* = 8.4 Hz, 2H), 5.71 (d, *J* = 3.2 Hz, 1H), 4.28 - 4.17 (m, 1H), 3.98 (d, *J* = 10.8 Hz, 1H), 3.81 (dd, *J* = 11.2, 4.0 Hz, 1H), 3.61 (d, *J* = 6.4 Hz, 2H), 2.32 (dd, *J* = 13.6, 5.2 Hz, 1H), 1.93 - 1.83 (m, 1H), 1.16 - 0.95 (m, 49H) ppm. **^{13}C NMR** (101 MHz, $CDCl_3$) δ 191.06, 161.99, 131.87, 130.92, 116.98, 96.09, 74.62, 74.38, 71.49, 63.16, 38.09, 18.04, 18.02, 17.77, 17.58, 17.56, 17.51, 17.46, 17.42, 17.39, 13.17, 13.09, 12.52, 12.43, 12.08 ppm. **IR (thin film, cm^{-1})**: 2942, 2866, 1701, 1601, 1580, 1463, 1383, 1306, 1246, 1193, 1161, 1107, 938, 883, 796, 758, 700. **ESI-HRMS:** Calculated for $C_{34}H_{62}O_7NaSi_3$ $(M+Na)^+$: 689.3696, Found: 689.3674. **$[\alpha]_D^{25}$** = +71.5 (c = 0.52, $CHCl_3$).

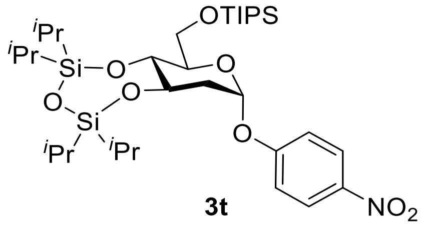

3t

(5a*R*,6*R*,8*R*,9a*R*)-2,2,4,4-tetraisopropyl-8-(4-nitrophenoxy)-6-(((triisopropylsilyl)oxy)methyl)tetrahydro-6*H*-pyrano[3,4-*f*][1,3,5,2,4]trioxadisilepine

3t was synthesized according to the general procedure C and isolated by column chromatography on silica gel using petroleum ether/ethyl acetate as the eluent, giving the titled product as a white solid (47 mg, 69% yield).

^{1}H NMR (400 MHz, $CDCl_3$) δ 8.16 (d, *J* = 8.8 Hz, 2H), 7.20 (d, *J* = 8.8 Hz, 2H), 5.70 (s, 1H), 4.24 - 4.16 (m, 1H), 3.99 (d, *J* = 10.8 Hz, 1H), 3.80 (dd, *J* = 11.6, 4.8 Hz, 1H), 3.63 - 3.55 (m, 2H), 2.33 (dd, *J* = 13.6, 5.2 Hz, 1H), 1.95 - 1.84 (m, 1H), 1.19 - 0.92 (m, 49H) ppm. **^{13}C NMR** (101 MHz, $CDCl_3$) δ 161.95, 142.43, 125.76, 116.77, 96.43, 74.84, 74.34, 71.40, 63.17, 37.98, 18.02, 18.01, 17.77, 17.57, 17.55, 17.50, 17.45, 17.41, 17.38, 13.15, 13.09, 12.50, 12.42, 12.06 ppm. **IR (thin film, cm^{-1})**: 2943, 2866, 2049, 1979, 1593, 1519, 1494, 1463, 1883, 1342, 1250, 1193, 984, 883, 795, 688. **ESI-HRMS:** Calculated for $C_{33}H_{61}NO_8NaSi_3$ $(M+Na)^+$: 706.3597, Found: 706.3578. **$[\alpha]_D^{25}$** = +101.9 (c = 0.26, $CHCl_3$).

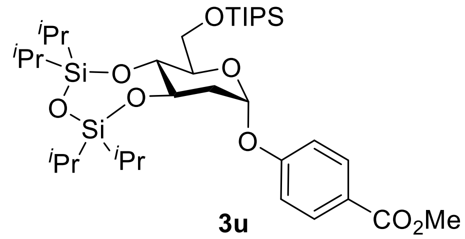

3u

methyl 4-(((5a*R*,6*R*,8*R*,9a*R*)-2,2,4,4-tetraisopropyl-6-(((triisopropylsilyl)oxy)methyl)tetrahydro-6*H*-pyrano[3,4-*f*][1,3,5,2,4]trioxadisilepin-8-yl)oxy)benzoate

3u was synthesized according to the general procedure C and isolated by column chromatography on silica gel using petroleum ether/ethyl acetate as the eluent, giving the titled product as a white solid (54 mg, 77% yield).

^{1}H NMR (400 MHz, $CDCl_3$) δ 7.95 (d, *J* = 8.4Hz, 2H), 7.13 (d, *J* = 8.4 Hz, 2H), 5.66 (d, *J* = 3.2 Hz, 1H), 4.26 - 4.18 (m, 1H), 3.96 (d, *J* = 11.2 Hz, 1H), 3.89 (s, 3H), 3.82 (dd, *J* = 11.6, 4.8 Hz, 1H), 3.65 - 3.57 (m, 2H), 2.30 (dd, *J* = 13.6, 5.2 Hz, 1H), 1.91 - 1.81 (m, 1H), 1.16 - 0.91 (m, 49H) ppm. **^{13}C NMR** (101 MHz, $CDCl_3$) δ 167.00, 161.29, 131.52, 123.72, 116.33, 96.02, 74.41, 74.35, 71.55, 63.08, 51.98, 38.17, 18.05, 18.03, 17.77, 17.59, 17.57, 17.53, 17.46, 17.43, 17.40, 13.18, 13.08, 12.52, 12.43, 12.10 ppm. **ESI-HRMS:** Calculated for $C_{35}H_{64}O_8NaSi_3$ $(M+Na)^+$: 719.3801, Found: 719.3775. **$[\alpha]_D^{25}$** = +88.6 (c = 0.29, $CHCl_3$).

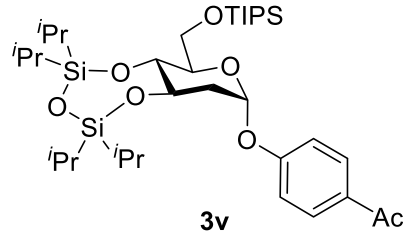

3v

1-(4-(((5a*R*,6*R*,8*R*,9a*R*)-2,2,4,4-tetraisopropyl-6-(((triisopropylsilyl)oxy)methyl)tetrahydro-6*H*-pyrano[3,4-*f*][1,3,5,2,4]trioxadisilepin-8-yl)oxy)phenyl)ethan-1-one

3v was synthesized according to the general procedure C and isolated by column chromatography on silica gel using petroleum ether/ethyl acetate as the eluent, giving the titled product as a colorless syrup (54 mg, 79% yield).

^{1}H NMR (400 MHz, $CDCl_3$) δ 7.89 (d, *J* = 8.8 Hz, 2H), 7.15 (d, *J* = 8.8 Hz, 2H), 5.68 (d, *J* = 3.2 Hz, 1H), 4.26 - 4.17 (m, 1H), 3.97 (d, *J* = 10.8 Hz, 1H), 3.81 (dd, *J* = 11.2, 4.8 Hz, 1H), 3.64 - 3.57 (m, 2H), 2.55 (s, 3H), 2.31 (dd, *J* = 13.6, 5.2 Hz, 1H), 1.92 - 1.82 (m, 1H), 1.16 - 0.90 (m, 49H) ppm. **^{13}C NMR** (101 MHz, $CDCl_3$) δ 197.01, 160.86, 131.33, 130.47, 116.39, 95.97, 74.48, 74.39, 71.53, 63.15, 38.13, 26.47, 18.05, 18.03, 17.77, 17.58, 17.56, 17.52, 17.46, 17.42, 17.39, 13.17, 13.08, 12.52, 12.43, 12.09 ppm. **IR (thin film, cm^{-1})**: 2942, 2866, 2323, 2166, 2049, 1979, 1684, 1600, 1506, 1463, 1356, 1245, 1193, 1107, 1046, 983, 883, 796, 699. **ESI-HRMS:** Calculated for $C_{35}H_{64}O_7NaSi_3$ $(M+Na)^+$: 703.3852, Found: 703.3828. **$[\alpha]_D^{25}$** = +82.9 (c = 0.44, $CHCl_3$).

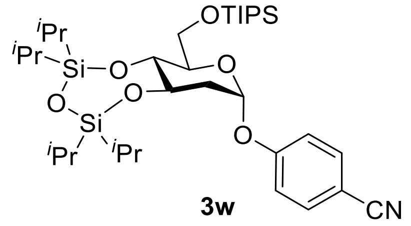

3w

4-(((5a*R*,6*R*,8*R*,9a*R*)-2,2,4,4-tetraisopropyl-6-(((triisopropylsilyl)oxy)methyl)tetrahydro-6*H*-pyrano[3,4-*f*][1,3,5,2,4]trioxadisilepin-8-yl)oxy)benzonitrile

3w was synthesized according to the general procedure C and isolated by column chromatography on silica gel using petroleum ether/ethyl acetate as the eluent, giving the titled product as a white solid (63 mg, 95% yield).

^{1}H NMR (400 MHz, $CDCl_3$) δ 7.55 (d, *J* = 8.4 Hz, 2H), 7.18 (d, *J* = 8.4 Hz, 2H), 5.65 (d, *J* = 3.6 Hz, 1H), 4.22 - 4.15 (m, 1H), 3.99 (d, *J* = 10.8 Hz, 1H), 3.78 (dd, *J* = 11.2, 4.8 Hz, 1H), 3.62 - 3.53 (m, 2H), 2.30 (dd, *J* = 13.6, 5.2 Hz, 1H), 1.91 - 1.83 (m, 1H), 1.17 - 0.93 (m, 49H) ppm. **^{13}C NMR** (101 MHz, $CDCl_3$) δ 160.26, 133.93, 119.30, 117.48, 105.20, 96.21, 74.69, 74.39, 71.40, 63.24, 38.01, 18.02, 18.01, 17.76, 17.55, 17.50, 17.45, 17.41, 17.38, 13.13, 13.06, 12.47, 12.38, 12.04 ppm. **IR (thin film, cm^{-1})**: 2943, 2866, 2225, 2050, 1979, 1605, 1507, 1463, 1383, 1248, 1193, 1172, 1108, 1046, 984, 883, 831, 796, 699. **ESI-HRMS:** Calculated for $C_{34}H_{61}NO_6NaSi_3$ $(M+Na)^+$: 686.3699, Found: 686.3678. **$[\alpha]_D^{25}$** = +115.7 (c = 0.21, $CHCl_3$).

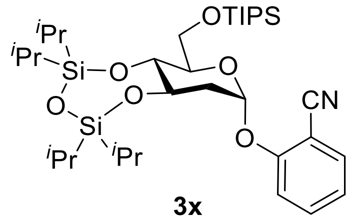

3x

2-(((5a*R*,6*R*,8*R*,9a*R*)-2,2,4,4-tetraisopropyl-6-(((triisopropylsilyl)oxy)methyl)tetrahydro-6*H*-pyrano[3,4-*f*][1,3,5,2,4]trioxadisilepin-8-yl)oxy)benzonitrile

3x was synthesized according to the general procedure C and isolated by column chromatography on silica gel using petroleum ether/ethyl acetate as the eluent, giving the titled product as a white solid (48 mg, 72% yield).

^{1}H NMR (400 MHz, $CDCl_3$) δ 7.55 (dd, *J* = 7.6, 1.6 Hz, 1H), 7.49 - 7.43 (m, 1H), 7.40 (d, *J* = 8.4 Hz, 1H), 7.03 (t, *J* = 7.2 Hz, 1H), 5.69 (d, *J* = 3.2 Hz, 1H), 4.39 - 4.29 (m, 1H), 4.02 (dd, *J* = 10.8, 1.6 Hz, 1H), 3.81 (dd, *J* = 11.2, 6.4 Hz, 1H), 3.72 - 3.64 (m, 1H), 3.58 (t, *J* = 8.4 Hz, 1H), 2.42 (dd, *J* = 13.2, 5.2 Hz, 1H), 1.94 - 1.82 (m, 1H), 1.15 - 0.93 (m, 49H) ppm. **^{13}C NMR** (101 MHz, $CDCl_3$) δ 158.69, 134.28, 133.46, 122.06, 116.14, 116.04, 103.27, 97.16, 75.05, 74.47, 71.13, 63.35, 38.07, 18.06, 18.04, 17.64, 17.59, 17.55, 17.51, 17.44, 17.39, 13.19, 13.01, 12.53, 12.45, 12.08 ppm. **IR (thin film, cm^{-1})**: 2943, 2866, 2322, 2161, 2049, 1980, 1104, 985, 884. **ESI-HRMS:** Calculated for $C_{34}H_{61}NO_6NaSi_3$ $(M+Na)^+$: 686.3699, Found: 686.3674. **$[\alpha]_D^{25}$** = +41.6 (c = 0.18, $CHCl_3$).

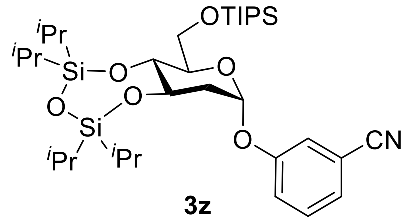

3z

3-(((5a*R*,6*R*,8*R*,9a*R*)-2,2,4,4-tetraisopropyl-6-(((triisopropylsilyl)oxy)methyl)tetrahydro-6*H*-pyrano[3,4-*f*][1,3,5,2,4]trioxadisilepin-8-yl)oxy)benzonitrile

3z was synthesized according to the general procedure C and isolated by column chromatography on silica gel using petroleum ether/ethyl acetate as the eluent, giving the titled product as a colorless syrup (52 mg, 78% yield).

^{1}H NMR (400 MHz, $CDCl_3$) δ 7.42 - 7.30 (m, 3H), 7.27 (d, *J* = 6.4 Hz, 1H), 5.61 (s, 1H), 4.24 - 4.15 (m, 1H), 4.01 (d, *J* = 10.8 Hz, 1H), 3.83 - 3.76 (m, 1H), 3.65 - 3.54 (m, 2H), 2.30 (dd, *J* = 14.4, 5.2 Hz, 1H), 1.93 - 1.82 (m, 1H), 1.15 - 0.97 (m, 49H) ppm. **^{13}C NMR** (101 MHz, $CDCl_3$) δ 157.11, 130.34, 125.68, 121.76, 120.12, 118.70, 113.32, 96.43, 74.65, 74.43, 71.46, 63.23, 38.11, 18.03, 17.77, 17.55, 17.51, 17.45, 17.41, 17.39, 13.15, 13.08, 12.49, 12.41, 12.06 ppm. **IR (thin film, cm^{-1})**: 2943, 2866, 1463, 1256, 1109, 982, 883, 791, 681. **ESI-HRMS:** Calculated for $C_{34}H_{61}NO_6NaSi_3$ $(M+Na)^+$: 686.3699, Found: 686.3675. **$[\alpha]_D^{25}$** = +86.4 (c = 0.53, $CHCl_3$).

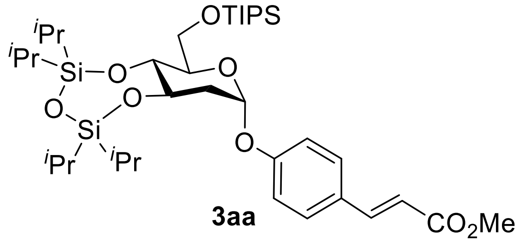

3aa

methyl (E)-3-(4-(((5a*R*,6*R*,8*R*,9a*R*)-2,2,4,4-tetraisopropyl-6-(((triisopropylsilyl)oxy)methyl)tetrahydro-6*H*-pyrano[3,4-*f*][1,3,5,2,4]trioxadisilepin-8-yl)oxy)phenyl)acrylate

3aa was synthesized according to the general procedure C and isolated by column chromatography on silica gel using petroleum ether/ethyl acetate as the eluent, giving the titled product as a white solid (59 mg, 81% yield).

^{1}H NMR (400 MHz, $CDCl_3$) δ 7.64 (d, *J* = 15.9 Hz, 1H), 7.43 (d, *J* = 8.0 Hz, 2H), 7.12 (d, *J* = 8.0 Hz, 2H), 6.31 (d, *J* = 15.6 Hz, 1H), 5.63 (s, 1H), 4.25 - 4.16 (m, 1H), 3.99 (d, *J* = 10.8 Hz, 1H), 3.84 - 3.76 (m, 4H), 3.66 - 3.55 (m, 2H), 2.29 (dd, *J* = 13.6, 5.2 Hz, 1H), 1.90 - 1.80 (m, 1H), 1.17 - 0.91 (m, 49H) ppm. **^{13}C NMR** (101 MHz, $CDCl_3$) δ 167.93, 158.79, 144.76, 129.64, 128.22, 117.17, 115.71, 96.04, 74.45, 74.31, 71.53, 63.23, 51.75, 38.18, 18.05, 18.03, 17.77, 17.57, 17.53, 17.46, 17.43, 17.39, 13.14, 13.06, 12.48, 12.38, 12.06 ppm. **IR (thin film, cm^{-1})**: 2943, 2866, 1981,1721, 1509, 1116, 1110, 984, 885, 824, 700. **ESI-HRMS:** Calculated for $C_{37}H_{66}O_8NaSi_3$ $(M+Na)^+$: 745.3958, Found: 745.3945. **$[\alpha]_D^{25}$** = +100.0 (c = 0.17, $CHCl_3$).

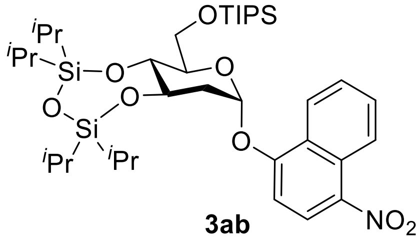

3ab

(5a*R*,6*R*,8*R*,9a*R*)-2,2,4,4-tetraisopropyl-8-((4-nitronaphthalen-1-yl)oxy)-6-(((triisopropylsilyl)oxy)methyl)tetrahydro-6*H*-pyrano[3,4-*f*][1,3,5,2,4]trioxadisilepine

3ab was synthesized according to the general procedure C and isolated by column chromatography on silica gel using petroleum ether/ethyl acetate as the eluent, giving the titled product as a yellow syrup (29 mg, 40% yield).

^{1}H NMR (400 MHz, $CDCl_3$) δ 8.76 (d, *J* = 8.8 Hz, 1H), 8.31 (dd, *J* = 18.0, 9.2 Hz, 2H), 7.74 (t, *J* = 7.6 Hz, 1H), 7.61 (t, *J* = 7.6 Hz, 1H), 7.32 (d, *J* = 8.8 Hz, 1H), 5.92 (s, 1H), 4.45 - 4.36 (m, 1H), 3.98 (d, *J* = 10.8 Hz, 1H), 3.83 (dd, *J* = 11.6, 5.6 Hz, 1H), 3.70 (t, *J* = 9.2 Hz, 1H), 3.65 - 3.56 (m, 1H), 2.51 (dd, *J* = 13.6, 4.8 Hz, 1H), 2.06 - 1.96 (m, 1H), 1.15 - 0.92 (m, 49H) ppm. **^{13}C NMR** (101 MHz, $CDCl_3$) δ 157.45, 140.18, 130.00, 127.04, 127.01, 126.86, 126.03, 123.76, 122.47, 106.80, 96.98, 75.20, 74.22, 71.66, 63.04, 38.20, 18.02, 18.00, 17.83, 17.58, 17.54, 17.50, 17.42, 17.38, 13.19, 12.59, 12.43, 12.07 ppm. **IR (thin film, cm^{-1})**: 2944, 2866, 2322, 2165, 2049, 1980, 1109, 988, 765. **ESI-HRMS:** Calculated for $C_{37}H_{63}NO_8NaSi_3$ $(M+Na)^+$: 756.3754, Found: 756.3736. **$[\alpha]_D^{25}$** = +59.1 (c = 0.22, $CHCl_3$).

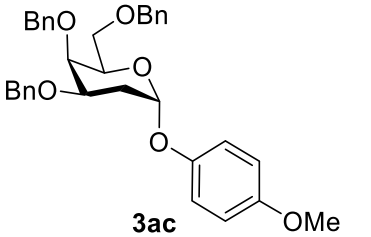

3ac

(2*R*,3*R*,4*R*,6*R*)-3,4-bis(benzyloxy)-2-((benzyloxy)methyl)-6-(4-methoxyphenoxy)tetrahydro-2*H*-pyran[S5]

3ac was synthesized according to the general procedure C and isolated by column chromatography on silica gel using petroleum ether/ethyl acetate as the eluent, giving the titled product as a colorless syrup (31 mg, 56% yield).

^{1}H NMR (400 MHz, $CDCl_3$) δ 7.44 - 7.21 (m, 15H), 7.01 (d, *J* = 9.2 Hz, 2H), 6.80 (d, *J* = 8.8 Hz, 2H), 5.61 (d, *J* = 3.2 Hz, 1H), 4.99 (d, *J* = 11.6 Hz, 1H), 4.73 - 4.64 (m, 3H), 4.41 (q, *J* = 11.6 Hz, 2H), 4.18 - 4.08 (m, 2H), 4.03 (s, 1H), 3.76 (s, 3H), 3.69 - 3.62 (m, 1H), 3.61 - 3.54 (m, 1H), 2.40 (td, *J* = 12.4, 3.6 Hz, 1H), 2.22 (dd, *J* = 12.8, 4.8 Hz, 1H) ppm. **^{13}C NMR** (101 MHz, $CDCl_3$) δ 154.84, 151.03, 138.93, 138.59, 138.18, 128.57, 128.44, 128.37, 128.36, 127.86, 127.74, 127.72, 127.68, 127.47, 118.14, 114.61, 97.53, 74.71, 74.52, 73.46, 73.04, 70.67, 69.48, 55.72, 31.44 ppm. **ESI-HRMS:** Calculated for $C_{34}H_{36}O_6Na$ $(M+Na)^+$: 563.2404, Found: 563.2389. **$[\alpha]_D^{25}$** = +73.0 (c = 0.20, $CHCl_3$).

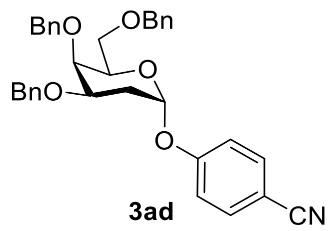

3ad

4-(((2*R*,4*R*,5*R*,6*R*)-4,5-bis(benzyloxy)-6-((benzyloxy)methyl)tetrahydro-2*H*-pyran-2-yl)oxy)benzonitrile

3ad was synthesized according to the general procedure C and isolated by column chromatography on silica gel using petroleum ether/ethyl acetate as the eluent, giving the titled product as a colorless syrup (41 mg, 76% yield).

^{1}H NMR (400 MHz, $CDCl_3$) δ 7.55 (d, *J* = 8.8 Hz, 2H), 7.43 - 7.26 (m, 13H), 7.22 - 7.18 (m, 2H), 7.12 (d, *J* = 8.8 Hz, 2H), 5.77 (d, *J* = 3.6 Hz, 1H), 4.99 (d, *J* = 11.6 Hz, 1H), 4.70 (s, 2H), 4.65 (d, *J* = 11.2 Hz, 1H), 4.39 (q, *J* = 11.6 Hz, 2H), 4.14 - 4.08 (m, 1H), 4.02 (s, 1H), 3.95 (t, *J* = 6.4 Hz, 1H), 3.67 - 3.59 (m, 1H), 3.56 - 3.50 (m, 1H), 2.45 (td, *J* = 12.8, 4.0 Hz, 1H), 2.23 (dd, *J* = 12.8, 4.4 Hz, 1H) ppm. **^{13}C NMR** (101 MHz, $CDCl_3$) δ 160.18, 138.66, 138.31, 137.87, 133.98, 128.60, 128.47, 128.41, 128.33, 127.88, 127.82, 127.77, 127.45, 119.18, 117.14, 105.16, 96.66, 74.59, 74.26, 73.47, 72.63, 71.27, 70.71, 69.14, 30.98 ppm. **IR (thin film, cm^{-1})**: 3030, 2942, 28630, 2647, 2322, 2223, 1980, 1742, 1603, 1506, 1453, 1361, 1247, 1172, 1097, 1056, 1027, 737, 697. **ESI-HRMS:** Calculated for $C_{34}H_{33}NO_5Na$ $(M+Na)^+$: 558.2251, Found: 558.2234. **[α]$_D$^{25}** = +73.2 (c = 0.34, $CHCl_3$).

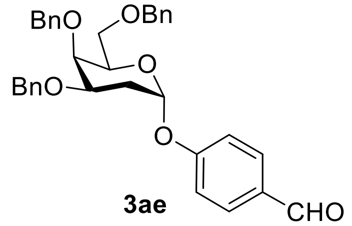

3ae

4-(((2*R*,4*R*,5*R*,6*R*)-4,5-bis(benzyloxy)-6-((benzyloxy)methyl)tetrahydro-2*H*-pyran-2-yl)oxy)benzaldehyde

3ae was synthesized according to the general procedure C and isolated by column chromatography on silica gel using petroleum ether/ethyl acetate as the eluent, giving the titled product as a colorless syrup (40 mg, 75% yield).

^{1}H NMR (400 MHz, $CDCl_3$) δ 9.89 (s, 1H), 7.81 (d, *J* = 8.8 Hz, 2H), 7.44 - 7.25 (m, 13H), 7.22 - 7.15 (m, 4H), 5.83 (d, *J* = 3.6 Hz, 1H), 4.99 (d, *J* = 11.6 Hz, 1H), 4.70 (s, 2H), 4.66 (d, *J* = 11.2 Hz, 1H), 4.39 (q, *J* = 11.6 Hz, 2H), 4.17 - 4.09 (m, 1H), 4.04 (s, 1H), 3.98 (t, *J* = 6.8 Hz, 1H), 3.68 - 3.61 (m, 1H), 3.56 - 3.50 (m, 1H), 2.46 (td, *J* = 12.8, 3.6 Hz, 1H), 2.24 (dd, *J* = 12.8, 4.4 Hz, 1H) ppm. **^{13}C NMR** (101 MHz, $CDCl_3$) δ 190.99, 161.88, 138.75, 138.38, 137.94, 131.92, 130.84, 128.60, 128.45, 128.41, 128.32, 127.82, 127.79, 127.76, 127.48, 116.69, 96.54, 74.60, 74.36, 73.45, 72.74, 71.24, 70.73, 69.14, 31.04 ppm. **IR (thin film, cm^{-1})**: 3030, 2922, 2854, 2323, 2164, 2050, 1980, 1693, 1599, 1577, 1506, 1359, 1307, 1255, 1161, 1093, 1054, 1025, 904, 861, 796, 734, 696, 662. **ESI-HRMS:** Calculated for $C_{34}H_{34}O_6Na$ $(M+Na)^+$: 561.2248, Found: 561.2231. **[α]$_D$^{25}** = +38.4 (c = 0.39, $CHCl_3$).

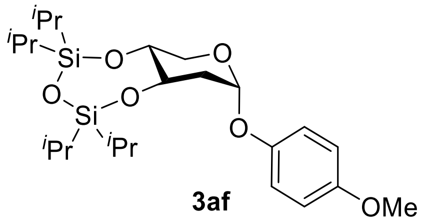

(5a*R*,8*R*,9a*S*)-2,2,4,4-tetraisopropyl-8-(4-methoxyphenoxy)tetrahydro-6*H*-pyrano[3,4-*f*][1,3,5,2,4]trioxadisilepine

3af was synthesized according to the general procedure C and isolated by column chromatography on silica gel using petroleum ether/ethyl acetate as the eluent, giving the titled product as a colorless syrup (36 mg, 75% yield).

^{1}H NMR (400 MHz, $CDCl_3$) δ 6.99 (d, *J* = 9.2 Hz, 2H), 6.82 (d, *J* = 8.8 Hz, 2H), 5.53 - 5.49 (m, 1H), 4.56 - 4.49 (m, 1H), 4.16 (s, 1H), 3.95 - 3.88 (m, 1H), 3.77 (s, 3H), 3.73 (dd, *J* = 12.0, 4.4 Hz, 1H), 2.27 - 2.18 (m, 1H), 2.02 (dt, *J* = 12.8, 4.0 Hz, 1H), 1.14 - 0.94 (m, 28H) ppm. **^{13}C NMR** (101 MHz, $CDCl_3$) δ 154.76, 151.21, 117.56, 114.65, 97.41, 70.59, 68.95, 64.52, 55.79, 35.07, 17.79, 17.73, 17.60, 17.52, 17.43, 17.35, 17.31, 14.08, 13.71, 13.50, 12.97 ppm. **IR (thin film, cm^{-1})**: 2944, 2866, 2642, 2323, 2288, 2161, 2049, 1979, 1507, 1463, 1226, 1119, 1024, 1009, 885, 824, 693. **ESI-HRMS:** Calculated for $C_{24}H_{42}O_6Si_2Na$ $(M+Na)^+$: 505.2412, Found: 505.2399. **$[\alpha]_D^{25}$** = -61.6 (c = 0.24, $CHCl_3$).

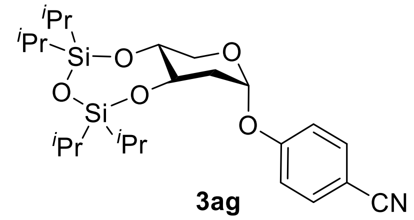

4-(((5a*R*,8*R*,9a*S*)-2,2,4,4-tetraisopropyltetrahydro-6*H*-pyrano[3,4-*f*][1,3,5,2,4]trioxadisilepin-8-yl)oxy)benzonitrile

3ag was synthesized according to the general procedure C and isolated by column chromatography on silica gel using petroleum ether/ethyl acetate as the eluent, giving the titled product as a white solid (24 mg, 50% yield).

^{1}H NMR (400 MHz, $CDCl_3$) δ 7.58 (d, *J* = 8.8 Hz, 2H), 7.10 (d, *J* = 8.8 Hz, 2H), 5.69 - 5.65 (m, 1H), 4.51 (dt, *J* = 9.6, 3.2 Hz, 1H), 4.18 (s, 1H), 3.86 - 3.80 (m, 1H), 3.75 (dd, *J* = 11.6, 4.0 Hz, 1H), 2.26 (ddd, *J* = 13.2, 10.0, 3.2 Hz, 1H), 2.05 (dt, *J* = 13.2, 4.0 Hz, 1H), 1.12 - 0.98 (m, 28H) ppm. **^{13}C NMR** (101 MHz, $CDCl_3$) δ 160.34, 134.06, 119.22, 116.95, 105.18, 96.75, 70.21, 68.63, 64.93, 34.44, 17.76, 17.71, 17.57, 17.49, 17.41, 17.32, 17.28, 14.11, 13.72, 13.42, 12.91 ppm. **IR (thin film, cm^{-1})**: 2944, 2866, 2226, 2162, 2050, 1980, 1604, 1506, 1463, 1384, 1305, 1254, 1170, 1124, 1093, 1057, 1019, 1006, 897, 884, 861, 798, 693, 602, 546. **ESI-HRMS:** Calculated for $C_{24}H_{39}O_5NSi_2Na$ $(M+Na)^+$: 500.2259, Found: 500.2249. **$[\alpha]_D^{25}$** = -83.7 (c = 0.24, $CHCl_3$).

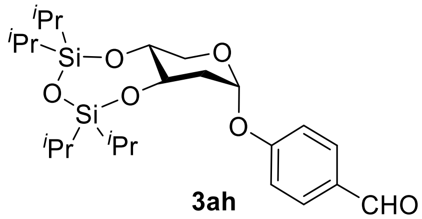

3ah

4-(((5a*R*,8*R*,9a*S*)-2,2,4,4-tetraisopropyltetrahydro-6*H*-pyrano[3,4-*f*][1,3,5,2,4]trioxadisilepin-8-yl)oxy)benzaldehyde

3ah was synthesized according to the general procedure C and isolated by column chromatography on silica gel using petroleum ether/ethyl acetate as the eluent, giving the titled product as a white solid (23 mg, 47% yield). m.p.: 62.5 - 65.0 °C.

^{1}H NMR (400 MHz, $CDCl_3$) δ 9.89 (s, 1H), 7.83 (d, J = 8.7 Hz, 2H), 7.15 (d, J = 8.7 Hz, 2H), 5.73 (t, J = 3.2 Hz, 1H), 4.57 - 4.50 (m, 1H), 4.20 - 4.16 (m, 1H), 3.86 (dd, J = 12.0, 2.4 Hz, 1H), 3.76 (dd, J = 12.0, 4.0 Hz, 1H), 2.28 (ddd, J = 12.8, 10.0, 3.2 Hz, 1H), 2.07 (dt, J = 13.2, 4.0 Hz, 1H), 1.12 - 0.97 (m, 28H) ppm. **^{13}C NMR** (101 MHz, $CDCl_3$) δ 191.03, 162.04, 131.99, 130.87, 116.52, 96.66, 70.29, 68.71, 64.93, 34.52, 17.77, 17.73, 17.58, 17.51, 17.43, 17.34, 17.30, 14.13, 13.75, 13.44, 12.94 ppm. **IR (thin film, cm^{-1})**: 2943, 2866, 2637, 2323, 2165, 2050, 1981, 1963, 1602, 1258, 1021, 796. **ESI-HRMS:** Calculated for $C_{24}H_{40}O_6Si_2Na$ $(M+Na)^+$: 503.2256, Found: 503.2246. **[α]$_D^{25}$** = -81.5 (c = 0.13, $CHCl_3$).

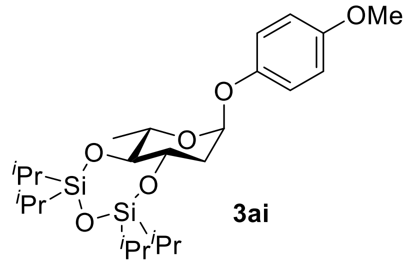

3ai

(5a*S*,6*S*,8*S*,9a*S*)-2,2,4,4-tetraisopropyl-8-(4-methoxyphenoxy)-6-methyltetrahydro-6*H*-pyrano[3,4-*f*][1,3,5,2,4]trioxadisilepine

3ai was synthesized according to the general procedure C and isolated by column chromatography on silica gel using petroleum ether/ethyl acetate as the eluent, giving the titled product as a colorless syrup (37 mg, 75% yield).

^{1}H NMR (400 MHz, $CDCl_3$) δ 7.03 - 6.98 (m, 2H), 6.85 - 6.79 (m, 2H), 5.43 (d, J = 3.6 Hz, 1H), 4.17 (ddd, J = 11.2, 8.0, 5.2 Hz, 1H), 3.83 - 3.74 (m, 4H), 3.32 (t, J = 8.8 Hz, 1H), 2.29 (dd, J = 13.6, 5.6 Hz, 1H), 1.85 (ddd, J = 13.6, 11.6, 4.0 Hz, 1H), 1.26 (d, J = 6.4 Hz, 3H), 1.14 - 1.00 (m, 28H) ppm. **^{13}C NMR** (101 MHz, $CDCl_3$) δ 154.83, 151.07, 118.08, 114.64, 97.03, 80.09, 71.42, 68.82, 55.78, 38.70, 18.19, 17.78, 17.58, 17.53, 17.50, 17.44, 17.41, 13.11, 13.06, 12.44 ppm. **IR (thin film, cm^{-1})**: 2926, 2866, 2323, 2161, 2049, 2032, 1979, 1506, 1463, 1383, 1258, 1227, 1120, 1053, 984, 883, 824, 700, 599. **ESI-HRMS:** Calculated for $C_{25}H_{44}O_6Si_2Na$ $(M+Na)^+$: 519.2569, Found: 519.2559. **[α]$_D^{25}$** = -78.3 (c = 0.18, $CHCl_3$).

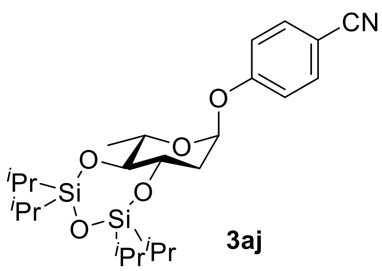

3aj

4-(((5a*S*,6*S*,8*S*,9a*S*)-2,2,4,4-tetraisopropyl-6-methyltetrahydro-6*H*-pyrano[3,4-*f*][1,3,5,2,4]trioxadisilepin-8-yl)oxy)benzonitrile

3aj was synthesized according to the general procedure C and isolated by column chromatography on silica gel using petroleum ether/ethyl acetate as the eluent, giving the titled product as a colorless syrup (26 mg, 53% yield).

^{1}H NMR (400 MHz, $CDCl_3$) δ 7.58 (d, J = 8.8 Hz, 2H), 7.14 (d, J = 8.8 Hz, 2H), 5.61 (d, J = 3.2 Hz, 1H), 4.14 (ddd, J = 11.6, 8.4, 5.2 Hz, 1H), 3.67 - 3.58 (m, 1H), 3.33 (t, J = 8.8 Hz, 1H), 2.30 (dd, J = 13.6, 5.2 Hz, 1H), 1.94 - 1.85 (m, 1H), 1.23 (d, J = 6.4 Hz, 3H), 1.14 - 0.98 (m, 28H) ppm. **^{13}C NMR** (101 MHz, $CDCl_3$) δ 160.19, 134.02, 119.25, 117.10, 105.14, 96.09, 79.68, 71.15, 69.50, 38.24, 18.14, 17.75, 17.54, 17.48, 17.44, 17.39, 13.06, 13.05, 12.38 ppm. **IR (thin film, cm^{-1})**: 2944, 2867, 2323, 2226, 2160, 2037, 1980, 1605, 1507, 1463, 1384, 1247, 1200, 1173, 1123, 1054, 980, 923, 884, 835, 701. **ESI-HRMS:** Calculated for $C_{25}H_{41}NO_5Si_2Na$ $(M+Na)^+$: 514.2415, Found: 514.2405. **$[\alpha]_D^{25}$** = -105.0 (c = 0.38, $CHCl_3$).

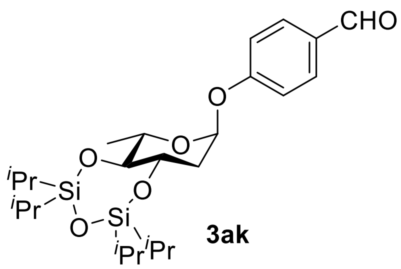

3ak

4-(((5a*S*,6*S*,8*S*,9a*S*)-2,2,4,4-tetraisopropyl-6-methyltetrahydro-6*H*-pyrano[3,4-*f*][1,3,5,2,4]trioxadisilepin-8-yl)oxy)benzaldehyde

3ak was synthesized according to the general procedure C and isolated by column chromatography on silica gel using petroleum ether/ethyl acetate as the eluent, giving the titled product as a colorless syrup (36 mg, 72% yield).

^{1}H NMR (400 MHz, $CDCl_3$) δ 9.90 (s, 1H), 7.83 (d, J = 8.4 Hz, 2H), 7.19 (d, J = 8.4 Hz, 2H), 5.66 (d, J = 3.2 Hz, 1H), 4.17 (ddd, J = 11.6, 8.4, 5.2 Hz, 1H), 3.70 - 3.61 (m, 1H), 3.34 (t, J = 8.8 Hz, 1H), 2.32 (dd, J = 13.6, 5.2 Hz, 1H), 1.95 - 1.86 (m, 1H), 1.24 (d, J = 6.4 Hz, 3H), 1.15 - 0.99 (m, 28H) ppm. **^{13}C NMR** (101 MHz, $CDCl_3$) δ 191.07, 161.91, 131.95, 130.83, 116.67, 95.99, 79.78, 71.21, 69.46, 38.32, 18.17, 17.76, 17.56, 17.49, 17.46, 17.41, 13.09, 13.07, 12.41 ppm. **IR (thin film, cm^{-1})**: 3377, 2943, 2866, 2728, 2323, 2166, 2049, 1979, 1700, 1601, 1579, 1506, 1462, 11385, 1306, 1244, 1199, 1161, 1120, 1094, 1053, 978, 922, 878, 816, 795, 757, 699. **ESI-HRMS:** Calculated for $C_{25}H_{42}O_6Si_2Na$ $(M+Na)^+$: 517.2412, Found: 517.2403. **$[\alpha]_D^{25}$** = -103.0 (c = 0.33, $CHCl_3$).

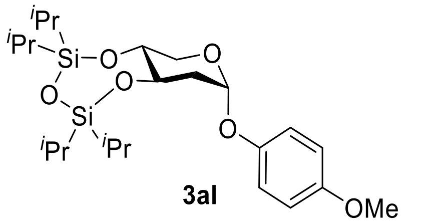

3al

(5a*R*,8*R*,9a*R*)-2,2,4,4-tetraisopropyl-8-(4-methoxyphenoxy)tetrahydro-6*H*-pyrano[3,4-*f*][1,3,5,2,4]trioxadisilepine

3al was synthesized according to the general procedure C and isolated by column chromatography on silica gel using petroleum ether/ethyl acetate as the eluent, giving the titled product as a colorless syrup (36 mg, 75% yield).

^{1}H NMR (400 MHz, $CDCl_3$) δ 7.02 - 6.97 (m, 2H), 6.85 - 6.80 (m, 2H), 5.46 (d, *J* = 3.6 Hz, 1H), 4.16 (ddd, *J* = 11.2, 8.0, 5.2 Hz, 1H), 3.77 (s, 3H), 3.75 - 3.65 (m, 2H), 3.59 (t, *J* = 10.8 Hz, 1H), 2.30 (dd, *J* = 13.6, 5.6 Hz, 1H), 1.82 - 1.73 (m, 1H), 1.14 - 0.99 (m, 28H) ppm. **^{13}C NMR** (101 MHz, $CDCl_3$) δ 154.94, 150.86, 118.14, 114.67, 97.19, 74.23, 71.77, 62.97, 55.79, 38.15, 17.77, 17.71, 17.49, 17.47, 17.45, 17.43, 17.40, 13.17, 12.47, 12.46 ppm. **IR (thin film, cm^{-1})**: 2943, 2866, 2323, 2162, 2050, 1979, 1507, 1464, 1387, 1225, 1192, 1114, 999, 884, 822, 701. **ESI-HRMS:** Calculated for $C_{24}H_{42}O_6Si_2Na$ $(M+Na)^+$: 505.2412, Found: 505.2404. **$[\alpha]_D^{25}$** = +67.6 (c = 0.25, $CHCl_3$).

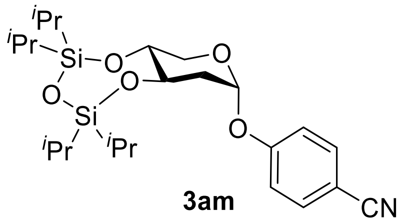

3am

4-(((5a*R*,8*R*,9a*R*)-2,2,4,4-tetraisopropyltetrahydro-6*H*-pyrano[3,4-*f*][1,3,5,2,4]trioxadisilepin-8-yl)oxy)benzonitrile

3am was synthesized according to the general procedure C and isolated by column chromatography on silica gel using petroleum ether/ethyl acetate as the eluent, giving the titled product as a colorless syrup (24 mg, 50% yield).

^{1}H NMR (400 MHz, $CDCl_3$) δ 7.59 (d, *J* = 8.8 Hz, 2H), 7.13 (d, *J* = 8.8 Hz, 2H), 5.63 (d, *J* = 3.2 Hz, 1H), 4.17 - 4.08 (m, 1H), 3.78 - 3.63 (m, 2H), 3.43 (t, *J* = 10.4 Hz, 1H), 2.31 (dd, *J* = 14.0, 5.6 Hz, 1H), 1.87 - 1.78 (m, 1H), 1.15 - 0.97 (m, 28H) ppm. **^{13}C NMR** (101 MHz, $CDCl_3$) δ 160.02, 134.03, 119.21, 117.16, 105.30, 96.27, 73.79, 71.51, 63.32, 37.71, 17.74, 17.69, 17.44, 17.41, 17.39, 17.35, 13.15, 13.13, 12.40 ppm. **IR (thin film, cm^{-1})**: 2944, 2866, 2323, 2226, 2161, 2049, 1979, 1605, 1507, 1463, 1382, 1239, 1113, 992, 883, 838, 702. **ESI-HRMS:** Calculated for $C_{24}H_{39}NO_5Si_2Na$ $(M+Na)^+$: 500.2259, Found: 500.2252. **$[\alpha]_D^{25}$** = +84.4 (c = 0.34, $CHCl_3$).

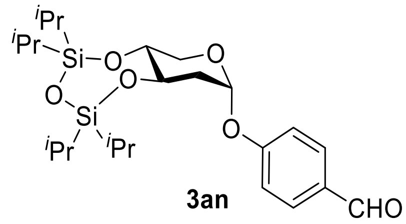

3an

4-(((5a*R*,8*R*,9a*R*)-2,2,4,4-tetraisopropyltetrahydro-6*H*-pyrano[3,4-*f*][1,3,5,2,4]trioxadisilepin-8-yl)oxy)benzaldehyde

3an was synthesized according to the general procedure C and isolated by column chromatography on silica gel using petroleum ether/ethyl acetate as the eluent, giving the titled product as a colorless syrup (26 mg, 54% yield).

^{1}H NMR (400 MHz, $CDCl_3$) δ 9.90 (s, 1H), 7.87 - 7.80 (m, 2H), 7.20 - 7.16 (m, 2H), 5.68 (d, *J* = 2.4 Hz, 1H), 4.16 (ddd, *J* = 11.2, 8.0, 5.2 Hz, 1H), 3.78 - 3.66 (m, 2H), 3.47 (t, *J* = 10.4 Hz, 1H), 2.33 (ddd, *J* = 14.0, 5.6, 1.6 Hz, 1H), 1.84 (ddd, *J* = 14.4, 11.2, 3.6 Hz, 1H), 1.14 - 0.97 (m, 28H) ppm. **^{13}C NMR** (101 MHz, $CDCl_3$) δ 191.05, 161.72, 131.94, 130.93, 116.72, 96.16, 73.88, 71.57, 63.33, 37.78, 17.75, 17.69, 17.45, 17.42, 17.40, 17.36, 13.16, 13.14, 12.43 ppm. **IR (thin film, cm^{-1})**: 2943, 2866, 2162, 2049, 1979, 1698, 1601, 1506, 1436, 1385, 1225, 1200, 1162, 1109, 988, 881, 831, 798, 699. **ESI-HRMS:** Calculated for $C_{24}H_{40}O_6Si_2Na$ $(M+Na)^+$: 503.2256, Found: 503.2244. **[α]$_D^{25}$** = +95.3 (c = 0.39, $CHCl_3$).

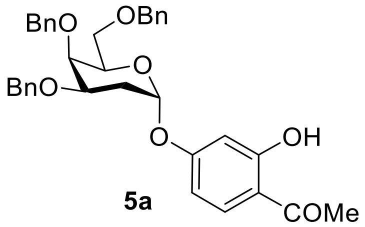

5a

1-(4-(((2*R*,4*R*,5*R*,6*R*)-4,5-bis(benzyloxy)-6-((benzyloxy)methyl)tetrahydro-2*H*-pyran-2-yl)oxy)-2-hydroxyphenyl)ethan-1-one

5a was synthesized according to the general procedure C and isolated by column chromatography on silica gel using petroleum ether/ethyl acetate as the eluent, giving the titled product as a colorless syrup, (250 mg, 72% yield).

^{1}H NMR (400 MHz, $CDCl_3$) δ 12.59 (s, 1H), 7.61 (d, *J* = 8.9 Hz, 1H), 7.42 - 7.18 (m, 15H), 6.63 (d, *J* = 2.4 Hz, 1H), 6.56 (dd, *J* = 8.9, 2.3 Hz, 1H), 5.78 (d, *J* = 3.4 Hz, 1H), 4.97 (d, *J* = 11.5 Hz, 1H), 4.68 (s, 2H), 4.64 (d, *J* = 11.5 Hz, 1H), 4.39 (q, *J* = 12.0 Hz, 2H), 4.14 - 4.06 (m, 1H), 4.02 (s, 1H), 3.96 (t, *J* = 6.6 Hz, 1H), 3.68 - 3.61 (m, 1H), 3.53 (dd, *J* = 9.4, 5.7 Hz, 1H), 2.55 (s, 3H), 2.42 (td, *J* = 12.5, 3.7 Hz, 1H), 2.20 (dd, *J* = 13.0, 4.6 Hz, 1H) ppm. **^{13}C NMR** (101 MHz, $CDCl_3$) δ 202.89, 164.89, 163.29, 138.79, 138.41, 138.05, 132.48, 128.61, 128.46, 128.42, 128.36, 127.86, 127.82, 127.80, 127.76, 127.50, 114.92, 108.36, 104.39, 96.40, 74.63, 74.36, 73.51, 72.74, 71.30, 70.73, 69.12, 30.97, 26.42 ppm. **IR (thin film, cm^{-1})**: 3190, 2287, 2211, 2160, 2107, 2038, 1979, 1568, 1361, 1256, 1117, 1057, 1049, 858, 798, 755, 689, 643, 522, 496, 425. **ESI-HRMS:** Calculated for $C_{35}H_{36}O_7Na$ $(M+Na)^+$: 591.2353, Found: 591.2360. **[α]$_D^{25}$** = +114.8 (c = 0.41, $CHCl_3$).

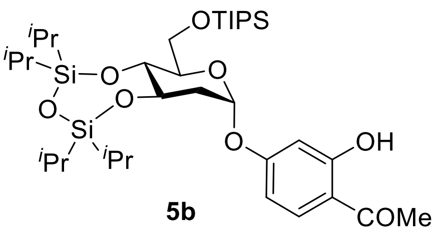

5b

1-(2-hydroxy-4-(((5a*R*,6*R*,8*R*)-2,2,4,4-tetraisopropyl-6-(((triisopropylsilyl)oxy)methyl)tetrahydro-6*H*-pyrano[3,4-*f*][1,3,5,2,4]trioxadisilepin-8-yl)oxy)phenyl)ethan-1-one

5b was synthesized according to the general procedure C and isolated by column chromatography on silica gel using petroleum ether/ethyl acetate as the eluent, giving the titled product as a white solid (332 mg, 95% yield).

^{1}H NMR (400 MHz, $CDCl_3$) δ 12.52 (s, 1H), 7.61 (d, *J* = 8.8 Hz, 1H), 6.67 - 6.58 (m, 2H), 5.68 (d, *J* = 3.3 Hz, 1H), 4.19 (ddd, *J* = 11.5, 8.0, 5.3 Hz, 1H), 3.96 (dd, *J* = 11.0, 1.9 Hz, 1H), 3.82 (dd, *J* = 10.9, 5.2 Hz, 1H), 3.61 (t, *J* = 8.0 Hz, 1H), 3.59 - 3.54 (m, 1H), 2.55 (s, 3H), 2.29 (dd, *J* = 13.6, 5.3 Hz, 1H), 1.91 - 1.81 (m, 1H), 1.18 - 0.95 (m, 49H) ppm. **^{13}C NMR** (101 MHz, $CDCl_3$) δ 202.82, 164.94, 163.26, 132.36, 114.90, 108.47, 104.62, 95.78, 74.60, 74.17, 71.48, 62.93, 37.96, 26.42, 18.04, 18.01, 17.76, 17.58, 17.57, 17.52, 17.46, 17.43, 17.40, 13.16, 13.06, 12.50, 12.41, 12.08 ppm. **IR (thin film, cm^{-1})**: 3019, 2050, 2001, 1538, 1370, 1214, 1000, 746, 669, 579, 473. **ESI-HRMS:** Calculated for $C_{35}H_{64}O_8Si_3Na$ $(M+Na)^+$: 719.3801, Found: 719.3809. **$[\alpha]_D^{25}$** = +105.0 (c = 0.28, $CHCl_3$).

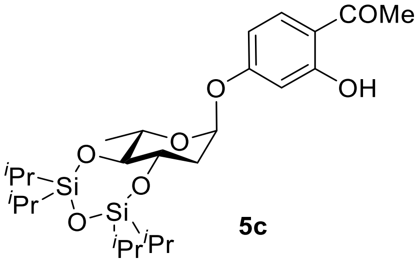

5c

1-(2-hydroxy-4-(((5a*S*,6*S*,8*S*,9a*S*)-2,2,4,4-tetraisopropyl-6-methyltetrahydro-6*H*-pyrano[3,4-*f*][1,3,5,2,4]trioxadisilepin-8-yl)oxy)phenyl)ethan-1-one

5c was synthesized according to the general procedure C and isolated by column chromatography on silica gel using petroleum ether/ethyl acetate as the eluent, giving the titled product as a colorless syrup (189 mg, 66% yield).

^{1}H NMR (400 MHz, $CDCl_3$) δ 12.60 (s, 1H), 7.64 (d, *J* = 8.9 Hz, 1H), 6.66 (d, *J* = 2.4 Hz, 1H), 6.58 (dd, *J* = 8.8, 2.4 Hz, 1H), 5.62 (d, *J* = 3.4 Hz, 1H), 4.14 (ddd, *J* = 11.3, 8.3, 5.3 Hz, 1H), 3.69 - 3.59 (m, 1H), 3.32 (t, *J* = 8.8 Hz, 1H), 2.57 (s, 3H), 2.29 (dd, *J* = 13.7, 5.3 Hz, 1H), 1.88 (ddd, *J* = 13.6, 11.3, 3.6 Hz, 1H), 1.24 (d, *J* = 6.2 Hz, 3H), 1.14 - 0.98 (m, 28H) ppm. **^{13}C NMR** (101 MHz, $CDCl_3$) δ 202.90, 164.98, 163.33, 132.40, 114.88, 108.53, 104.29, 95.88, 79.78, 71.21, 69.48, 38.25, 26.44, 18.17, 17.76, 17.56, 17.49, 17.47, 17.41, 13.07, 12.43 ppm. **IR (thin film, cm^{-1})**: 3160, 2945, 2867, 2323, 2188, 2050, 1980, 1635, 1574, 1368, 1251, 1119, 1052, 989, 858, 636, 434, 428. **ESI-HRMS:** Calculated for $C_{26}H_{44}O_7Si_2Na$ $(M+Na)^+$: 547.2518, Found: 547.2527. **$[\alpha]_D^{25}$** = -117.7 (c = 0.17, $CHCl_3$).

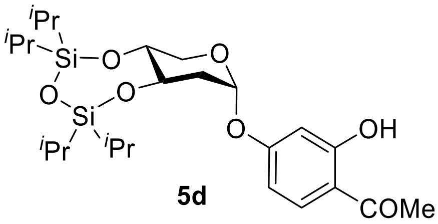

5d

1-(2-hydroxy-4-(((5a*R*,8*R*)-2,2,4,4-tetraisopropyltetrahydro-6*H*-pyrano[3,4-*f*][1,3,5,2,4]trioxadisilepin-8-yl)oxy)phenyl)ethan-1-one

5d was synthesized according to the general procedure C and isolated by column chromatography on silica gel using petroleum ether/ethyl acetate as the eluent, giving the titled product as a colorless syrup (148 mg, 55% yield).

^{1}H NMR (400 MHz, $CDCl_3$) δ 12.59 (s, 1H), 7.64 (d, *J* = 8.9 Hz, 1H), 6.61 (d, *J* = 2.4 Hz, 1H), 6.55 (dd, *J* = 8.9, 2.4 Hz, 1H), 5.68 (s, 1H), 4.54 - 4.76 (m, 1H), 4.17 (s, 1H), 3.84 (dd, *J* = 12.0, 2.2 Hz, 1H), 3.75 (dd, *J* = 11.9, 4.3 Hz, 1H), 2.56 (s, 3H), 2.29 - 2.20 (m, 1H), 2.04 (dt, *J* = 13.1, 3.8 Hz, 1H), 1.13 - 1.00 (m, 28H) ppm. **^{13}C NMR** (101 MHz, $CDCl_3$) δ 202.90, 164.97, 163.44, 132.49, 114.93, 108.30, 104.23, 96.51, 70.33, 68.73, 64.93, 34.54, 26.44, 17.78, 17.73, 17.59, 17.51, 17.43, 17.30, 14.09, 13.73, 13.47, 12.97 ppm. **IR (thin film, cm^{-1})**: 3019, 2050, 1214, 745, 670, 483, 463. **ESI-HRMS:** Calculated for $C_{25}H_{42}O_7Si_2Na$ $(M+Na)^+$: 533.2361, Found: 533.2369. **$[\alpha]_D^{25}$** = -81.8 (c = 0.22, $CHCl_3$).

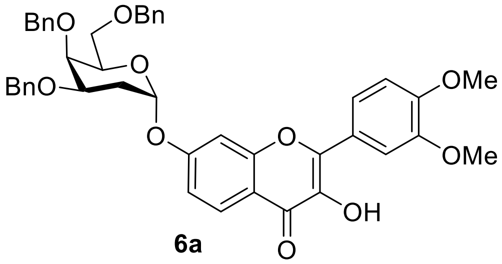

6a

7-(((2*R*,4*R*,5*R*,6*R*)-4,5-bis(benzyloxy)-6-((benzyloxy)methyl)tetrahydro-2*H*-pyran-2-yl)oxy)-2-(3,4-dimethoxyphenyl)-3-hydroxy-4*H*-chromen-4-one

6a was synthesized according to the general procedure D and isolated by column chromatography on silica gel using petroleum ether/ethyl acetate as the eluent, giving the titled product as a brown syrup (23 mg, 48% yield).

^{1}H NMR (400 MHz, $CDCl_3$) δ 8.12 (d, *J* = 8.9 Hz, 1H), 7.84 (dd, *J* = 8.6, 2.0 Hz, 1H), 7.78 (s, 1H), 7.43 - 7.12 (m, 17H), 7.05 (dd, *J* = 8.9, 2.1 Hz, 1H), 7.00 (d, *J* = 8.6 Hz, 1H), 5.86 (d, *J* = 3.4 Hz, 1H), 4.99 (d, *J* = 11.4 Hz, 1H), 4.70 (s, 2H), 4.64 (d, *J* = 11.4 Hz, 1H), 4.44 - 4.32 (m, 2H), 4.17 - 4.09 (m, 1H), 4.06 - 3.99 (m, 2H), 3.96 (s, 6H), 3.64 - 3.60 (m, 1H), 3.56 (dd, *J* = 9.4, 5.9 Hz, 1H), 2.48 (td, *J* = 12.6, 3.5 Hz, 1H), 2.26 (dd, *J* = 13.1, 4.6 Hz, 1H) ppm. **^{13}C NMR** (101 MHz, $CDCl_3$) δ 172.72, 161.22, 156.94, 150.68, 148.97, 144.80, 138.77, 138.37, 137.89, 137.56, 128.66, 128.46, 128.32, 127.88, 127.82, 127.68, 127.52, 126.76, 123.95, 121.40, 115.78, 115.53, 111.07, 110.57, 103.41, 96.95, 74.65, 74.36, 73.50, 72.77, 71.44, 70.79, 69.18, 56.12, 31.07 ppm. **IR (thin film, cm^{-1})**: = 2923, 2853, 2323, 2285, 2161, 2050, 2038, 1979, 1612, 1514, 1453, 1400, 1258, 1180, 1093, 1020, 795, 697, 521. **ESI-HRMS:** Calculated for $C_{44}H_{43}O_{10}$ $(M+H)^+$: 731.2851, Found: 731.2871. **$[\alpha]_D^{25}$** = +105.3 (c = 0.15, $CHCl_3$).

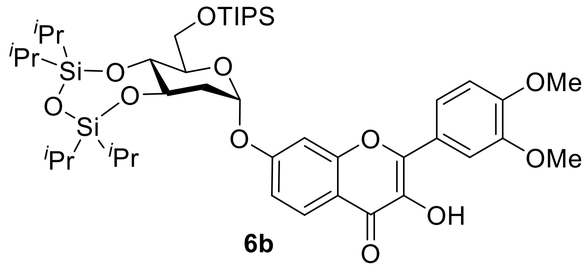

6b

2-(3,4-dimethoxyphenyl)-3-hydroxy-7-(((5a*R*,6*R*,8*R*)-2,2,4,4-tetraisopropyl-6-(((triisopropylsilyl)oxy)methyl)tetrahydro-6*H*-pyrano[3,4-*f*][1,3,5,2,4]trioxadisilepin-8-yl)oxy)-4*H*-chromen-4-one

6b was synthesized according to the general procedure D and isolated by column chromatography on silica gel using petroleum ether/ethyl acetate as the eluent, giving the titled product as a brown syrup (23 mg, 66% yield).

^{1}H NMR (500 MHz, $CDCl_3$) δ 8.12 (d, *J* = 8.8 Hz, 1H), 7.89 - 7.81 (m, 1H), 7.85 - 7.82 (m, 1H), 7.32 (s, 1H), 7.11 (d, *J* = 8.8, Hz, 1H), 7.00 (d, *J* = 8.6 Hz, 1H), 5.78 (s, 1H), 4.27 - 4.18 (m, 1H), 4.04 - 3.89 (m, 8H), 3.82 (dd, *J* = 11.0, 5.3 Hz, 1H), 3.66 - 3.59 (m, 2H), 2.36 (dd, *J* = 13.0, 5.5 Hz, 1H), 1.97 - 1.88 (m, 1H), 1.16 - 0.91 (m, 49H). ppm. **^{13}C NMR** (101 MHz, $CDCl_3$) δ 172.77, 161.12, 156.98, 150.68, 148.98, 144.76, 137.53, 126.61, 124.02, 121.38, 116.05, 115.44, 111.00, 110.69, 103.40, 96.19, 74.71, 74.37, 71.44, 63.13, 56.20, 56.12, 38.00, 17.98, 17.96, 17.78, 17.56, 17.54, 17.51, 17.46, 17.42, 17.38, 13.13, 13.07, 12.47, 12.37, 11.99 ppm. **IR (thin film, cm^{-1})**: = 2941, 2865, 2323, 2161, 2050, 1978, 1616, 1514, 1457, 1401, 1258, 1074, 1012, 883, 794, 701, 478. **ESI-HRMS:** Calculated for $C_{44}H_{71}O_{11}Si_3$ $(M+H)^+$: 859.4299, Found: 859.4294. **$[\alpha]_D^{25}$** = +98.0 (c = 0.25, $CHCl_3$).

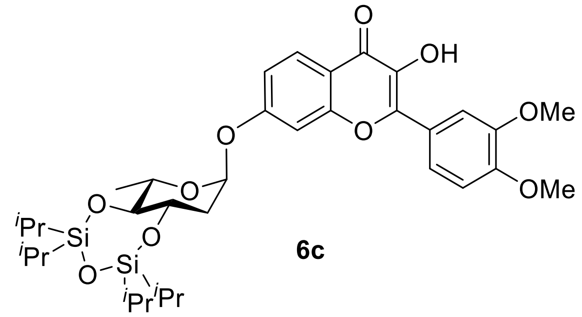

6c

2-(3,4-dimethoxyphenyl)-3-hydroxy-7-(((5a*S*,6*S*,8*S*,9a*S*)-2,2,4,4-tetraisopropyl-6-methyltetrahydro-6*H*-pyrano[3,4-*f*][1,3,5,2,4]trioxadisilepin-8-yl)oxy)-4*H*-chromen-4-one

6c was synthesized according to the general procedure D and isolated by column chromatography on silica gel using petroleum ether/ethyl acetate as the eluent, giving the titled product as a brown syrup (23 mg, 35% yield).

^{1}H NMR (400 MHz, $CDCl_3$) δ 8.15 (d, *J* = 8.9 Hz, 1H), 7.91 - 7.81 (m, 2H), 7.26 (s, 1H), 7.11 (d, *J* = 8.9 Hz, 1H), 7.02 (d, *J* = 8.5 Hz, 1H), 5.72 (s, 1H), 4.23 - 4.13 (m, 1H), 4.00 (s, 3H), 3.97 (s, 3H), 3.75 - 3.65 (m, 1H), 3.52 - 3.42 (m, 1H), 3.36 (t, *J* = 8.7 Hz, 1H), 2.36 (dd, *J* = 13.7, 5.3 Hz, 1H), 1.98 - 1.89 (m, 1H), 1.29 - 1.25 (m, 3H), 1.19 - 0.78 (m, 28H) ppm. **^{13}C NMR** (101 MHz, $CDCl_3$) δ 172.77, 161.20, 157.01, 150.73, 149.01, 144.83, 137.57, 126.81, 124.01, 121.35, 115.75, 115.52, 111.08, 110.79, 103.31, 96.32, 79.77, 71.18, 69.67, 56.24, 56.13, 38.32, 18.23, 17.79, 17.57, 17.50, 17.47, 17.41, 13.10, 13.08, 12.42 ppm. **IR (thin film, cm^{-1})**: = 2927, 2867, 2322, 2285, 2078, 2050, 1980, 1616, 1515, 1457, 1401,

1265, 1121, 981, 886, 701. **ESI-HRMS:** Calculated for $C_{35}H_{51}O_{10}Si_2$ $(M+H)^+$: 687.3015, Found: 687.3024. **[α]$_D^{25}$** = -127.6 (c = 0.30, $CHCl_3$).

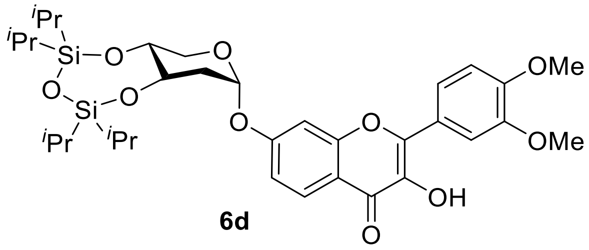

6d

2-(3,4-dimethoxyphenyl)-3-hydroxy-7-(((5a*R*,8*R*)-2,2,4,4-tetraisopropyltetrahydro-6*H*-pyrano[3,4-*f*][1,3,5,2,4]trioxadisilepin-8-yl)oxy)-4*H*-chromen-4-one

6d was synthesized according to the general procedure D and isolated by column chromatography on silica gel using petroleum ether/ethyl acetate as the eluent, giving the titled product as a brown syrup (23 mg, 30% yield).

^{1}H NMR (400 MHz, $CDCl_3$) δ 8.14 (d, *J* = 8.9 Hz, 1H), 7.87 (dd, *J* = 8.6, 2.1 Hz, 1H), 7.81 (d, *J* = 2.1 Hz, 1H), 7.27 (s, 1H), 7.06 (dd, *J* = 8.9, 2.3 Hz, 1H), 7.01 (d, *J* = 8.6 Hz, 1H), 5.81 - 5.76 (m, 1H), 4.58 - 4.52 (m, 1H), 4.22 (s, 1H), 4.00 (s, 3H), 3.97 (s, 3H), 3.91 (dd, *J* = 12.1, 1.8 Hz, 1H), 3.80 (dd, *J* = 12.1, 3.6 Hz, 1H), 2.36 - 2.27 (m, 1H), 2.11 (dt, *J* = 13.2, 3.6 Hz, 1H), 1.14 - 0.96 (m, 28H) ppm. **^{13}C NMR** (101 MHz, $CDCl_3$) δ 172.75, 161.36, 157.00, 150.71, 149.03, 144.79, 137.59, 126.80, 123.99, 121.42, 115.75, 115.50, 111.09, 110.52, 103.22, 97.19, 70.18, 68.64, 65.06, 56.20, 56.13, 34.26, 17.78, 17.75, 17.58, 17.52, 17.44, 17.35, 17.31, 14.23, 13.81, 13.38, 12.91 ppm. **IR (thin film, cm^{-1})**: = 2943, 2866, 2323, 2049, 1615, 1515, 1456, 1401, 1335, 1259, 1167, 1122, 1019, 884, 855, 800, 757, 733, 696, 594. **ESI-HRMS:** Calculated for $C_{34}H_{49}O_{10}Si_2$ $(M+H)^+$: 673.2859, Found: 673.2870. **[α]$_D^{25}$** = -92.5 (c = 0.43, $CHCl_3$).

Structure determination

1. The structure of compound **3a** was determined according to the analysis of ^{3}J value and NOE. Based on the analysis of ^{1}H and COSY spectra, the protons at C1, C2, C3, C4 can be assigned. Two protons at 1.88 ppm and 2.32 ppm are corresponding to the axial proton (H^{2a}) and equatorial proton (H^{2e}) respectively. The J value of these two protons is calculated as follows, based on the calculated data, the H^{1} should be located in the equatorial orientation.

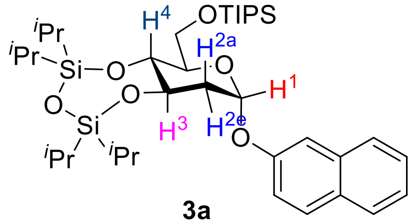

H^{2a}: 1.88 ppm J = 13.5, 11.4, 3.6 Hz

H^{2e}: 2.32 ppm J = 13.6, 5.3, 1.3 Hz

In addition, 1D NOE spectrum with the irradiation at H^{1}, a correlation between H^{1} and H^{4} is observed. Therefore, the configuration of the compound **3a** is assigned as α.

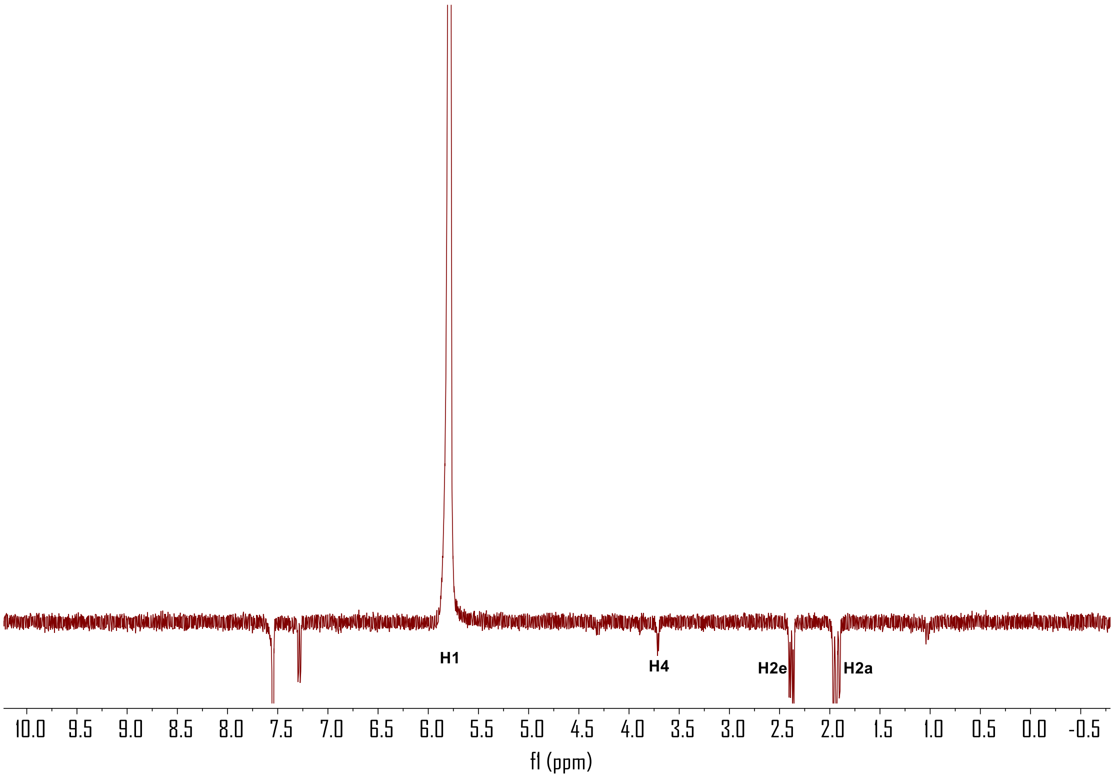

2. The structure of compound **3ai** was determined according to the analysis of ^3J value and NOE. Based on the analysis of ^{1}H and COSY spectra, the protons at C1, C2, C3, C4 can be assigned. Two protons at 1.85 ppm and 2.29 ppm are corresponding to the axial proton (H^{2a}) and equatorial proton (H^{2e}) respectively. The J value of these two protons is calculated as follows, based on the calculated data, the H^1 should be located in the equatorial orientation.

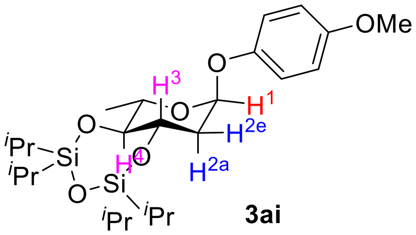

3ai

H^{2a}: 1.85 ppm J = 13.6, 11.6, 4.0 Hz

H^{2e}: 2.29 ppm J = 13.6, 5.6, 1.3 Hz

In addition, 1D NOE spectrum with the irradiation at H^1, correlations between H^1 and CH_3, H^1 and H^4 are observed. Therefore, the configuration of the compound **3ai** is assigned as α.

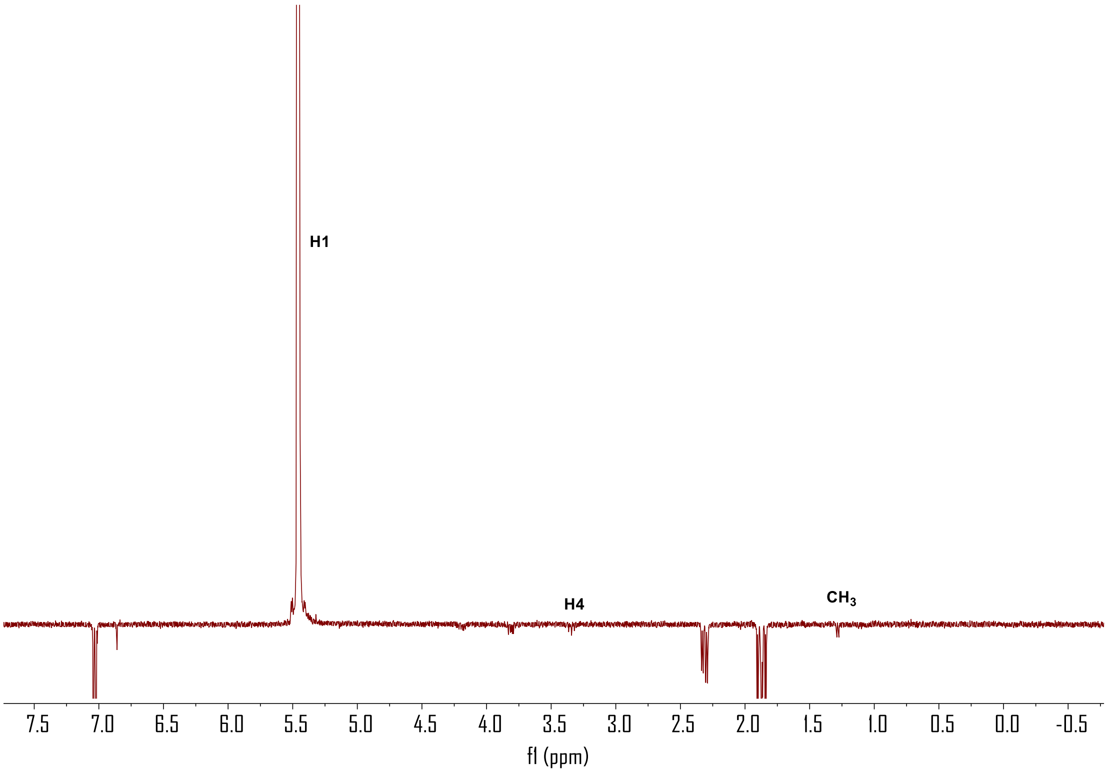

Table S3. Benchmarking studies with challenging aglycone 2,4-dihydroacetophenone between this method and other conventional catalysis

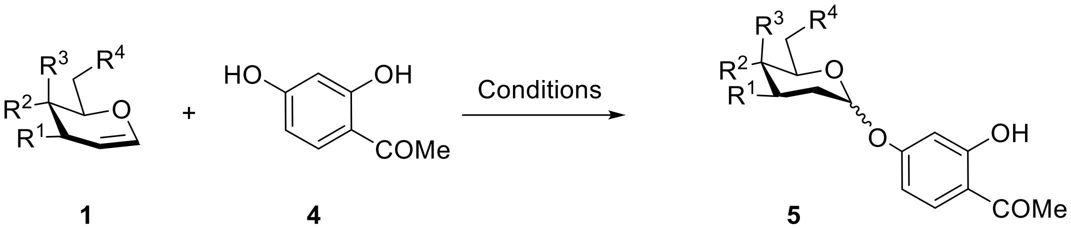

Entry	Catalyst	R[1]	R[2]	R[3]	R[4]	Yield(%)[e]	α : β[f]
1[a]	**Cat. A**	OBn	H	OBn	OBn	72	>20:1
	(Py·HBr)	$O^iPr_2SiOSi^iPr_2O$		H	OTIPS	95	>20:1
2[b]	TMSI/PPh_3[5]	OBn	H	OBn	OBn	74	1:0.4
		$O^iPr_2SiOSi^iPr_2O$		H	OTIPS	71	16:1
3[c]	$CuBr_2$[6]	OBn	H	OBn	OBn	73	1:1.6
		$O^iPr_2SiOSi^iPr_2O$		H	OTIPS	44	12:1
4[d]	$Bi(OTf)_3$[7]	OAc	H	OAc	OAc	NP[g]	ND[h]
		$O^iPr_2SiOSi^iPr_2O$		H	OTIPS	Messy	ND

[a]**1** (0.1 mmol), **4** (0.15 mmol), 20 mol% cat. **A** in toluene (2 mL), 12 h, nitrogen, 70 °C; [b]**1** (0.1 mmol), **4** (0.2 mmol), 20 mol% TMSI/PPh_3 in DCM (2 mL), 12 h, nitrogen, 40 °C; [c]**1** (0.1 mmol), **4** (0.2 mmol), 5 mol% $CuBr_2$ in CH_2Cl_2 (2 mL), 12 h, nitrogen, room temperature; [d]**1** (0.1 mmol), **4** (0.2 mmol), 5 mol% $Bi(OTf)_3$ in 1,4-dioxane (2 mL), 12 h, nitrogen, 50 °C; [e]Isolated yields. [f]α/β ratio was determined by ^1H NMR analysis. [g]NP = No product; [h]ND = Not determined.

Reaction with flavonoid substrate and alcohols

1. Flavonoid substrates were tested under standard conditions, however, the reactions did not proceed well. In most cases, due to the solubility of flavonoid substrates, only trace products were observed. A co-solvent THF or CH_3CN was added to improve the solubility, the result was also not promoted.

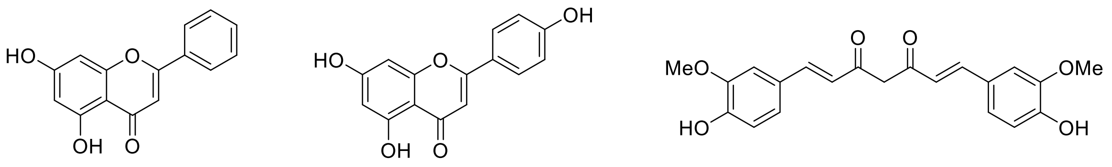

Chrysin Apigenin Curcumin

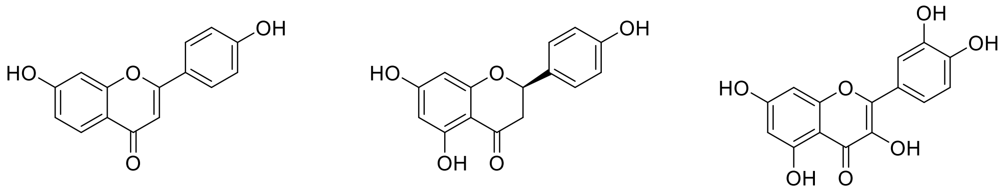

Daidzein Naringenin Quercetin

2. The method was compatible for alcohols, albeit giving lower yields ans lower selectivity. Isopropanol and diacetone galactal were tested and the details were listed as follows. The results were determined according to the known literature (C. Xu, V. U. B. Rao, J. Weigen, C. C. J. Loh, *Nat Commun* **2020**, *11*, 4911)

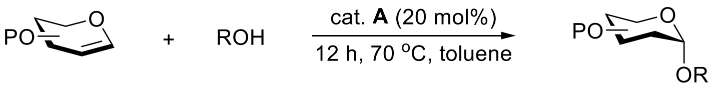

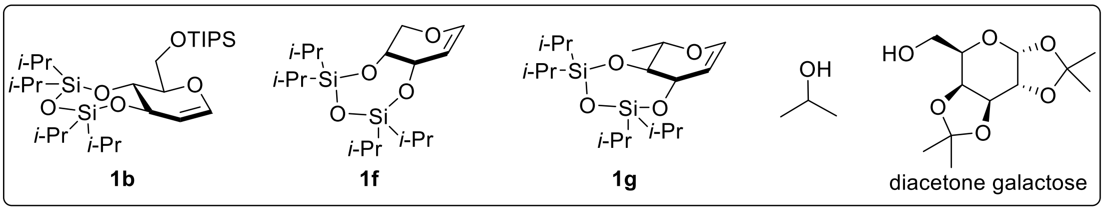

Donor	Acceptor	Isolated yield	α /β
**1b**	isopropanol	10%	7.4:1
**1b**	Diacetone galactose	63%	6.6:1
**1f**	Diacetone galactose	60%	>20:1
**1g**	Diacetone galactose	71%	16:1

Gram-scale reaction

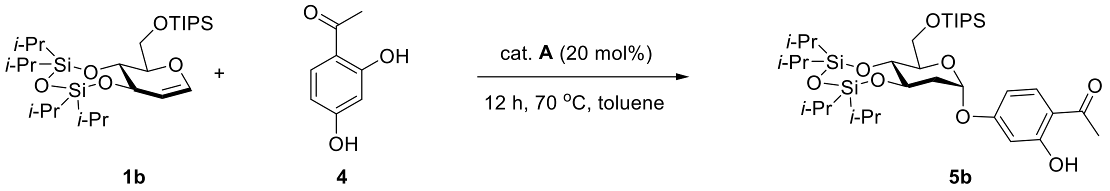

In a glove box filled with nitrogen, to an oven-dried 50 mL tube round-bottom flask with a stirring bar were added sugar **1b** (2 mmol, 1.0900 g, 1.0 equiv.), phenol **4** (3 mmol, 456.5 mg 1.5 equiv.), cat. **A** (0.4 mmol, 64.4 mg, 0.2 eq), toluene (20 mL). The mixture was stirred at 70°C for 12 h. Upon completion, solvent was removed with rotary evaporator and the residue was purified by silica gel chromatography (EtOAc/petroleum ether = 1/50) to afford the product **5b** (1.2711 g, 91%).

Mechanistic studies

Investigation of the interaction between catalyst and glycal

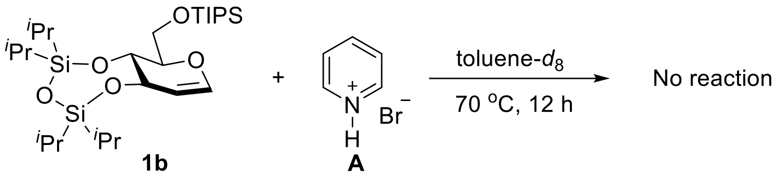

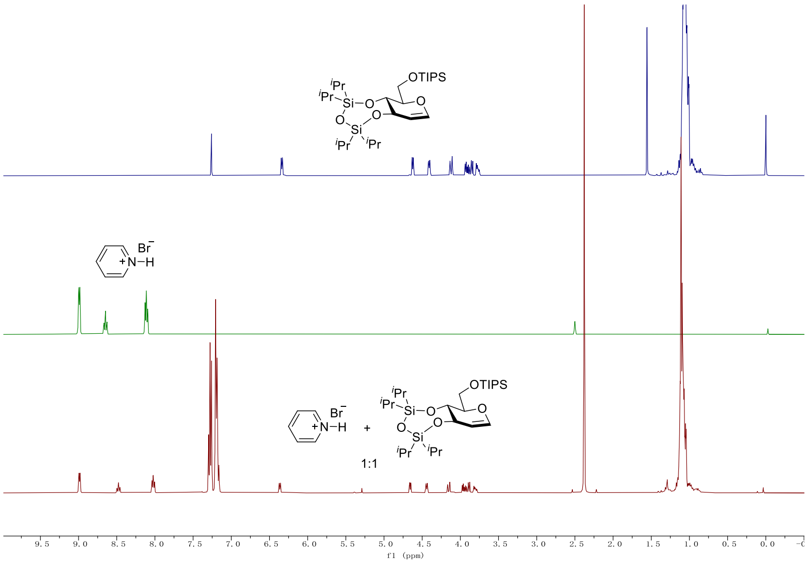

Figure S1. Stacked ^{1}H NMR spectra for the studies of interaction between catalyst and glycal

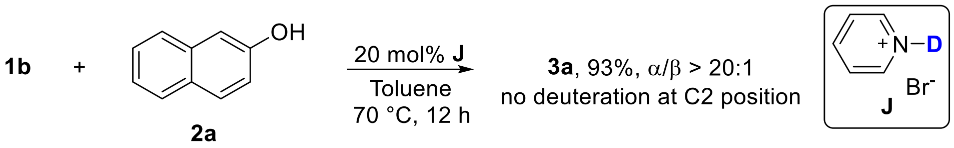

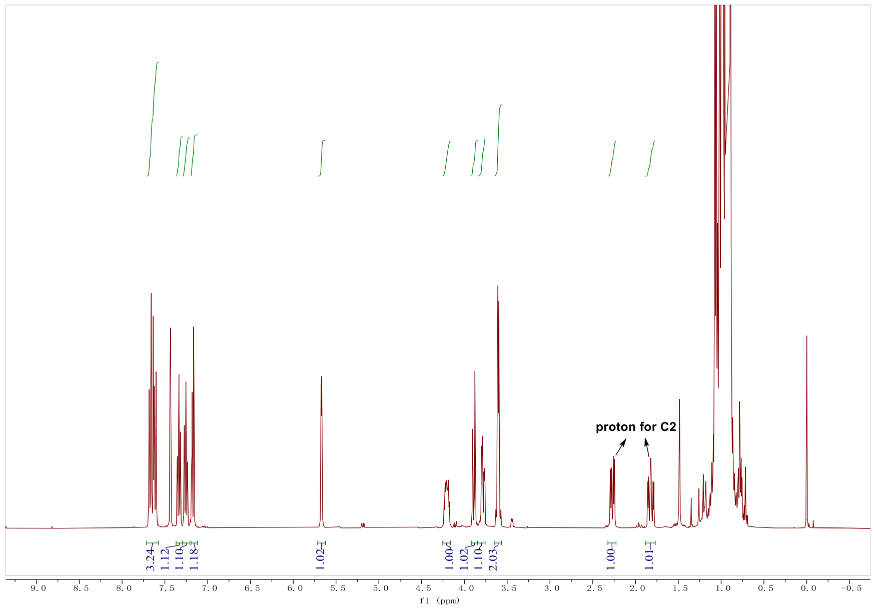

Figure S2. ^{1}H NMR spectrum of the isolated product **3a** from the titled reaction

The ^{1}H-NMR titration for studying the equilibrium of the reaction between 2c and catalyst A.

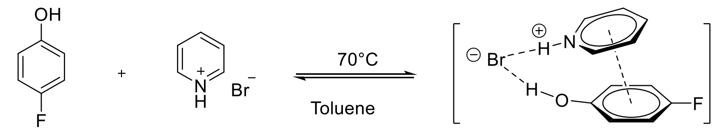

2c **A** **A···2c (Int.1c)**

In the glovebox, **2c** (2.80 mg, 0.025 mmol, 1.0 eq) was added into a J-Young NMR tube, then 0.5 mL of toluene-d_8 was added. After each ^{1}H-NMR measurement, 0.80 mg (0.005 mmol) of catalyst **A** was added and the NMR spectrum was remeasured.

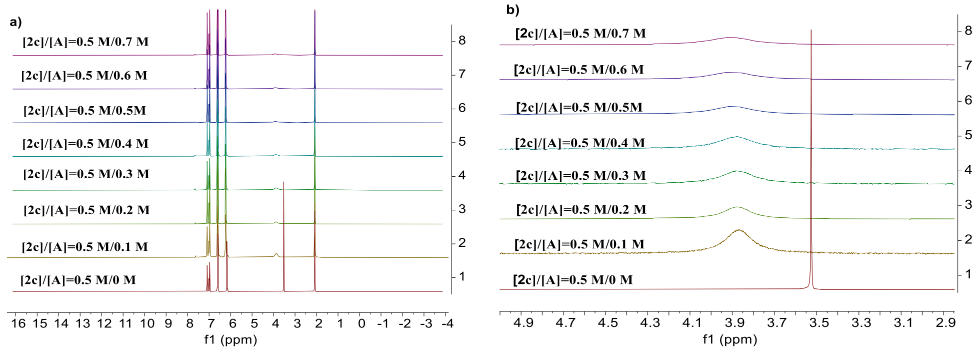

Figure S3. The full spectra of the NMR titration of the reaction between **2c** and **A**; (b) the zoomed-in spectra from 5.0 ppm-2.8 ppm.

Table S4. Chemical shift and peak width of OH in **2c** in each NMR titration measurement

Entry	Ratio	Chemical shift (ppm)	Peak width (Hz)
**1**	1/0	3.526	1.12
**2**	1/0.2	3.869	60.9
**3**	1/0.4	3.876	64.92
**4**	1/0.6	3.874	80.43
**5**	1/0.8	3.884	80.09
**6**	1/1	3.879	91.46
**7**	1/1.2	3.901	97.57
**8**	1/1.4	3.911	103.81

The UV-Vis titration of 2c and A mixture in toluene

Two groups of experiments were set up: the first group included three UV-Vis measurements for **2c** solution at different concentration (0.025 M, 0.05 M, 0.1 M) in toluene; the second group included three UV-Vis measurements for the mixture of **2c** and **A** (0.075 M, constant) at different concentration in toluene.

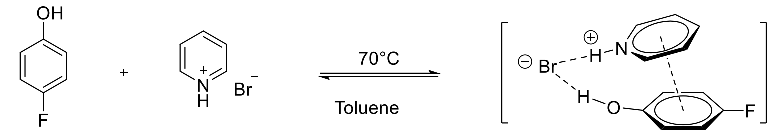

2c **A** **A···2c (Int.1c)**

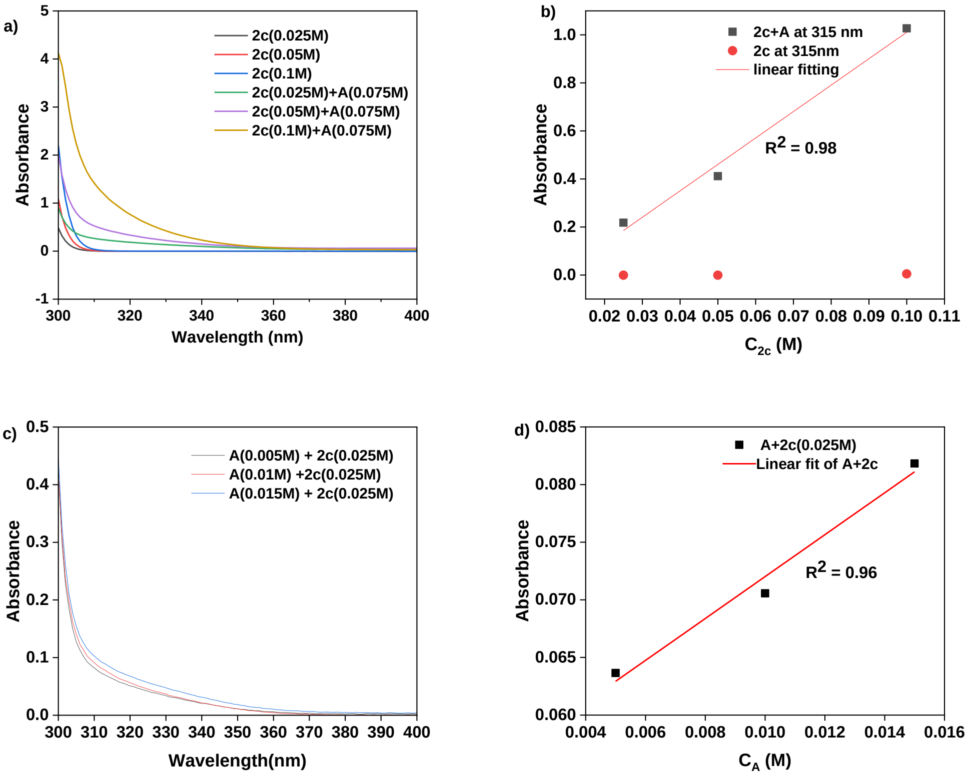

Figure S4. A) Concentration-dependent UV-Vis spectra of **2c** (black 0.025 M, red 0.05 M, blue 0.1 M) and the **A···2c (Int.1c)** generated from the reaction between **2c** (green 0.025 M, purple 0.05 M, khaki 0.1 M) with **A** (0.075 M) in toluene; b) Concentration-dependent absorbance at 315 nm of **2c** and **A···2c (Int.1c)** generated from the reaction between **2c** and **A** in toluene; c) Concentration-dependent UV-Vis spectra of the **A···2c (Int.1c)** generated from the reaction between **2c** (0.025 M) and **A** (black 0.005 M, red 0.01 M, blue 0.015 M) in toluene; d) Concentration-dependent absorbance at 315 nm of **A···2c (Int.1c)** generated from the reaction between **2c** (0.025 M) and **A** (0.005 M, 0.01 M, 0.015 M) in toluene and linear fit.

Fluorescence titration of 2c and A mixture in toluene

Two groups of experiments were set up for fluorescence titration: the first group included three samples of **2c** in toluene at different concentration (0.025 M, 0.05 M and 0.1 M); the second group included three samples of **2c** and **A** mixture in toluene at three different concentrations of **2c** (0.025 M, 0.05 M and 0.1 M) but the same concentration of **A** (0.01 M).

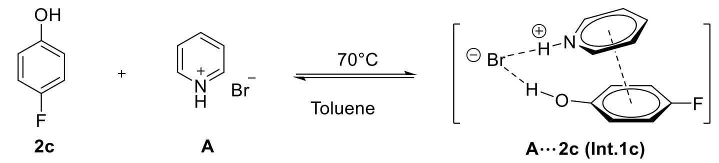

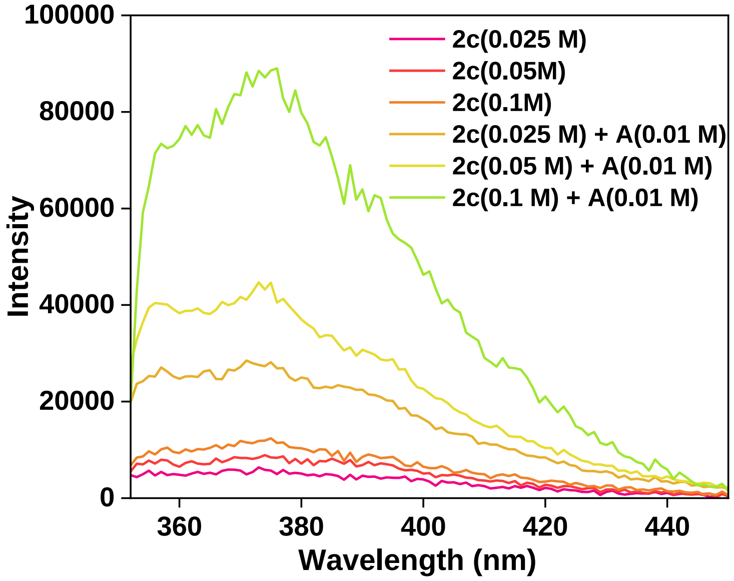

Figure S5. Concentration dependent fluorescence spectra of **2c** in toluene and concentration dependent fluorescence spectra of the **A**···**2c** (**Int.1c**) generated from the reaction between **2c** (0.025 M, 0.05 M,0.1 M) and **A** (0.01 M) in toluene.

Determination of the order of the reaction

$$\text{step 1:} \quad \beta B + \gamma C \underset{k_{-1}}{\overset{k_1}{\rightleftharpoons}} \delta BC$$

$$\text{step 2:} \quad \delta BC + \alpha A \xrightarrow{k_2} AB + C$$

Based on the results from NMR, fluorescence, and UV titration, it was revealed that the phenolic compound **B** and the Py·HBr catalyst **C** formed the intermediate **BC** after the equilibrium. The equilibrium constant of step 1 can be written as:

$$K = \frac{[BC]^{\delta}}{[B]^{\beta}[C]^{\gamma}} \qquad \textbf{Eq.S1}$$

$$\ln K = \delta \ln[BC] - \beta \ln[B] - \gamma \ln[C] \qquad \textbf{Eq.S2}$$

For two reactions with different concentrations of **B** and the same concentration of **C**

$$\delta \ln \frac{[BC]_2}{[BC]_1} = \beta \ln\left(\frac{[B]_2}{[B]_1}\right) + \gamma \ln\left(\frac{[C]_2}{[C]_1}\right) \text{ and } \ln\left(\frac{[C]_2}{[C]_1}\right) = 0 \qquad \textbf{Eq.S3}$$

According to LB's law:

$$A_{BC} = \varepsilon l[\text{BC}] \qquad \textbf{Eq.S4}$$

Eq.S3 can be written as:

$$\delta \ln \frac{A_{BC1}}{A_{BC2}} = \beta \ln \frac{[B]_1}{[B]_2} \qquad \textbf{Eq.S5}$$

In the same way, for two reactions with the same concentration of **B** and different concentrations of **C**, there are:

$$\delta \ln \frac{A_{BC3}}{A_{BC4}} = \gamma \ln \frac{[C]1}{[C]2} \qquad \textbf{Eq.S6}$$

According to the results of the UV titration experiments (Page S37, Figure S4b and S4d), when the **C** concentration is fixed, the **B** concentration has a linear relationship with the **BC** absorbance. When the **B** concentration is fixed, the **C** concentration and the **BC** absorbance also have a linear relationship. As a result, $\beta = \gamma = \delta$

According to the ^{1}H NMR spectrum of the product, the ratio of **A** and **B** is 1 : 1, so the consumption rate of **B** for the entire reaction is equal to the consumption rate of **A**, so the order of **A** is the same as the order of **B**. To sum up, $\alpha = \beta = \gamma = \delta = 1$

Chemical kinetics

step 1: $B + C \underset{k_{-1}}{\overset{k_1}{\rightleftharpoons}} BC$

step 2: $BC + A \xrightarrow{k_2} AB + C$

The kinetics of pyridinium bromide-catalyzed reactions are following the model of the consecutive two-step reactions involving a pre-equilibrium to form the intermediate **BC** from **B** and **C**. From the former section, we have determined that all species involved in the transformation are following the first order, thus, we can write the following formula:

$$[BC] = K[B][C] \quad \textbf{Eq.S7}$$

The reaction rate of the entire process can be written as:

$$r = \frac{d[AB]}{dt} = -\frac{d[A]}{dt} = k_2[A][BC] = k_2K[A][B][C] \quad \textbf{Eq.S8}$$

Since only the decrease in absorbance of **BC** with time can be observed during the reaction, that is $\frac{dA_{[BC]}}{dt}$

According to LB's law:

$$A_{[BC]} = \varepsilon l[BC] \quad \textbf{Eq.S9}$$

The absorbance of **BC** can be expressed as:

$$A_{[BC]} = \varepsilon lK[B][C] \quad \textbf{Eq.S10}$$

Herein, we define V_0 as the rate of the absorbance change of **BC** intermediate, therefore, a following formula can be obtained.

$$V_0 = -\frac{dA_{[BC]}}{dt} = -\varepsilon lK * ([C] * \frac{d[B]}{dt} + [B] * \frac{d[C]}{dt}) \quad \textbf{Eq.S11}$$

Now assuming that the catalyst concentration [C] remains unchanged during the reaction, **Eq.S11** can be approximated as:

$$V_0 = -\frac{dA_{[BC]}}{dt} = -\varepsilon lK[C] * \frac{d[B]}{dt} \quad \textbf{Eq.S12}$$

Eq.S8 can also be written as:

$$r = -\frac{d[B]}{dt} = -\frac{1}{\varepsilon lK[C]} * \frac{dA_{[BC]}}{dt} = k_2K[A][B][C] \quad \textbf{Eq.S13}$$

As a result, the rate of the absorbance change of **BC** intermediate (V_0) can be written as:

$$V_0 = -\frac{dA_{[BC]}}{dt} = \varepsilon lK[C]k_2K[A][B][C] \quad \textbf{Eq.S14}$$

Then, we define k_{obs} based on the following relationship:

$$k_{obs} = k_2K \quad \textbf{Eq.S15}$$

Therefore,

$$r = k_{obs}[A][B][C] \quad \textbf{Eq.S16}$$

Thus, **Eq.S14** can be written as:

$$V_0 = -\frac{dA_{[BC]}}{dt} = \varepsilon lK[C]k_{obs}[A][B][C] \quad \textbf{Eq.S17}$$

Then, we define the k_{Aobs}:

$$k_{Aobs} = \frac{-\frac{dA_{[BC]}}{dt}}{[A][B][C]} = \frac{V_0}{[A][B][C]} \quad \textbf{Eq.S18}$$

Finally, we receive the relationship between the k_{Aobs} and k_{obs}

$$k_{obs} = k_{Aobs}/\varepsilon lK[C] \quad \textbf{Eq.S19}$$

And k_2 of the second step can be received from the following equation:

$$k_2 = \frac{k_{obs}}{K} = \frac{k_{Aobs}}{K(\varepsilon lK[C])} \quad \textbf{Eq.S20}$$

The UV-Vis titration of 2 and catalyst mixture in toluene

In this section, we set two groups of UV-Vis titration experiments by varying the concentration of **2** (0.025 M, 0.0375 M, 0.05 M and 0.1 M), but with the same concentration of **A** (or **J**) (0.075 M). Then, we can obtain the concentration-dependent absorbance of **A···2** (or **J···2-*d***), according to **Eq.S12** $\frac{dA_{[BC]}}{dt} = \varepsilon lK[C] * \frac{d[B]}{dt}$, we can determine the slope of linear fitting is *εlK*[C].

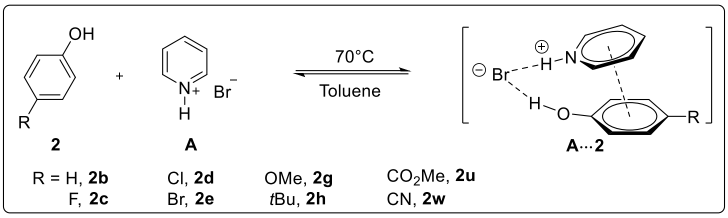

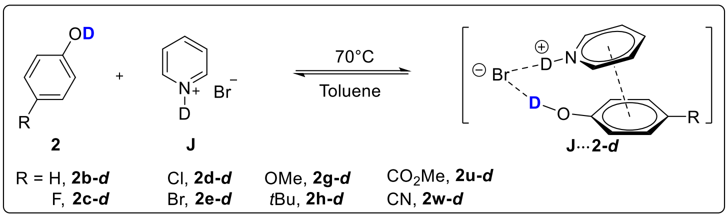

Table S5. The *εlK[C]* of mixture **2+A** and **2+J**

**Entry**	**Mixture**	$\varepsilon lK[C]$	**Entry**	**Mixture**	$\boldsymbol{\varepsilon lK[C]}$
1	**2b+A**	13.84911	9	**2b-*d*+J**	13.81161
2	**2c+A**	11.00786	10	**2c-*d*+J**	11.07621
3	**2d+A**	11.0405	11	**2d-*d*+J**	10.8611
4	**2e+A**	8.19312	12	**2e-*d*+J**	8.33322
5	**2g+A**	4.09764	13	**2g-*d*+J**	4.09727
6	**2h+A**	6.99749	14	**2h-*d*+J**	6.92365
7	**2u+A**	12.66093	15	**2u-*d*+J**	12.63792
8	**2w+A**	7.16986	16	**2w-*d*+J**	7.07978

Note: the titration spectra were not listed in SI file for simplicity.

Kinetic monitoring

The kinetic profiles for other *para*-substituted phenols (H, F, Cl, Br, OMe, *t*Bu, CO_2Me, CN) were recorded through the measurement of absorbance of intermediate catalyst-phenol (**cat···2**, **Int.1**) every half hour for the first 2 hours, and every one hour for the next 6-7 hours. Due to the large amount of data, herein, only two kinetic profiles of the reaction for substrate **2w** and **2w-*d*** are listed respectively as examples. The time-dependent UV-Vis spectra at 330 nm was recorded.

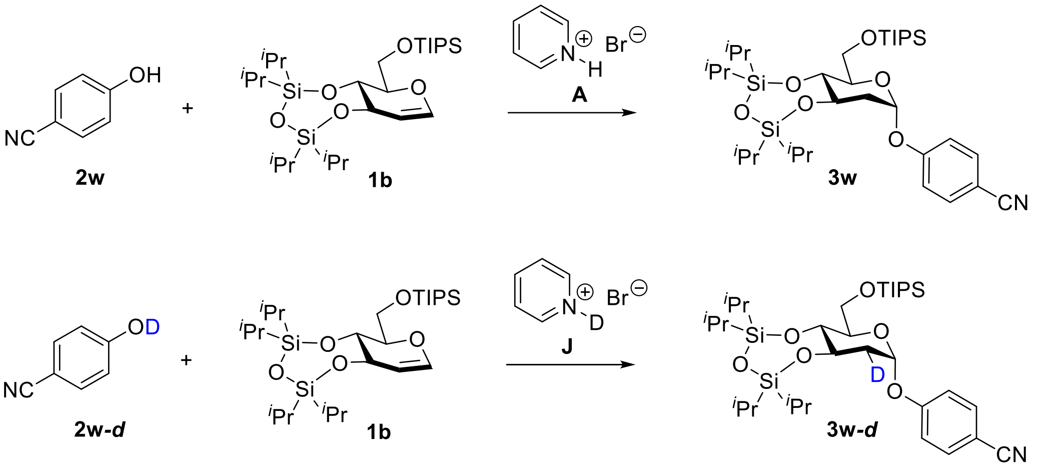

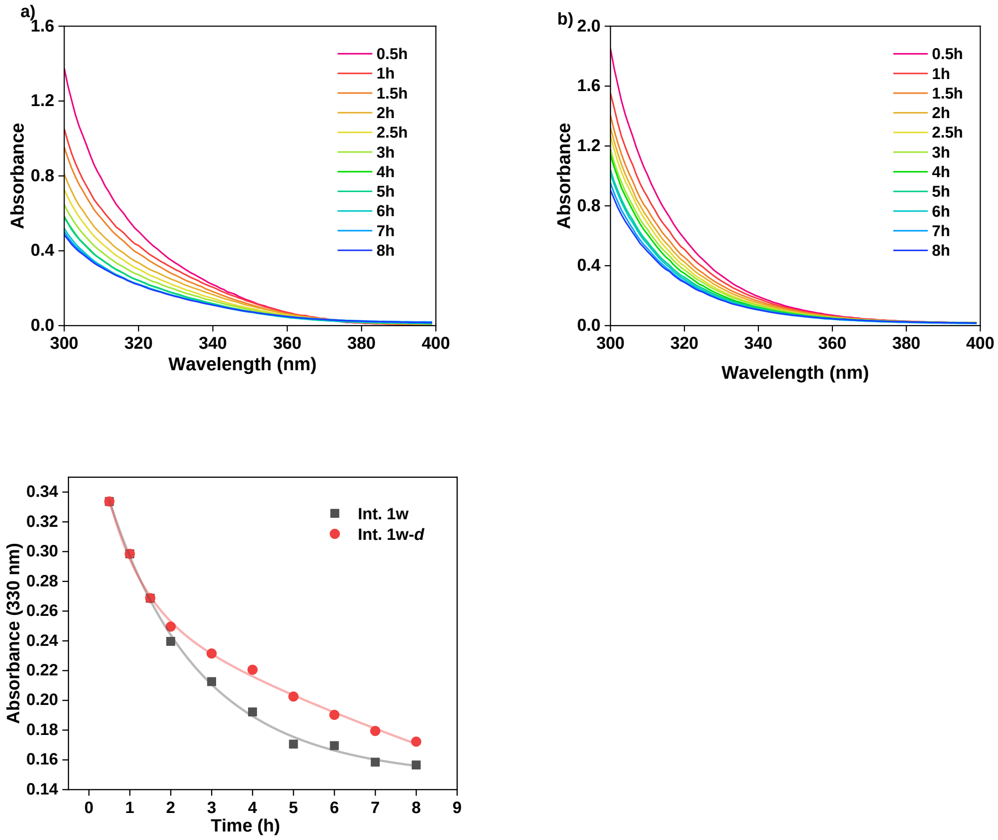

Figure S6. a) Time-dependent UV-Vis spectra of **Int. 1w**; a) Time-dependent UV-Vis spectra of **Int. 1w -*d***; c) Time-dependent absorbance at 330 nm of compound **Int. 1w** and **Int. 1w-*d*** in 8h.

The determination of the V_0

With the kinetic monitoring profiles in hand, we are able to obtain the initial rates for each reaction by linearly fitting the first three points (1.5 h). All fittings are listed in the following part. According to the **Eq.S14** ($V_0 = -\frac{dA_{[BC]}}{dt} = \varepsilon l K[C]k_2 K[A][B][C]$), we can know the slop for the fitting formula is -V_0. The wavelength of measurement is depended on the phenol substrate. In detail, 315 nm is used for **2c, 2d, 2h, 2u** and their deuterated analogs; 300 nm is used for **2b** and its deuterated analogs; 320 nm is used for **2e** and its deuterated analogs; 330 nm is used for **2g**, **2w** and their deuterated analogs.

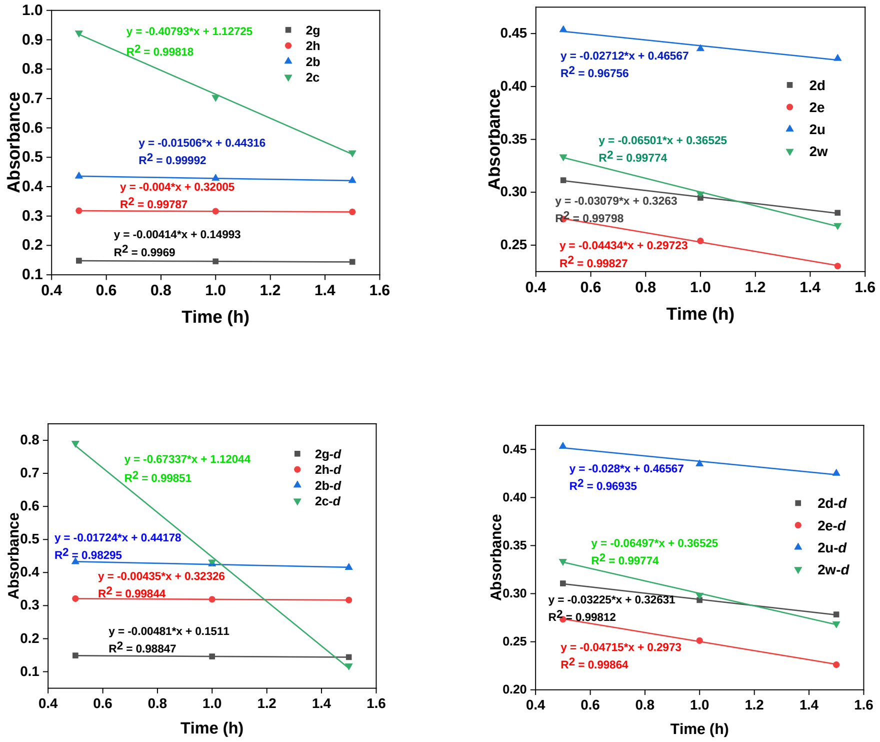

Figure S7. Time-dependent absorbance of compound in first 1.5h. a) Compound **2g**, **2h**, **2b**, **3c**; b) Compound **2d**, **2e**, **2u**, **2w**; C) Compound **2g-*d***, **2h-*d***, **2b-*d***, **2h-*d***; d) Compound **2d-*d***, **2e-*d***, **2w-*d***, **2u-*d***.

From the results in Figure S7, then we can obtain the value of V_0 (slope = -V_0) for each reaction.

Table S6. The initial reaction rate of a compound

Entry	V_0 (A.U./h)	Entry	V_0 (A.U./h)
**2b**	0.01506	**2b-*d***	0.01724
**2c**	0.40793	**2c-*d***	0.67337
**2d**	0.03079	**2d-*d***	0.03225
**2e**	0.04434	**2e-*d***	0.04715
**2g**	0.00414	**2g-*d***	0.00481
**2h**	0.00400	**2h-*d***	0.00435
**2u**	0.02712	**2u-*d***	0.02800
**2w**	0.06501	**2w-*d***	0.06497

The determination of the k_2

According to the following formula, we can calculate the rate constant for the step 2,

$$k_2 = \frac{k_{obs}}{K} = \frac{k_{Aobs}}{K(\varepsilon lK[C])} = \frac{V_0}{[A][B][C]K(\varepsilon lK[C])}$$

From Table S5 (page 42) and Table S6 (page 44), $\varepsilon lK[C]$ and V_0 are known.

From the DFT calculation, the equilibrium constant K can be calculated (Table S12, page 54). Besides, the initial concentrations for three components of the reaction are known, for these kinetics, the initial concentration of phenol **2** is 0.05 M, the initial concentration of catalyst is 0.01 M, the initial concentration of glycal **1b** is 0.05 M. Therefore, k_2 can be calculated and listed in the following table.

Table S7. Calculation of k_2

Substrate	ΔG (kcal/mol)	ln*K*	*K*	V_0 (A.U./h)	V_0/3600 (A.U./s)	V_0/3600 /([A][B][C])	$\varepsilon lK[C]$	k_2
**2b**	2.661	-3.90	0.0201	0.01506	$4.18*10^{-6}$	0.16733	13.84	**0.5988**
**2c**	0.191	-0.28	0.7560	0.40793	$1.13*10^{-4}$	4.53256	11.00	**0.5446**
**2d**	2.068	-3.03	0.0481	0.03079	$8.55*10^{-6}$	0.34209	11.04	**0.6433**
**2e**	1.608	-2.35	0.0946	0.04434	$1.23*10^{-5}$	0.49267	8.19	**0.6353**
**2g**	2.592	-3.80	0.0223	0.00414	$1.15*10^{-6}$	0.04604	4.09	**0.5028**
**2h**	3.013	-4.41	0.0120	0.00400	$1.11*10^{-6}$	0.04441	6.99	**0.5266**
**2u**	2.270	-3.3	0.0358	0.02712	$7.53*10^{-6}$	0.30129	12.66	**0.6641**
**2w**	1.352	-1.98	0.1376	0.06501	$1.80*10^{-5}$	0.72232	7.16	**0.7320**
**2b-*d***	2.433	-3.57	0.0281	0.01724	$4.79*10^{-6}$	0.19159	13.811	**0.4923**
**2c-*d***	-0.304	0.445	1.5608	0.67337	$1.87*10^{-4}$	7.48191	11.07	**0.4327**
**2d-*d***	1.852	-2.71	0.0660	0.03225	$8.96*10^{-6}$	0.35835	10.86	**0.4992**
**2e-*d***	1.398	-2.05	0.1285	0.04715	$1.31*10^{-5}$	0.52388	8.33	**0.4889**
**2g-*d***	2.364	-3.46	0.0311	0.00481	$1.33*10^{-6}$	0.05346	4.09	**0.4184**
**2h-*d***	2.797	-4.10	0.0165	0.00435	$1.21*10^{-6}$	0.04839	6.92	**0.4224**
**2u-*d***	2.056	-3.01	0.0490	0.02800	$7.77*10^{-6}$	0.3111	12.63	**0.5021**
**2w-*d***	1.142	-1.67	0.1874	0.06497	$1.80*10^{-6}$	0.72183	7.079	**0.5440**

Hammett Plot

Table S8. Calculation for Hammett plot

Substrate	k_2	Functional group	k_H/k_X	$lg(k_H/k_X)$	σ-[8]
**2b**	0.5988	H	1	0	0
**2c**	0.5446	F	0.909	-0.0412	-0.03
**2d**	0.6433	Cl	1.074	0.0311	0.19
**2e**	0.6353	Br	1.061	0.0257	0.25
**2g**	0.5028	OMe	0.840	-0.0759	-0.26
**2h**	0.5266	*t*Bu	0.879	-0.0558	-0.13
**2u**	0.6641	CO_2Me	1.109	0.0449	0.75
**2w**	0.7320	CN	1.222	0.0872	1

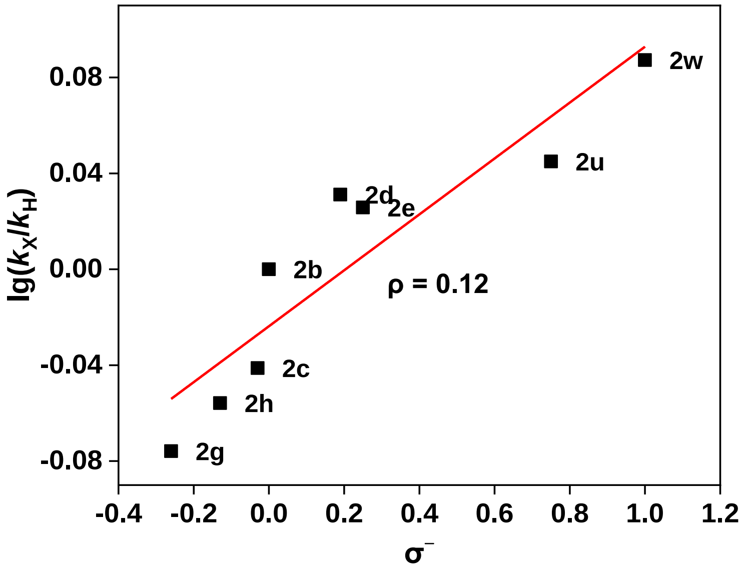

Figure S8. Hammett plot

The determination of relationship between k_2 and KIE

Table S9. KIE of the reaction

Substrate	k_2 ($M^{-1}s^{-1}$)	Substrate	k_2 ($M^{-1}s^{-1}$)	KIE
**2g**	0.502851	**2g-*d***	0.41842	1.2018
**2h**	0.526605	**2h-*d***	0.42240	1.2467
**2b**	0.598803	**2b-*d***	0.49234	1.2162
**2c**	0.544629	**2c-*d***	0.43277	1.2585
**2d**	0.643312	**2d-*d***	0.49927	1.2885
**2e**	0.635366	**2e-*d***	0.48893	1.2995
**2u**	0.664126	**2u-*d***	0.50218	1.3225
**2w**	0.732061	**2w-*d***	0.54407	1.3455

The theoretical equilibrium isotope effect (EIE) if the reaction proceeded through a stepwise protonation mechanism.

The predicted equilibrium isotope effect (EIE = $k_{obs(H)}/k_{obs(D)}$) of the reactions can be calculated from the following equation[9]:

$$\mathrm{EIE} = \exp\{\frac{hc}{k_B T}\frac{((\overline{v_{O-D}} - \overline{v_{C-D}}) - (\overline{v_{O-H}} - \overline{v_{C-H}}))}{2}\}$$

Stretching wavenumbers $\tilde{v}_{\text{C-H}}$ and $\tilde{v}_{\text{C-D}}$ of *sp*^{3} C-H/C-D bond in the hexane[10] and $\tilde{v}_{\text{O-H}}$ and $\tilde{v}_{\text{O-D}}$ of OH/OD bond in the phenol[11,12] are listed in **Table S10**.

Table S10. IR Data of sp^3 C-H/C-D bond in hexane and OH/OD bond in **2b**

Bond types	Stretching wavenumbers/ cm^{-1}	
	$\tilde{v}_{X-H}$	$\tilde{v}_{X-D}$
C-H/C-D	2959	2216
O-H/O-D	3657	2650

We can use these data to calculate the expected EIE of the step-wise protonation reaction between *para*-substituted phenol and **2b**, which is 0.9944. If the second step is going through a step-wise protonation mechanism, the observed KIE should equal to EIE. Because the observed KIE value of k_2 is from 1.2~1.35 for different phenols, we can exclude stepwise mechanism.

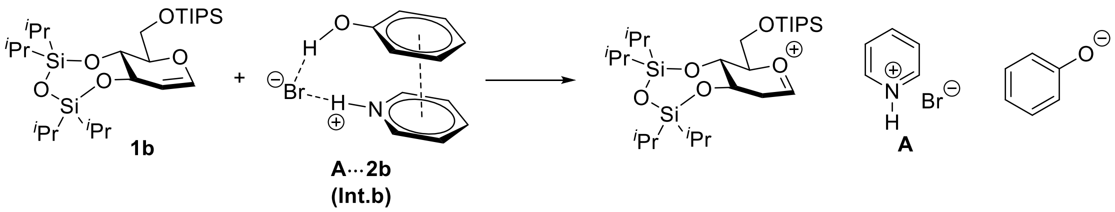

Figure S9. The step-wise protonation reaction between **1b** and **2b**.

NMR evidence for the cis-concerted mechanism.

In the glovebox, **2h** (3.78 mg, 0.025 mmol, 1.0 eq.), **2h-*d*** (3.78 mg, 0.025 mmol, 1.0 eq.), **1b** (20.44 mg, 0.0375 mmol, 1.5 eq.) and **A** (0.80 mg, 0.005 mmol, 0.20 eq.) was added into a J-Young NMR tube, then 0.5 mL of toluene-d_8 were added. The mixture reacted at 70 °C for 1 hour and the ^{1}H NMR spectrum was recorded as follows.

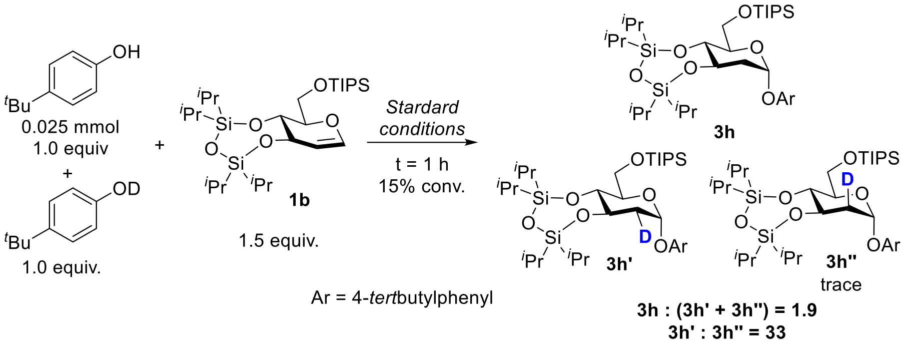

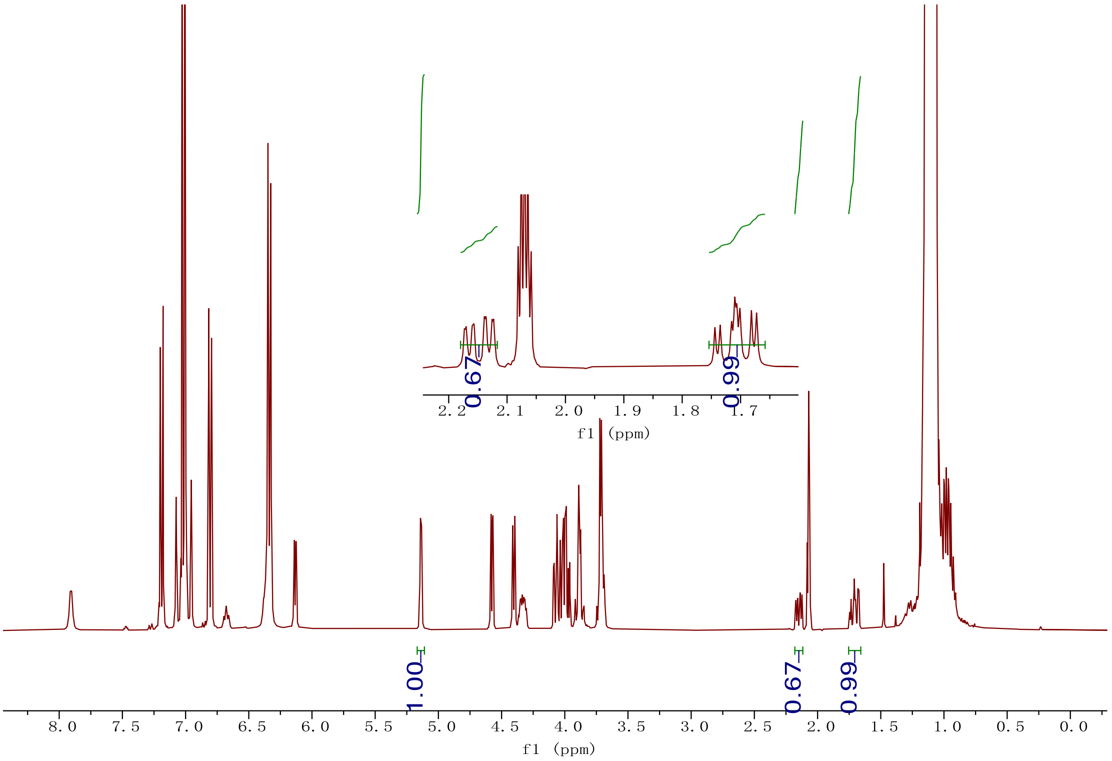

Figure S10. Competition experiment

The integral of 0.67 was for the equatorial proton of the CH_2 at C2 position, the integral of 0.99 was for the axial proton of the CH_2 at C2 position. Therefore, we can calculate the deuteration ratio for equatorial (0.33) and axial proton (0.01) at C2 position respectively. That is, the ratio of **3h'** to **3h''** is 33. This large value supports that the addition of phenol to glycal proceeds via a concerted process. The average KIE for H/D is 0.66/0.34 = 1.94.

DFT calculation data

All density functional theory (DFT) calculations were performed using Gaussian 16 package[13]. The geometries and frequency calculations were performed by the B3LYP[14] density functional in conjunction with the 6-31+G(d,p) basis set in toluene solution with the SMD continuum solvation models To obtain more accurate electronic energies, the single-point energy calculations were performed at the B2PLYPD3/def2TZVP level with the optimized structures. Structures are generated using GaussView。

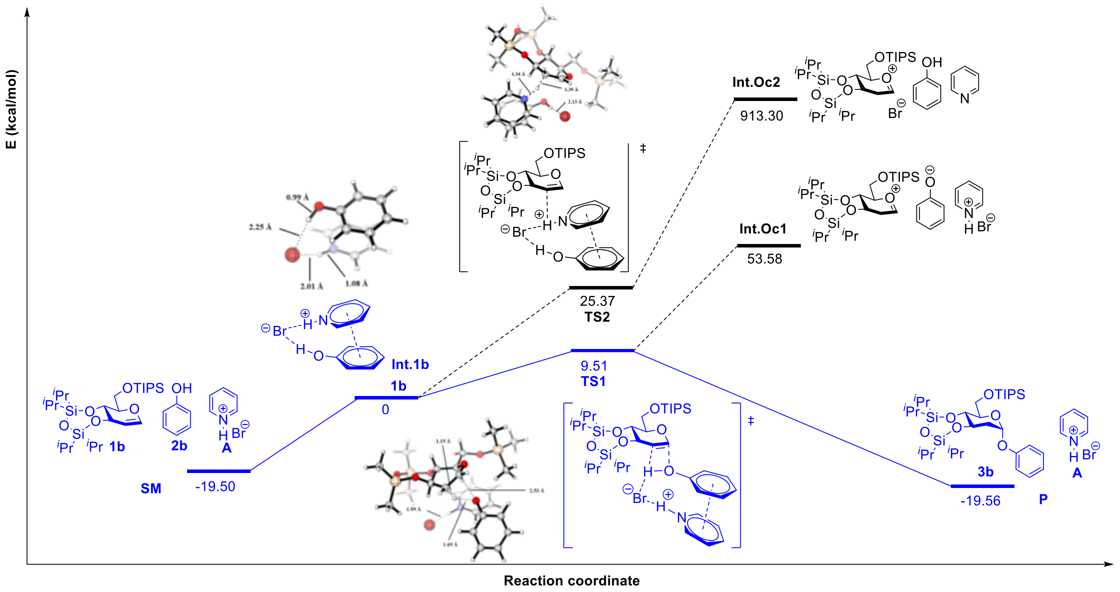

Figure S11. Computational mechanistic studies and the proposed mechanism.

Table S11. Overview of calculated structures

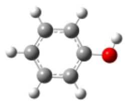	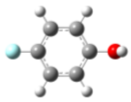	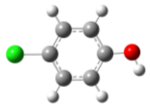
**2b**<br>E = -306.962929 Hartree	**2c**<br>E =-406.114473 Hartree	**2d**<br>E = -766.392163 Hartree
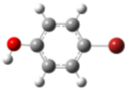	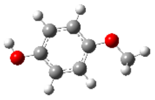	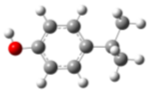
**2e**<br>E = -2880.055609 Hartree	**2g**<br>E =-421.316249 Hartree	**2h**<br>E = -463.898454 Hartree
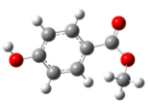	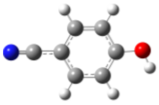	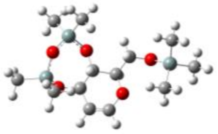
**2u**<br>E = -534.514047 Hartree	**2w**<br>E = -399.075938 Hartree	**1b**<br>E = -1757.276966 Hartree
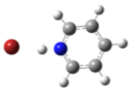	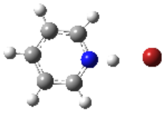	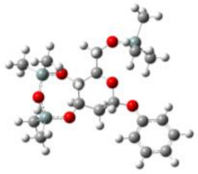
**A**<br>E = -2822.165744 Hartree	**J**<br>E = -2822.5768622 Hartree	**3b**<br>E = -2064.177662 Hartree
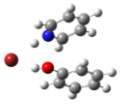	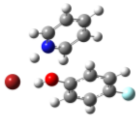	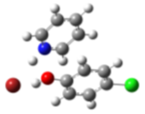
**A···2b (Int.1b)**<br>E = -3129.097604 Hartree	**A···2c (Int.1c)**<br>E = -3228.29733 Hartree	**A···2d (Int.1d)**<br>E = -3588.570745 Hartree
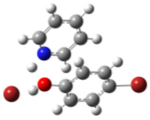	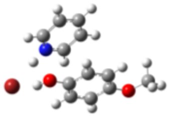	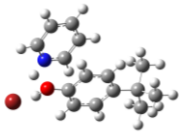
**A···2e (Int.1e)**<br>E = -5702.233525 Hartree	**A···2g (Int.1g)**<br>E = -3243.49349 Hartree	**A···2h (Int.1h)**<br>E = -3286.075413 Hartree
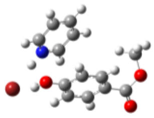	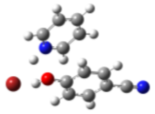	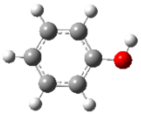
**A···2u (Int.1u)**<br>E = -3356.691803 Hartree	**A···2w (Int.1w)**<br>E = -3221.255265 Hartree	**2b-*d***<br>E = -307.3458669 Hartree

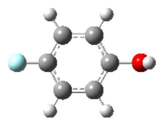	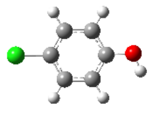	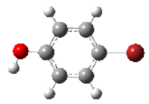
**2c-*d***<br>E = -406.573 Hartree	**2d-*d***<br>E = -766.8361434 Hartree	**2e-*d***<br>E = -2880.527152 Hartree
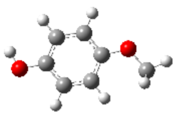	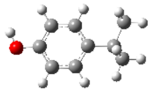	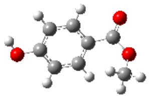
**2g-*d***<br>E = -421.8303231 Hartree	**2h-*d***<br>E = -464.5073866 Hartree	**2u-*d***<br>E = -535.1523161 Hartree
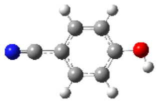	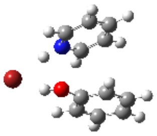	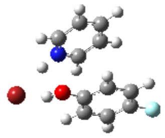
**2w-*d***<br>E = -399.5688309 Hartree	**J···2b-*d***<br>E = -3129.943216 Hartree	**J···2c-*d***<br>E = -3229.176757 Hartree
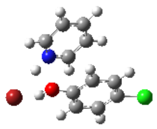	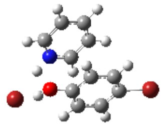	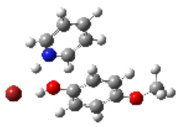
**J···2d-*d***<br>E = -3589.434692 Hartree	**J···2e-*d***<br>E = -5703.12572 Hartree	**J···2g-*d***<br>E = -3244.427527 Hartree
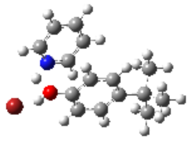	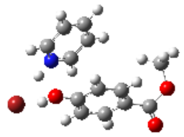	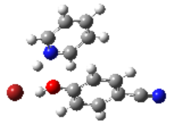
**J···2h-*d***<br>E = -3287.104968 Hartree	**J···2u-*d***<br>E = -3357.749296 Hartree	**J···2w-*d***<br>E = -3222.167978 Hartree
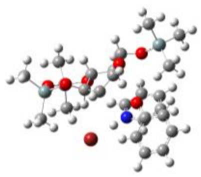	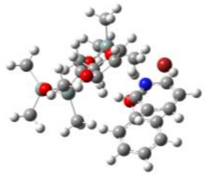	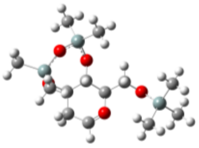
**TS1**<br>E = -4886.35942 Hartree	**TS2**<br>E = -4886.334002 Hartree	**Int.Oc1**<br>E = -1757.299864 Hartree
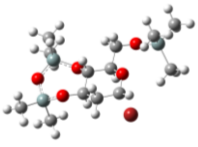	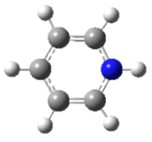	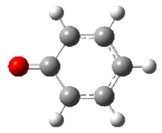
**Int.Oc2**<br>E = -4329.207397 Hartree	**Py**<br>E = -248.7488502	**2c**$^-$<br>E = -306.8235683

Table S12. Summary of single point energy (E) and G for compounds, intermediates and transition states in toluene

Entry	E (Hartree)	G (Hartree)	Entry	E (Hartree)	G (Hartree)
**2b**	-306.962929	-307.275355	**2b-*d***	-307.3458669	-307.2789859
**2c**	-406.114473	-406.513615	**2c-*d***	-406.573402	-406.516812
**2d**	-766.392163	-766.777944	**2d-*d***	-766.8361434	-766.7815754
**2e**	-2880.055609	-2880.47059	**2e-*d***	-2880.527152	-2880.474225
**2g**	-421.316249	-421.731875	**2g-*d***	-421.8303231	-421.7354991
**2h**	-463.898454	-464.332705	**2h-*d***	-464.5073866	-464.3363356
**2u**	-534.514047	-535.047351	**2u-*d***	-535.1523161	-535.0509861
**2w**	-399.075938	-399.50283	**2w-*d***	-399.5688309	-399.5064669
**A···2b (Int.1b)**	-3129.097604	-3129.7873	**J···2b-*d* (Int.1b-*d*)**	-3129.943216	-3129.794181
**A···2c (Int.1c)**	-3228.29733	-3229.0295	**J···2c-*d* (Int.1c-*d*)**	-3229.176757	-3229.036369
**A···2d (Int.1d)**	-3588.570745	-3589.2909	**J···2d-*d* (Int.1d-*d*)**	-3589.434692	-3589.297696
**A···2e (Int.1e)**	-5702.233525	-5702.9843	**J···2e-*d* (Int.1e-*d*)**	-5703.12572	-5702.991069
**A···2g (Int.1g)**	-3243.49349	-3244.244	**J···2g-*d* (Int.1g-*d*)**	-3244.427527	-3244.250804
**A···2h (Int.1h)**	-3286.075413	-3286.8441	**J···2h-*d* (Int.1h-*d*)**	-3287.104968	-3286.850952
**A···2u (Int.1u)**	-3356.691803	-3357.56	**J···2u-*d* (Int.1u-*d*)**	-3357.749296	-3357.566783
**A···2w (Int.1w)**	-3221.255265	-3222.0169	**J···2w-*d* (Int.1w-*d*)**	-3222.167978	-3222.023721
**A**	-2822.165744	-2822.51623	**J**	-2822.576862	-2822.519073
**1b**	-1757.276966	-1756.94765	**3b**	-2064.177662	-2063.752245
**TS1**	-4886.35942		**TS2**	-4886.334002	
**Int.Oc1**	-1757.299864		**Int.Oc2**	-4329.207397	
**Pyridine**	-248.7488502		**2c^{-}**	-306.8235683	

Table S13. The ΔG and *K* of **2** and **2-*d***

Entry	ΔG (kcal/mol)	*K* (M^{-1})	Entry	ΔG (kcal/mol)	*K* (M^{-1})
**2b**	2.661	0.0202	**2b-*d***	2.433	0.0282
**2c**	0.191	0.7560	**2c-*d***	-0.304	1.5608
**2d**	2.068	0.0482	**2d-*d***	1.852	0.0661
**2e**	1.608	0.0946	**2e-*d***	1.398	0.1286
**2g**	2.592	0.0223	**2g-*d***	2.364	0.0312
**2h**	3.013	0.0121	**2h-*d***	2.797	0.0165
**2u**	2.27	0.0358	**2u-*d***	2.056	0.0490
**2w**	1.352	0.1376	**2w-*d***	1.142	0.1874

$\Delta G = \Delta G_{\mathbf{Int.1}} - \Delta G_{\mathbf{2}} - \Delta G_{\mathbf{Cat.A}}$

$\ln K = -\Delta G/RT$

Coordinates and total energies

2b E=-306.962929 Hartree

C	-0.21966300	-1.22436200	0.00001000
C	-0.94140600	-0.02569200	0.00003300
C	-0.26589200	1.19909900	0.00002000
C	1.12901100	1.21968700	0.00000400
C	1.85609600	0.02943700	-0.00001500
C	1.17227600	-1.18916800	0.00000400
H	-0.76173800	-2.16413400	-0.00003600
H	-0.82976200	2.12922500	-0.00001900
H	1.64586300	2.17480100	-0.00003300
H	2.94104100	0.04967100	-0.00001900
H	1.72691100	-2.12291100	-0.00003300
O	-2.30357100	-0.11261800	-0.00000700
H	-2.67627700	0.78028100	-0.00013100

2c E =-406.114473 Hartree

C	-0.68018100	-1.21083400	-0.01351300
C	-1.37718600	-0.00010400	-0.01506900
C	-0.68031000	1.21069100	-0.01368400
C	0.71428800	1.21637500	-0.00218700
C	1.38531000	0.00004800	0.00502300
C	0.71440400	-1.21636200	-0.00212800
H	-1.23700300	-2.14186800	-0.02755200
H	-1.23715000	2.14171700	-0.02792900
H	1.27877900	2.14217800	-0.00161100
H	1.27903500	-2.14208000	-0.00147100
O	-2.76003500	-0.00034700	-0.08225700
H	-3.11661000	0.00307700	0.81726400
F	2.73947500	0.00009600	0.01652300

2d E= -3588.570745 Hartree

C	-1.11655400	-1.22323400	0.00007200
C	-1.82446600	-0.01636600	-0.00015800
C	-1.13110500	1.19836800	-0.00014700
C	0.26273200	1.20911400	0.00008200
C	0.95469700	0.00155700	-0.00007600
C	0.27450800	-1.21565300	-0.00005300
H	-1.66616600	-2.15813200	0.00018500
H	-1.67697100	2.13837600	-0.00009600
H	0.80197400	2.14946900	0.00022400
H	0.82578100	-2.14901600	-0.00000900
O	-3.18475100	-0.08776900	0.00000500
H	-3.55151500	0.80769100	0.00085700

Cl	2.71917800	0.01123800	0.00002800

2e E= -5702.233525 Hartree

C	-1.76416900	-1.22344700	-0.00001300
C	-2.47034200	-0.01525100	0.00013000
C	-1.77510500	1.19847500	0.00003200
C	-0.38114800	1.20648900	-0.00007700
C	0.30897600	-0.00155600	0.00000700
C	-0.37305700	-1.21727600	-0.00003800
H	-2.31476000	-2.15781300	-0.00020200
H	-2.31938300	2.13951900	-0.00000100
H	0.15769000	2.14672700	-0.00020400
H	0.17543700	-2.15199200	-0.00003400
O	-3.83057500	-0.08441900	-0.00000400
H	-4.19592800	0.81166500	-0.00012300
Br	2.22487500	0.00579000	0.00001000

2g E= -421.316249 Hartree

C	-0.96836800	1.21772500	0.00002000
C	-1.84493700	0.13346000	0.00001900
C	-1.32807000	-1.16753900	0.00002000
C	0.04687500	-1.37435000	0.00001900
C	0.92893700	-0.28726500	0.00000000
C	0.41248500	1.01315700	0.00000500
H	-1.37452800	2.22372900	0.00000300
H	-2.00222100	-2.02116600	-0.00000400
H	0.45658300	-2.37887500	0.00001600
H	1.07000400	1.87367900	-0.00002900
O	-3.18858500	0.39716600	-0.00002500
H	-3.67144000	-0.44075600	-0.00008100
O	2.26168200	-0.59648900	-0.00003800
C	3.18614600	0.48036100	0.00000400
H	3.07950100	1.10863800	0.89392700
H	4.17925000	0.02749800	0.00011600
H	3.07967300	1.10854100	-0.89398100

2h E= -463.898454 Hartree

C	1.75682800	-1.21179500	0.00006300
C	2.48070500	-0.01484100	0.00001100
C	1.79345300	1.19884200	0.00006200
C	0.39683700	1.21219900	0.00011400
C	-0.35103300	0.03034400	0.00000800
C	0.36777700	-1.17785600	0.00007400
H	2.29486500	-2.15410400	0.00014800

H	2.34526000	2.13640000	0.00008000
H	-0.10217000	2.17393000	0.00026600
H	-0.16837400	-2.12188900	0.00019400
O	3.84404600	-0.09716500	-0.00009700
H	4.21161500	0.79780100	-0.00038000
C	-1.88615000	0.00937800	-0.00002000
C	-2.48581300	1.42427000	0.00057000
H	-3.57871600	1.36208500	0.00096200
H	-2.18770800	1.99245700	0.88772700
H	-2.18841600	1.99293000	-0.88650900
C	-2.39016400	-0.72895600	1.25813700
H	-3.48563100	-0.75638800	1.27384000
H	-2.03159400	-1.76186000	1.29365700
H	-2.04929000	-0.22601600	2.16901000
C	-2.39010400	-0.72789400	-1.25886100
H	-2.04922000	-0.22411700	-2.16927500
H	-2.03145300	-1.76072900	-1.29528800
H	-3.48555000	-0.75533500	-1.27459500

2u E = -3356.691803 Hartree

C	-1.53434000	1.13008000	0.51073200
C	-2.37249700	0.13693300	-0.01088100
C	-1.82959800	-1.07495900	-0.45753900
C	-0.45939600	-1.28566200	-0.38111700
C	0.39493500	-0.28856400	0.10897400
C	-0.16356000	0.91664900	0.56193500
H	-1.97320400	2.05283200	0.87397300
H	-2.48232900	-1.84897400	-0.85327700
H	-0.03360700	-2.22972000	-0.70253500
H	0.47183500	1.68494700	0.98717000
O	-3.70367200	0.40156800	-0.05377700
H	-4.17148000	-0.36341700	-0.41893300
C	1.84523000	-0.60955200	0.22736800
O	2.76292800	0.38032700	0.07194500
C	2.51189400	1.53380700	-0.74560100
H	2.30287700	2.40927300	-0.12416400
H	3.42921700	1.71296400	-1.31155900
H	1.68506700	1.37247900	-1.44007100
O	2.24269400	-1.72474100	0.49510500

2w E= -3221.255265 Hartree

C	-1.00738900	-1.22725200	0.00004600
C	-1.71257500	-0.01557300	-0.00001000
C	-1.02068500	1.20329000	0.00009900

C	0.36720700	1.21128700	0.00009400
C	1.07998200	0.00102500	0.00008500
C	0.37805000	-1.21856300	0.00014900
H	-1.56164100	-2.15900000	0.00005600
H	-1.57010100	2.14085100	0.00021000
H	0.90569100	2.15240900	0.00015000
H	0.92785600	-2.15315600	0.00022700
O	-3.06607700	-0.08625100	-0.00018600
H	-3.43837100	0.80769600	-0.00029200
C	2.50849400	0.00953000	-0.00002800
N	3.67238200	0.01696400	-0.00021000

2b-*d* E = -307.3458669 Hartree

C	0.21966300	-1.22436200	-0.00001000
C	0.94140600	-0.02569200	-0.00003300
C	0.26589200	1.19909900	-0.00002000
C	-1.12901100	1.21968700	-0.00000400
C	-1.85609600	0.02943700	0.00001500
C	-1.17227600	-1.18916800	-0.00000400
H	0.76173800	-2.16413400	0.00003600
H	0.82976200	2.12922500	0.00001900
H	-1.64586300	2.17480100	0.00003300
H	-2.94104100	0.04967100	0.00001900
H	-1.72691100	-2.12291100	0.00003300
O	2.30357100	-0.11261700	0.00000700
H(Iso=2)	2.67627700	0.78028200	0.00013100

2c-*d* E = -406.573 Hartree

C	0.68018100	1.21083400	-0.01351300
C	1.37718600	0.00010400	-0.01506900
C	0.68031000	-1.21069100	-0.01368400
C	-0.71428800	-1.21637500	-0.00218700
C	-1.38531000	-0.00004800	0.00502300
C	-0.71440400	1.21636200	-0.00212800
H	1.23700300	2.14186800	-0.02755200
H	1.23715000	-2.14171700	-0.02792900
H	-1.27877900	-2.14217800	-0.00161100
H	-1.27903500	2.14208000	-0.00147100
O	2.76003500	0.00034700	-0.08225700
H(Iso=2)	3.11661000	-0.00307700	0.81726400
F	-2.73947500	-0.00009600	0.01652300

2d-*d* E = -766.8361434 Hartree

C	1.11655400	1.22323400	0.00007200

C	1.82446600	0.01636600	-0.00015800
C	1.13110500	-1.19836800	-0.00014700
C	-0.26273200	-1.20911400	0.00008200
C	-0.95469700	-0.00155700	-0.00007600
C	-0.27450800	1.21565300	-0.00005300
H	1.66616600	2.15813200	0.00018500
H	1.67697100	-2.13837600	-0.00009600
H	-0.80197400	-2.14946900	0.00022400
H	-0.82578100	2.14901600	-0.00000900
O	3.18475100	0.08776900	0.00000500
H(Iso=2)	3.55151500	-0.80769100	0.00085700
Cl	-2.71917800	-0.01123800	0.00002800

2e-*d* E = -2880.527152 Hartree

C	-1.76416900	1.22344700	0.00001300
C	-2.47034200	0.01525100	-0.00013000
C	-1.77510500	-1.19847500	-0.00003200
C	-0.38114800	-1.20648900	0.00007700
C	0.30897600	0.00155600	-0.00000700
C	-0.37305700	1.21727600	0.00003800
H	-2.31476000	2.15781300	0.00020200
H	-2.31938300	-2.13951900	0.00000100
H	0.15769000	-2.14672700	0.00020400
H	0.17543700	2.15199200	0.00003400
O	-3.83057500	0.08441900	0.00000400
H(Iso=2)	-4.19592800	-0.81166500	0.00012300
Br	2.22487500	-0.00579000	-0.00001000

2g-*d* E = -421.8303231 Hartree

C	-0.96836800	-1.21772500	-0.00002000
C	-1.84493700	-0.13346000	-0.00001900
C	-1.32807000	1.16753900	-0.00002000
C	0.04687500	1.37435000	-0.00001900
C	0.92893700	0.28726500	0.00000000
C	0.41248500	-1.01315700	-0.00000500
H	-1.37452800	-2.22372900	-0.00000300
H	-2.00222100	2.02116600	0.00000400
H	0.45658300	2.37887500	-0.00001600
H	1.07000400	-1.87367900	0.00002900
O	-3.18858500	-0.39716600	0.00002500
H(Iso=2)	-3.67144000	0.44075600	0.00008100
O	2.26168200	0.59648900	0.00003800
C	3.18614600	-0.48036100	-0.00000400
H	3.07950100	-1.10863800	-0.89392700

H	4.17925000	-0.02749800	-0.00011600
H	3.07967300	-1.10854100	0.89398100

2h-*d* E = -464.5073866 Hartree

C	-1.75682800	-1.21179500	-0.00006300
C	-2.48070500	-0.01484100	-0.00001100
C	-1.79345300	1.19884200	-0.00006200
C	-0.39683700	1.21219900	-0.00011400
C	0.35103300	0.03034400	-0.00000800
C	-0.36777700	-1.17785600	-0.00007400
H	-2.29486500	-2.15410400	-0.00014800
H	-2.34526000	2.13640000	-0.00008000
H	0.10217000	2.17393000	-0.00026600
H	0.16837400	-2.12188900	-0.00019400
O	-3.84404600	-0.09716500	0.00009700
H(Iso=2)	-4.21161500	0.79780100	0.00038000
C	1.88615000	0.00937800	0.00002000
C	2.48581300	1.42427000	-0.00057000
H	3.57871600	1.36208500	-0.00096200
H	2.18770800	1.99245700	-0.88772700
H	2.18841600	1.99293000	0.88650900
C	2.39016400	-0.72895600	-1.25813700
H	3.48563100	-0.75638800	-1.27384000
H	2.03159400	-1.76186000	-1.29365700
H	2.04929000	-0.22601600	-2.16901000
C	2.39010400	-0.72789400	1.25886100
H	2.04922000	-0.22411700	2.16927500
H	2.03145300	-1.76072900	1.29528800
H	3.48555000	-0.75533500	1.27459500

2u-*d* E = -535.1523161 Hartree

C	-1.53434000	-1.13008000	-0.51073200
C	-2.37249700	-0.13693300	0.01088100
C	-1.82959800	1.07495900	0.45753900
C	-0.45939600	1.28566200	0.38111700
C	0.39493500	0.28856400	-0.10897400
C	-0.16356000	-0.91664900	-0.56193500
H	-1.97320400	-2.05283200	-0.87397300
H	-2.48232900	1.84897400	0.85327700
H	-0.03360700	2.22972000	0.70253500
H	0.47183500	-1.68494700	-0.98717000
O	-3.70367200	-0.40156800	0.05377700
H(Iso=2)	-4.17148000	0.36341700	0.41893300
C	1.84523000	0.60955200	-0.22736800

O	2.76292800	-0.38032700	-0.07194500
C	2.51189400	-1.53380700	0.74560100
H	2.30287700	-2.40927300	0.12416400
H	3.42921700	-1.71296400	1.31155900
H	1.68506700	-1.37247900	1.44007100
O	2.24269400	1.72474100	-0.49510500

2w-*d* E = -399.5688309 Hartree

C	1.00738900	1.22725200	0.00004600
C	1.71257500	0.01557300	-0.00001000
C	1.02068500	-1.20329000	0.00009900
C	-0.36720700	-1.21128700	0.00009400
C	-1.07998200	-0.00102500	0.00008500
C	-0.37805000	1.21856300	0.00014900
H	1.56164100	2.15900000	0.00005600
H	1.57010100	-2.14085100	0.00021000
H	-0.90569100	-2.15240900	0.00015000
H	-0.92785600	2.15315600	0.00022700
O	3.06607700	0.08625100	-0.00018600
H(Iso=2)	3.43837100	-0.80769600	-0.00029200
C	-2.50849400	-0.00953000	-0.00002800
N	-3.67238200	-0.01696400	-0.00021000

A···2b (Int.1b) E= -3129.097604 Hartree

C	0.59894700	1.19365000	-1.42254900
C	1.84773800	1.76005500	-1.22760900
C	2.06862200	2.51569000	-0.07495800
C	1.04066000	2.69207300	0.85272800
C	-0.18624700	2.09811400	0.60732700
N	-0.36948100	1.38706000	-0.51461500
H	3.04264500	2.95865100	0.10380600
H	0.34418700	0.57331500	-2.27137200
H	2.63217400	1.59469000	-1.95498900
H	1.18954200	3.26554100	1.75919000
H	-1.03571300	2.14143800	1.27720500
Br	-2.90481200	-0.27969900	-0.16481200
H	-1.30541700	0.85363400	-0.59048400
C	1.47614100	-2.19630500	-1.10841800
C	0.39024300	-1.83854300	-0.30935100
C	0.61383400	-1.21515100	0.92961600
C	1.92844200	-0.97029900	1.35283300
C	3.00150200	-1.33451600	0.54370400
C	2.78569300	-1.94541500	-0.69482100
H	1.29109100	-2.67718800	-2.06522000

H	-0.63002300	-1.99941300	-0.64214500
H	2.08421500	-0.48885800	2.31287000
H	4.01491100	-1.13870100	0.88327700
H	3.62522100	-2.23001600	-1.32129500
O	-0.39211500	-0.81215300	1.74174200
H	-1.25456800	-0.83195400	1.25495600

A···2c (Int.1c) E= -3228.29733 Hartree

C	0.17682800	1.26305300	-1.42539200
C	1.30315700	2.04820700	-1.24206600
C	1.33676300	2.92835100	-0.15932600
C	0.24739400	3.00841000	0.70991100
C	-0.85083600	2.19634900	0.47881600
N	-0.85604500	1.36745900	-0.57535600
H	2.21346400	3.54373900	0.01201200
H	0.07064500	0.53760000	-2.22096600
H	2.13881900	1.95744600	-1.92438100
H	0.25180800	3.67794900	1.56114200
H	-1.72799300	2.15937300	1.11234100
Br	-3.01917900	-0.77455600	-0.27481200
H	-1.67706000	0.66778800	-0.64216100
C	1.68302700	-1.85221300	-0.73890400
C	0.49224500	-1.64499500	-0.04200000
C	0.49168900	-0.89298200	1.14469400
C	1.69875000	-0.36624000	1.62742300
C	2.88846000	-0.57398200	0.93363100
C	2.86108900	-1.31028600	-0.24446400
H	1.69950300	-2.42893000	-1.65769300
H	-0.44756500	-2.02650500	-0.42674800
H	1.68616600	0.21095200	2.54573600
H	3.82919100	-0.17221600	1.29453400
O	-0.63561200	-0.62790100	1.84862300
H	-1.43590700	-0.85164700	1.30909000
F	4.01955800	-1.50412400	-0.92887700

A···2d (Int.1d) E= -3588.570745 Hartree

C	-0.07827200	1.26589500	-1.42772600
C	0.93507200	2.17887900	-1.18651600
C	0.77473800	3.09906900	-0.14906200
C	-0.39082400	3.09038000	0.61924600
C	-1.36796500	2.15034600	0.33639100
N	-1.18734300	1.28535400	-0.67259700
H	1.56057800	3.81533400	0.06543000
H	-0.03292900	0.50301400	-2.19378700
H	1.83481400	2.15570000	-1.78827200
H	-0.53590100	3.78963300	1.43340200

H	-2.28936200	2.03628600	0.89353500
Br	-3.16539500	-1.02773300	-0.38137200
H	-1.92170300	0.49984700	-0.76876300
C	1.61920200	-1.62638600	-0.46909500
C	0.36940000	-1.53831400	0.14118400
C	0.19860100	-0.74556200	1.28857100
C	1.30244500	-0.06614300	1.82482900
C	2.55074900	-0.15429100	1.21677800
C	2.69805500	-0.92764900	0.06630900
H	1.74827600	-2.23354200	-1.35845000
H	-0.48994600	-2.04294700	-0.28727700
H	1.16251200	0.53883200	2.71426400
H	3.40100400	0.37699200	1.62987000
O	-0.99539300	-0.58353000	1.89944500
H	-1.72843600	-0.90125900	1.31042900
Cl	4.27572200	-1.01493100	-0.72677500

A···2e (Int.1e) E= -5702.233525 Hartree

C	-0.48832500	1.20475700	-1.38819200
C	0.48965900	2.14611100	-1.11247700
C	0.22522900	3.12401300	-0.15216400
C	-1.00425100	3.13986300	0.50938400
C	-1.93850500	2.16423300	0.20302200
N	-1.65900800	1.24565400	-0.73353700
H	0.97997700	3.86509300	0.08816700
H	-0.36649800	0.40181200	-2.10242400
H	1.44345600	2.09446500	-1.62227100
H	-1.22995100	3.88287200	1.26417300
H	-2.90115100	2.06280200	0.68848200
Br	-3.57908900	-1.12524200	-0.49604800
H	-2.36504800	0.43594800	-0.84372500
C	1.19169900	-1.46992400	-0.21813300
C	-0.09232100	-1.47497500	0.32376600
C	-0.39495600	-0.66086800	1.42864700
C	0.61876100	0.11839000	2.00601000
C	1.90052000	0.12355800	1.46520800
C	2.17318300	-0.65951600	0.34520700
H	1.41817100	-2.08522100	-1.08182400
H	-0.88115000	-2.06152300	-0.13472200
H	0.37885700	0.73473500	2.86587300
H	2.67605400	0.74185100	1.90291900
O	-1.63450100	-0.57011700	1.95631100
H	-2.30454100	-0.92309400	1.31450500
Br	3.91499600	-0.58453600	-0.45585300

A···2g (Int.1g) E= -3243.49349 Hartree

C	-0.11149900	1.27071000	-1.42200300
C	0.91553700	2.16558600	-1.17087100
C	0.78202200	3.05653900	-0.10447700
C	-0.37064000	3.03788500	0.68233500
C	-1.36268800	2.11720100	0.38767000
N	-1.20813400	1.28132700	-0.64936100
H	1.57884600	3.75811200	0.11827700
H	-0.08528700	0.52888300	-2.20910400
H	1.80474300	2.15014500	-1.78823200
H	-0.49416200	3.71362200	1.51952600
H	-2.27628200	1.99383100	0.95527300
Br	-3.25952600	-0.95710600	-0.33217000
H	-1.95870600	0.51132000	-0.75093600
C	1.52632000	-1.69234400	-0.61939500
C	0.30714200	-1.57153800	0.03832900
C	0.20888200	-0.79702000	1.20753300
C	1.35642800	-0.17109500	1.70118400
C	2.58201600	-0.29568800	1.04309900
C	2.67184300	-1.05326200	-0.12937600
H	1.60848600	-2.28586300	-1.52456400
H	-0.58617500	-2.03824100	-0.36376500
H	1.27844000	0.42380000	2.60542100
H	3.45253800	0.20187200	1.45332100
O	-0.95953900	-0.60828000	1.87474100
H	-1.72023400	-0.89740000	1.31224000
C	5.00680700	-0.63337300	-0.36557200
H	5.79761600	-0.90153700	-1.06868300
H	4.92893800	0.46146300	-0.31795800
H	5.26885600	-1.01450100	0.62998300
O	3.81825200	-1.22643000	-0.86099900

A···2h (Int.1h) E= -3286.075413 Hartree

C	-0.52550400	1.26241000	-1.39082900
C	0.30798900	2.33261000	-1.11168300
C	-0.09621100	3.26707600	-0.15640000
C	-1.32128600	3.11572800	0.49443900
C	-2.11192200	2.02302000	0.17844200
N	-1.69853600	1.14813800	-0.74976000
H	0.54696600	4.10627000	0.08632500
H	-0.28238300	0.47703900	-2.09340800
H	1.26000000	2.41954400	-1.61927300
H	-1.65451500	3.82204500	1.24455100
H	-3.06035200	1.79043500	0.64614900
Br	-3.53039600	-1.27713500	-0.42958500

H	-2.31515500	0.27024700	-0.86428100
C	1.30138900	-1.45277200	-0.20909900
C	0.01445400	-1.45291900	0.33314700
C	-0.29618700	-0.60185100	1.40139600
C	0.70941400	0.22632400	1.91944800
C	1.98319600	0.21111400	1.36432900
C	2.31556100	-0.62252200	0.28215900
H	1.50004800	-2.12337200	-1.03738900
H	-0.76573200	-2.07539100	-0.09239600
H	0.46840400	0.88465200	2.74807200
H	2.73613600	0.87166500	1.78438900
O	-1.53294500	-0.51701600	1.94919500
H	-2.19690600	-0.94008200	1.34870600
C	3.73174800	-0.58389600	-0.30907200
C	4.76415400	-0.90646200	0.79170500
H	5.78094400	-0.88495000	0.38346400
H	4.72122900	-0.18622700	1.61388200
H	4.58656200	-1.90172300	1.21140500
C	4.01236500	0.82683500	-0.86997200
H	5.03192600	0.89126300	-1.26669000
H	3.31988300	1.06425300	-1.68531700
H	3.90394500	1.59739400	-0.10068200
C	3.91411100	-1.59794500	-1.44917400
H	3.74751400	-2.62476100	-1.10883900
H	3.23313300	-1.40082300	-2.28374100
H	4.93589100	-1.53908100	-1.83770600

A···2u (Int.1u) E= -3356.691803 Hartree

C	-0.60403900	1.42249400	-1.47394800
C	0.32373800	2.42258000	-1.23178000
C	0.13596200	3.26152000	-0.13131500
C	-0.97557600	3.08941500	0.69527800
C	-1.87331400	2.07592400	0.40197800
N	-1.66472700	1.28848200	-0.66371400
H	0.85468600	4.04546100	0.08238300
H	-0.52761200	0.71449100	-2.28905100
H	1.17651300	2.53401700	-1.88996100
H	-1.14282600	3.72317400	1.55730200
H	-2.75208500	1.84362900	0.99029200
Br	-3.57283500	-1.08644200	-0.41441500
H	-2.35166800	0.46212100	-0.78106500
C	1.25969500	-1.60882500	-0.43963200
C	-0.01575700	-1.51299500	0.09794800
C	-0.21263600	-0.81535700	1.30485400

C	0.88946900	-0.23627900	1.95576300
C	2.15595600	-0.32384900	1.39630400
C	2.36044500	-0.99855800	0.18140300
H	1.42094100	-2.16231900	-1.35870400
H	-0.87171900	-1.95123800	-0.40430200
H	0.72787900	0.27379200	2.89948400
H	2.99634000	0.11191500	1.92526600
O	-1.42317800	-0.66136800	1.87294200
H	-2.14098300	-0.97427800	1.25984000
C	3.70074700	-1.19230100	-0.43089900
O	4.00652100	-2.19799200	-1.03769900
O	4.64924100	-0.22273600	-0.28566500
C	4.30946700	1.16113800	-0.14183900
H	4.45960600	1.49265800	0.88978300
H	4.98955700	1.71697900	-0.79287900
H	3.27808700	1.36300900	-0.43918300

A···2w (Int.1w) E= -3221.255265 Hartree

C	0.05139800	1.29497100	-1.42724400
C	1.08122200	2.18387500	-1.16610100
C	0.94338000	3.07579800	-0.10087400
C	-0.21764500	3.06425500	0.67440000
C	-1.21231500	2.14957700	0.37069600
N	-1.05198000	1.30973800	-0.66319500
H	1.74217400	3.77286700	0.12844200
H	0.07847000	0.55596500	-2.21741400
H	1.97570500	2.16540200	-1.77600100
H	-0.34646600	3.74269000	1.50861700
H	-2.13309600	2.03622600	0.92905800
Br	-3.08624100	-0.96279700	-0.40117900
H	-1.80235300	0.54395900	-0.77714900
C	1.66520800	-1.69065100	-0.51978200
C	0.41923300	-1.58752400	0.08034400
C	0.26770100	-0.83148100	1.25927800
C	1.38974900	-0.20696200	1.83284800
C	2.63223400	-0.31128400	1.23062500
H	1.77975100	-2.26611100	-1.43241200
H	-0.45480500	-2.04690000	-0.36803600
H	1.25692200	0.36580900	2.74402900
H	3.49417100	0.18005100	1.66920800
O	-0.91792900	-0.65396800	1.86364000
H	-1.66329600	-0.93235000	1.26329700
C	2.78383100	-1.04815400	0.04046900
C	4.05618700	-1.12287400	-0.60151500

N	5.09106600	-1.16762900	-1.13371100

J···2b-*d* (Int.1b-*d*) E = -3129.943216 Hartree

C	0.59970000	1.19387200	-1.42266500
C	1.84842400	1.76035200	-1.22745500
C	2.06911800	2.51576700	-0.07461800
C	1.04105100	2.69184500	0.85301200
C	-0.18575000	2.09778900	0.60736600
N	-0.36879800	1.38692900	-0.51473000
H	3.04307100	2.95880400	0.10434500
H	0.34512600	0.57379000	-2.27175200
H	2.63298100	1.59525100	-1.95477300
H	1.18979600	3.26511200	1.75962600
H	-1.03530000	2.14091200	1.27715200
Br	-2.90461800	-0.27908800	-0.16489600
H(Iso=2)	-1.30478500	0.85361300	-0.59071500
C	1.47513200	-2.19730000	-1.10817000
C	0.38942300	-1.83911600	-0.30903400
C	0.61334500	-1.21532600	0.92966800
C	1.92805500	-0.97034000	1.35248100
C	3.00092400	-1.33494300	0.54326700
C	2.78479400	-1.94632300	-0.69495800
H	1.28985900	-2.67856800	-2.06473300
H	-0.63094900	-2.00011800	-0.64149700
H	2.08405500	-0.48853300	2.31229900
H	4.01441800	-1.13901800	0.88252300
H	3.62417100	-2.23123200	-1.32149400
O	-0.39245400	-0.81204700	1.74188000
H(Iso=2)	-1.25488900	-0.83171000	1.25507900

J···2c-*d* (Int.1c-*d*) E = -3229.176757 Hartree

C	0.17695200	1.26305400	-1.42529500
C	1.30315400	2.04838000	-1.24191600
C	1.33654600	2.92864300	-0.15926500
C	0.24705900	3.00862800	0.70983300
C	-0.85102400	2.19638300	0.47869200
N	-0.85602700	1.36738400	-0.57538700
H	2.21315300	3.54416200	0.01208200
H	0.07100200	0.53754100	-2.22084400
H	2.13887800	1.95765300	-1.92416000
H	0.25124500	3.67824300	1.56100500
H	-1.72823700	2.15942100	1.11214100
Br	-3.01910000	-0.77470100	-0.27460800
H(Iso=2)	-1.67701200	0.66765700	-0.64217100
C	1.68302200	-1.85229500	-0.73896700

C	0.49225400	-1.64499200	-0.04204100
C	0.49169600	-0.89291400	1.14460500
C	1.69881400	-0.36613600	1.62723100
C	2.88849800	-0.57394600	0.93343500
C	2.86108100	-1.31033000	-0.24460600
H	1.69942700	-2.42904800	-1.65773200
H	-0.44750200	-2.02667400	-0.42673600
H	1.68626100	0.21111200	2.54551100
H	3.82923100	-0.17216400	1.29431300
O	-0.63553200	-0.62777500	1.84847600
H(Iso=2)	-1.43587800	-0.85166300	1.30901400
F	4.01956500	-1.50422800	-0.92905100

J···2d-*d* (Int.1d-*d*) E = -3589.434692 Hartree

C	-0.07839200	1.26615500	-1.42762000
C	0.93507300	2.17898100	-1.18627100
C	0.77487200	3.09899400	-0.14862700
C	-0.39066400	3.09029400	0.61974300
C	-1.36794200	2.15043200	0.33674200
N	-1.18746400	1.28565300	-0.67246500
H	1.56081300	3.81512200	0.06596900
H	-0.03318700	0.50335500	-2.19378100
H	1.83482200	2.15578800	-1.78802500
H	-0.53560200	3.78940000	1.43405700
H	-2.28930300	2.03627800	0.89393400
Br	-3.16545100	-1.02746800	-0.38153500
H(Iso=2)	-1.92178300	0.50022400	-0.76869800
C	1.61923100	-1.62620000	-0.46954400
C	0.36944000	-1.53846500	0.14083100
C	0.19863200	-0.74625400	1.28861500
C	1.30246000	-0.06695400	1.82508400
C	2.55075200	-0.15474400	1.21693200
C	2.69807000	-0.92763000	0.06613700
H	1.74828800	-2.23295200	-1.35917900
H	-0.48992200	-2.04286200	-0.28789700
H	1.16252500	0.53767600	2.71475900
H	3.40098700	0.37643200	1.63021000
O	-0.99537600	-0.58451100	1.89954800
H(Iso=2)	-1.72837500	-0.90195500	1.31033200
Cl	4.27573900	-1.01448600	-0.72701100

J···2e-*d* (Int.1e-*d*) E = -5703.12572 Hartree

C	-0.48896300	1.20470300	-1.38899200
C	0.48953800	2.14549300	-1.11321100

C	0.22589900	3.12311000	-0.15238600
C	-1.00335700	3.13926600	0.50955400
C	-1.93820500	2.16425300	0.20302600
N	-1.65943600	1.24591300	-0.73397900
H	0.98113000	3.86364800	0.08809800
H	-0.36762000	0.40182700	-2.10341800
H	1.44304000	2.09379200	-1.62355100
H	-1.22839900	3.88196500	1.26484400
H	-2.90067800	2.06299100	0.68886300
Br	-3.57924100	-1.12512700	-0.49619500
H(Iso=2)	-2.36563500	0.43638700	-0.84400700
C	1.19170100	-1.46950100	-0.21822000
C	-0.09237300	-1.47458400	0.32352800
C	-0.39520100	-0.66055600	1.42843200
C	0.61853700	0.11853300	2.00604900
C	1.90039800	0.12362100	1.46547100
C	2.17315400	-0.65928300	0.34538800
H	1.41822300	-2.08464100	-1.08200200
H	-0.88114600	-2.06106700	-0.13511400
H	0.37852800	0.73484700	2.86589700
H	2.67591400	0.74180900	1.90335400
O	-1.63486100	-0.56968300	1.95583200
H(Iso=2)	-2.30480400	-0.92300300	1.31404000
Br	3.91523100	-0.58466800	-0.45516600

J···2g-*d* (Int.1g-*d*) E = -3244.427527 Hartree

C	-0.10916400	1.26923500	-1.42145200
C	0.91811900	2.16381800	-1.17033500
C	0.78422800	3.05580300	-0.10484100
C	-0.36900000	3.03836900	0.68116500
C	-1.36123500	2.11783300	0.38664500
N	-1.20632400	1.28099000	-0.64955500
H	1.58120700	3.75721900	0.11785900
H	-0.08271800	0.52673700	-2.20790000
H	1.80778300	2.14738900	-1.78700600
H	-0.49282900	3.71494500	1.51763400
H	-2.27527800	1.99532500	0.95372100
Br	-3.25969900	-0.95591700	-0.33262400
H(Iso=2)	-1.95720700	0.51128700	-0.75100800
C	1.52472900	-1.69289200	-0.61863000
C	0.30578200	-1.57180000	0.03945700
C	0.20788800	-0.79683100	1.20840000
C	1.35565000	-0.17097800	1.70160700
C	2.58107100	-0.29598100	1.04327100

C	2.67046800	-1.05384100	-0.12906000
H	1.60657800	-2.28656800	-1.52372100
H	-0.58770900	-2.03839700	-0.36236300
H	1.27798600	0.42430400	2.60561800
H	3.45174800	0.20169100	1.45300100
O	-0.96045900	-0.60755500	1.87567900
H(Iso=2)	-1.72124800	-0.89620400	1.31311000
C	5.00547100	-0.63438600	-0.36613300
H	5.79609600	-0.90296600	-1.06928400
H	4.92769900	0.46047500	-0.31888600
H	5.26772100	-1.01516400	0.62951000
O	3.81669200	-1.22744700	-0.86094300

J···2h-*d* (Int.1h-*d*) E = -3287.104968 Hartree

C	-0.52480500	1.26228400	-1.39090300
C	0.30893800	2.33224200	-1.11156900
C	-0.09507500	3.26670600	-0.15619200
C	-1.32025600	3.11560800	0.49451200
C	-2.11113500	2.02312100	0.17833200
N	-1.69790600	1.14822000	-0.74992600
H	0.54830800	4.10570100	0.08667600
H	-0.28181200	0.47696800	-2.09358900
H	1.26090500	2.41907600	-1.61925900
H	-1.65341300	3.82192300	1.24465200
H	-3.05965300	1.79074300	0.64595900
Br	-3.53045600	-1.27659500	-0.42971600
H(Iso=2)	-2.31463800	0.27046200	-0.86439000
C	1.30095200	-1.45298400	-0.20892600
C	0.01401400	-1.45305000	0.33335000
C	-0.29659500	-0.60193100	1.40157000
C	0.70909500	0.22622900	1.91955900
C	1.98286300	0.21092900	1.36443000
C	2.31515900	-0.62275300	0.28224900
H	1.49953000	-2.12359500	-1.03723400
H	-0.76619000	-2.07550200	-0.09219200
H	0.46810000	0.88462200	2.74814200
H	2.73585100	0.87147900	1.78440900
O	-1.53333400	-0.51695600	1.94931400
H(Iso=2)	-2.19730700	-0.93983100	1.34869400
C	3.73129600	-0.58414100	-0.30909000
C	4.76383300	-0.90662700	0.79158200
H	5.78057900	-0.88506600	0.38322500
H	4.72097800	-0.18637800	1.61375200
H	4.58634900	-1.90189000	1.21132900

C	4.01180300	0.82658800	-0.87008400
H	5.03139400	0.89112300	-1.26671500
H	3.31936600	1.06382300	-1.68551800
H	3.90319400	1.59718000	-0.10085500
C	3.91353700	-1.59825800	-1.44913800
H	3.74696400	-2.62504900	-1.10870900
H	3.23246600	-1.40119900	-2.28364900
H	4.93527300	-1.53942300	-1.83779700

J···2u-*d* (Int.1u-*d*) E = -3357.749296 Hartree

C	-0.60519300	1.42229500	-1.47449300
C	0.32333500	2.42177500	-1.23270900
C	0.13663200	3.26075200	-0.13209900
C	-0.97466900	3.08935300	0.69496400
C	-1.87319600	2.07646500	0.40202200
N	-1.66562400	1.28892500	-0.66380700
H	0.85598300	4.04419000	0.08132200
H	-0.52958600	0.71437800	-2.28973800
H	1.17588700	2.53265200	-1.89127200
H	-1.14113400	3.72321500	1.55706400
H	-2.75186600	1.84477200	0.99072200
Br	-3.57333600	-1.08651600	-0.41390600
H(Iso=2)	-2.35282900	0.46274300	-0.78074200
C	1.25950300	-1.60822500	-0.44008100
C	-0.01587600	-1.51222400	0.09758700
C	-0.21253500	-0.81479300	1.30467300
C	0.88976300	-0.23619500	1.95569000
C	2.15620700	-0.32400600	1.39616500
C	2.36047000	-0.99843900	0.18107500
H	1.42060500	-2.16149800	-1.35931000
H	-0.87197200	-1.95023100	-0.40464300
H	0.72836700	0.27370100	2.89953800
H	2.99672800	0.11140700	1.92520600
O	-1.42301100	-0.66059300	1.87280000
H(Iso=2)	-2.14095800	-0.97328500	1.25974500
C	3.70065900	-1.19231700	-0.43137400
O	4.00567900	-2.19722300	-1.03985700
O	4.65006600	-0.22399300	-0.28371500
C	4.31162500	1.16022200	-0.14002500
H	4.46435100	1.49193000	0.89116400
H	4.99060700	1.71540700	-0.79280800
H	3.27972100	1.36269800	-0.43507700

J···2w-*d* (Int.1w-*d*) E = -3222.167978 Hartree

C	0.05089000	1.29522500	-1.42735200
C	1.08046900	2.18445600	-1.16634300
C	0.94248800	3.07630900	-0.10107400
C	-0.21844400	3.06440400	0.67434800
C	-1.21288700	2.14944300	0.37074900
N	-1.05240700	1.30967100	-0.66317900
H	1.74110800	3.77360500	0.12816000
H	0.07807800	0.55624600	-2.21754400
H	1.97488200	2.16625900	-1.77635700
H	-0.34737000	3.74279700	1.50858600
H	-2.13359100	2.03580400	0.92919300
Br	-3.08604900	-0.96331800	-0.40120600
H(Iso=2)	-1.80260300	0.54374100	-0.77711600
C	1.66563900	-1.69043600	-0.51978000
C	0.41961100	-1.58735300	0.08025200
C	0.26798000	-0.83138800	1.25923100
C	1.38996500	-0.20686300	1.83291700
C	2.63250700	-0.31114500	1.23079000
H	1.78026900	-2.26585700	-1.43242500
H	-0.45439900	-2.04667000	-0.36825100
H	1.25706600	0.36585200	2.74412500
H	3.49440900	0.18016300	1.66947500
O	-0.91771200	-0.65400400	1.86354500
H(Iso=2)	-1.66299600	-0.93251500	1.26316100
C	2.78421900	-1.04800100	0.04063900
C	4.05664700	-1.12268600	-0.60122000
N	5.09155700	-1.16725000	-1.13340600

A E= -2822.165744 Hartree

C	-1.16995600	1.11166200	-0.00007000
C	-2.54825900	1.26405700	0.00002200
C	-3.34803300	0.11978100	0.00007200
C	-2.75329000	-1.14346800	0.00003800
C	-1.36882300	-1.22863800	-0.00005800
N	-0.62736800	-0.11297200	-0.00010900
H	-4.42894000	0.21134800	0.00014500
H	-0.46310900	1.93416300	-0.00010700
H	-2.98264000	2.25649500	0.00005400
H	-3.34947100	-2.04803200	0.00008800
H	-0.82341800	-2.16529500	-0.00009200
Br	2.37345400	0.00073500	0.00002400
H	0.49842900	-0.16394900	-0.00019800

J E = -2822.5768622 Hartree

C	-1.16969800	1.11151600	-0.00002200
C	-2.54796800	1.26418100	0.00005200
C	-3.34795900	0.12005900	0.00009700
C	-2.75346500	-1.14330600	0.00006400
C	-1.36901400	-1.22875200	-0.00001100
N	-0.62733700	-0.11324000	-0.00005100
H	-4.42885400	0.21180000	0.00015200
H	-0.46264200	1.93383700	-0.00006200
H	-2.98214900	2.25670500	0.00007300
H	-3.34985000	-2.04773400	0.00009400
H	-0.82380400	-2.16552700	-0.00004300
Br	2.37339400	0.00073900	-0.00002300
H(Iso=2)	0.49847500	-0.16443400	-0.00011900

1b E= -1757.276966 Hartree

C	0.54877300	-2.76109800	-0.41480000
C	0.95549600	-0.46829200	-0.90076000
C	-0.52033500	-0.24967200	-0.55981000
C	-1.35855600	-1.37401300	-1.17999800
C	-0.72462100	-2.68870400	-0.81445200
H	-0.60419500	-0.31512400	0.53505300
H	-1.32617400	-3.58843700	-0.87048600
H	1.03047500	-3.68581800	-0.11284600
H	1.08411600	-0.52270600	-1.99005000
O	1.41159100	-1.71070000	-0.32916200
C	1.84305400	0.63257300	-0.34734100
H	1.46317000	1.59380000	-0.70620600
H	1.76132400	0.62722700	0.74939300
O	3.17778200	0.46450500	-0.78774800
Si	4.46821600	0.19389600	0.25361300
C	4.66461000	1.67475600	1.39992900
H	3.77968600	1.82475200	2.02924900
H	5.52106600	1.53999700	2.07131400
H	4.82792400	2.59751400	0.83209200
C	4.21408600	-1.36015600	1.27925300
H	4.13073200	-2.24473700	0.64073700
H	5.06122400	-1.50898900	1.96016300
H	3.30468000	-1.30754200	1.88606800
C	5.95668900	0.01453400	-0.86954300
H	6.11064700	0.91477900	-1.47414700
H	6.86958000	-0.15747400	-0.28755600
H	5.83363400	-0.83147100	-1.55440800
O	-0.94847900	1.01604100	-1.03202100
Si	-2.03442000	1.92940100	-0.13153200

O	-2.73498900	-1.32856100	-0.80479000
H	-1.35170800	-1.22107400	-2.26726300
Si	-3.40949200	-0.71982800	0.59191700
O	-2.98308500	0.88581600	0.74429400
C	-1.10290100	2.99934600	1.08276000
H	-1.79095100	3.58723200	1.70048000
H	-0.49312400	2.38969600	1.75850900
H	-0.43602100	3.69773100	0.56526700
C	-3.04155000	2.90981500	-1.35259800
H	-2.40483400	3.56150300	-1.96094600
H	-3.58182400	2.24014100	-2.02994600
H	-3.77675500	3.54054000	-0.84094400
C	-2.80778200	-1.62579700	2.11335600
H	-3.22268500	-1.17707300	3.02306000
H	-3.11575200	-2.67688100	2.08676300
H	-1.71657900	-1.60509200	2.19776400
C	-5.25300800	-0.85346400	0.36283200
H	-5.78339600	-0.41725000	1.21646600
H	-5.57227900	-0.32156600	-0.53937700
H	-5.56851100	-1.89800400	0.26826200

3b E= -2064.177662 Hartree

C	1.20397400	-0.85704500	-0.21021200
C	0.00707700	1.22824900	-0.63479200
C	-1.25975300	0.45159500	-0.27308800
C	-1.23699500	-0.93762200	-0.93637500
C	0.19933200	-1.38752100	-1.22285400
H	-1.22352500	0.29763300	0.81503000
H	0.50964100	-1.02158600	-2.20619900
H	0.13326400	1.28862100	-1.72438900
O	1.11074900	0.53864700	-0.02809100
C	0.01129000	2.63895400	-0.06847300
H	-0.89702500	3.14226900	-0.41188400
H	-0.03010800	2.57551400	1.02824600
O	1.11787800	3.39985800	-0.50373900
Si	2.69167900	3.34598700	0.09447300
C	2.71206000	2.74969400	1.87748700
H	2.35544200	1.71892800	1.95293500
H	3.73545700	2.78159100	2.27033700
H	2.09333700	3.37870300	2.52786600
C	3.78311900	2.26953800	-0.98883800
H	4.82513100	2.31026700	-0.64739100
H	3.45226600	1.22835100	-0.96481200
H	3.76476000	2.61328100	-2.02963800

C	3.29284400	5.12509300	0.00228600
H	2.69970700	5.78314000	0.64703900
H	4.34032700	5.20345200	0.31722700
H	3.22592200	5.51349900	-1.02026800
O	-2.43350300	1.16210800	-0.64888300
Si	-3.79227300	1.10902200	0.33604700
O	-1.89126600	-1.89911900	-0.10792300
H	-1.76716800	-0.84372000	-1.89273200
Si	-3.55517600	-1.93252200	0.08731700
O	-4.07399700	-0.48345300	0.72520800
C	-3.47957200	2.04054100	1.92310700
H	-4.35075500	1.98728800	2.58549800
H	-2.62736800	1.62441500	2.47095200
H	-3.27099600	3.09770000	1.72611800
C	-5.18863200	1.80821400	-0.67724300
H	-4.98666000	2.84360900	-0.97192900
H	-5.34004700	1.22136000	-1.58915300
H	-6.12578000	1.79661300	-0.11020300
C	-3.90550200	-3.28307200	1.31661100
H	-3.57485400	-4.25694200	0.94046700
H	-3.38984600	-3.09262400	2.26340500
H	-4.97871400	-3.34911100	1.52560500
C	-4.39002000	-2.19503400	-1.56656500
H	-5.47948000	-2.19478800	-1.44988500
H	-4.13839600	-1.40420400	-2.28132300
H	-4.10048200	-3.15269600	-2.01303200
O	2.53061000	-1.09031100	-0.67928000
C	3.24005300	-2.16063800	-0.20328200
C	2.66072900	-3.30050800	0.36621800
C	4.63069500	-2.07702000	-0.34529100
C	3.48521200	-4.33993700	0.80528300
H	1.58557300	-3.39659600	0.45912000
C	5.43607900	-3.12552500	0.08599400
H	5.05459200	-1.18272100	-0.78952300
C	4.86934100	-4.26267000	0.66939100
H	3.02992700	-5.22060500	1.24868300
H	6.51352700	-3.05051500	-0.02723900
H	5.50033400	-5.07755700	1.00920200
H	0.24092000	-2.47817100	-1.25323200
H	1.09010300	-1.33277200	0.77085800

TS1 E= -4886.35942 Hartree

C	1.82600900	-1.14190900	-2.05673000
C	2.93342600	-0.34222900	-2.30747500

C	2.73746400	0.92882300	-2.83709700
C	1.44215800	1.37689600	-3.12215400
C	0.37575100	0.53985700	-2.85740400
N	0.59901900	-0.68302000	-2.33985600
H	3.58551500	1.57822000	-3.02417000
H	1.88305900	-2.14175300	-1.64774500
H	3.91976500	-0.71479100	-2.06218400
H	1.26659400	2.36623600	-3.52525300
H	-0.66051000	0.80569700	-3.01320100
Br	-1.65767900	-2.54123400	-1.41942600
H	-0.23813900	-1.31276400	-2.07598400
C	2.46570500	-4.85258900	0.39979800
C	1.84870700	-3.60391800	0.37912700
C	2.55013600	-2.43612100	0.79158600
C	3.89667100	-2.59994600	1.21143000
C	4.49228800	-3.85756200	1.23285100
C	3.78822800	-4.99732600	0.82870500
H	1.90270600	-5.72529800	0.07659200
H	0.82414000	-3.48898100	0.02865000
H	4.44792600	-1.71746300	1.52472400
H	5.52339000	-3.95182300	1.56722600
H	4.26168600	-5.97421600	0.84500800
O	1.96595000	-1.26000100	0.75829700
H	0.35576900	-1.31881600	1.25878800
C	0.71115500	0.11646100	2.46836100
C	0.33326100	1.44708000	0.55089600
C	-1.17409900	1.23242900	0.69792900
C	-1.50387100	-0.16129400	1.27658400
C	-0.31716400	-0.77883600	2.02316200
H	-1.56213300	1.98325000	1.40168300
H	-0.61257100	-1.49948300	2.78378700
H	1.33443200	-0.06361700	3.34137500
H	0.77468200	0.69719300	-0.10520200
O	0.98598800	1.21549100	1.86173800
C	0.71152800	2.85107800	0.11893200
H	0.04882900	3.11204700	-0.71029400
H	0.49821900	3.54317900	0.94618400
O	2.03972700	2.96651900	-0.33218400
Si	3.44667900	3.01108400	0.60128400
C	3.08918800	3.79685100	2.27293300
H	2.43702300	3.17825100	2.89613000
H	4.02869400	3.93921700	2.82035100
H	2.62336500	4.78315600	2.16634700
C	4.17144800	1.28996200	0.79113100

H	4.82305500	1.24660300	1.67297700
H	3.40454000	0.51391500	0.89346900
H	4.78783700	1.02947400	-0.07670900
C	4.62102600	4.10102700	-0.37692800
H	4.24486800	5.12637200	-0.46105000
H	5.60472900	4.14310300	0.10554200
H	4.77094500	3.71663800	-1.39217400
O	-1.71993600	1.40682400	-0.59594100
Si	-3.36032200	1.50977900	-0.97945400
O	-2.61440500	-0.07090500	2.16546200
H	-1.71661900	-0.83041900	0.43474200
Si	-4.17340900	-0.17559500	1.54642400
O	-4.25340100	0.86606700	0.25162000
C	-3.81589700	3.31538000	-1.12724600
H	-4.87773600	3.43361600	-1.37014200
H	-3.62978800	3.84553500	-0.18659000
H	-3.23550000	3.81042000	-1.91384200
C	-3.57412400	0.54312900	-2.55364300
H	-3.08236200	1.02885800	-3.40500300
H	-3.14576700	-0.45831200	-2.42365200
H	-4.63514900	0.43797300	-2.80593900
C	-5.29868400	0.44767000	2.89387600
H	-5.22571200	-0.17955800	3.78898500
H	-5.04526500	1.47425700	3.17772900
H	-6.34314800	0.43689400	2.56350000
C	-4.56975300	-1.90602300	0.98822100
H	-5.56147300	-1.94266900	0.52273100
H	-3.83854900	-2.26581100	0.25564500
H	-4.57170500	-2.59905300	1.83730300

TS2 E= -4886.334002 Hartree

C	-2.57125700	3.03213800	-1.39147300
C	-3.84111200	3.57856500	-1.23553600
C	-4.90113900	2.74026200	-0.89635900
C	-4.66535800	1.37472800	-0.73643700
C	-3.37684400	0.89614100	-0.93254600
N	-2.36341600	1.71653200	-1.24229000
H	-5.89827000	3.14450800	-0.75429900
H	-1.68398300	3.62666200	-1.58684700
H	-3.98418900	4.64539000	-1.36458000
H	-5.46125200	0.69068900	-0.46611700
H	-3.13207200	-0.15594600	-0.84862100
Br	0.73704200	3.63245900	-0.71690700
H	-1.14001700	1.10335200	-1.45833700

C	-3.19913900	2.76240100	2.35552500
C	-1.90989600	2.72358600	1.82960100
C	-1.16163700	1.54002200	1.91633400
C	-1.70945800	0.41679700	2.55211500
C	-3.00011500	0.47234100	3.07539700
C	-3.75672000	1.64194600	2.97721100
H	-3.77410600	3.68062400	2.27558800
H	-1.47315900	3.58111000	1.32878700
H	-1.10830400	-0.48364800	2.62924600
H	-3.41529800	-0.40659400	3.56071000
H	-4.76222200	1.68256300	3.38416200
O	0.08353400	1.42966100	1.39842200
H	0.30158300	2.21145300	0.81444400
C	1.02818400	0.76428800	-1.75142300
C	1.85227500	-0.66178300	0.00786000
C	0.82888400	-1.68781000	-0.48375800
C	-0.49208800	-1.00123700	-0.83818700
C	-0.23714000	0.17187200	-1.77772200
H	1.20873400	-2.18728000	-1.38751800
H	-0.61413300	0.03636100	-2.79414100
H	1.32001800	1.52999100	-2.45969100
H	1.51191000	-0.22111000	0.94557000
O	1.99210600	0.45404800	-0.93174300
C	3.24622000	-1.25151700	0.15647500
H	3.14388300	-2.18407200	0.72032000
H	3.62994400	-1.50325800	-0.84357600
O	4.12342600	-0.41166100	0.86081100
Si	5.00162000	0.90948000	0.28226900
C	5.27373200	0.75896700	-1.57451100
H	4.33740800	0.82700100	-2.13550200
H	5.92082700	1.57599200	-1.91564800
H	5.76889600	-0.18129300	-1.84335800
C	4.15622800	2.51641700	0.73207500
H	3.21985200	2.69364400	0.19308900
H	3.92879500	2.54130200	1.80444200
H	4.82303400	3.36284800	0.52282600
C	6.65279900	0.79842000	1.17281600
H	7.18591900	-0.12482700	0.91977900
H	7.29984900	1.64287300	0.90735000
H	6.51617000	0.81682500	2.25988100
O	0.64872200	-2.60681800	0.58324600
Si	0.35860000	-4.24770600	0.37356300
O	-1.40068300	-1.88483800	-1.48506900
H	-0.92339700	-0.64147900	0.10301400

Si	-2.18324000	-3.19035900	-0.78354700
O	-1.07414700	-4.39245000	-0.46259600
C	1.71585100	-5.03687800	-0.63462800
H	1.50216400	-6.09867000	-0.80034500
H	1.81495500	-4.56851700	-1.61952500
H	2.68294800	-4.96620400	-0.12520200
C	0.19159000	-4.93988100	2.09188300
H	1.12053100	-4.82399200	2.66002100
H	-0.60524700	-4.42639100	2.63978800
H	-0.05500400	-6.00666600	2.06063300
C	-3.36704200	-3.81581400	-2.07458900
H	-4.12310200	-3.06251700	-2.32025400
H	-2.83640800	-4.07559400	-2.99619700
H	-3.88709200	-4.71267600	-1.72122400
C	-3.01002200	-2.67609800	0.81294000
H	-3.47899400	-3.54349200	1.29100700
H	-2.28710900	-2.25852500	1.51858100
H	-3.78877100	-1.92280200	0.65460000

Int.Oc1 E = -1757.299864 Hartree

C	0.64182000	-2.71286100	-0.36724700
C	0.95935500	-0.46136000	-0.89299700
C	-0.51606900	-0.23823800	-0.55320500
C	-1.35590700	-1.36646500	-1.16407900
C	-0.78448800	-2.67557800	-0.80749600
H	-0.59955700	-0.29486500	0.54217900
H	-0.87910500	-3.31537700	-1.65990700
H	1.12252600	-3.63573100	-0.05813000
H	1.08746000	-0.52460500	-1.98187000
O	1.38348900	-1.73998700	-0.31391000
C	1.84847500	0.64280000	-0.34872300
H	1.46960800	1.60159800	-0.71508100
H	1.76719100	0.64628900	0.74805200
O	3.18281600	0.46960700	-0.78831400
Si	4.47335100	0.20573500	0.25465000
C	4.67201200	1.69543900	1.38905300
H	3.78753000	1.85151900	2.01751500
H	5.52858000	1.56499300	2.06114500
H	4.83620900	2.61344600	0.81381800
C	4.21776100	-1.33978800	1.29273900
H	4.13307200	-2.22932500	0.66132400
H	5.06499900	-1.48422100	1.97447200
H	3.30867000	-1.28123700	1.89948200
C	5.96114200	0.01562400	-0.86764100

H	6.11594100	0.91083600	-1.47946000
H	6.87406400	-0.15285200	-0.28467000
H	5.83678000	-0.83566000	-1.54569500
O	-0.94287400	1.02419200	-1.03531100
Si	-2.02733600	1.94601500	-0.14169000
O	-2.73212900	-1.31635300	-0.78869000
H	-1.34932200	-1.22220000	-2.25253000
Si	-3.40531800	-0.69569500	0.60339400
O	-2.97690300	0.91059300	0.74280400
C	-1.09402100	3.02446600	1.06366500
H	-1.78110300	3.61809000	1.67695900
H	-0.48470400	2.41947800	1.74400400
H	-0.42650900	3.71789600	0.54035600
C	-3.03378100	2.91789400	-1.37012200
H	-2.39652700	3.56394100	-1.98389800
H	-3.57514500	2.24350200	-2.04189600
H	-3.76801000	3.55356700	-0.86321200
C	-2.80407900	-1.59024600	2.13176000
H	-3.21806300	-1.13378600	3.03802700
H	-3.11333300	-2.64113400	2.11366500
H	-1.71281700	-1.57019500	2.21556100
C	-5.24908900	-0.82891200	0.37612400
H	-5.77859600	-0.38526800	1.22646900
H	-5.56808700	-0.30383000	-0.53016500
H	-5.56589600	-1.87378800	0.29000500
H	-1.37958800	-3.08690400	-0.01910100

Int.Oc2 E = -4329.207397 Hartree

Br	1.36212400	-2.85201900	-0.71914900
H	-0.71803400	-2.88233400	1.41172300
C	1.11028900	-1.79935400	1.02993400
C	0.88392100	0.34604300	-0.00101700
C	-0.59844500	0.41923300	0.39038900
C	-1.20529400	-0.98472800	0.48888300
C	-0.35623700	-1.85267600	1.41159400
H	-0.68429300	0.89701900	1.37731700
H	-0.43758200	-1.46366000	2.43451700
H	1.75989400	-2.33645700	1.71728400
H	0.97063200	-0.05631000	-1.01702100
O	1.60172800	-0.52726300	0.89500800
C	1.56727300	1.70233700	0.07096800
H	0.92750400	2.42387200	-0.44499100
H	1.63415900	2.00219500	1.12696100
O	2.83010900	1.72412300	-0.55570600

Si	4.28461800	1.11921400	0.03937400
C	4.28194900	1.09288500	1.92035900
H	3.52974000	0.40601700	2.31717300
H	5.26265200	0.76799900	2.28838000
H	4.09198100	2.08808900	2.33875500
C	4.63110500	-0.58163900	-0.67711300
H	3.79348200	-1.27041700	-0.54063100
H	4.82517000	-0.51174400	-1.75380300
H	5.51922500	-1.02711700	-0.21210500
C	5.59275300	2.31951000	-0.57489500
H	5.45630700	3.31780900	-0.14454600
H	6.59871900	1.97445800	-0.30733700
H	5.55721000	2.41909700	-1.66550300
O	-1.25836900	1.18664100	-0.60464400
Si	-2.64719200	2.09414700	-0.35622300
O	-2.52370600	-0.93422500	1.01615200
H	-1.20878300	-1.42111300	-0.51756200
Si	-3.89602800	-0.58467900	0.11966100
O	-3.93477200	1.05377600	-0.18568600
C	-2.48398100	3.11637100	1.19958500
H	-3.36460600	3.75591100	1.32649600
H	-2.41125300	2.48548700	2.09158100
H	-1.60197900	3.76556700	1.16710000
C	-2.86851000	3.10858400	-1.89966800
H	-2.05394600	3.83041600	-2.02167700
H	-2.88568100	2.46600600	-2.78603000
H	-3.81187600	3.66434000	-1.86920600
C	-5.34400000	-1.02127900	1.20342900
H	-5.38278500	-2.09819500	1.39864200
H	-5.28041500	-0.50452700	2.16660600
H	-6.28670100	-0.72972200	0.72793000
C	-3.87007600	-1.50956600	-1.50385800
H	-4.79533200	-1.32232900	-2.06026100
H	-3.03788000	-1.18873400	-2.13874300
H	-3.78381100	-2.59123500	-1.35270900

Pyridine E = -248.7488502

C	0.66919300	1.18633700	0.00012700
C	-0.71326500	1.21178800	-0.00007500
C	-1.41404800	0.00299400	0.00026600
C	-0.71879100	-1.20855000	-0.00003300
C	0.66406900	-1.18913300	-0.00003700
N	1.30660900	-0.00290000	0.00014900
H	-2.49929600	0.00561500	-0.00000600

H	1.29177700	2.07184100	-0.00029500
H	-1.22865700	2.16391800	-0.00094200
H	-1.23783600	-2.15870200	-0.00056100
H	1.28186900	-2.07796100	-0.00027600
H	2.32293400	-0.00502700	-0.00045800

2c^{-} E = -306.8235683

C	0.28690000	-1.21239300	0.00005900
C	1.08152200	0.00000700	0.00060800
C	0.28690500	1.21239400	0.00005800
C	-1.10119600	1.20071100	-0.00005000
C	-1.82846800	0.00000200	-0.00002200
C	-1.10119300	-1.20071600	-0.00004700
H	0.82901500	-2.15738700	-0.00022300
H	0.82900000	2.15740100	-0.00021500
H	-1.63871400	2.15061000	-0.00022700
H	-2.91554100	-0.00000300	-0.00019100
H	-1.63871900	-2.15061000	-0.00023300
O	2.34851700	-0.00000500	-0.00031800

Reference

[1] K. Bano, A. Jain, R. Sarkar, T. K. Panda, *ChemistrySelect* **2020**, *5*, 4470-4477.

[2] O. O. Kolodyazhnaya, O. I. Kolodyazhnyi, *Russian Journal of General Chemistry* **2011**, *81*, 307-314.

[3] H.-J. Tien, M.-Y. Yeh, J. M. Tien, *Journal of the Chinese Chemical Society* **1977**, *24*, 115-121.

[4] B. Febriansyah, C. S. D. Neo, D. Giovanni, S. Srivastava, Y. Lekina, T. M. Koh, Y. Li, Z. X. Shen, M. Asta, T. C. Sum, N. Mathews, J. England, *Chemistry of Materials* **2020**, *32*, 4431-4441.

[5] X.-K. Cui, M. Zhong, X.-B. Meng, Z.-J. Li, *Carbohydrate Research* **2012**, *358*, 19-22.

[6] J. Zhang, Y. Dong, M. Yuma, Y. Mei, N. Jiang, G. Yang, Z. Wang, *Synlett* **2020**, *31*, 1087-1093.

[7] M. Kumar, A. Gurawa, N. Kumar, S. Kashyap, *Organic Letters* **2022**, *24*, 575-580.

[8] C. Hansch, A. Leo, R. W. Taft, *Chemical Reviews* **1991**, *91*, 165-195.

[9] T. Liu, M. Guo, A. Orthaber, R. Lomoth, M. Lundberg, S. Ott, L. Hammarström, *Nature Chemistry* **2018**, *10*, 881-887.

[10] E. Tyrode, J. Hedberg, *the Journal of Physical Chemistry C* **2012**, *116*, 1080-1091.

[11] Y. Rezus, D. Madsen, H. Bakker, *The Journal of chemical physics* **2004**, *121*, 10599-10604.

[12] A. Fujii, T. Ebata, N. Mikami, *the Journal of Physical Chemistry A* **2002**, *106*, 8554-8560.

[13] M. Frisch, G. Trucks, H. Schlegel, G. Scuseria, M. Robb, J. Cheeseman, G. Scalmani, V. Barone, G. Petersson, H. Nakatsuji, Gaussian 16, Revision A. 03, Gaussian, Inc., Wallingford CT. *Gaussian16 (Revision A. 03)* **2016**.

[14] A. D. Becke, *the Journal of chemical physics* **1992,** *96*, 2155-2160.

NMR spectra

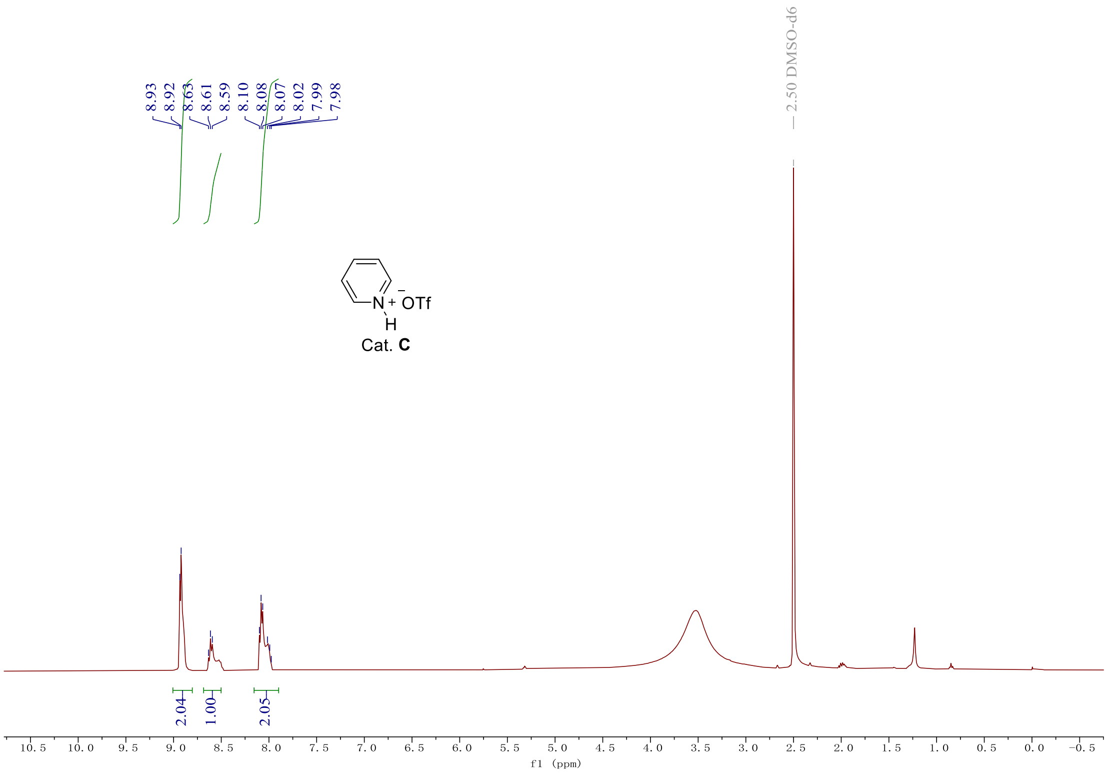

Figure S12. ^{1}H NMR (400 MHz, DMSO-d_6) Spectra for compound Cat. C

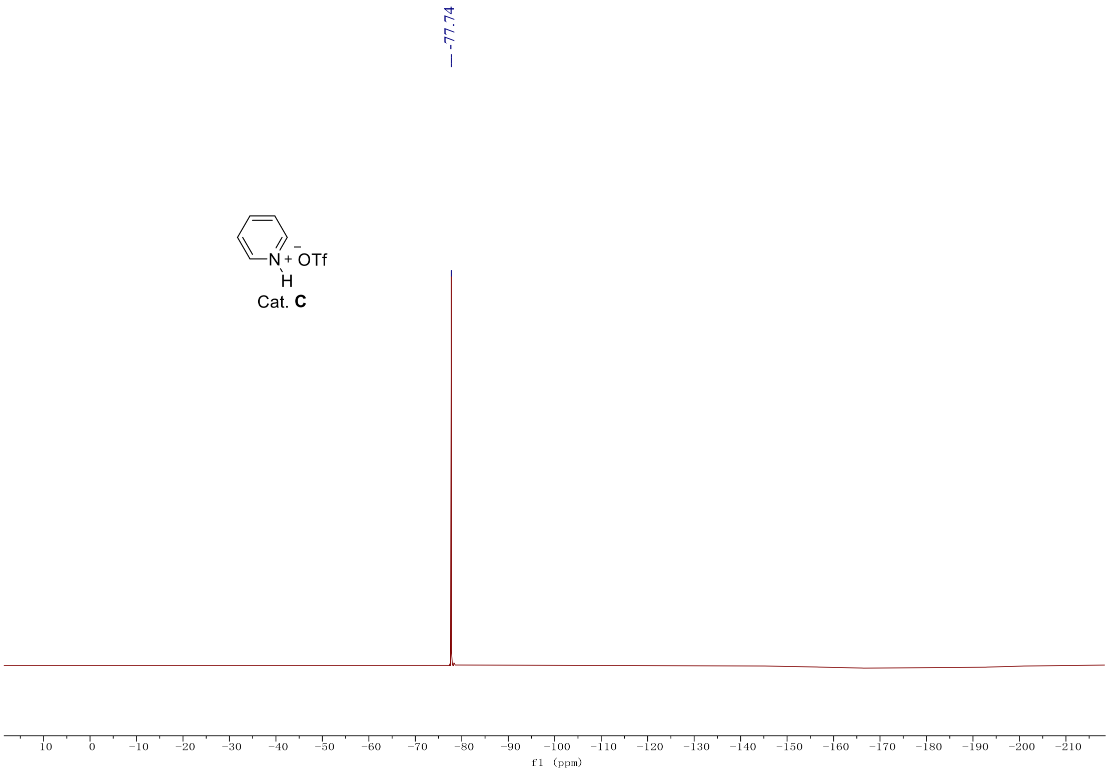

Figure S13. ^{19}F NMR (376 MHz, DMSO-d_6) Spectra for compound Cat. C

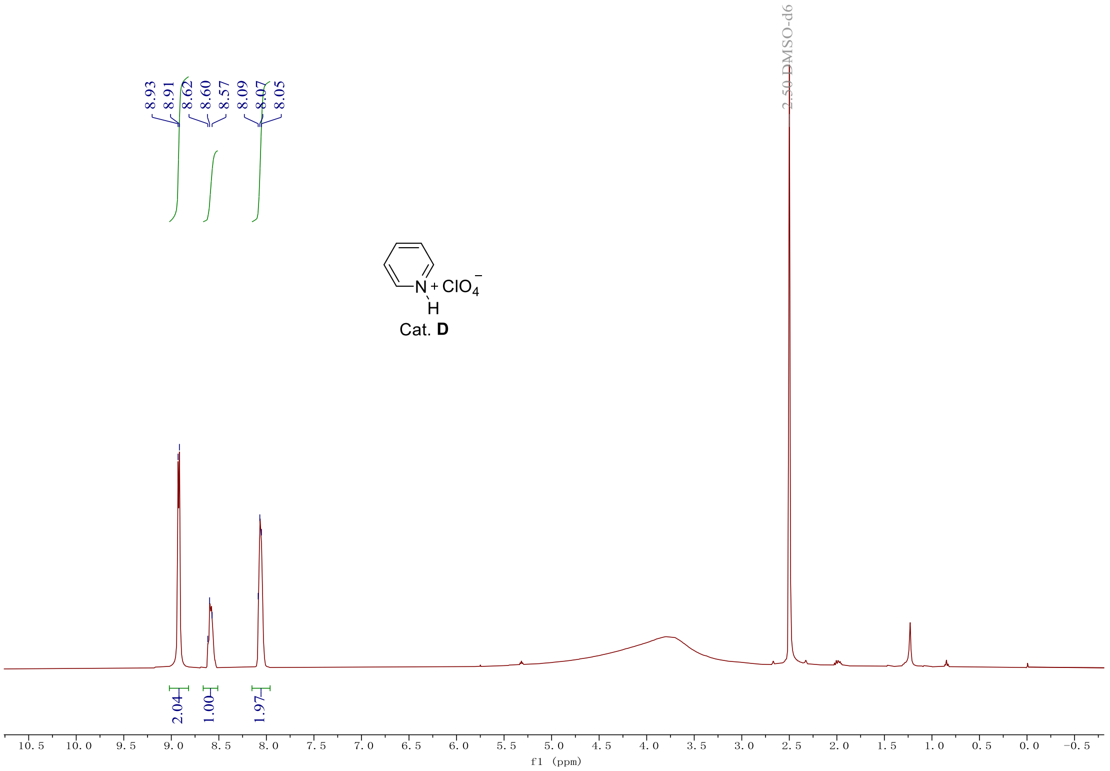

Figure S14. **^{1}H NMR (400 MHz, DMSO-d_6) Spectra for compound Cat. D**

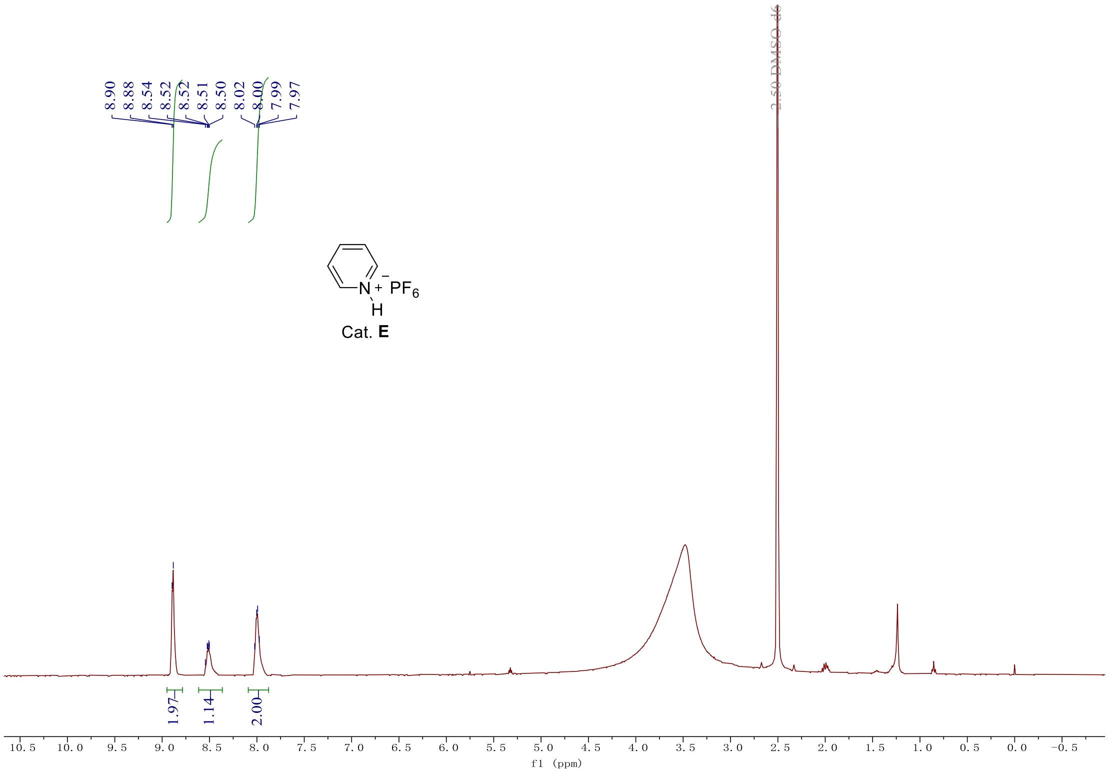

Figure S15. **^{1}H NMR (400 MHz, DMSO-d_6) Spectra for compound Cat. E**

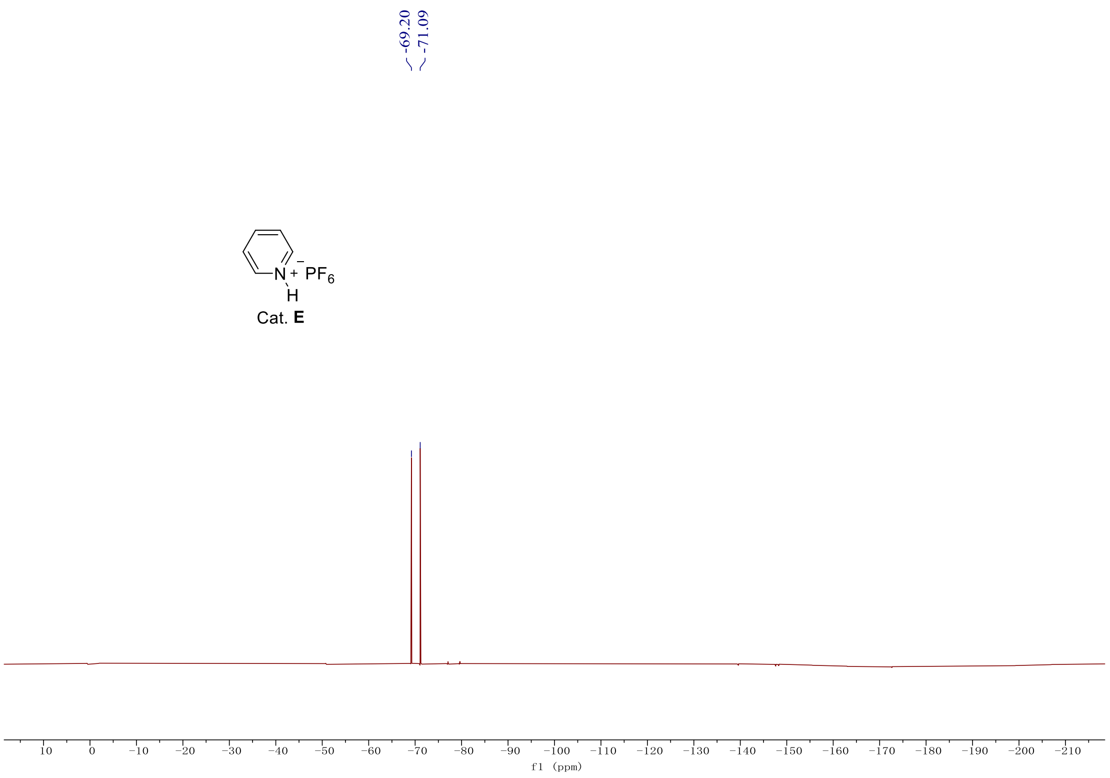

Figure S16. **^{19}F NMR (376 MHz, DMSO-*d*6) Spectra for compound Cat. E**

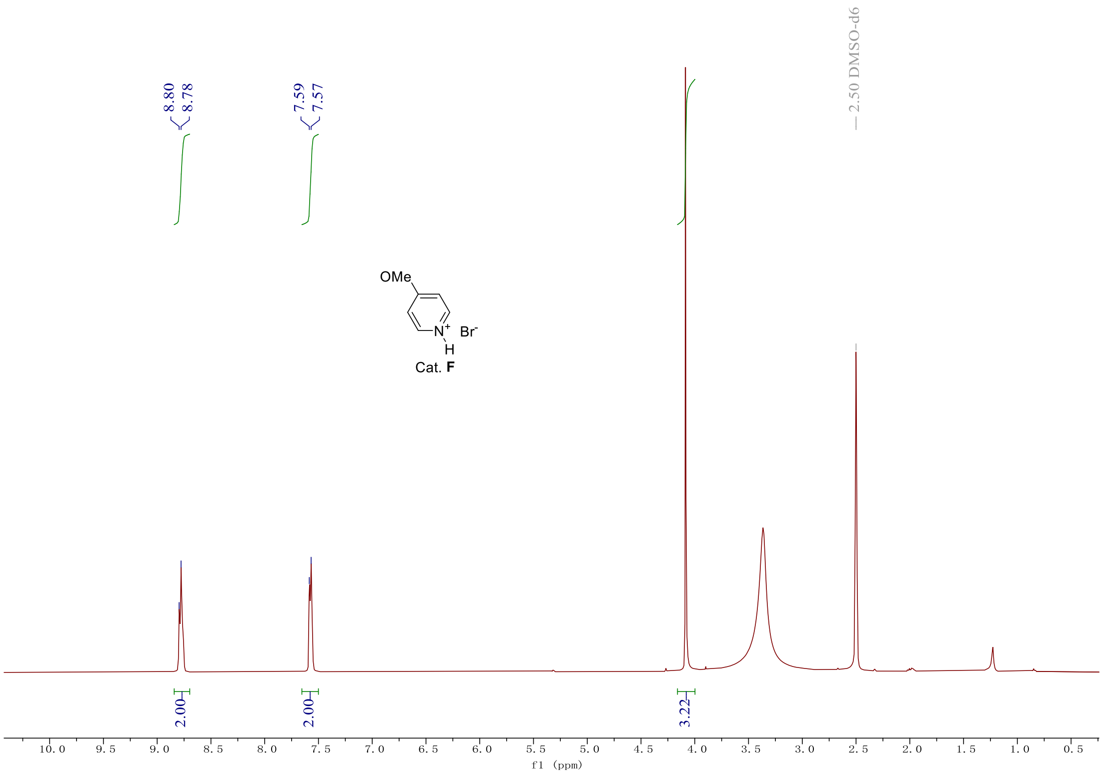

Figure S17. **^{1}H NMR (400 MHz, DMSO-*d*6) Spectra for compound Cat. F**

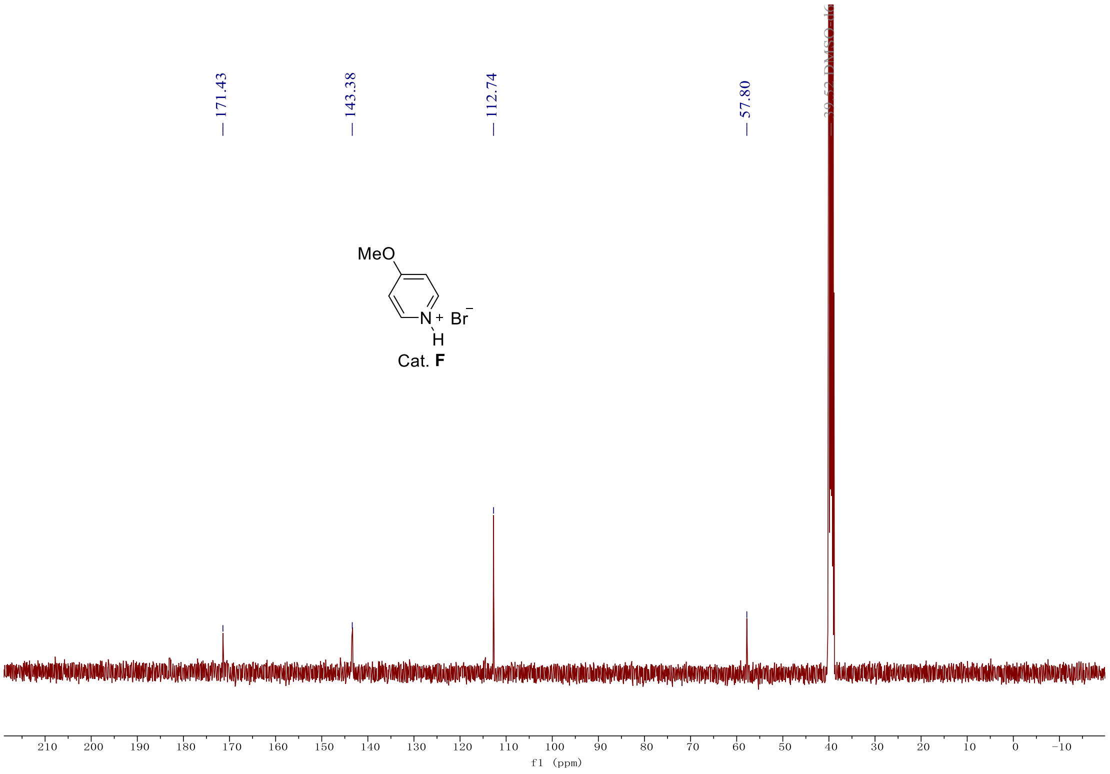

Figure S18. **^{13}C NMR (101 MHz, DMSO-*d*$_6$) Spectra for compound Cat. F**

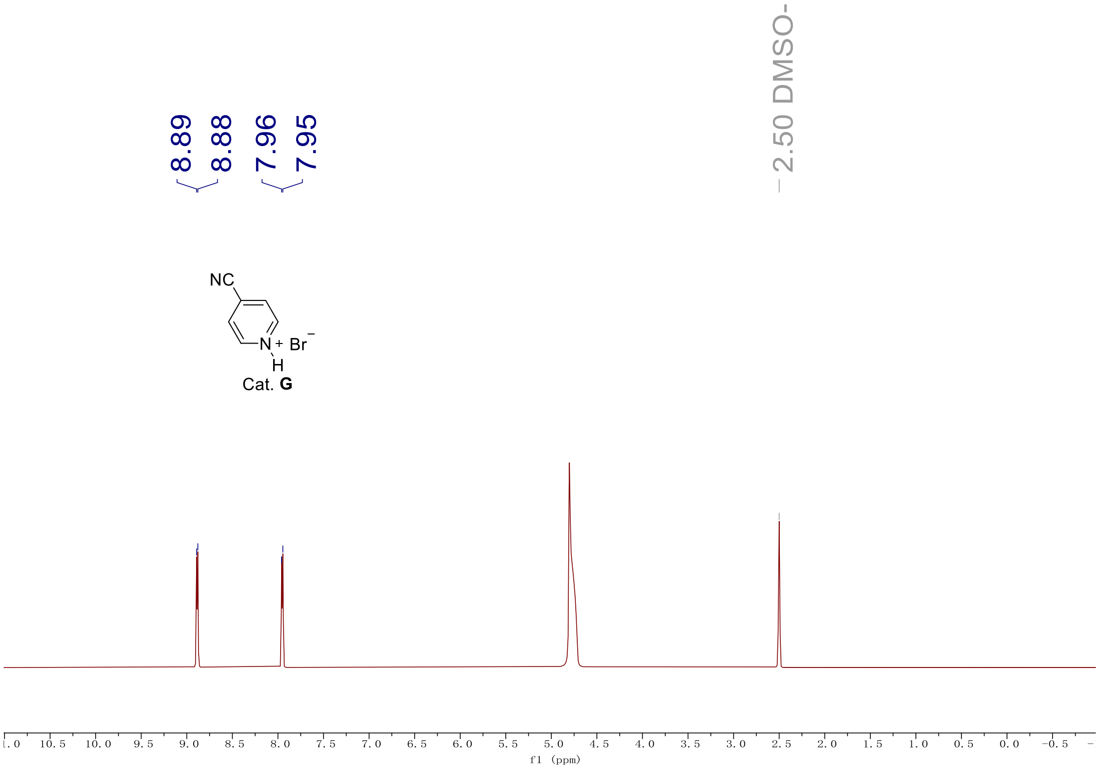

Figure S19. **^{1}H NMR (400 MHz, DMSO-*d*$_6$) Spectra for compound Cat. G**

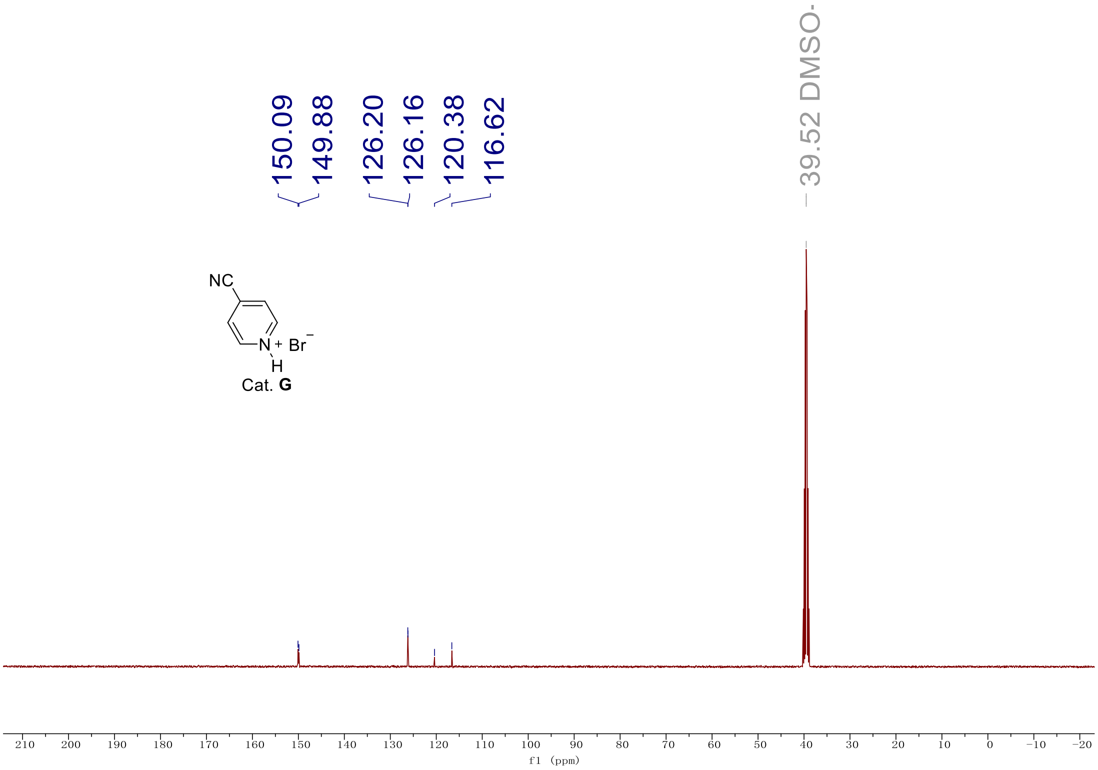

Figure S20. **^{13}C NMR (101 MHz, DMSO-d_6) Spectra for compound Cat. G**

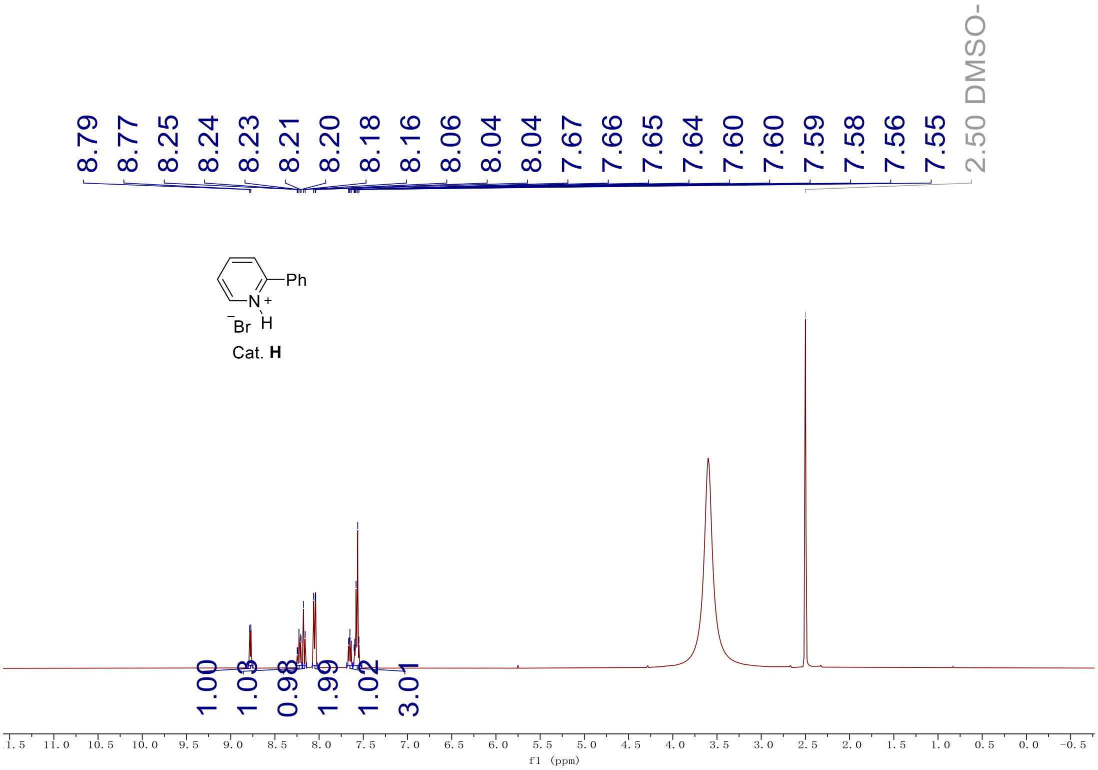

Figure S21. **^{1}H NMR (400 MHz, DMSO-d_6) Spectra for compound Cat. H**

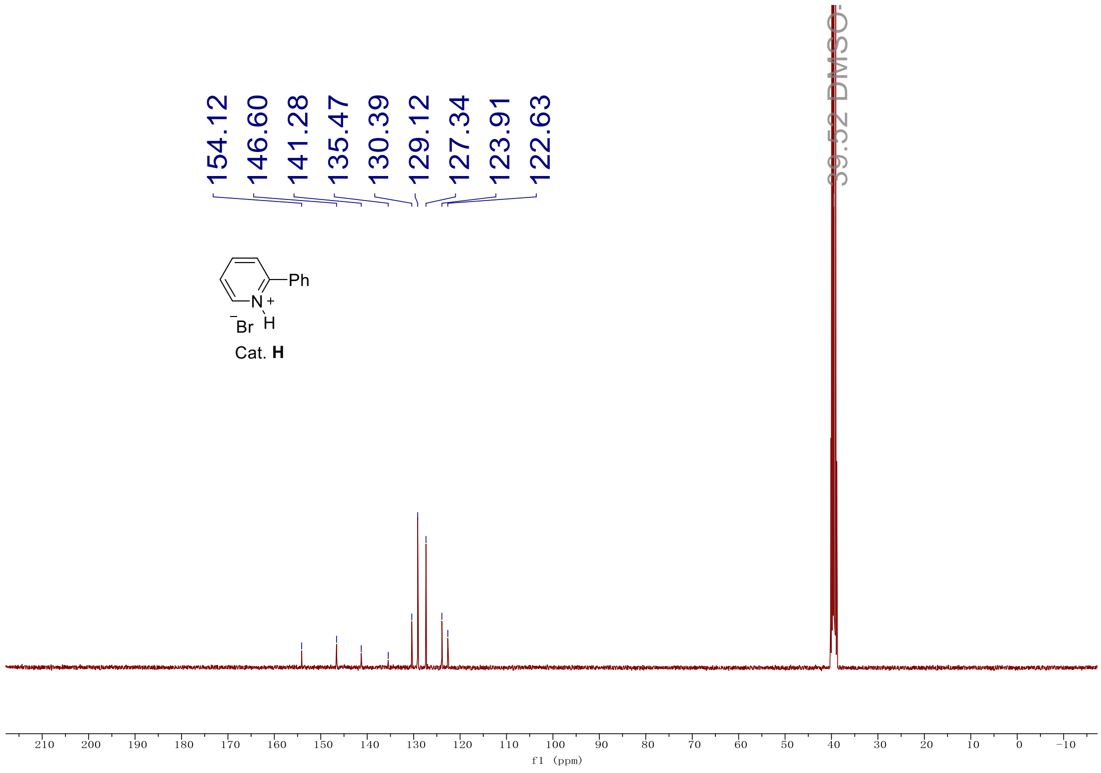

Figure S22. ^{13}C NMR (101 MHz, DMSO-*d*$_6$) Spectra for compound Cat. H

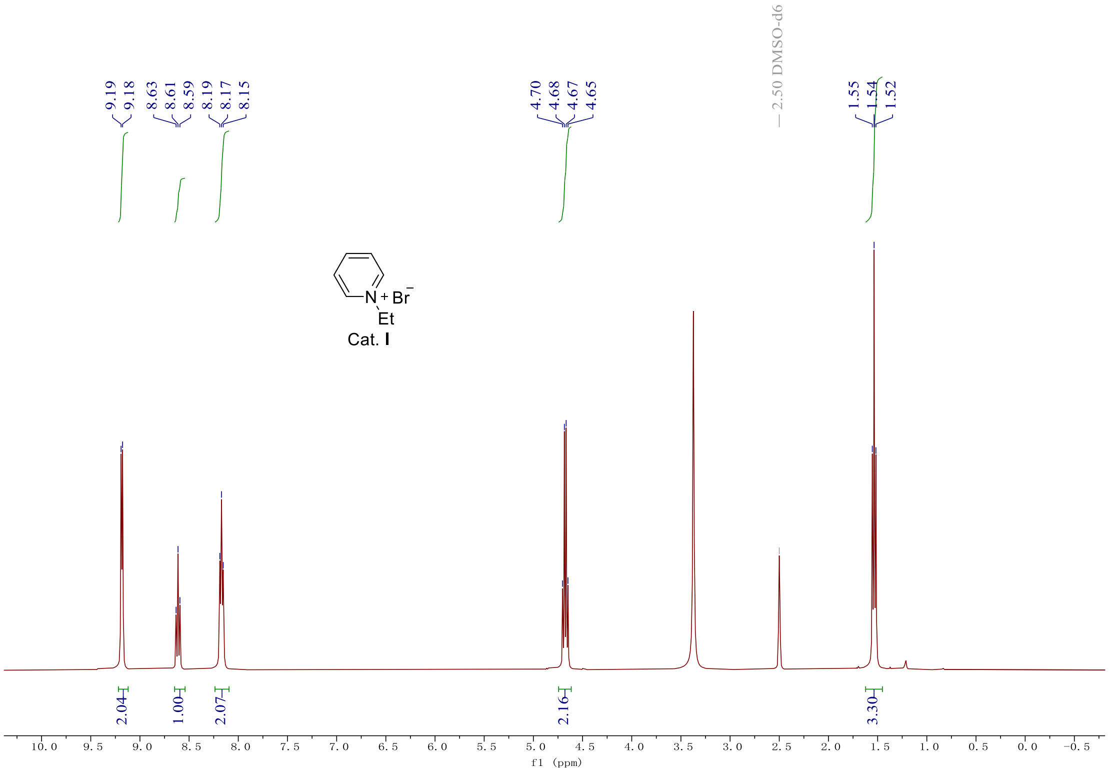

Figure S23. ^{1}H NMR (400 MHz, DMSO-*d*$_6$) Spectra for compound Cat. I

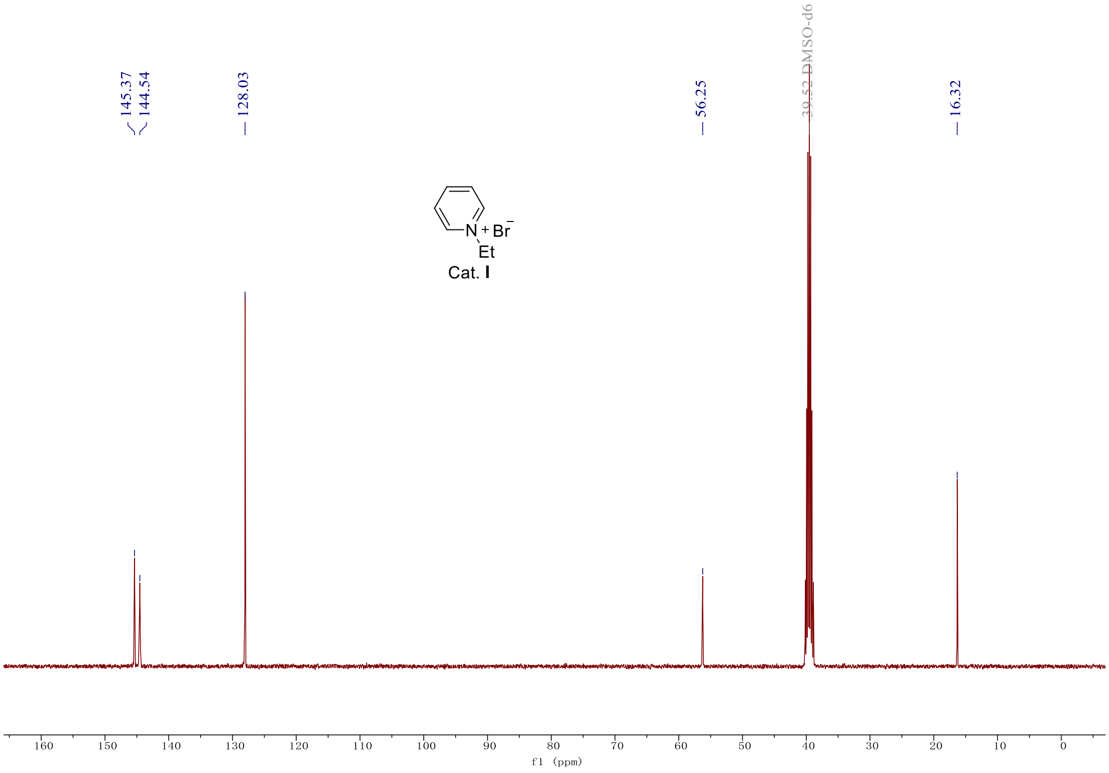

Figure S24. ^{13}**C NMR (101 MHz, DMSO-**d_6**) Spectra for compound Cat. I**

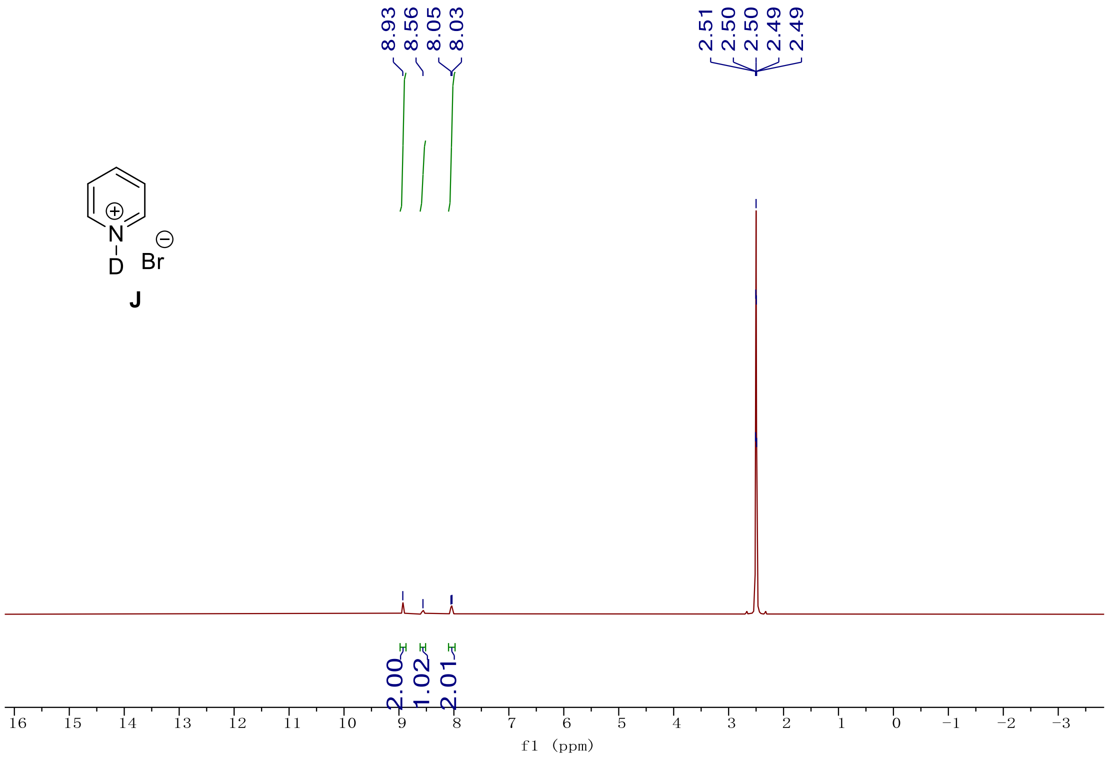

Figure S25. ^{1}**H NMR (400 MHz, DMSO-**d_6**) Spectra for compound Cat. J**

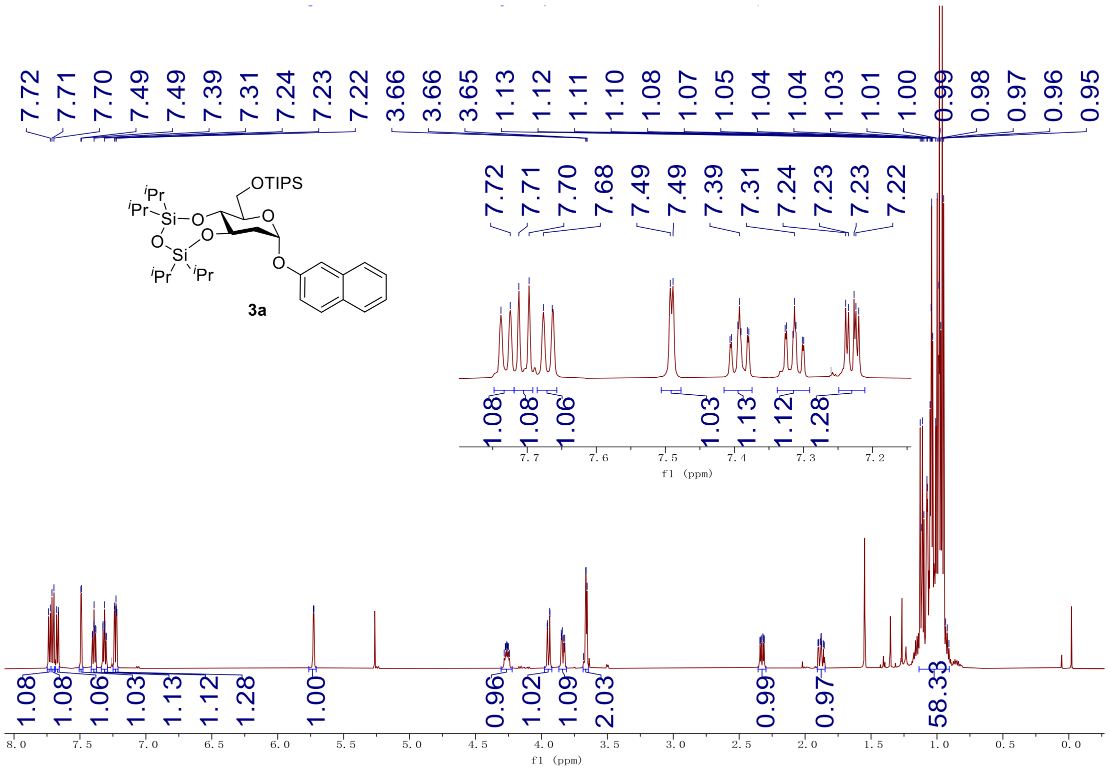

Figure S26. ^{1}H **NMR (600 MHz,** $CDCl_3$**) Spectra for compound 3a**

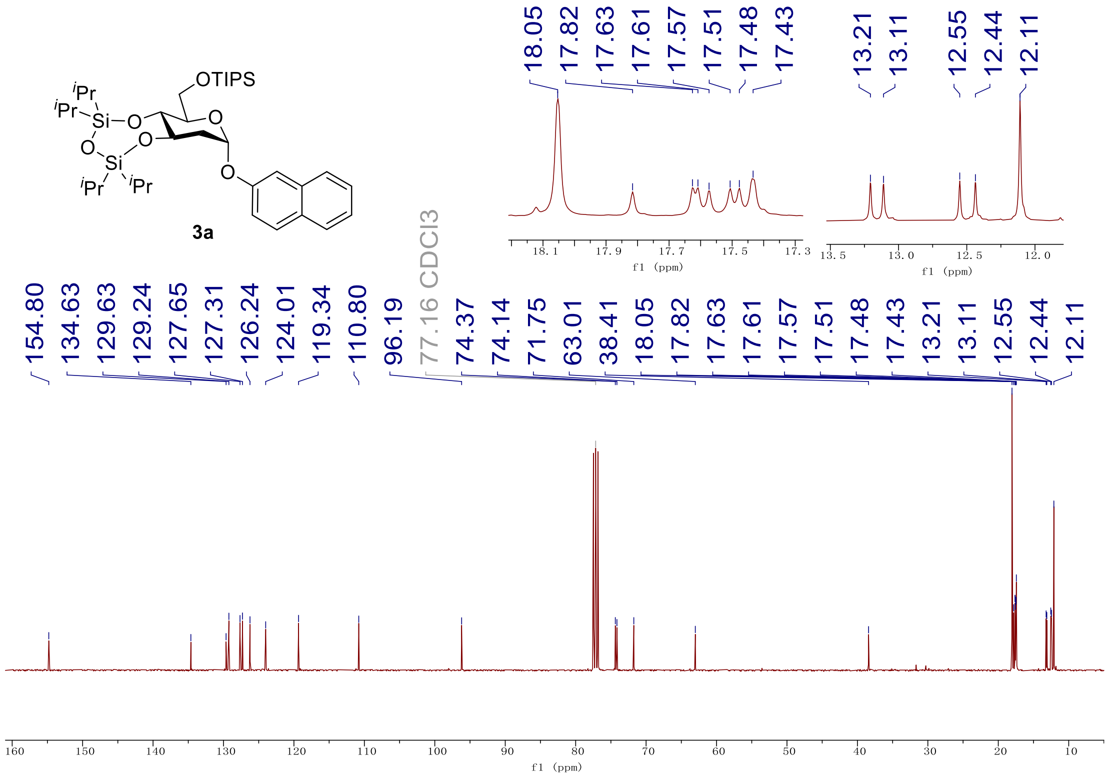

Figure S27. ^{13}C **NMR (101 MHz,** $CDCl_3$**) Spectra for compound 3a**

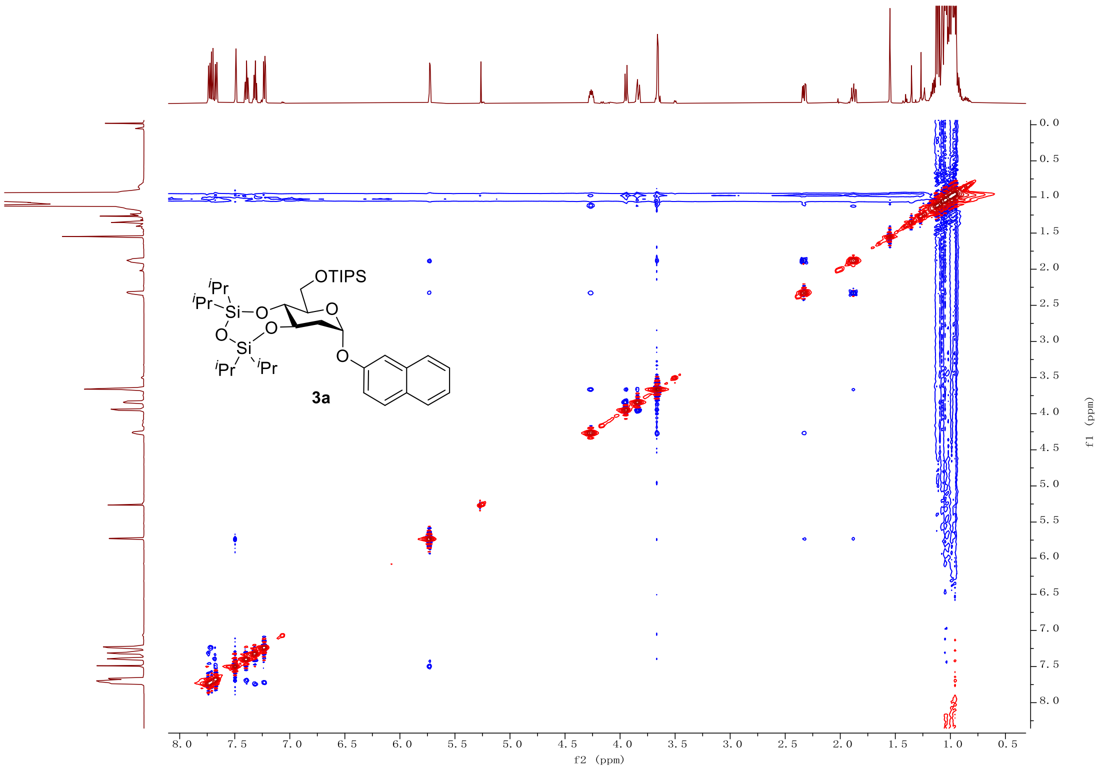

Figure S28: NOESY Spectra for 3a

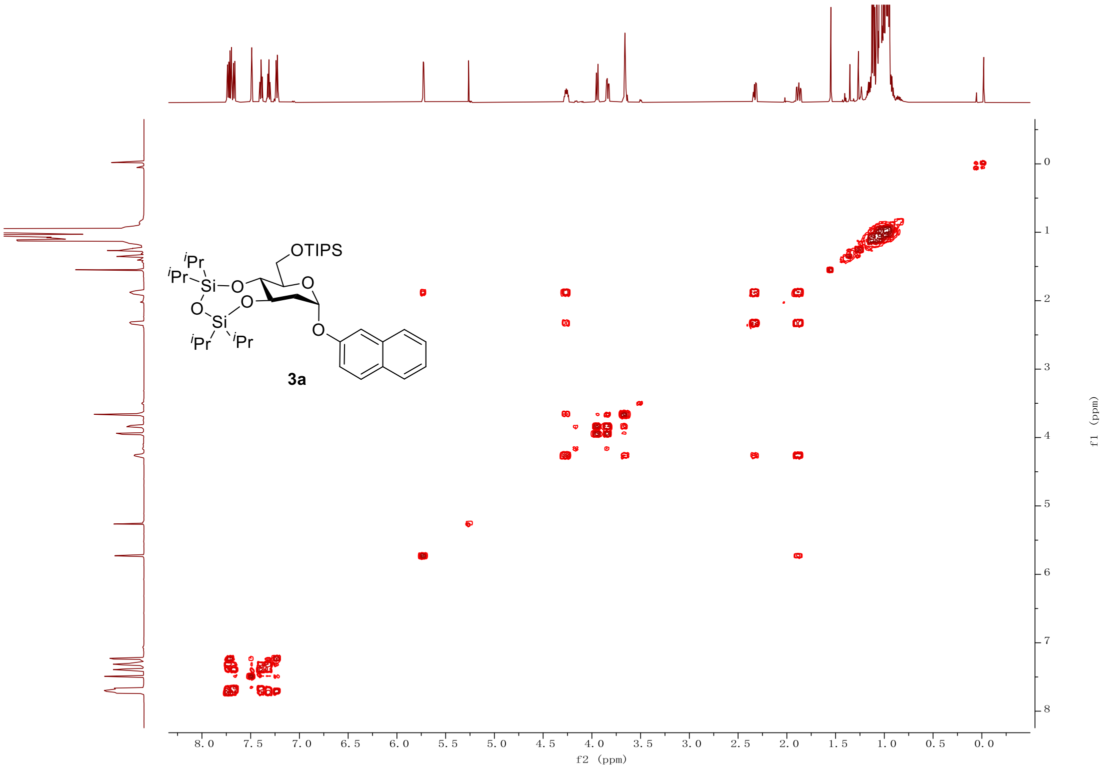

Figure S29: COSY Spectra for 3a

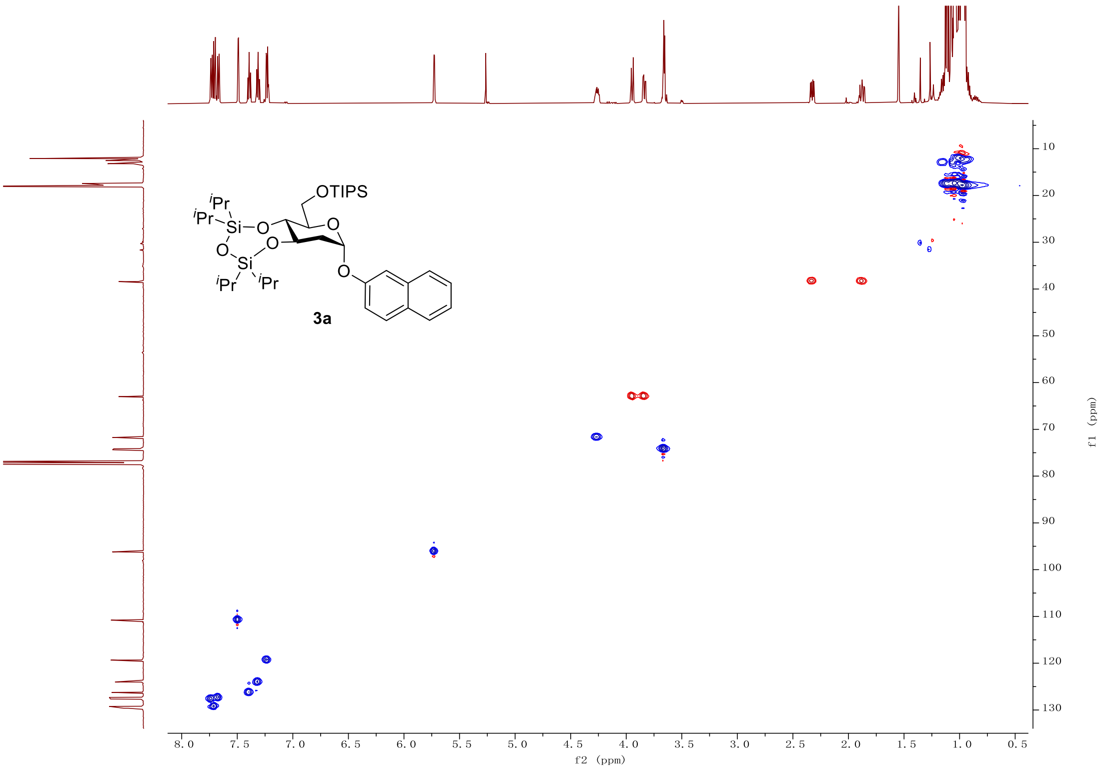

Figure S30: HSQC Spectra for 3a

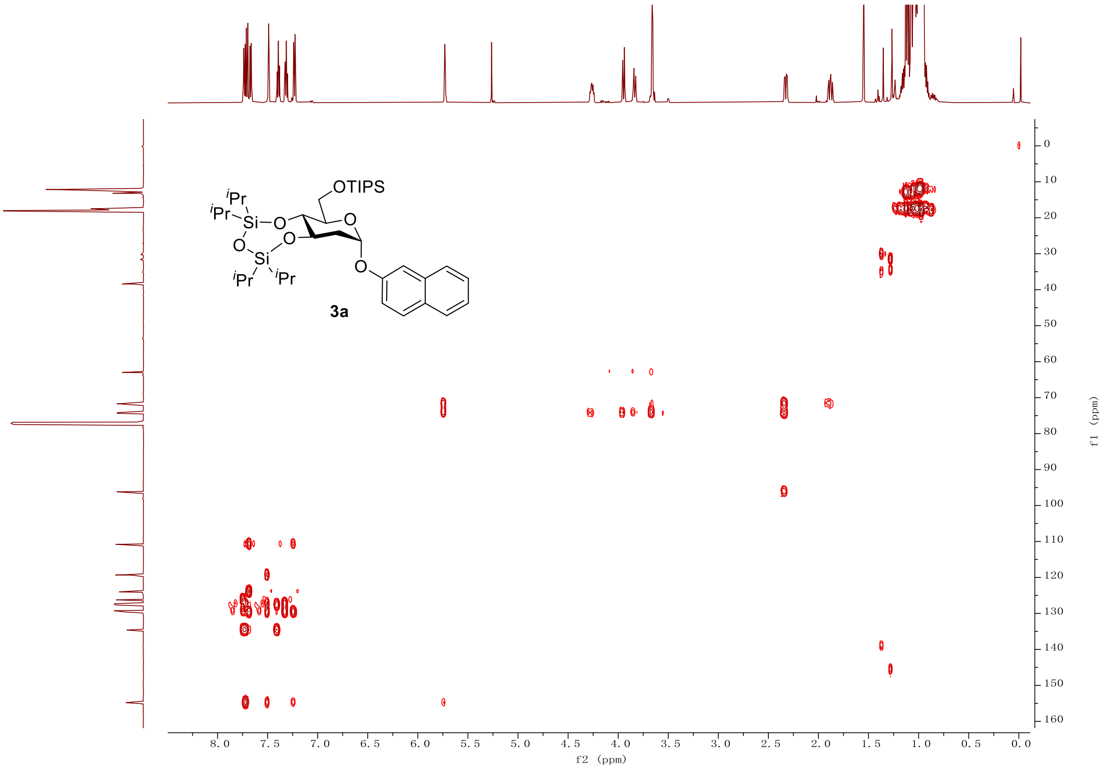

Figure S31: HMBC Spectra for 3a

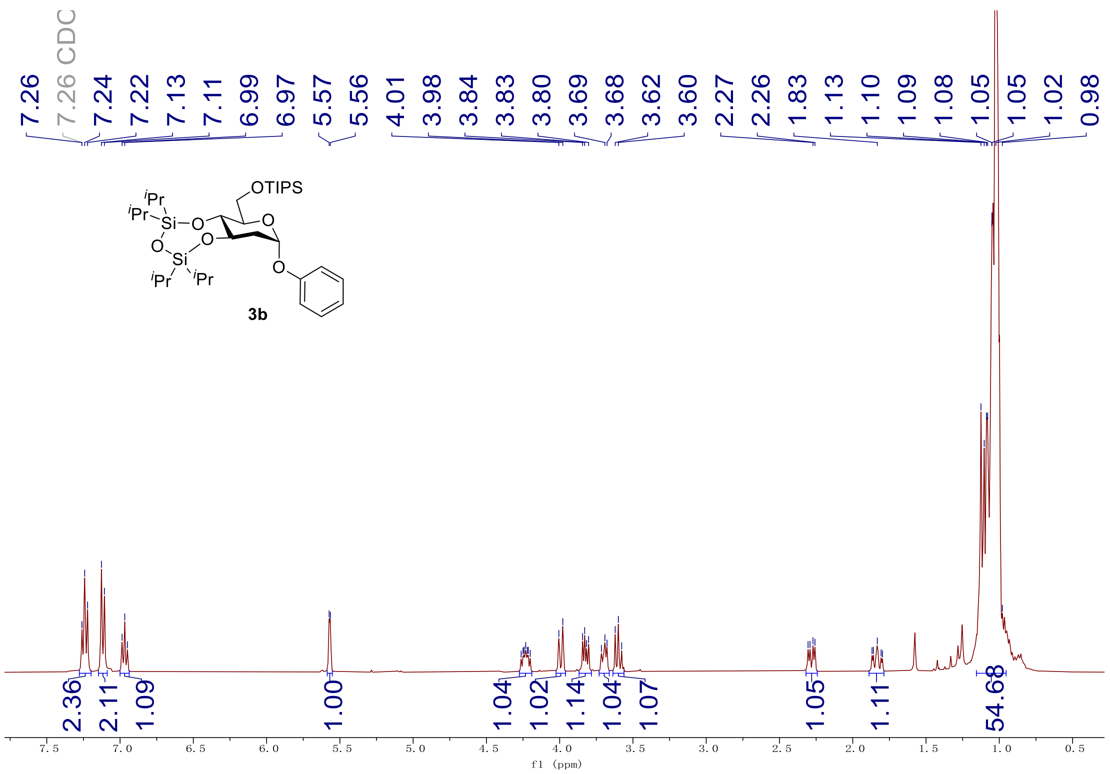

Figure S32. **^{1}H NMR (400 MHz, CDCl3) Spectra for compound 3b**

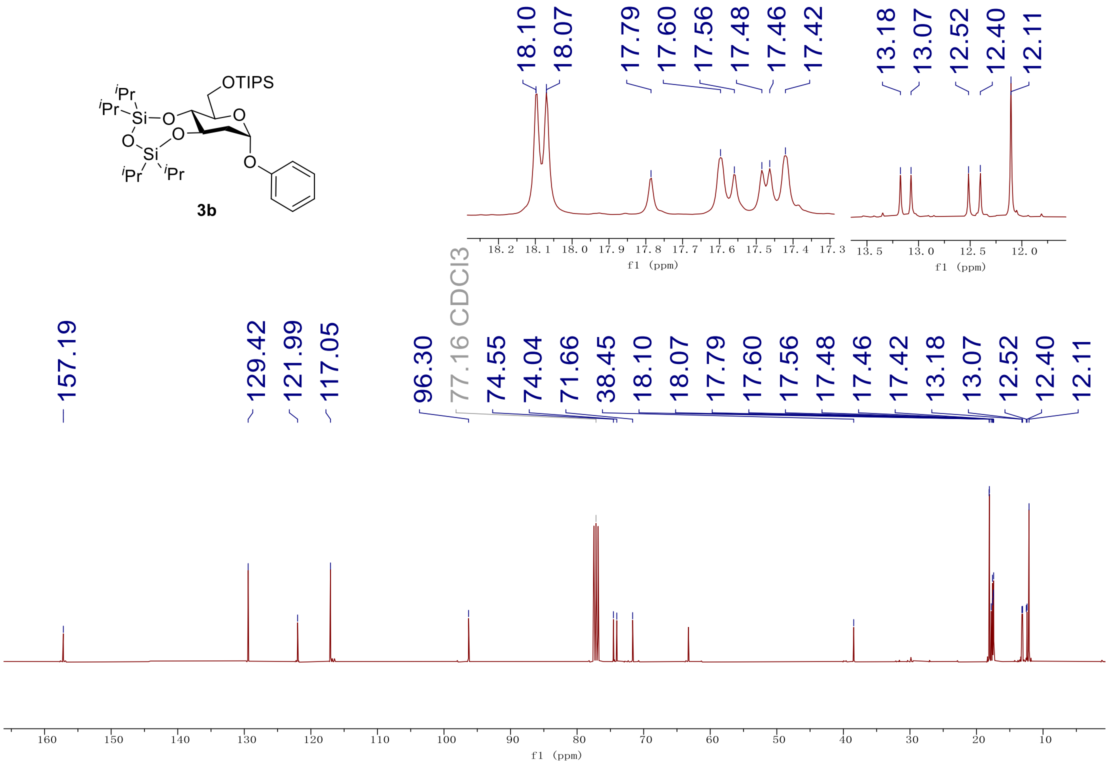

Figure S33. **^{13}C NMR (101 MHz, CDCl3) Spectra for compound 3b**

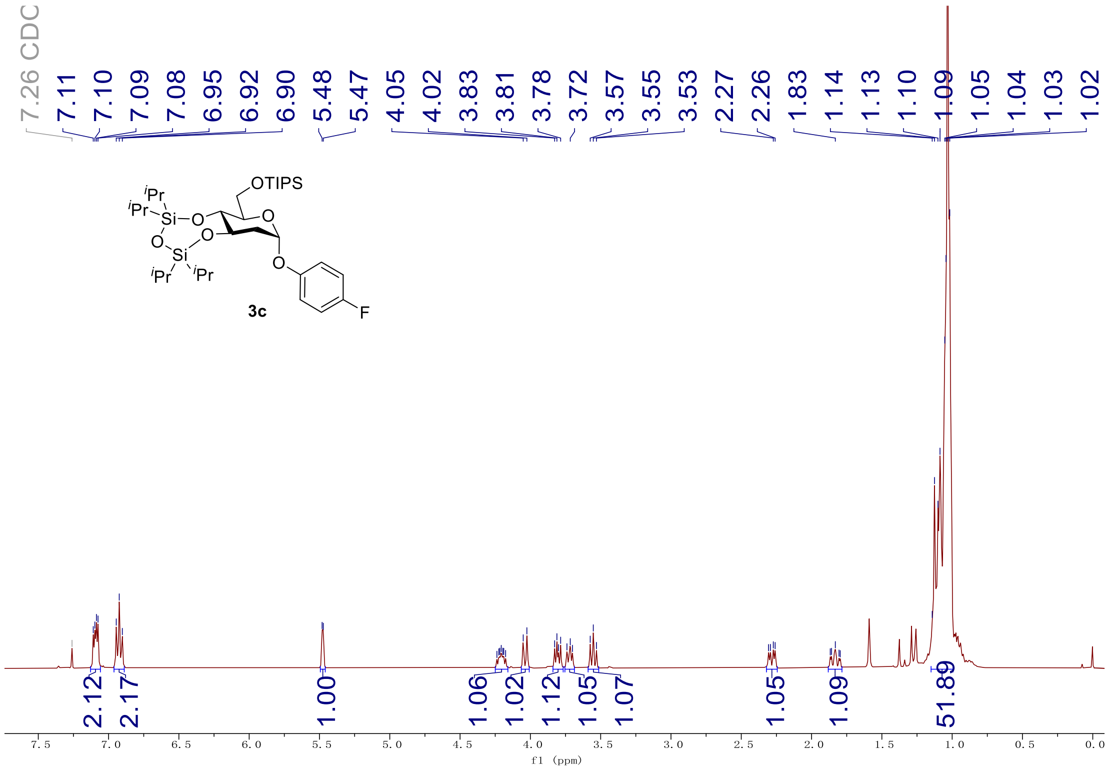

Figure S34. ^{1}H NMR (400 MHz, CDCl$_3$) Spectra for compound 1a

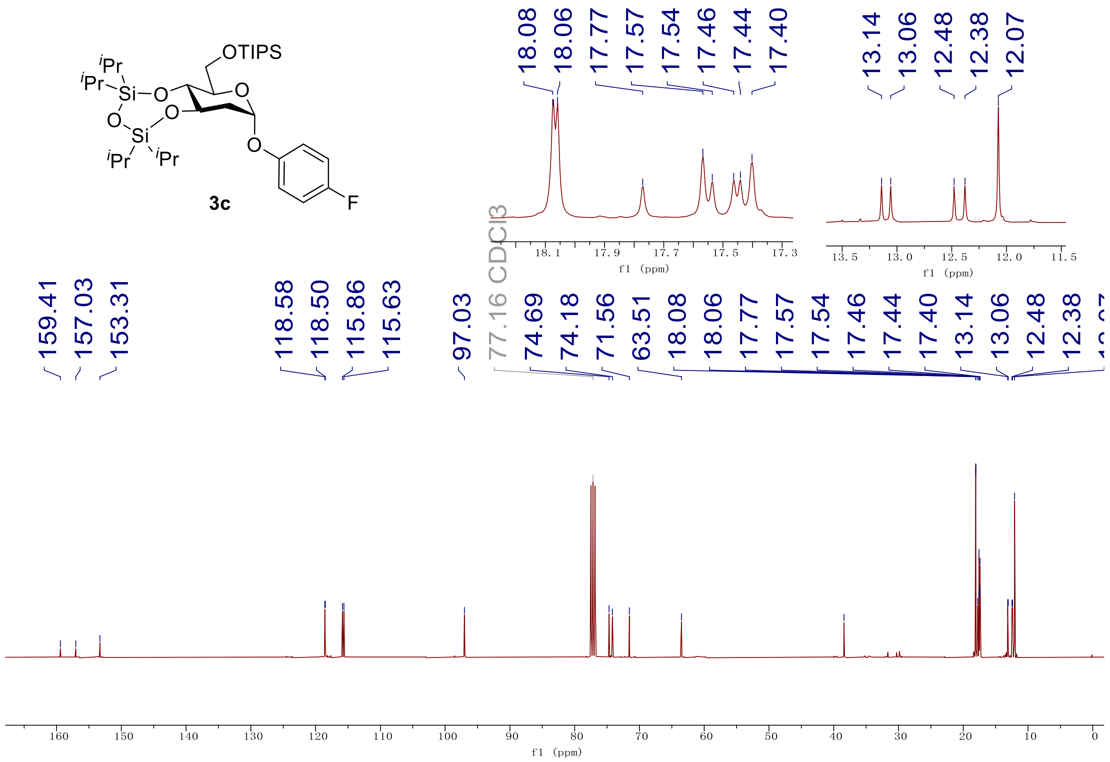

Figure S35. ^{13}C NMR (101 MHz, CDCl$_3$) Spectra for compound 1c

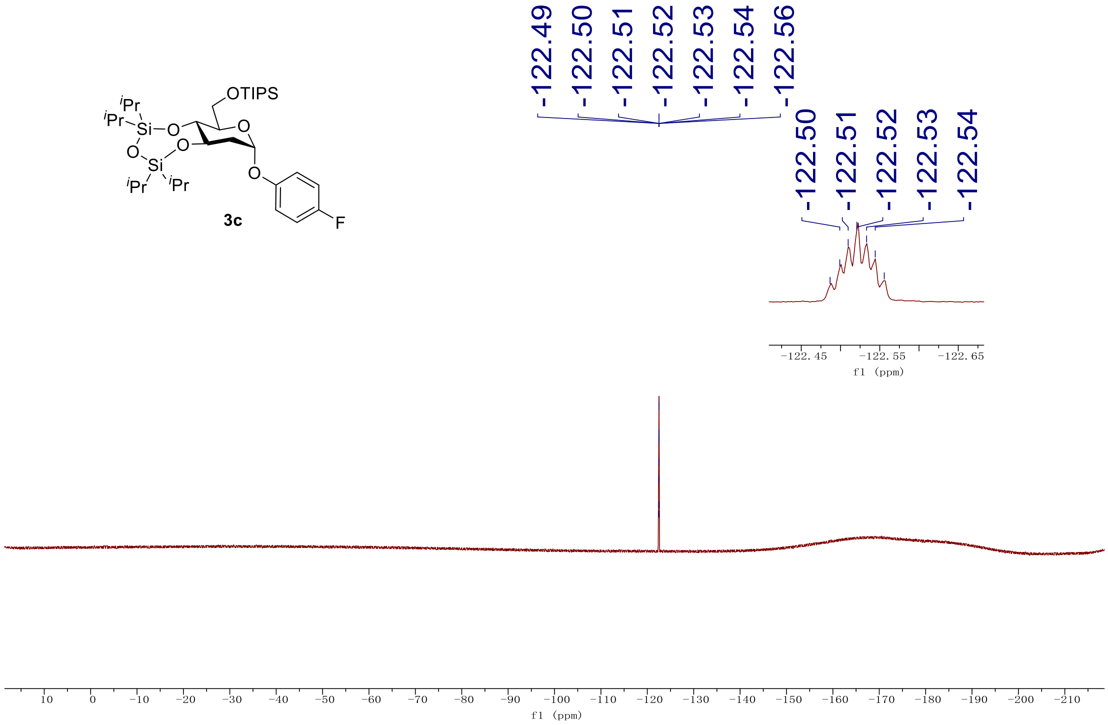

Figure S36. ^{19}F NMR (376 MHz, CDCl$_3$) Spectra for compound 3c

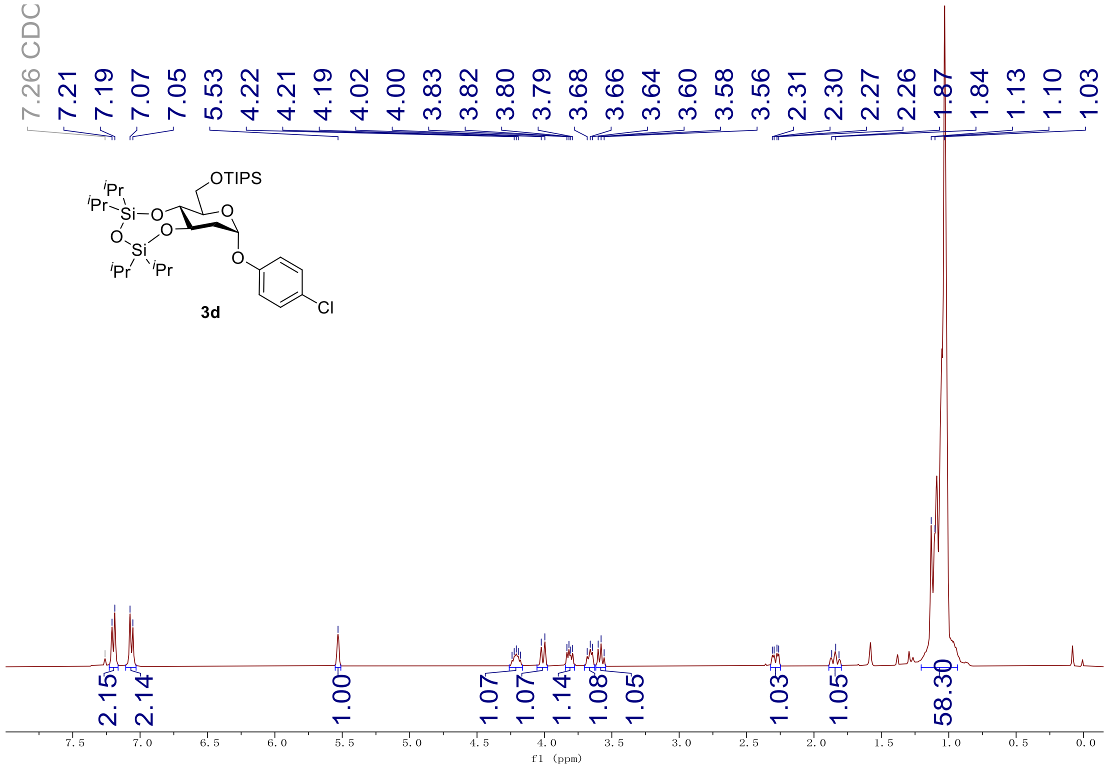

Figure S37. ^{1}H NMR (400 MHz, CDCl$_3$) Spectra for compound 3d

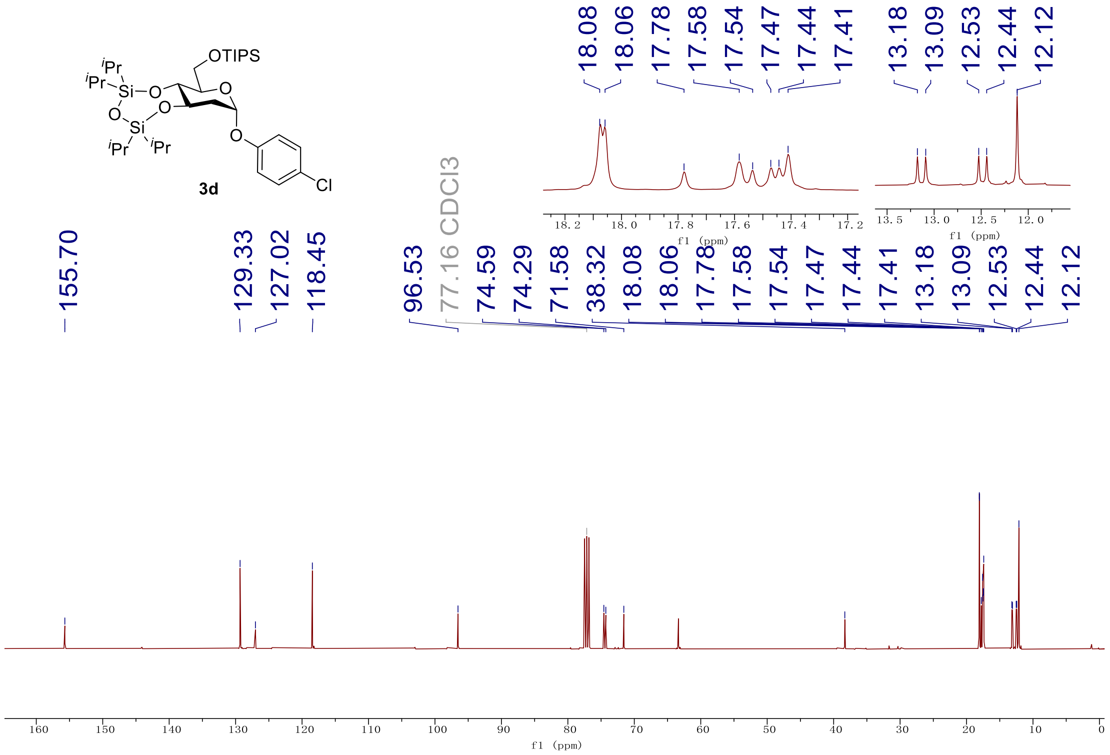

Figure S38. ^{13}C **NMR (101 MHz,** $CDCl_3$**) Spectra for compound 3d**

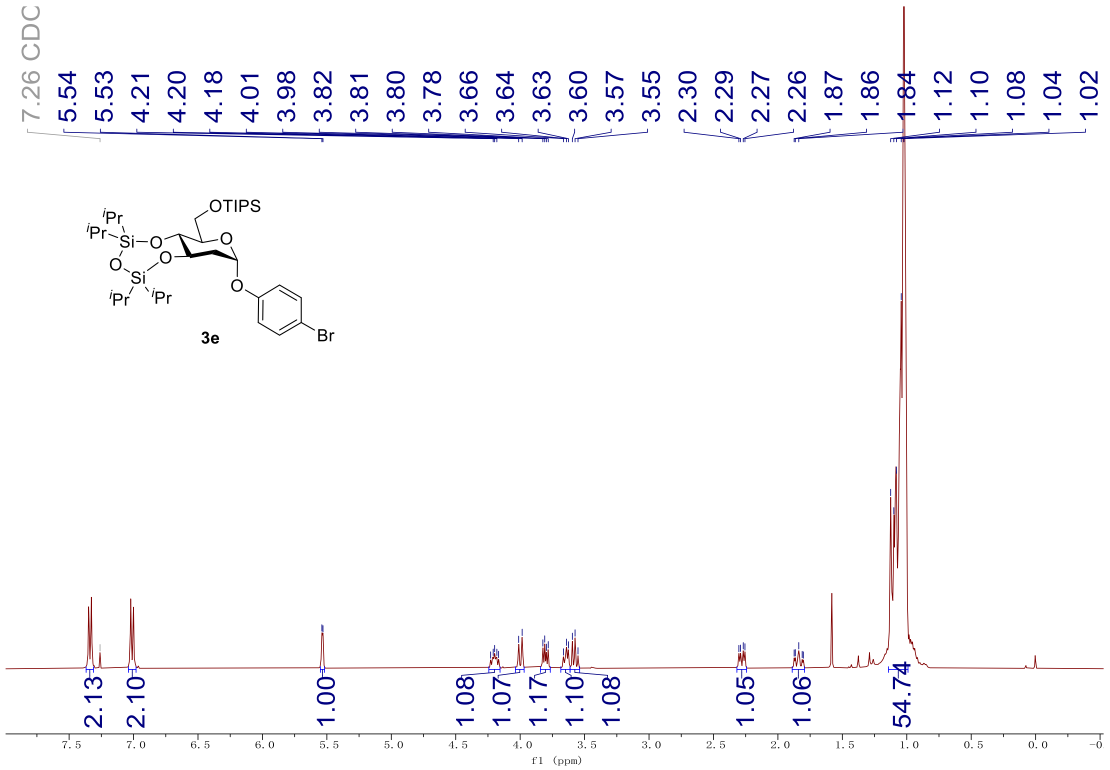

Figure S39. ^{1}H **NMR (400 MHz,** $CDCl_3$**) Spectra for compound 3e**

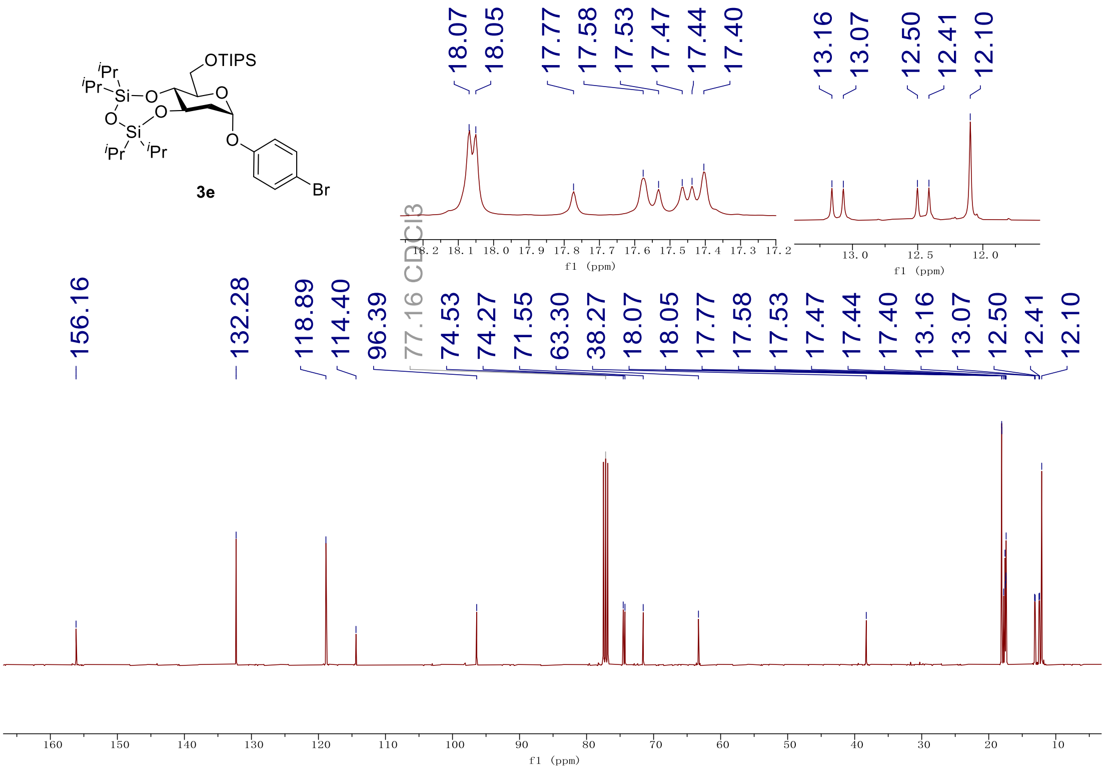

Figure S40. ^{13}**C NMR (101 MHz, CDCl$_3$) Spectra for compound 3e**

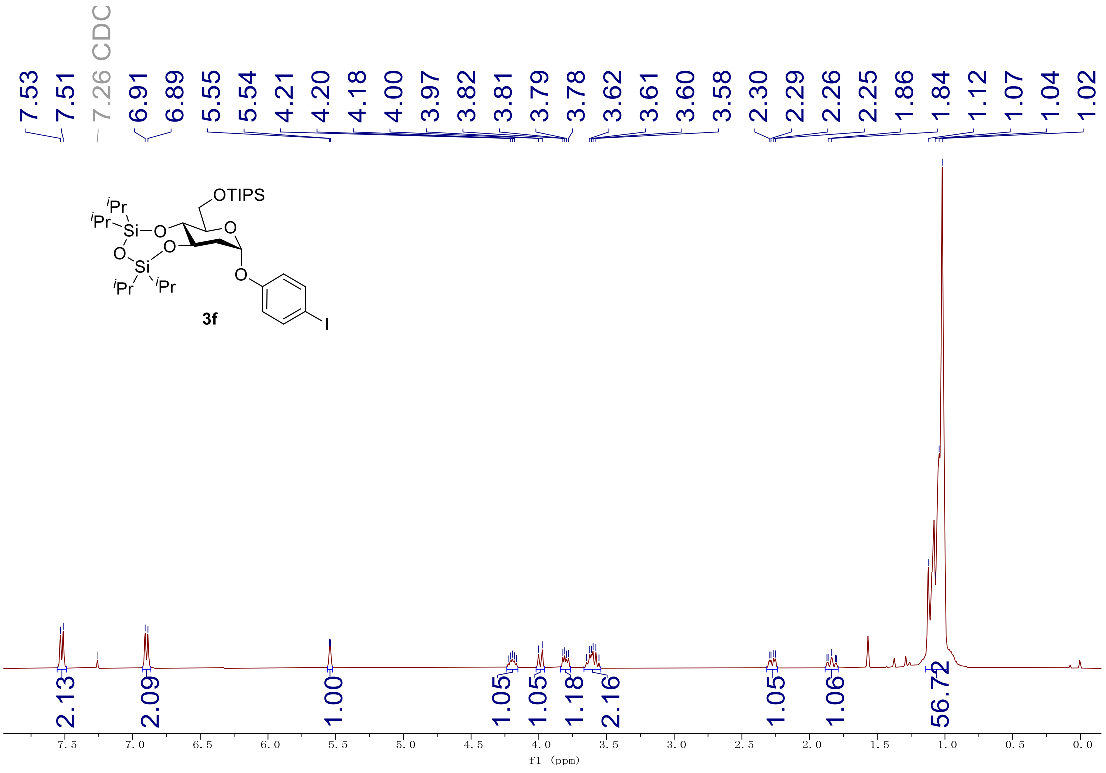

Figure S41. ^{1}**H NMR (400 MHz, CDCl$_3$) Spectra for compound 3f**

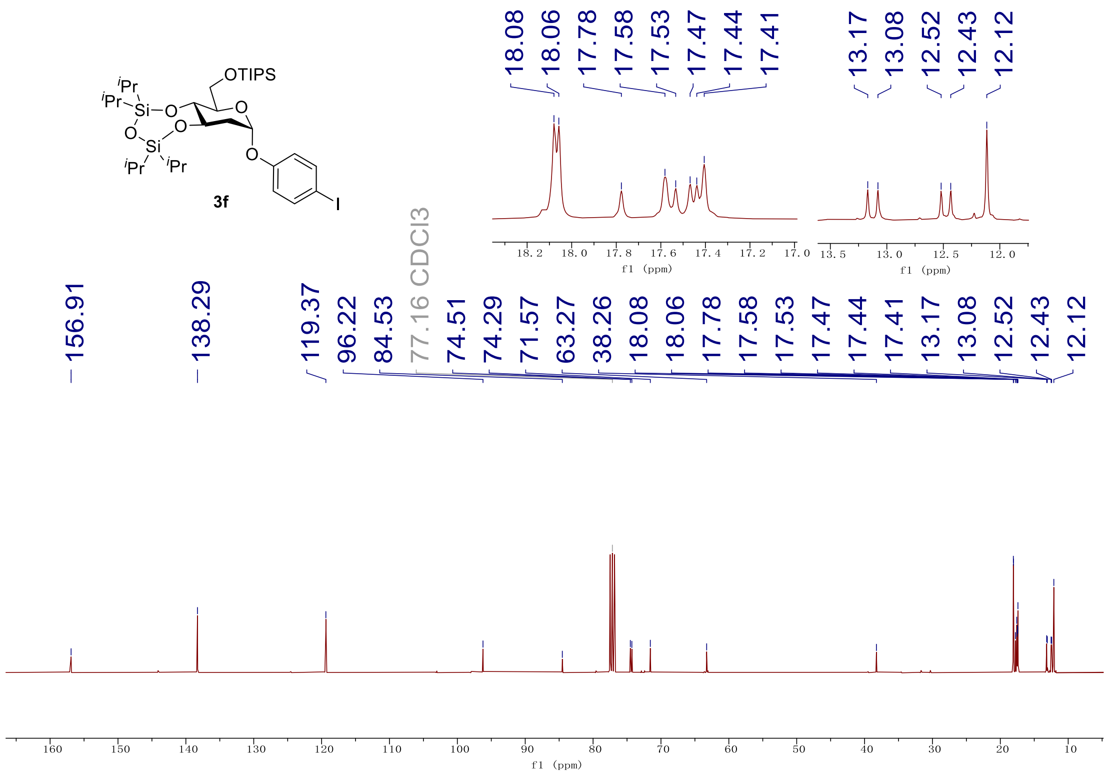

Figure S42. ^{13}**C NMR (101 MHz, CDCl$_3$) Spectra for compound 3f**

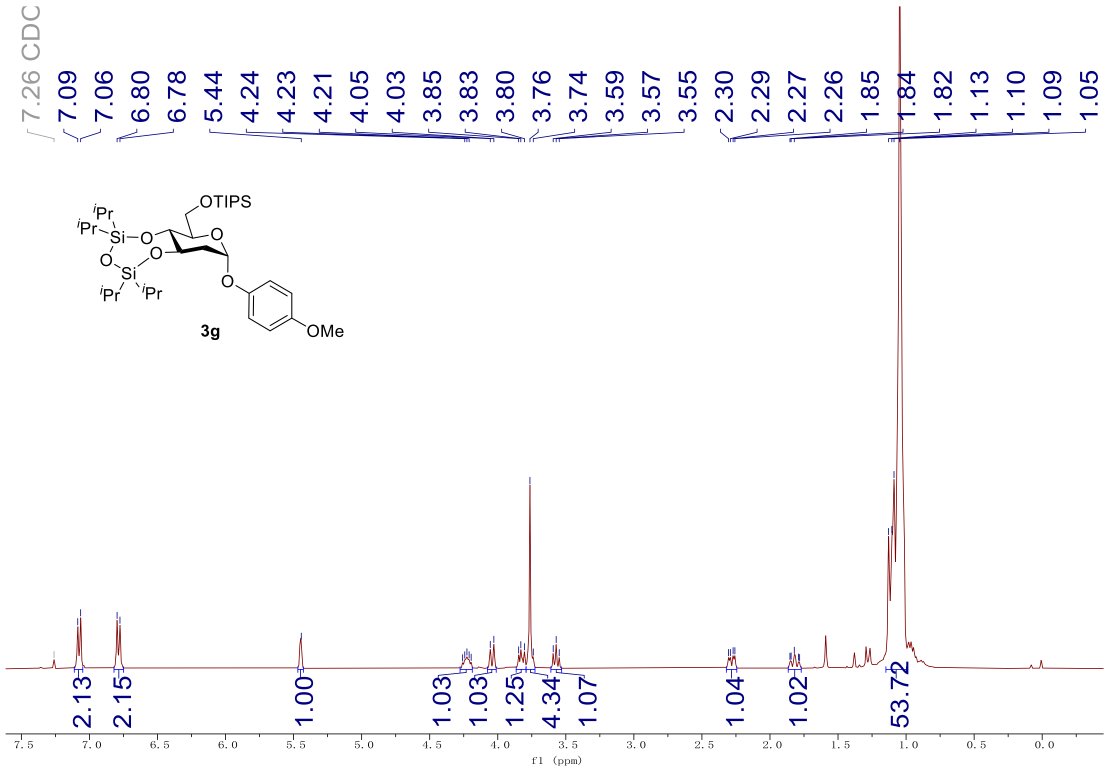

Figure S43. ^{1}**H NMR (400 MHz, CDCl$_3$) Spectra for compound 3g**

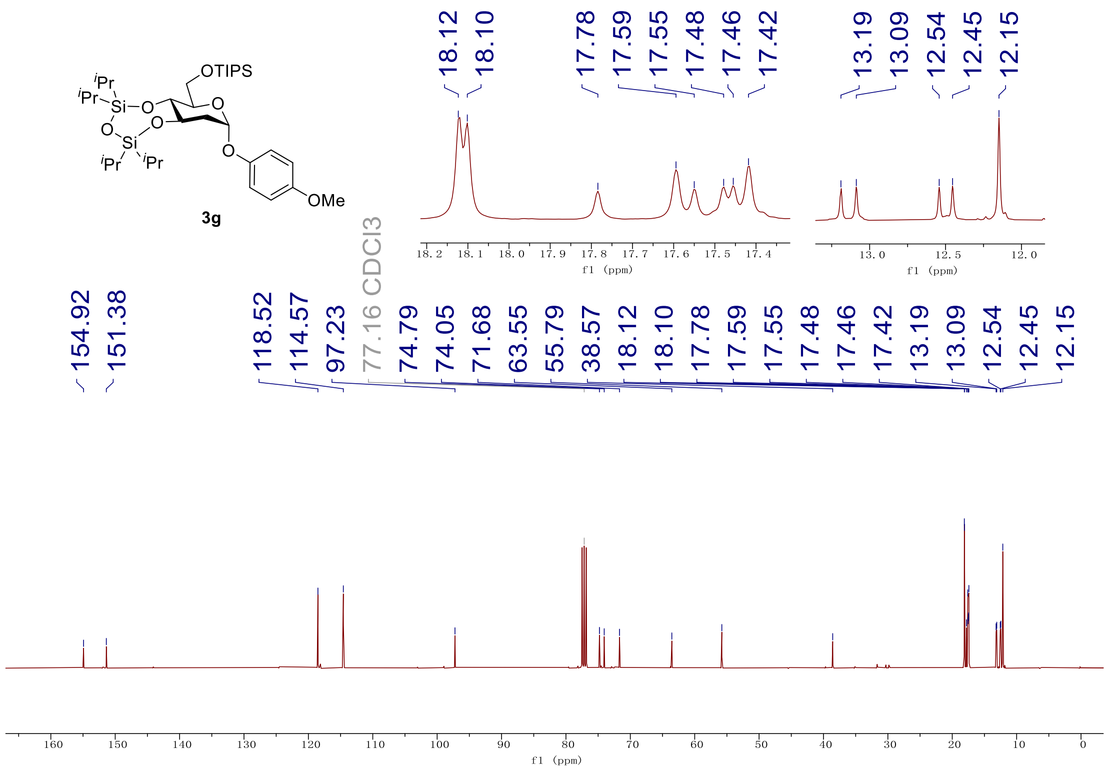

Figure S44. ^{13}C NMR (101 MHz, CDCl$_3$) Spectra for compound 3g

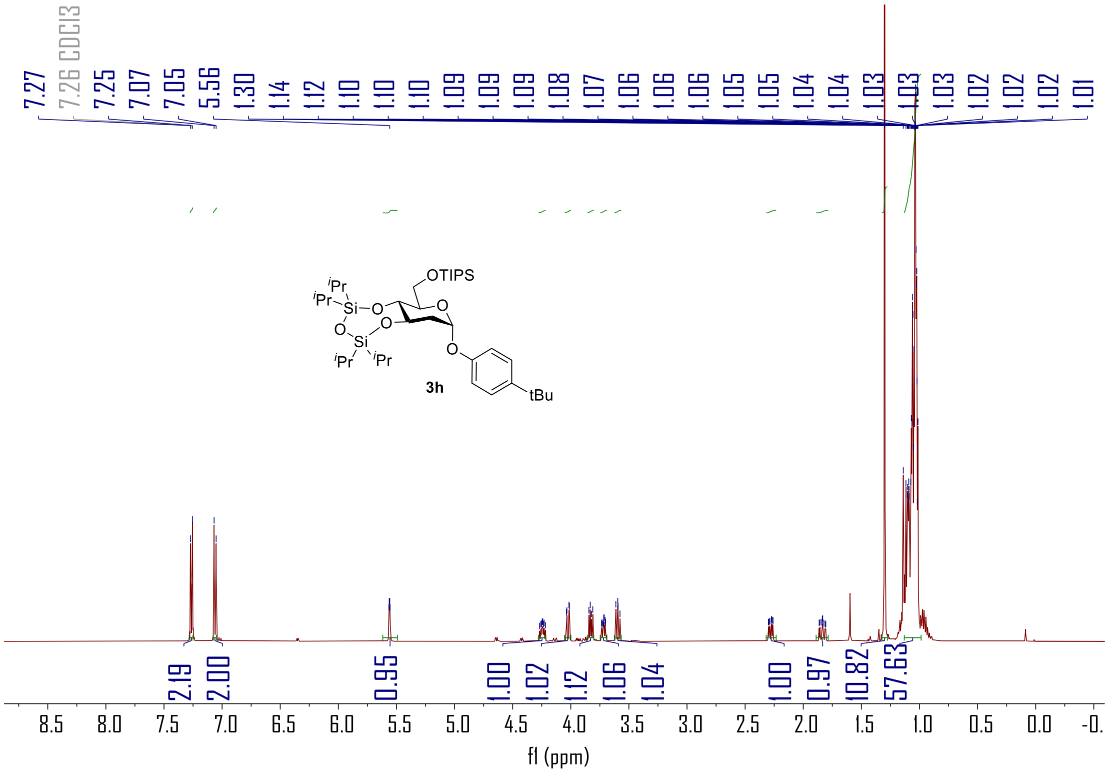

Figure S345. ^{1}H NMR (400 MHz, CDCl$_3$) Spectra for compound 3h

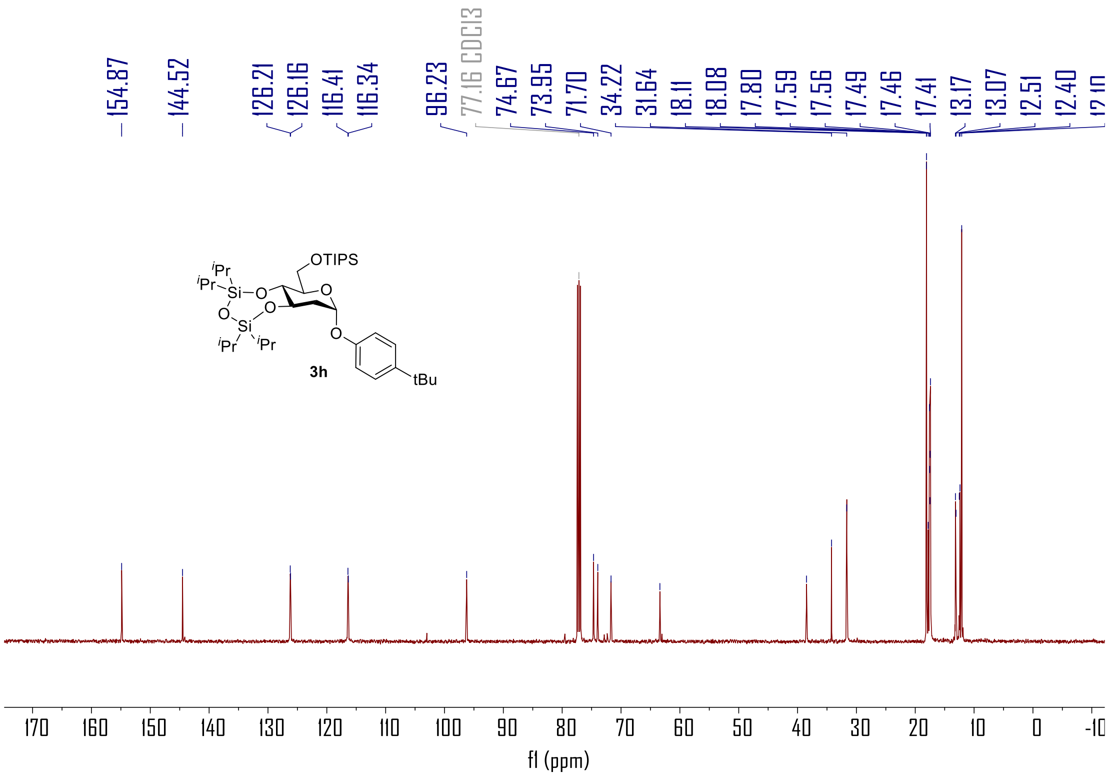

Figure S46. ^{13}**C NMR (101 MHz, CDCl$_3$) Spectra for compound 3h**

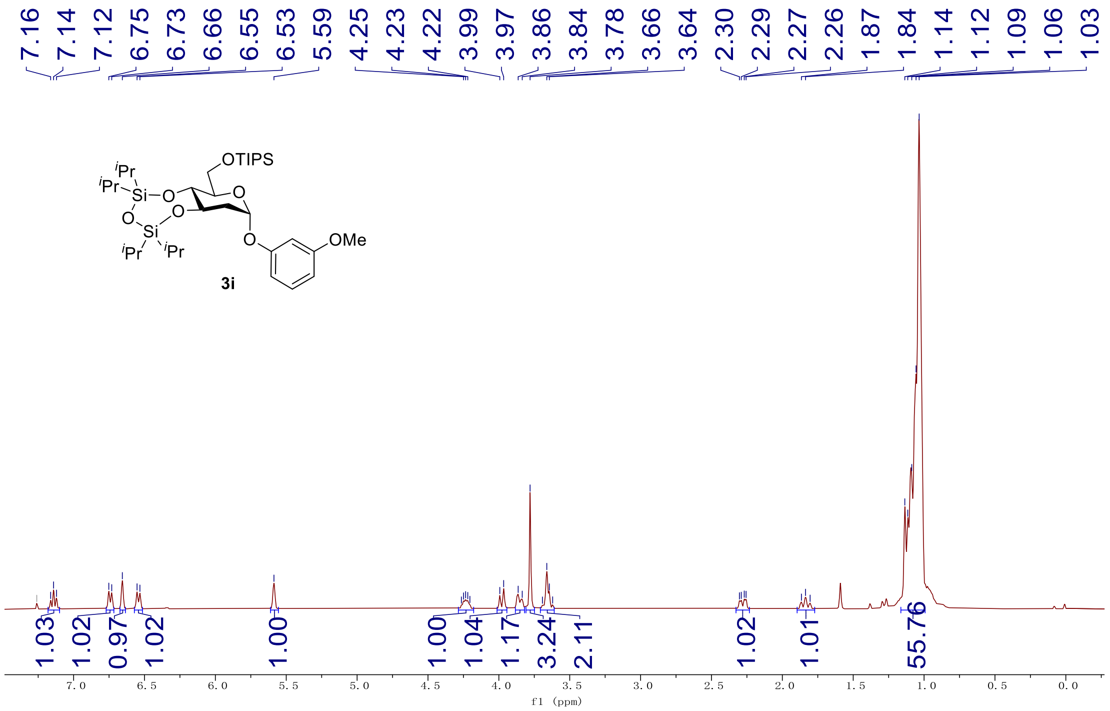

Figure S47. ^{1}**H NMR (400 MHz, CDCl$_3$) Spectra for compound 3i**

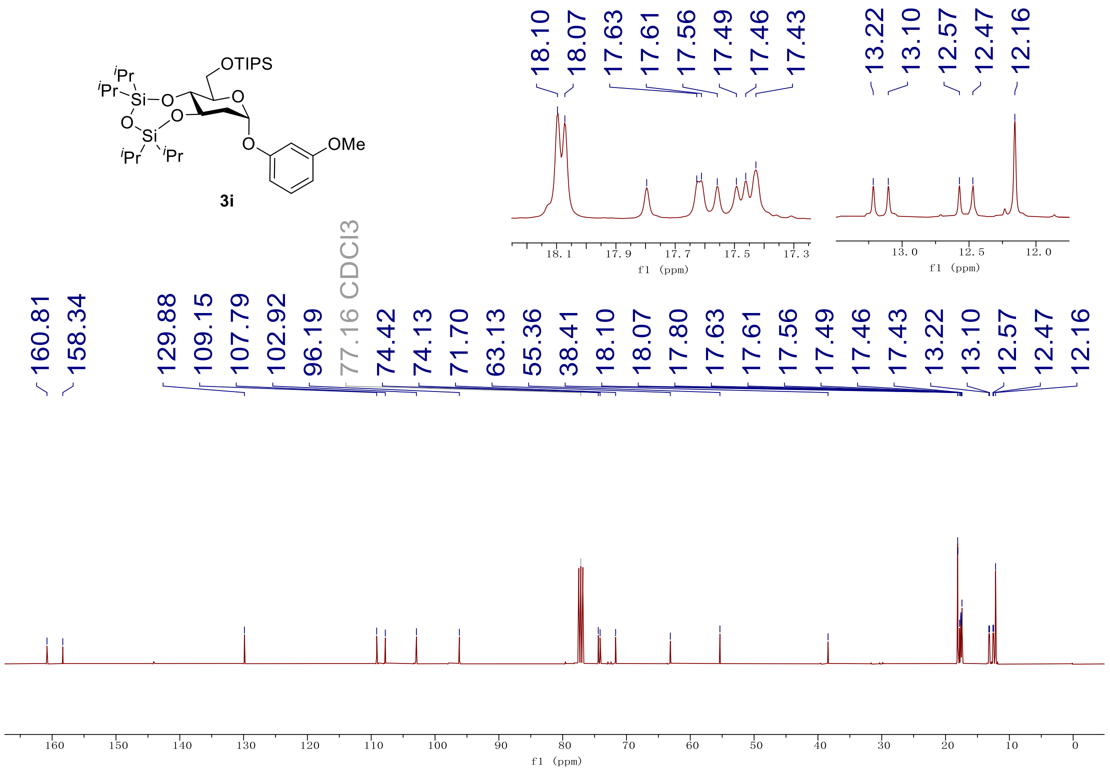

Figure S48. **^{13}C NMR (101 MHz, CDCl₃) Spectra for compound 3i**

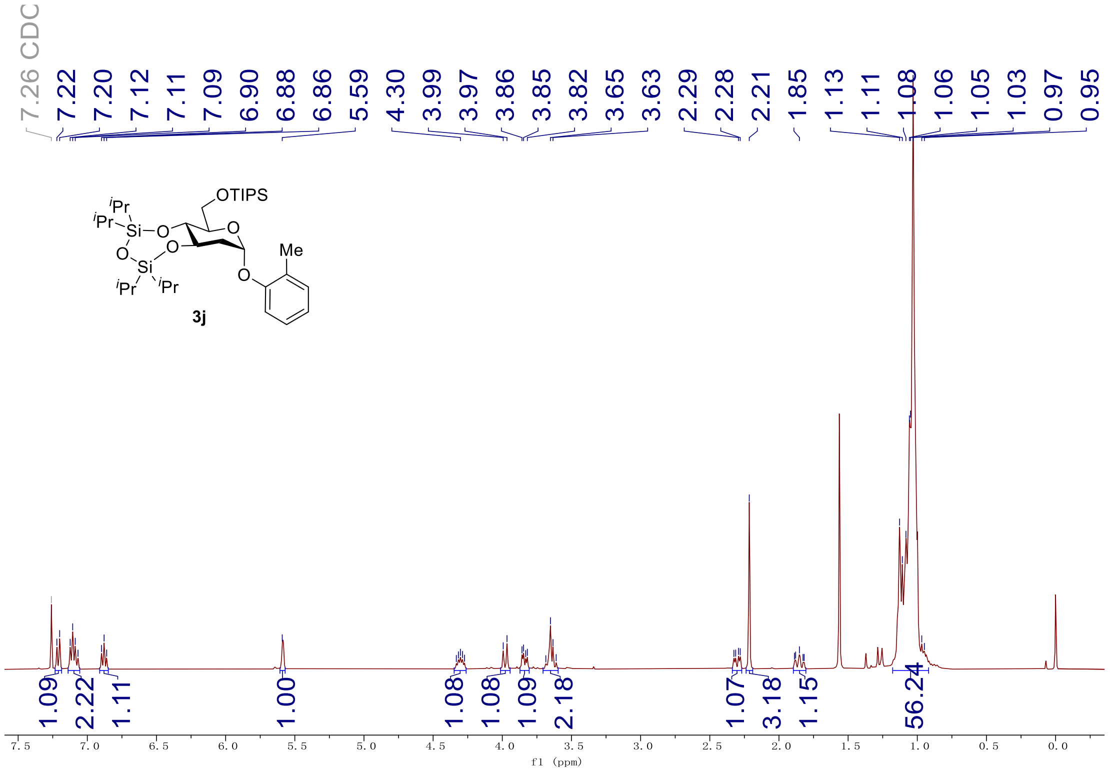

Figure S49. **^{1}H NMR (400 MHz, CDCl₃) Spectra for compound 3j**

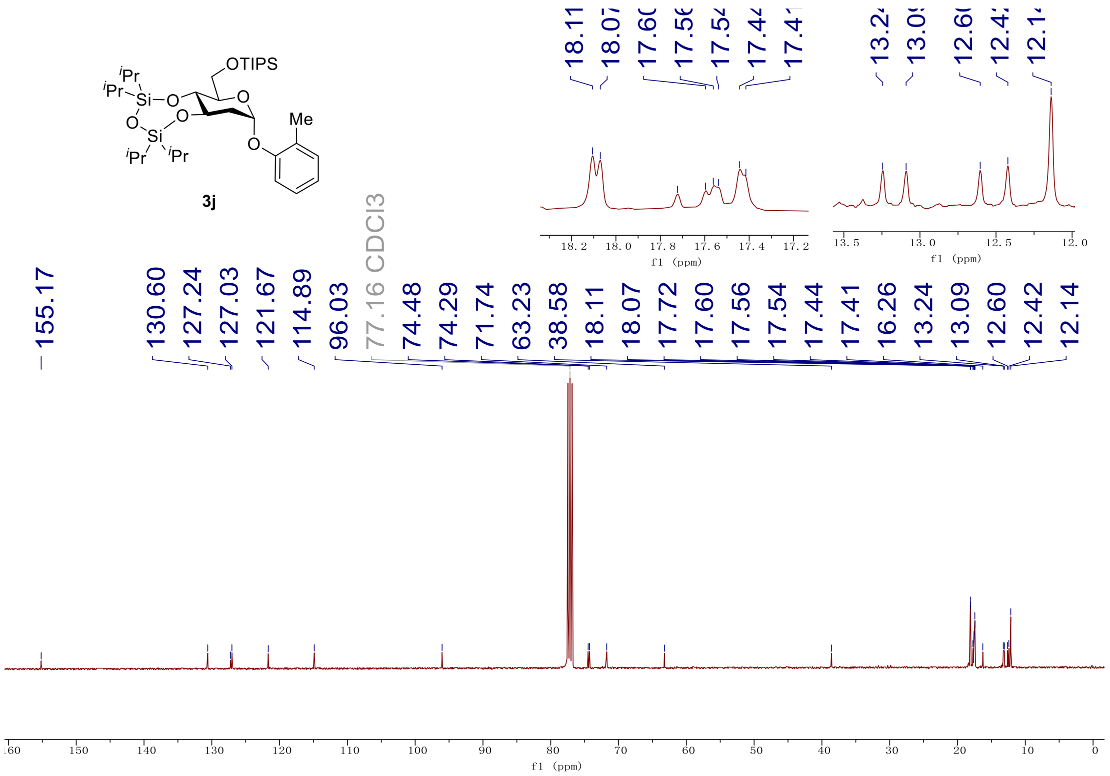

Figure S50. ^{13}**C NMR (101 MHz, CDCl$_3$) Spectra for compound 3j**

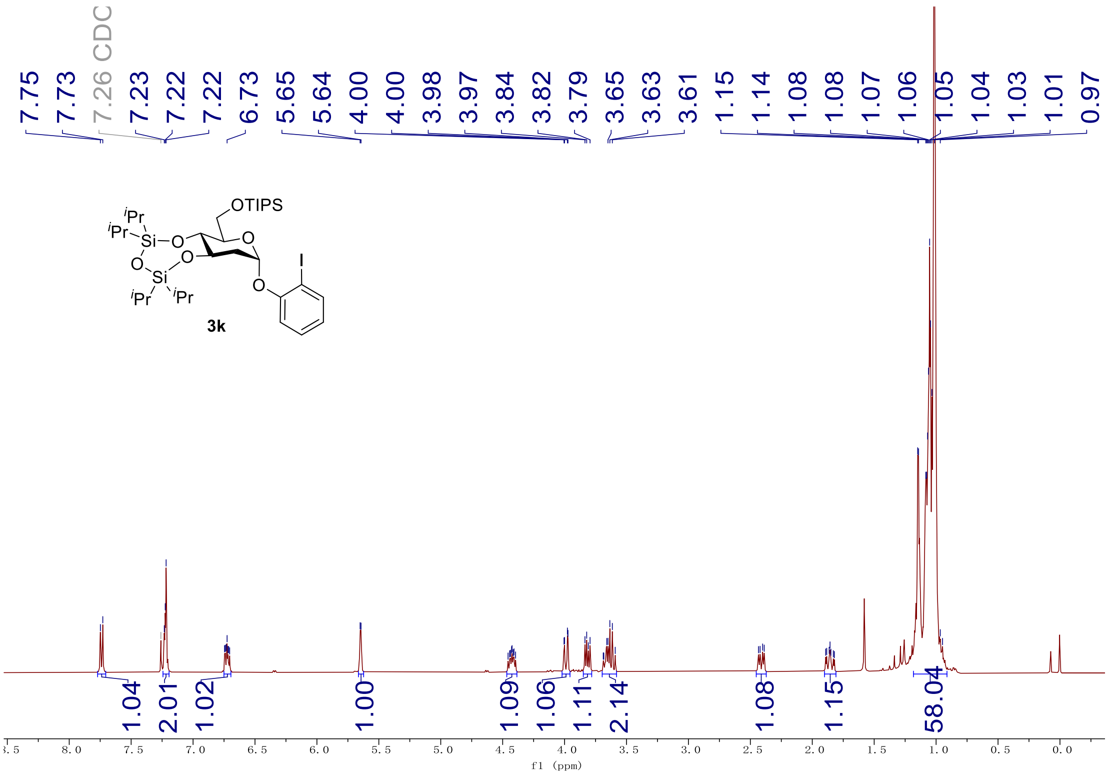

Figure S51. ^{1}**H NMR (400 MHz, CDCl$_3$) Spectra for compound 3k**

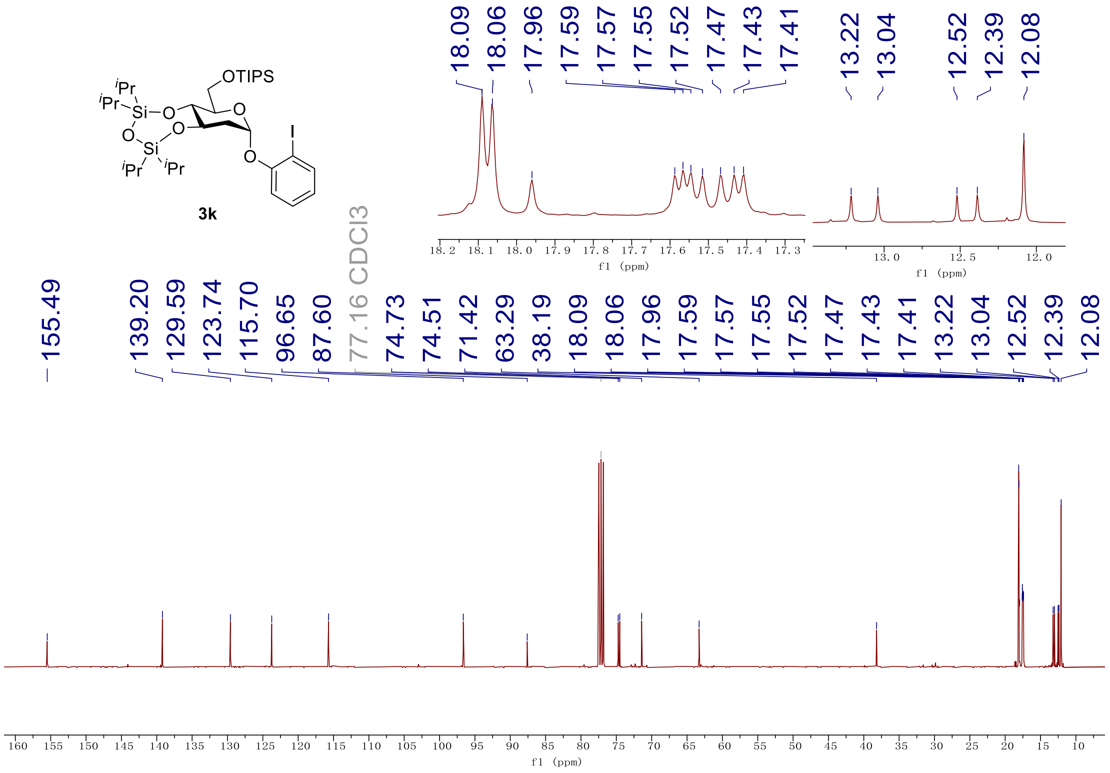

Figure S52. ^{13}C NMR (101 MHz, $CDCl_3$) Spectra for compound 3k

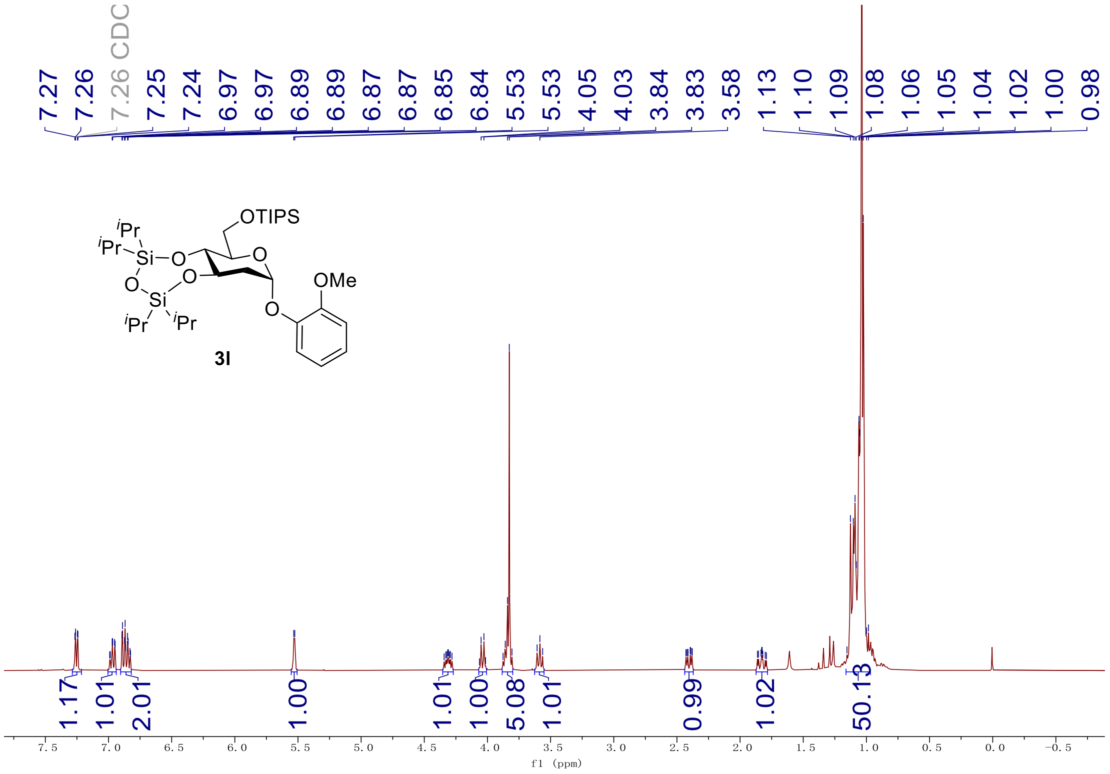

Figure S53. ^{1}H NMR (400 MHz, $CDCl_3$) Spectra for compound 3l

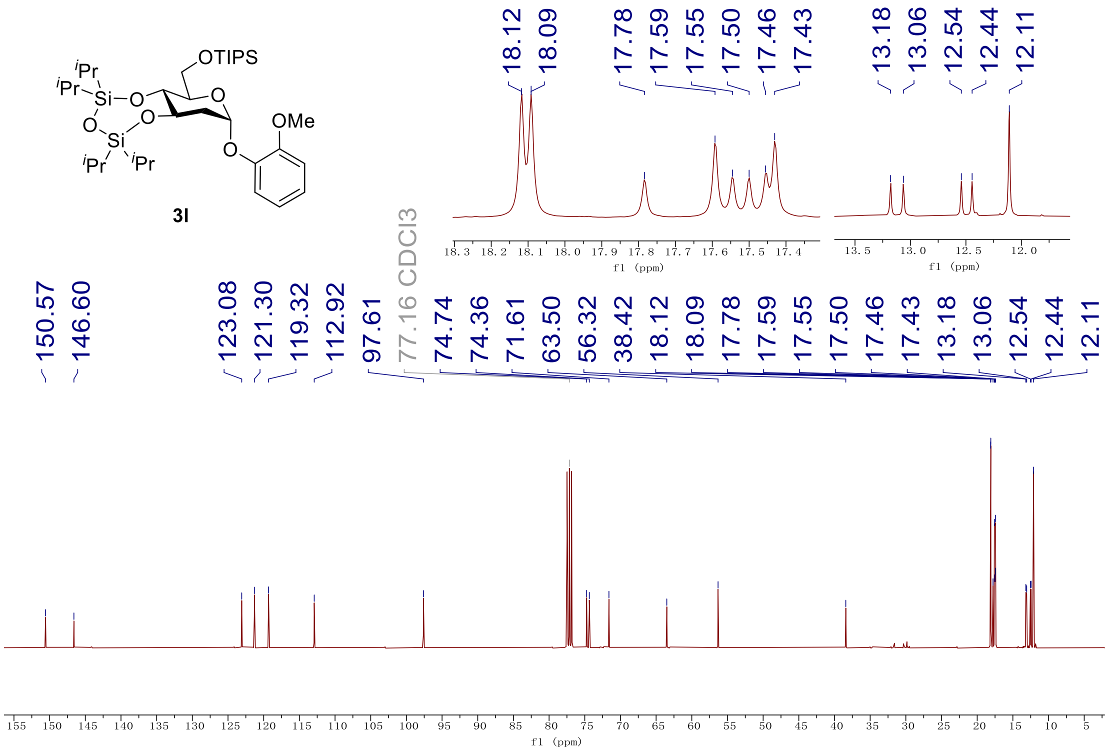

Figure S54. ^{13}**C NMR (101 MHz, CDCl**$_3$**) Spectra for compound 3l**

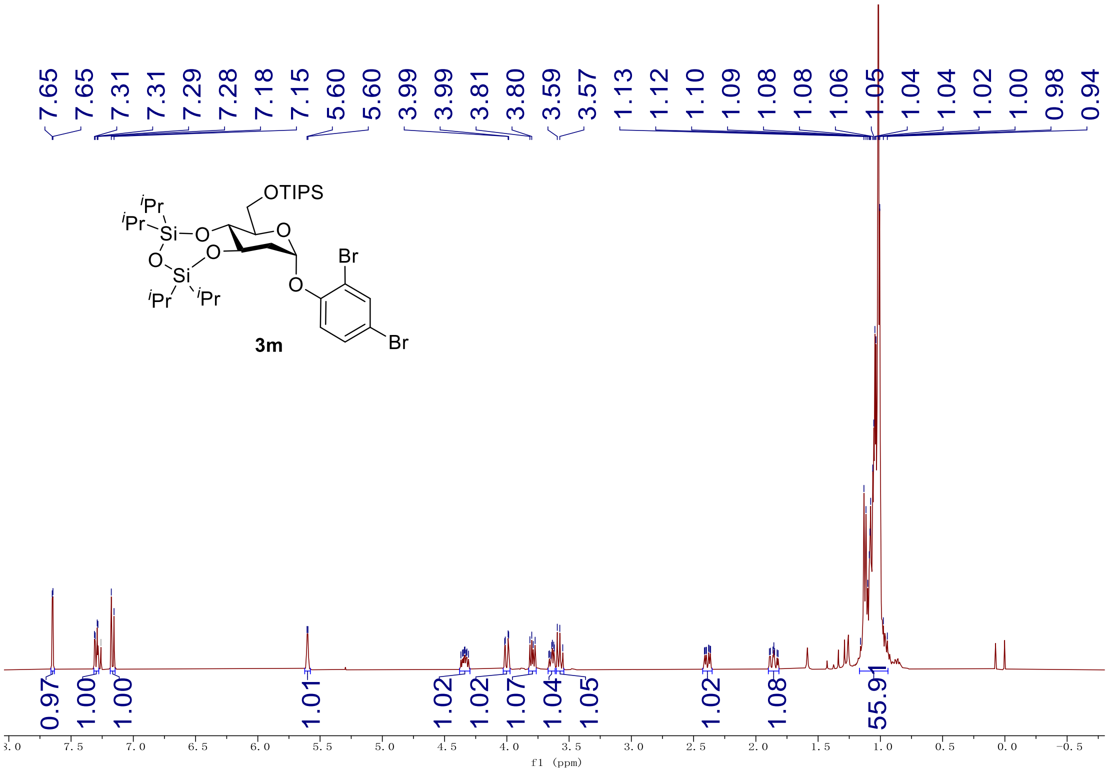

Figure S55. ^{1}**H NMR (400 MHz, CDCl**$_3$**) Spectra for compound 3m**

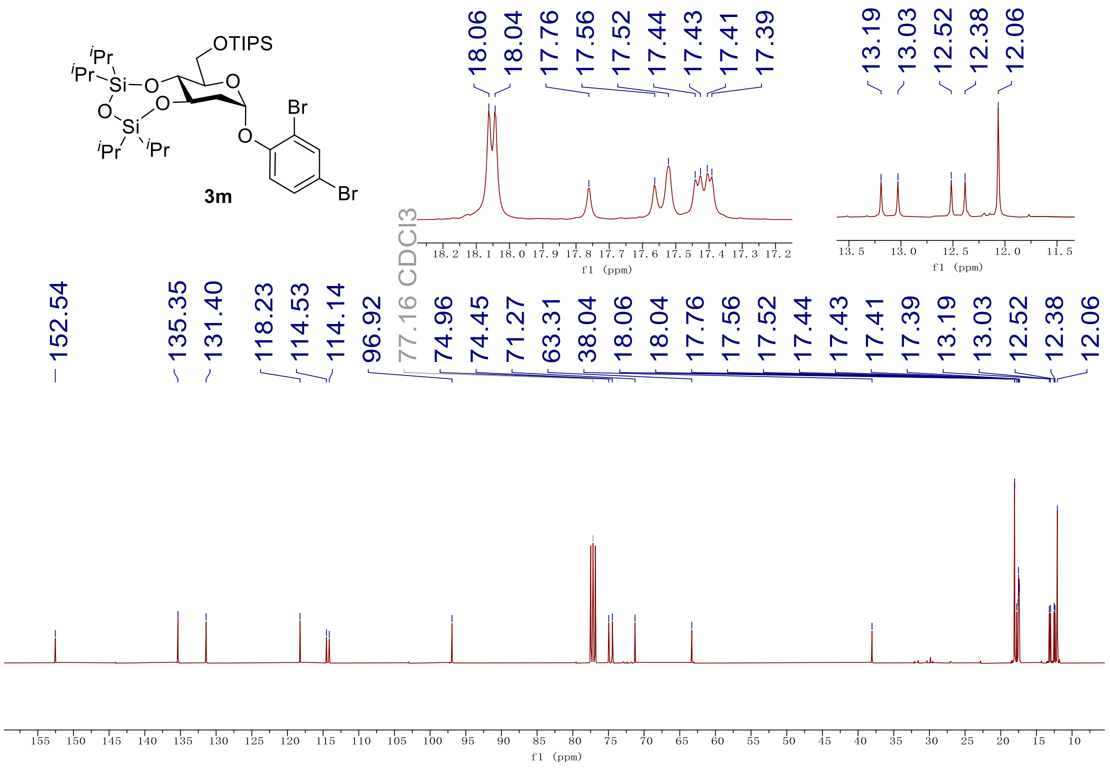

Figure S56. ^{13}C NMR (101 MHz, CDCl$_3$) Spectra for compound 3m

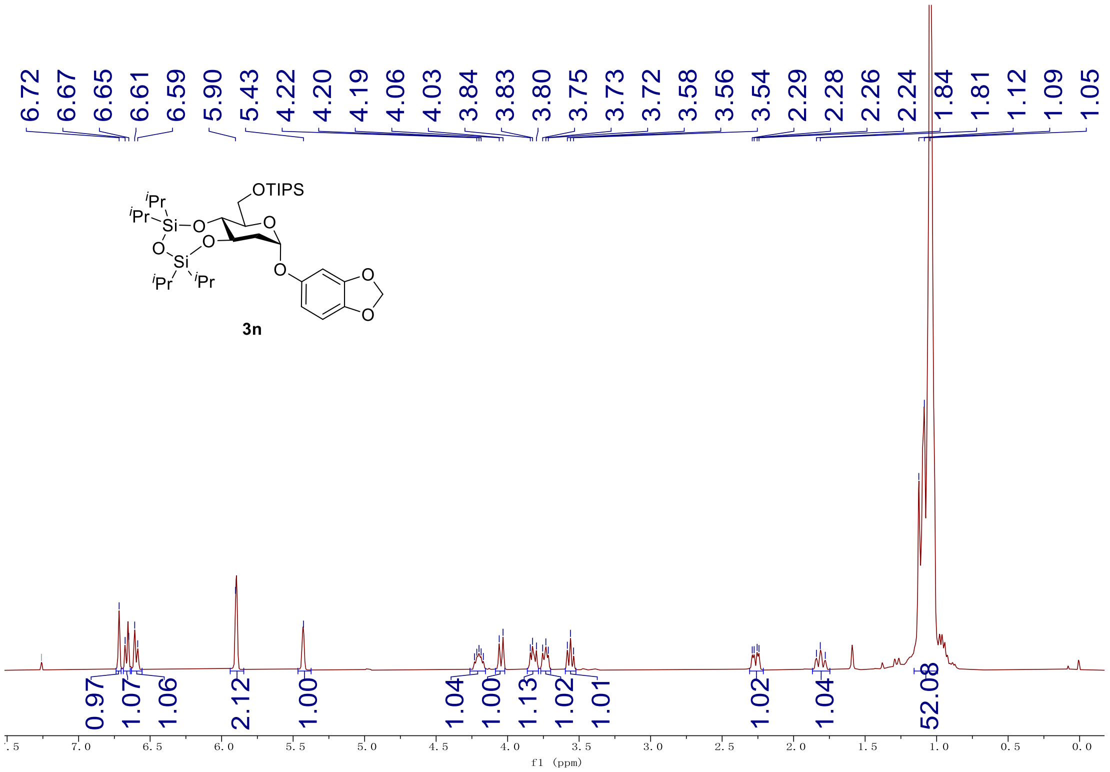

Figure S57. ^{1}H NMR (400 MHz, CDCl$_3$) Spectra for compound 3n

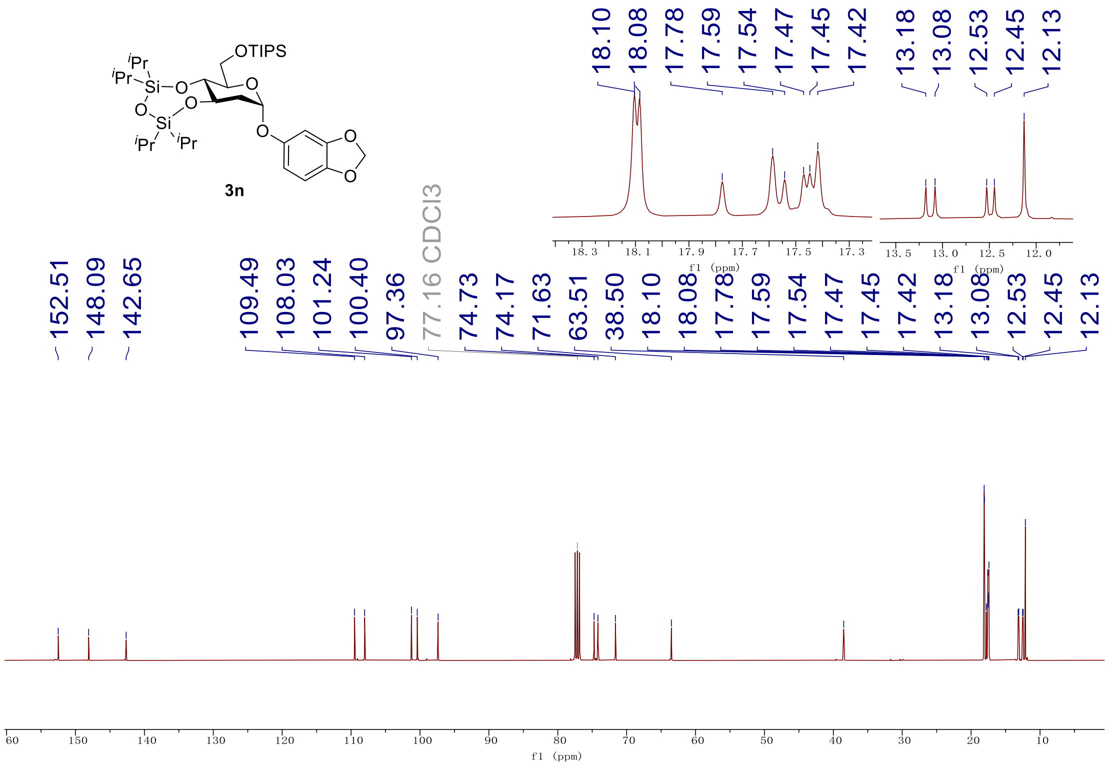

Figure S58. ^{13}C NMR (101 MHz, $CDCl_3$) Spectra for compound 3n

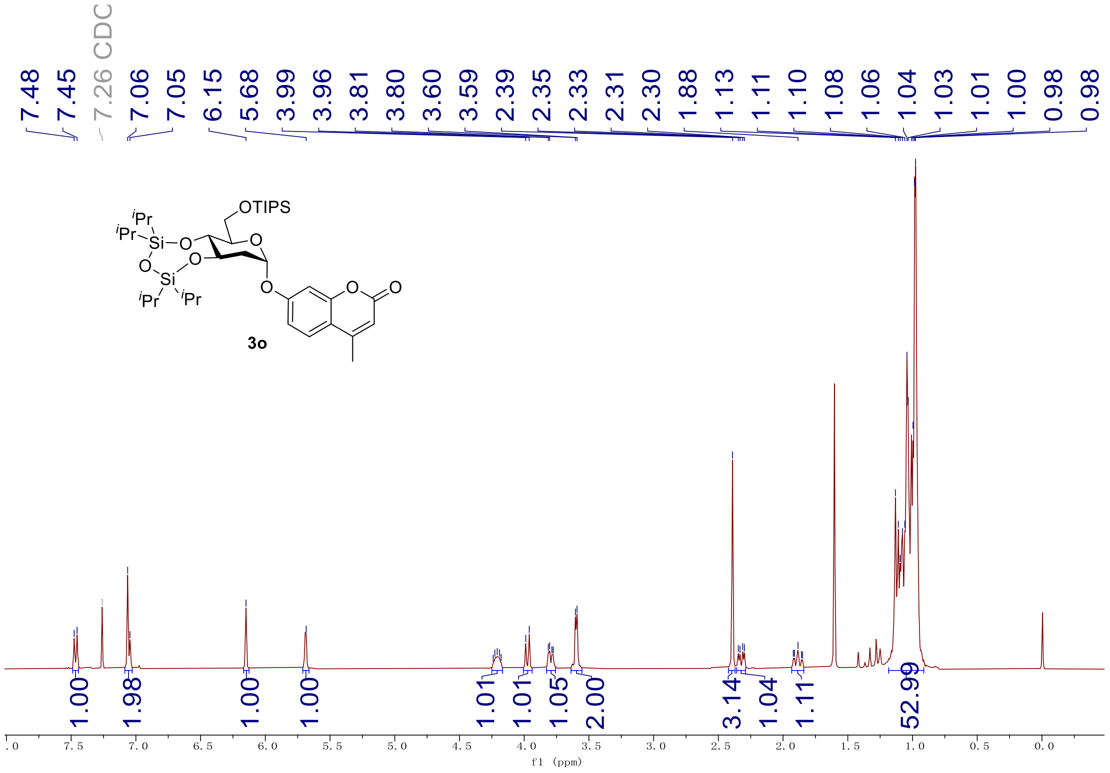

Figure S59. ^{1}H NMR (400 MHz, $CDCl_3$) Spectra for compound 3o

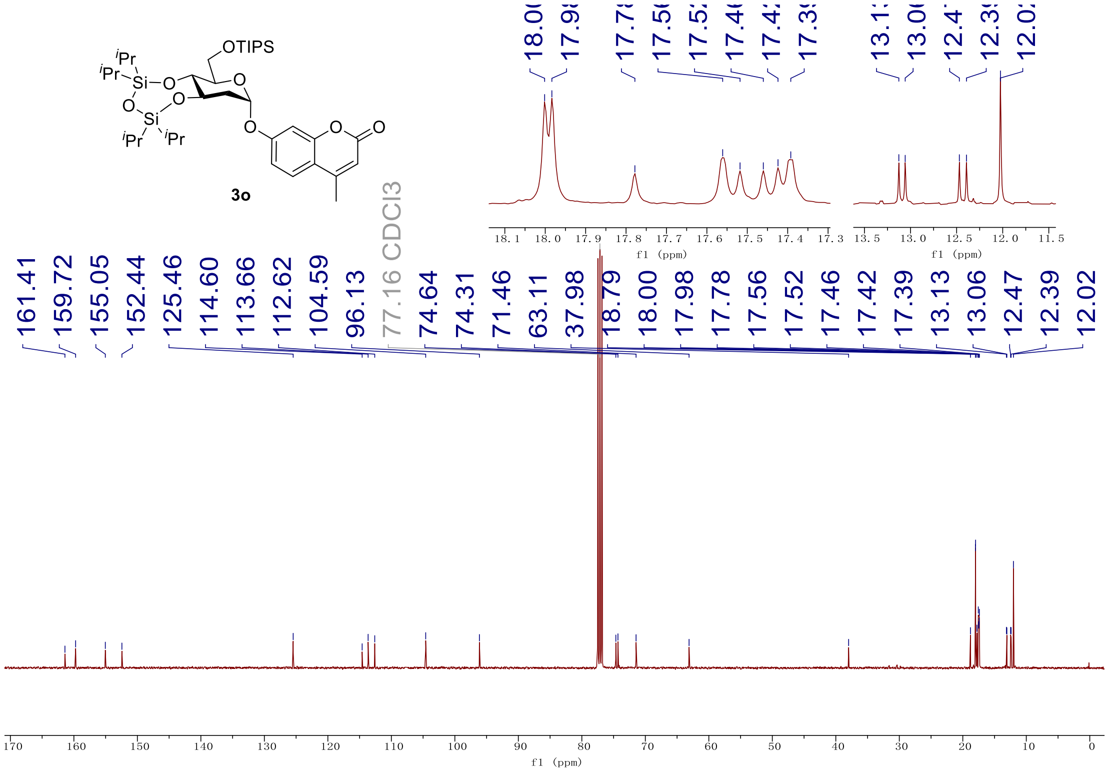

Figure S60. ^{13}C NMR (101 MHz, $CDCl_3$) Spectra for compound 3o

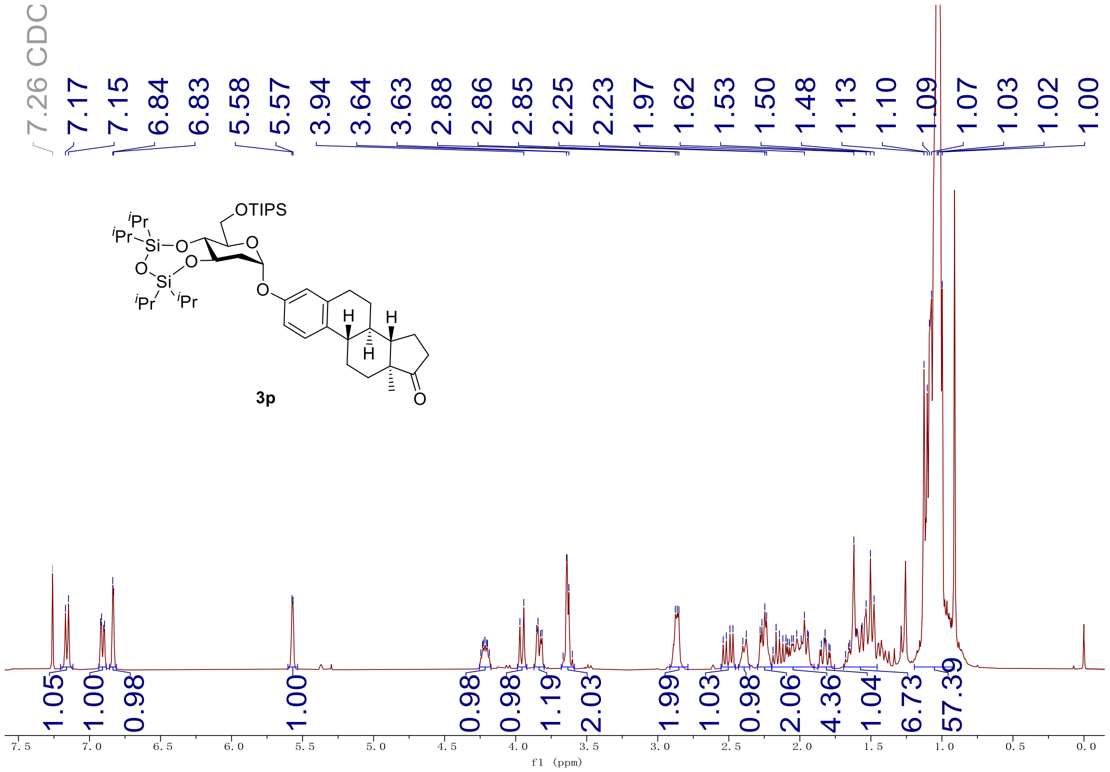

Figure S61. ^{1}H NMR (400 MHz, $CDCl_3$) Spectra for compound 3p

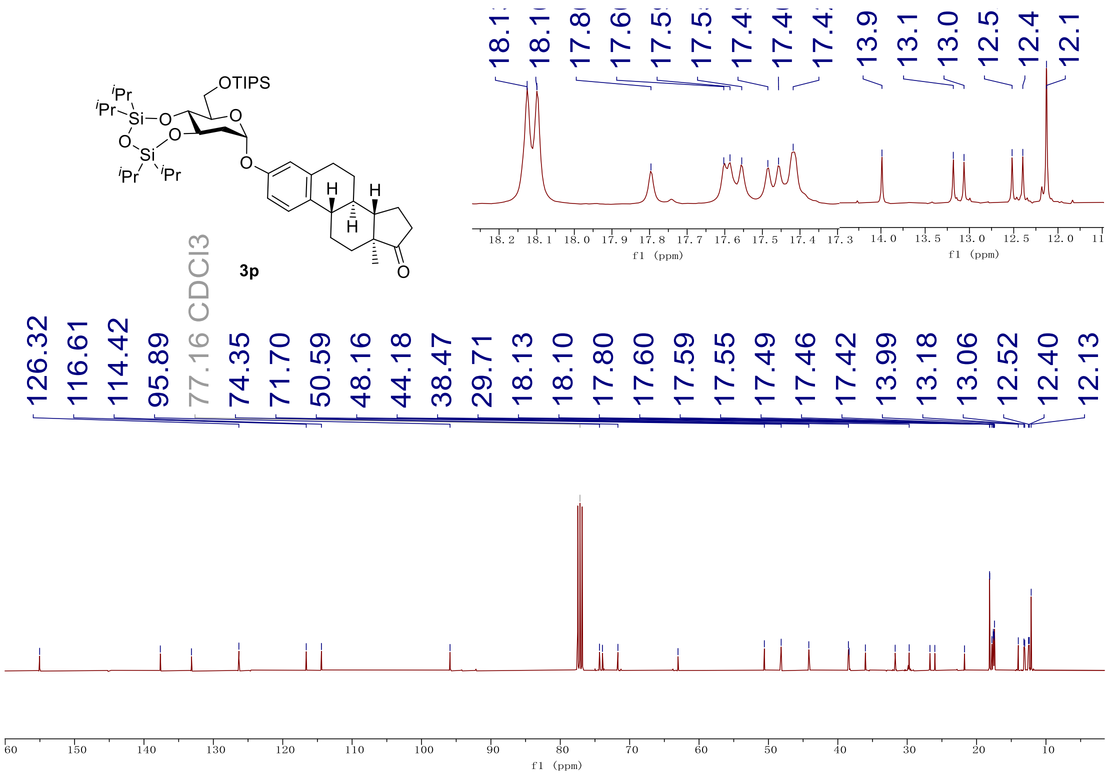

Figure S62. ^{13}C NMR (101 MHz, $CDCl_3$) Spectra for compound 3p

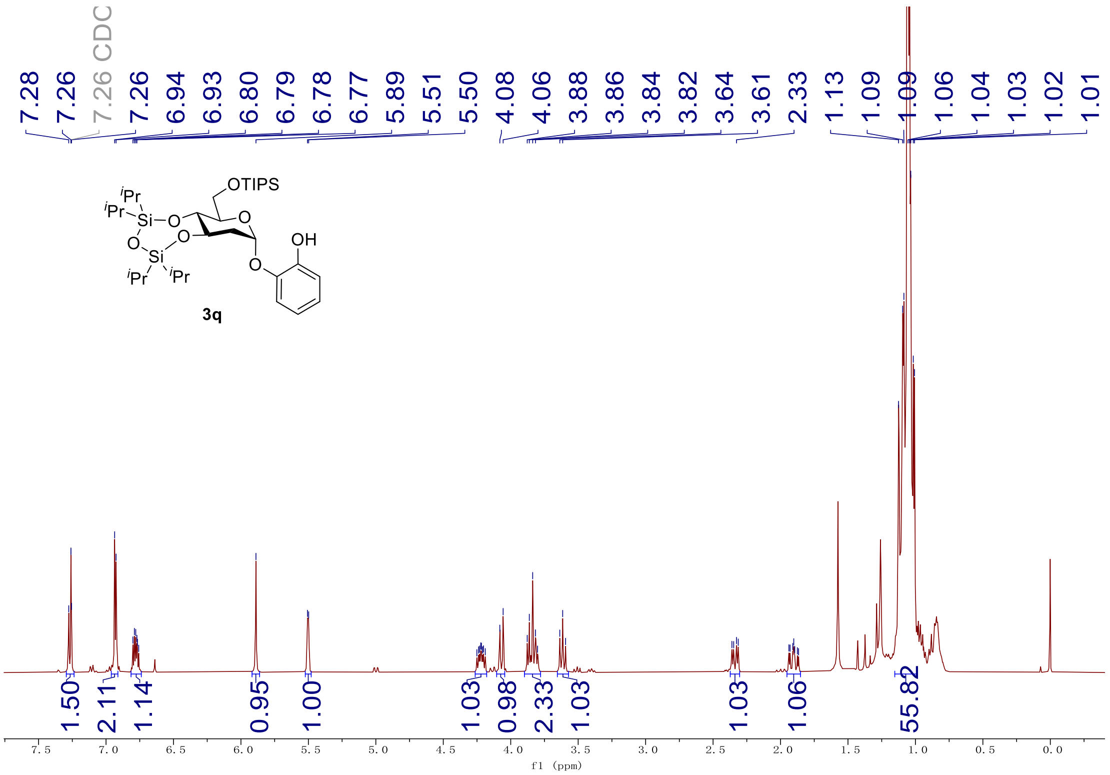

Figure S63. ^{1}H NMR (400 MHz, $CDCl_3$) Spectra for compound 3q

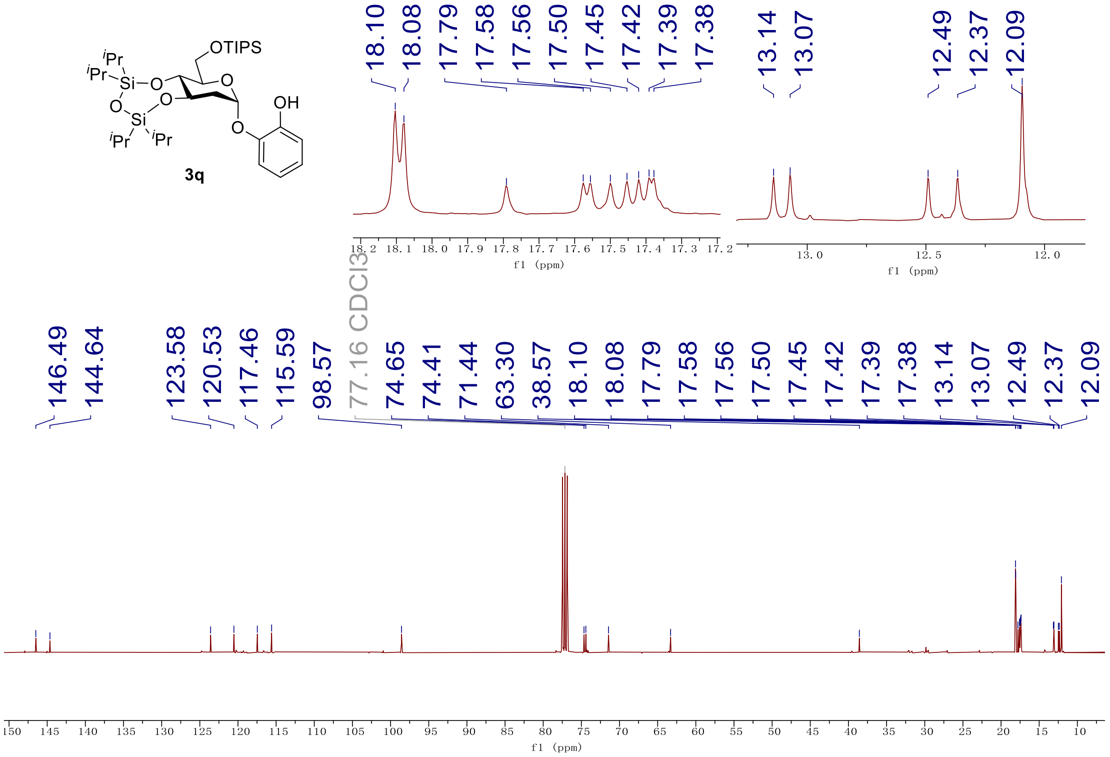

Figure S64. ^{13}C NMR (101 MHz, CDCl$_3$) Spectra for compound 3q

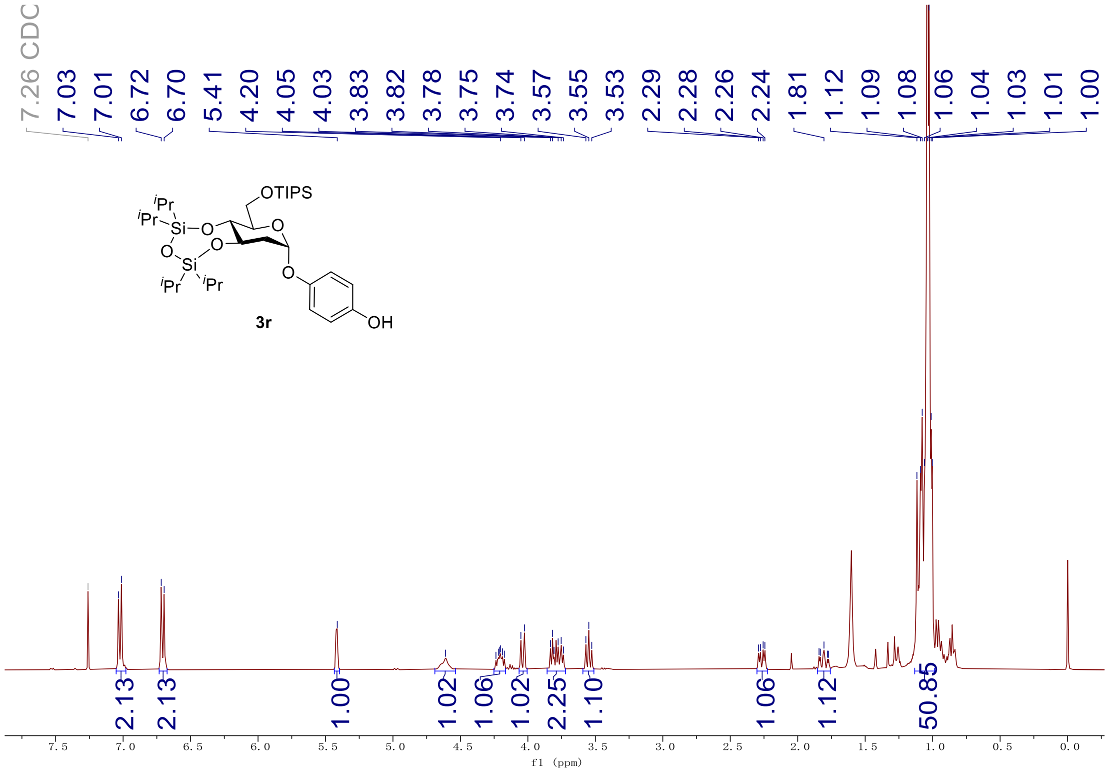

Figure S65. ^{1}H NMR (400 MHz, CDCl$_3$) Spectra for compound 3r

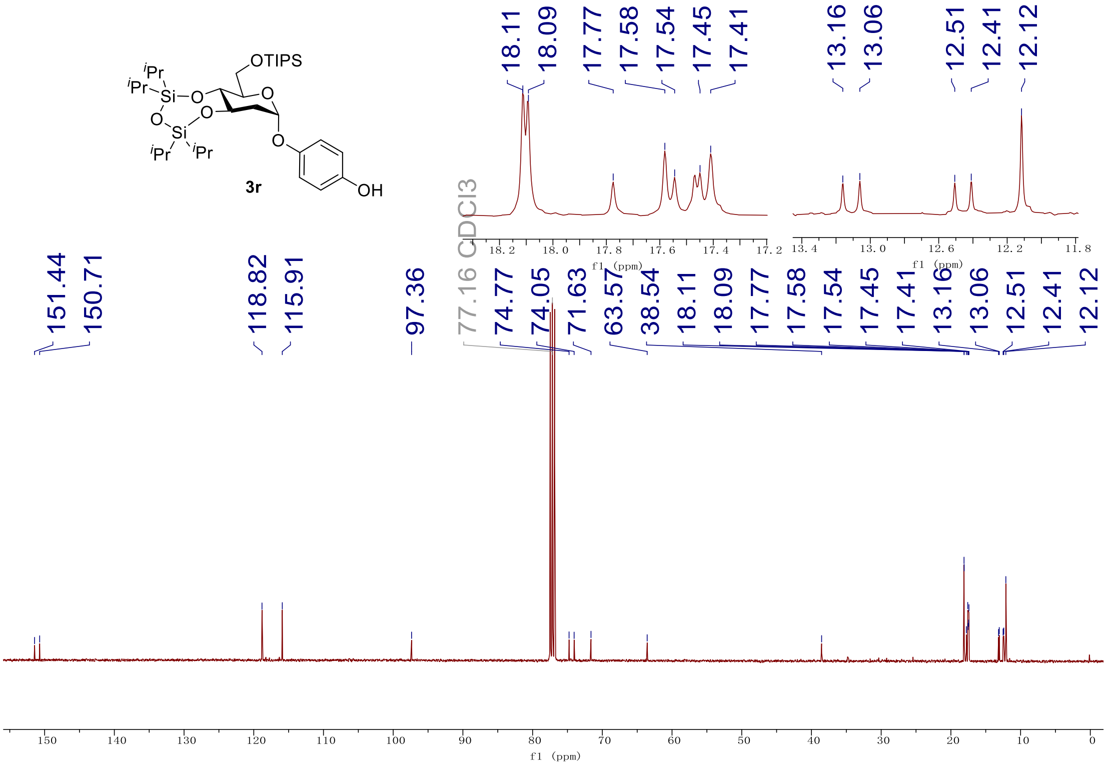

Figure S66. ^{13}**C NMR (101 MHz, CDCl$_3$) Spectra for compound 3r**

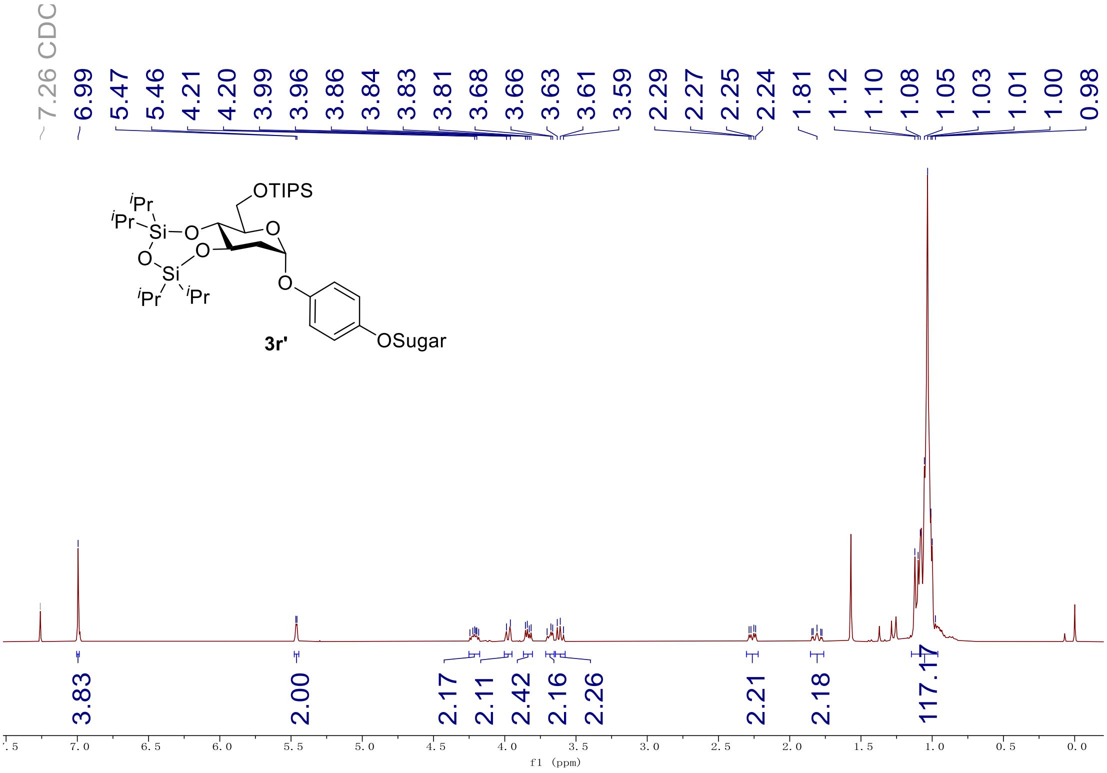

Figure S67. ^{1}**H NMR (400 MHz, CDCl$_3$) Spectra for compound 3r'**

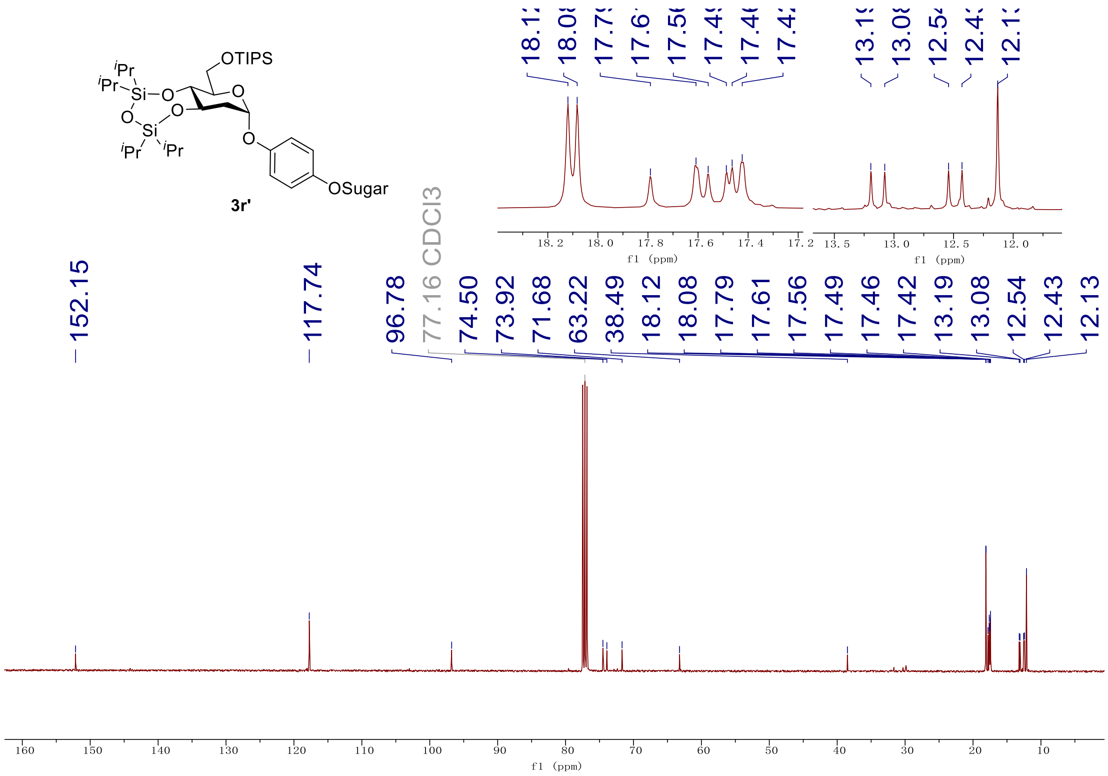

Figure S68. ^{13}C **NMR (101 MHz,** $CDCl_3$**) Spectra for compound 3r'**

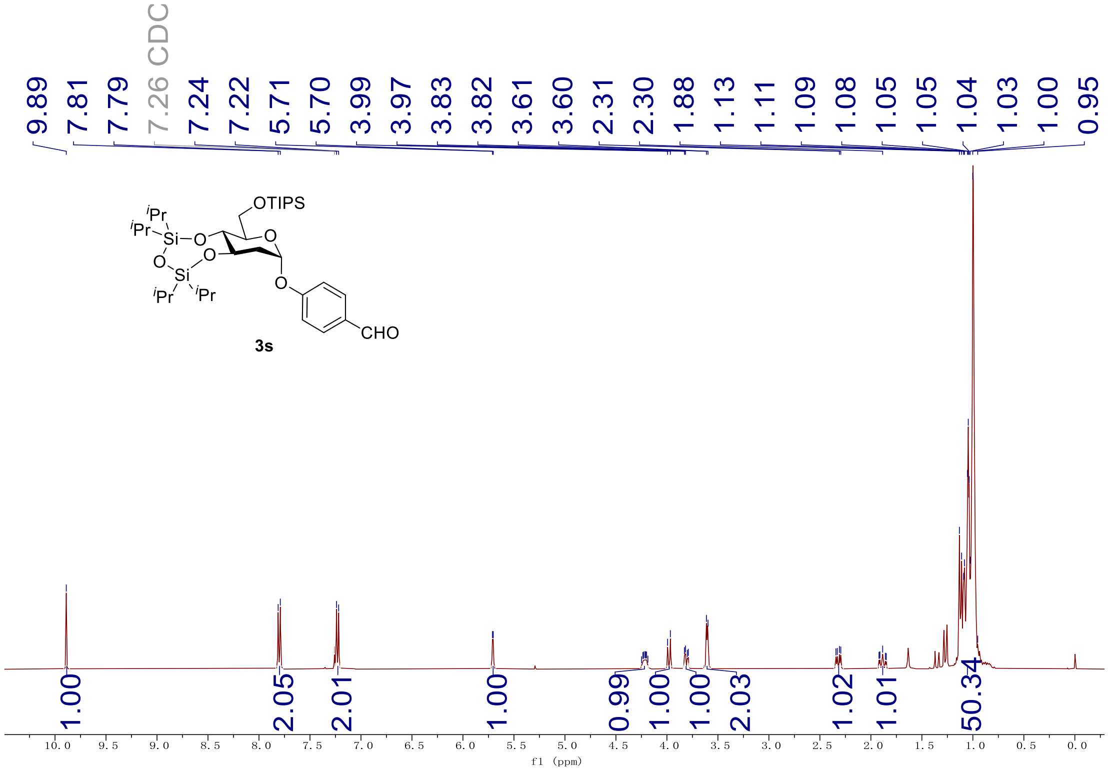

Figure S69. ^{1}H **NMR (400 MHz,** $CDCl_3$**) Spectra for compound 3s**

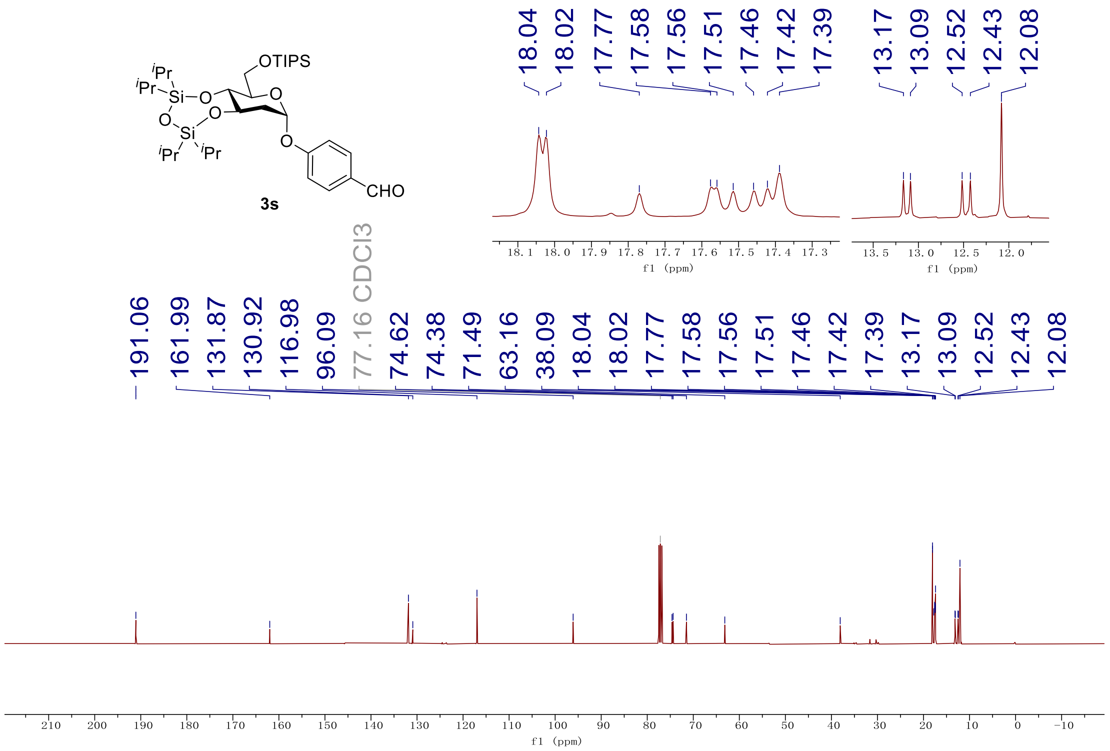

Figure S70. ^{13}C NMR (101 MHz, CDCl$_3$) Spectra for compound 3s

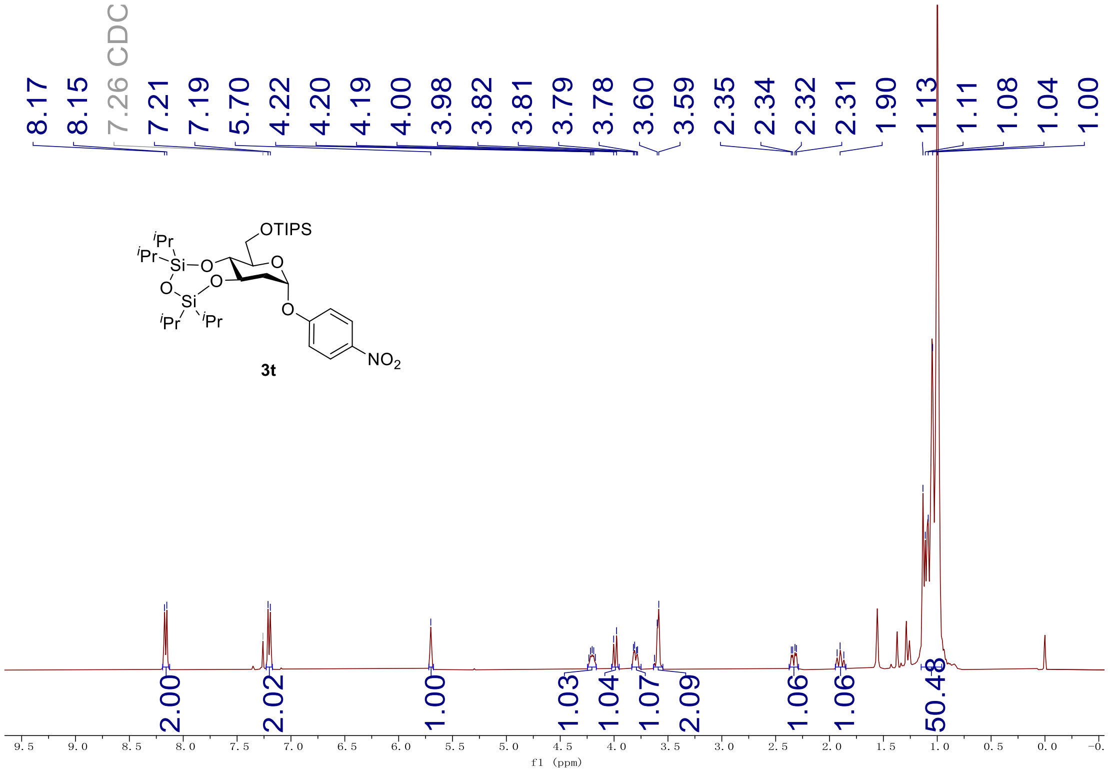

Figure S71. ^{1}H NMR (400 MHz, CDCl$_3$) Spectra for compound 3t

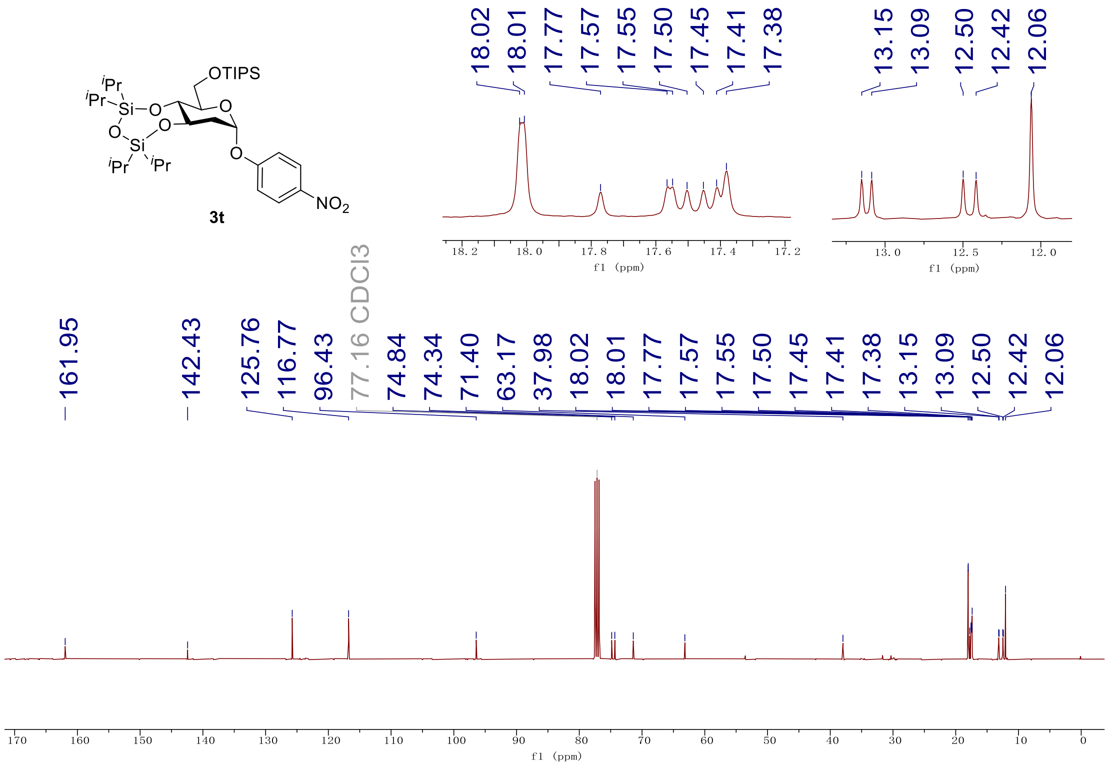

Figure S72. ^{13}C NMR (101 MHz, $CDCl_3$) Spectra for compound 3t

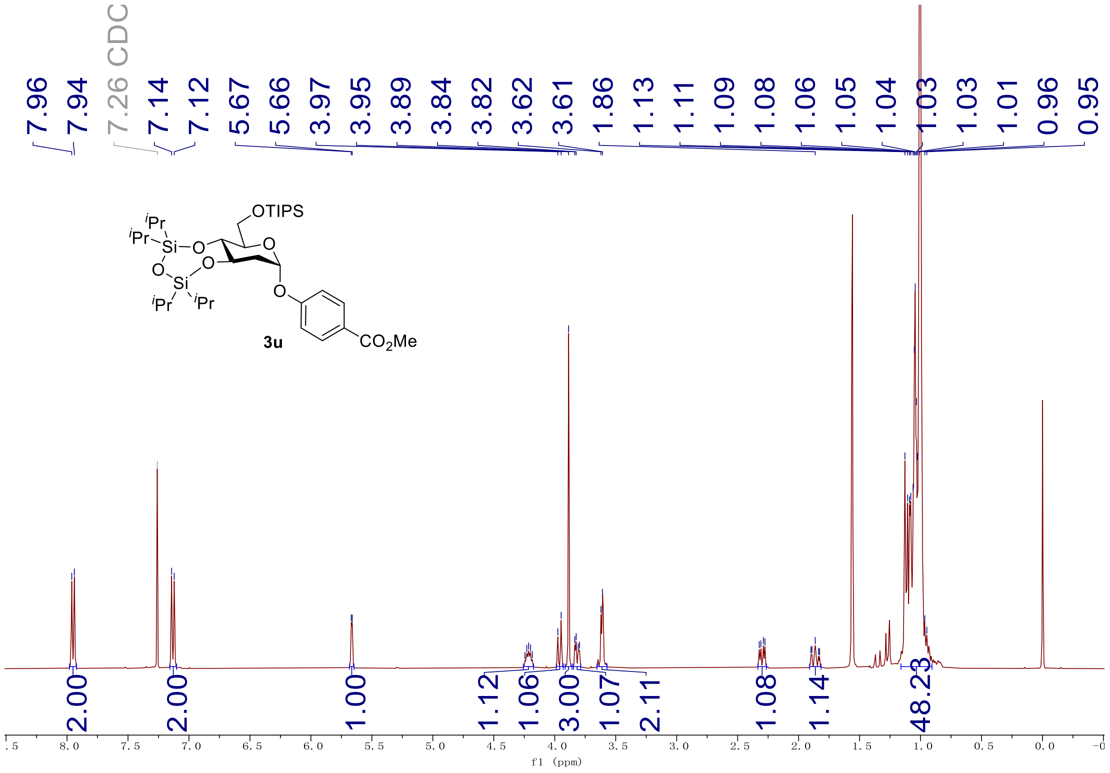

Figure S73. ^{1}H NMR (400 MHz, $CDCl_3$) Spectra for compound 3u

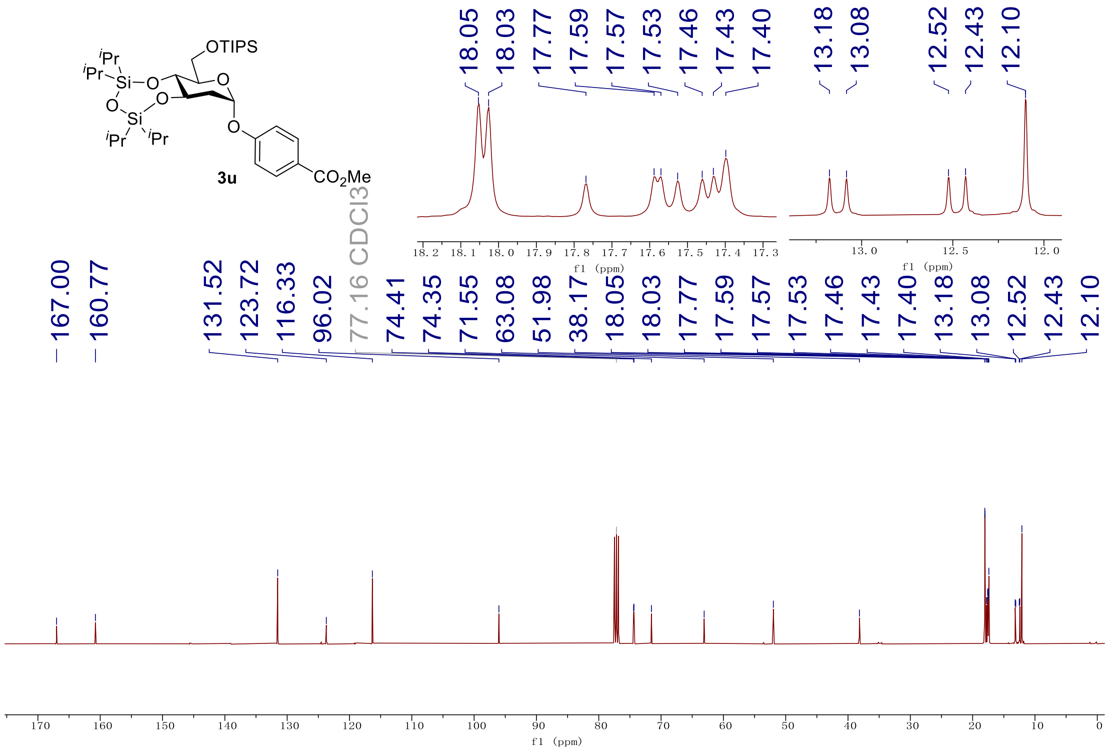

Figure S74. ^{13}C NMR (101 MHz, CDCl$_3$) Spectra for compound 3u

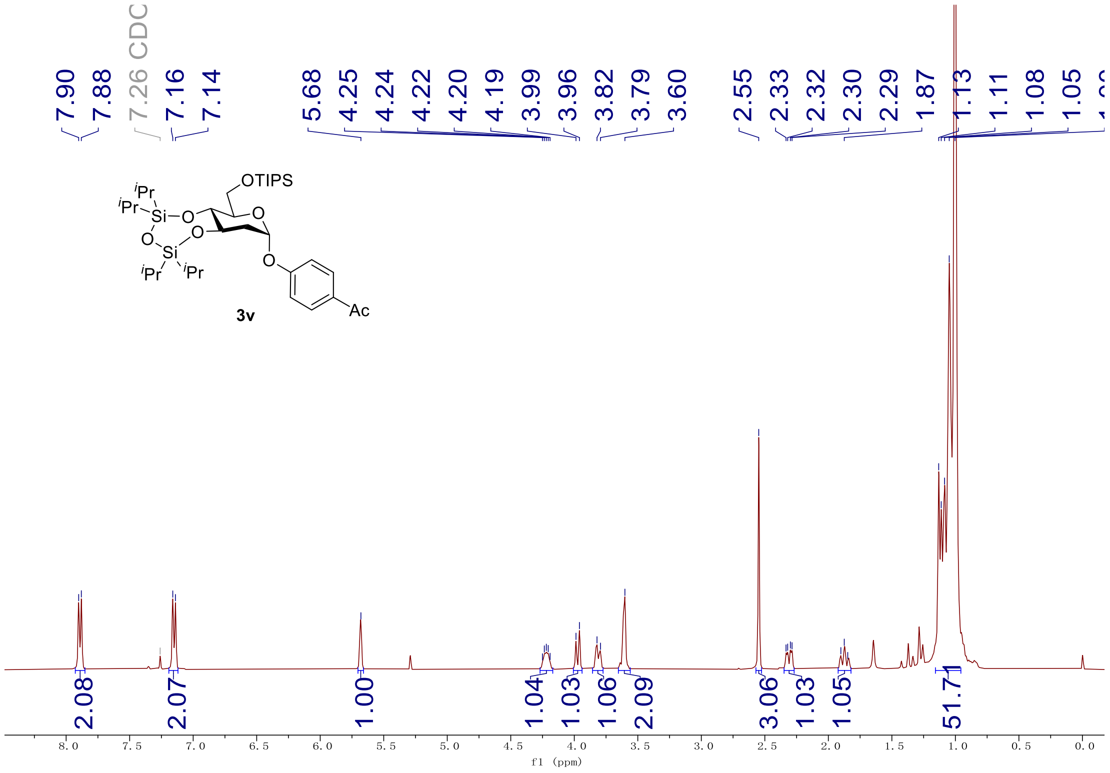

Figure S75. ^{1}H NMR (400 MHz, CDCl$_3$) Spectra for compound 3v

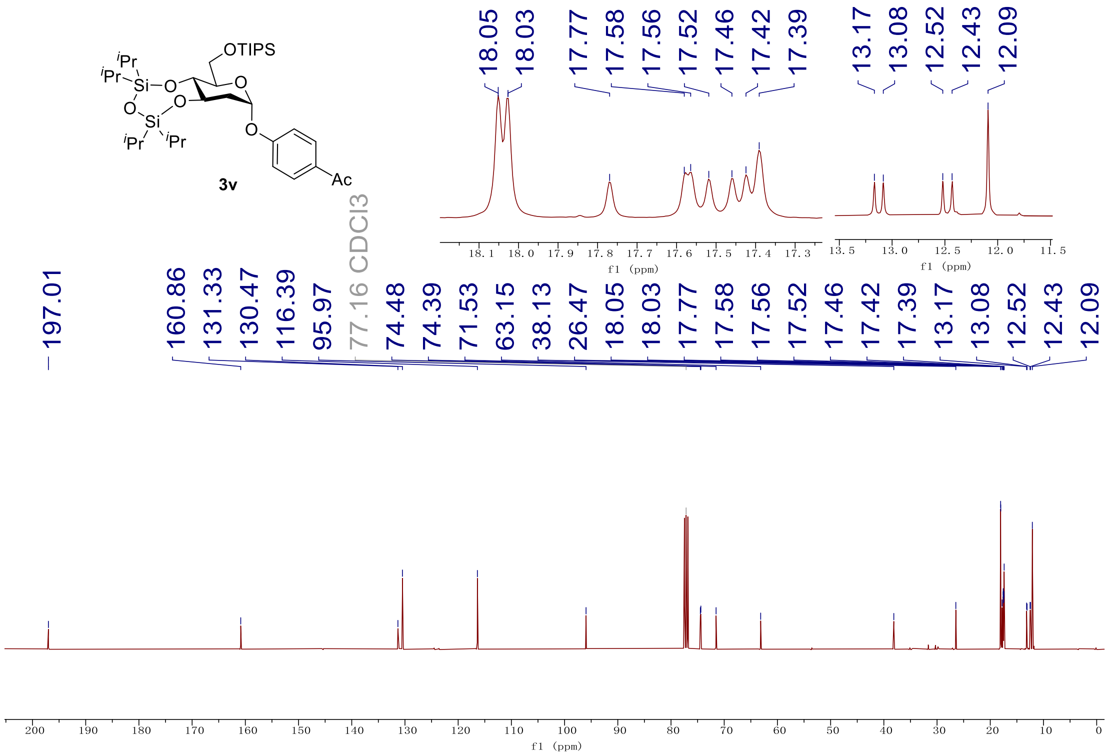

Figure S76. ^{13}C NMR (101 MHz, CDCl$_3$) Spectra for compound 3v

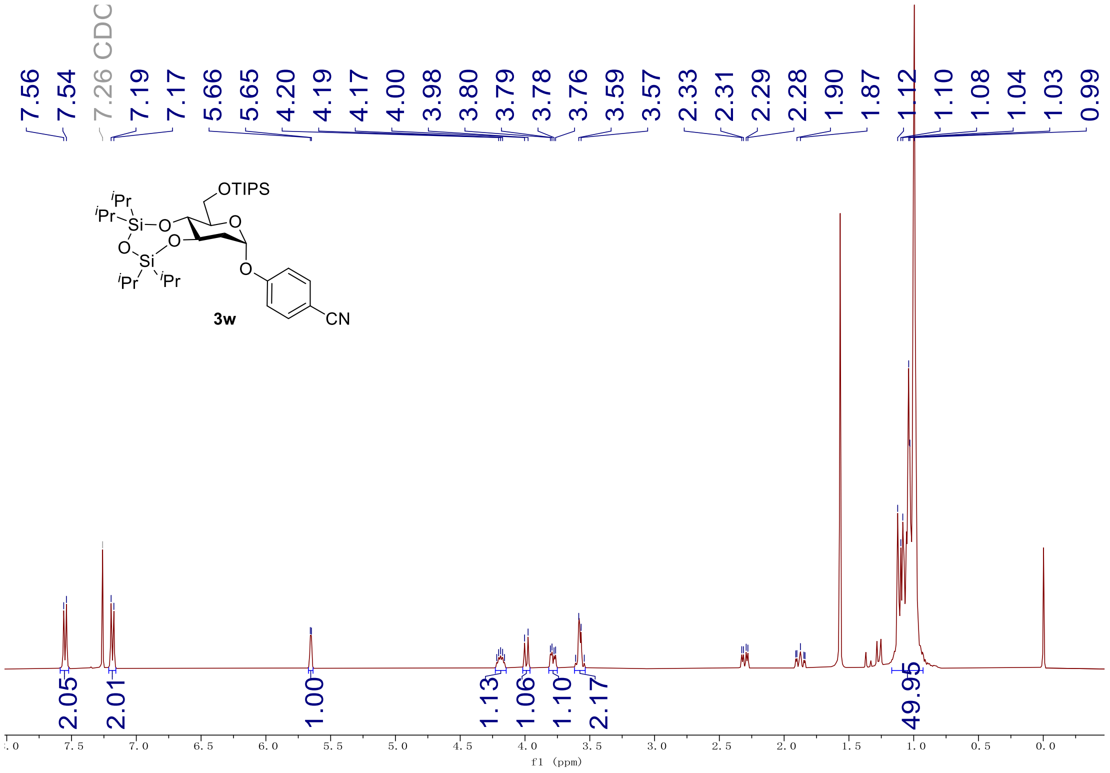

Figure S77. ^{1}H NMR (400 MHz, CDCl$_3$) Spectra for compound 3w

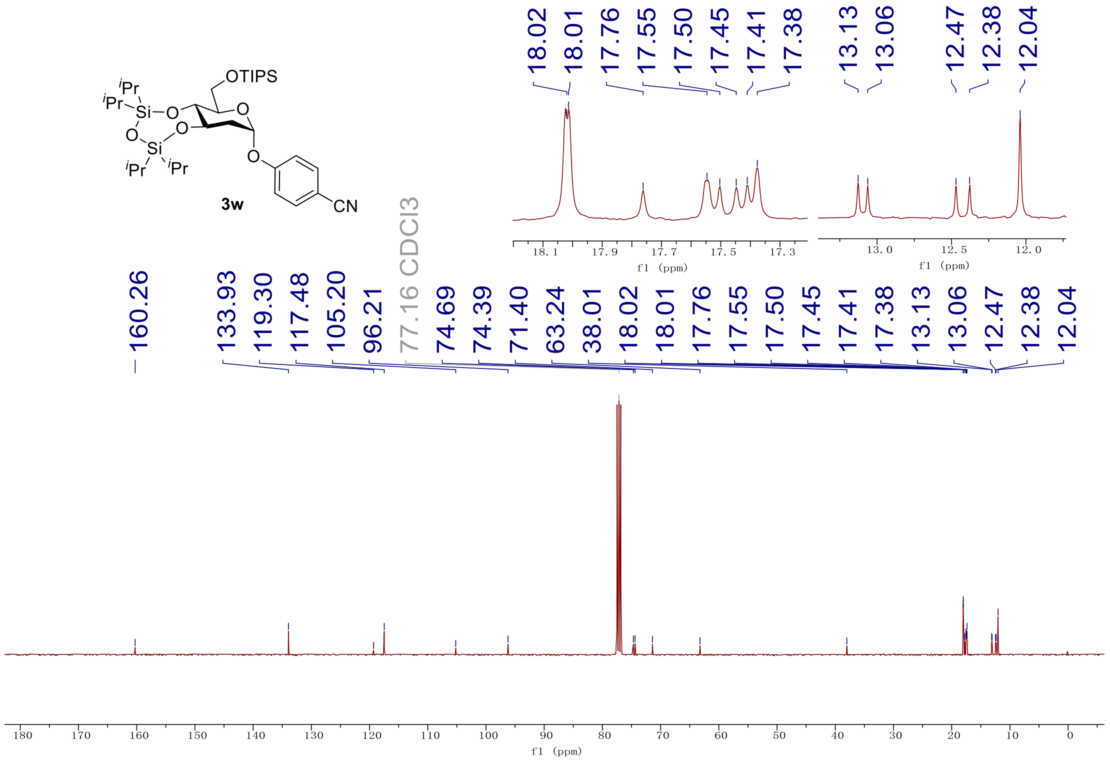

Figure S78. ^{13}C NMR (101 MHz, $CDCl_3$) Spectra for compound 3w

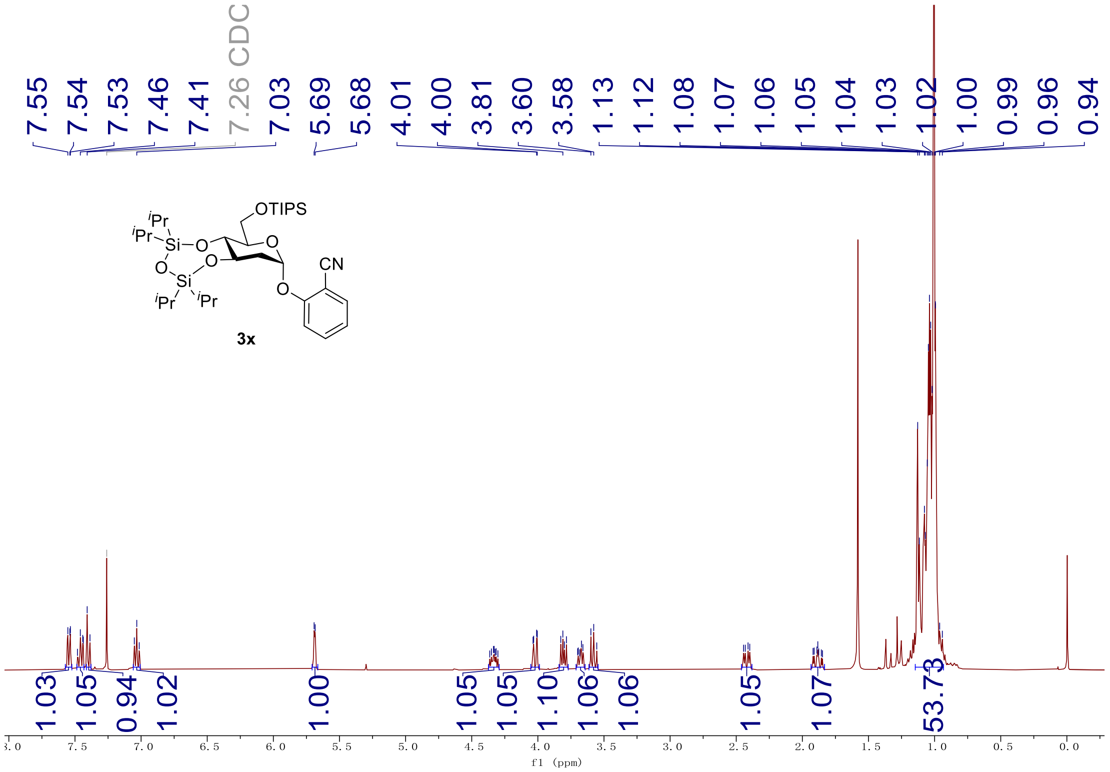

Figure S79. ^{1}H NMR (400 MHz, $CDCl_3$) Spectra for compound 3x

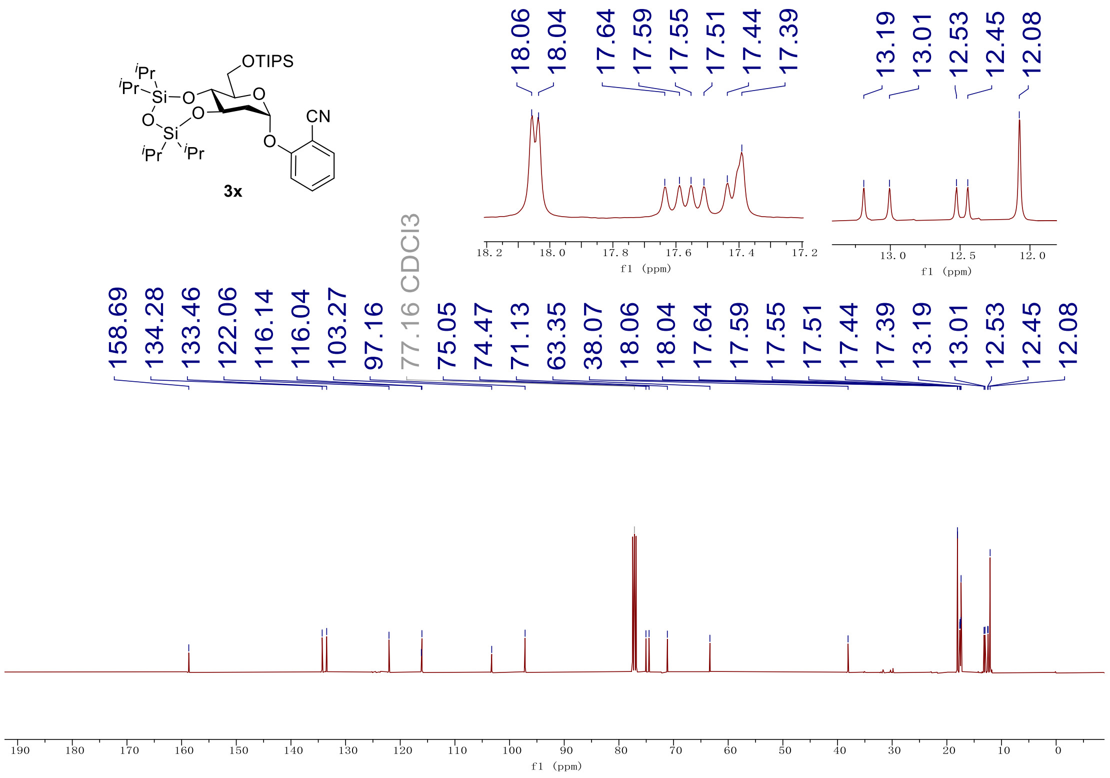

Figure S80. **^{13}C NMR (101 MHz, CDCl₃) Spectra for compound 3x**

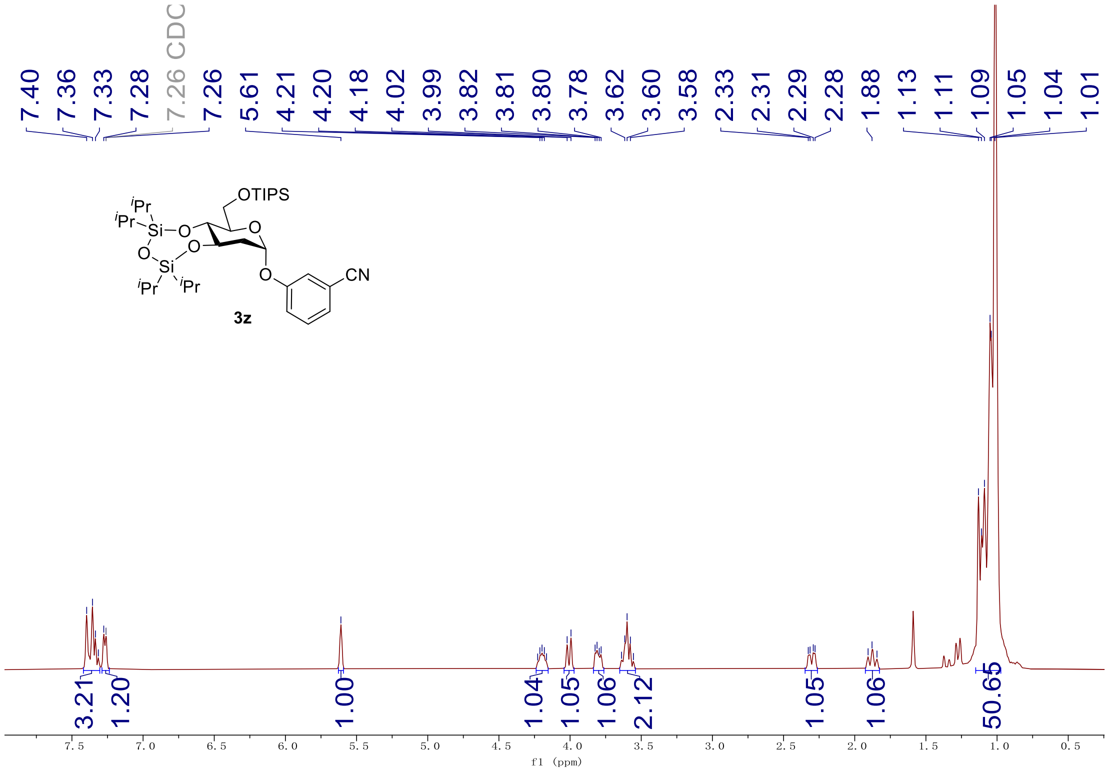

Figure S81. **^{1}H NMR (400 MHz, CDCl₃) Spectra for compound 3z**

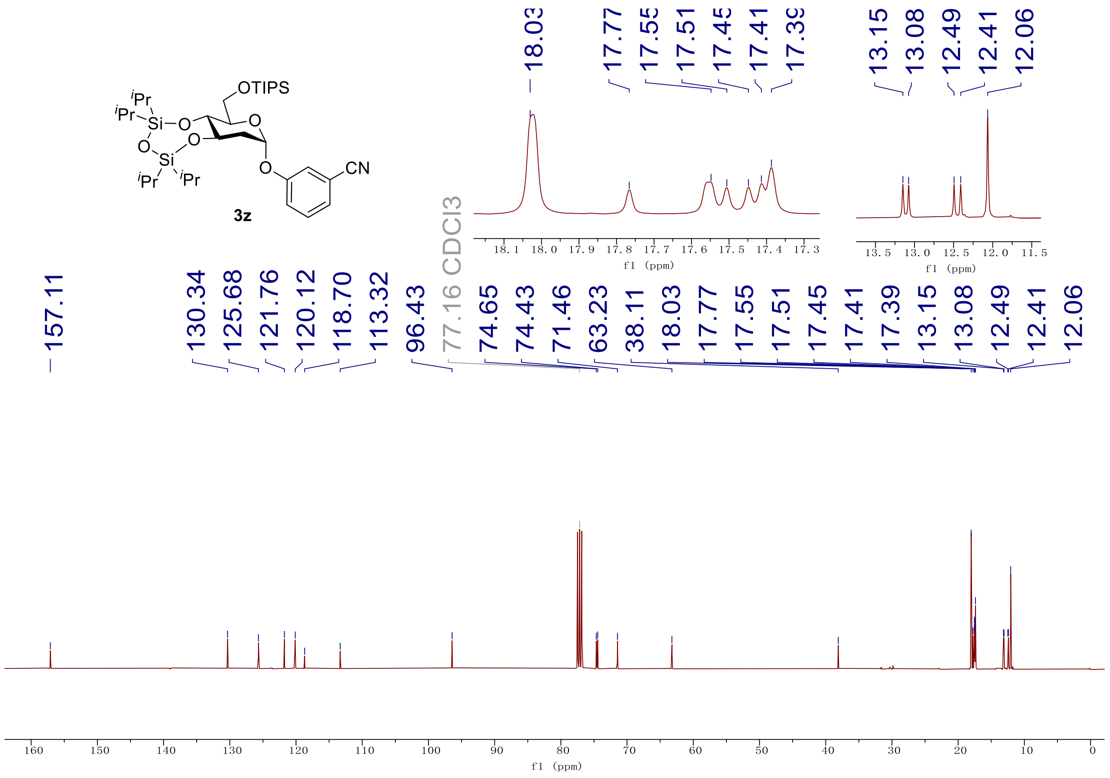

Figure S82. ^{13}C NMR (101 MHz, $CDCl_3$) Spectra for compound 3z

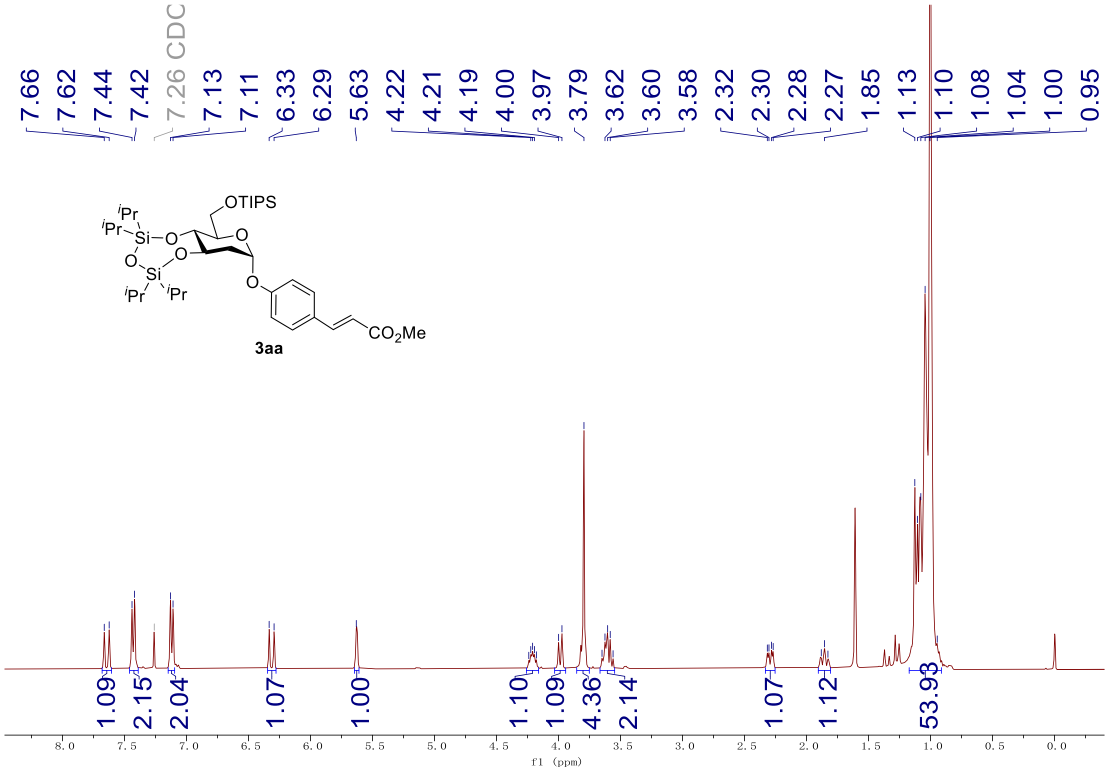

Figure S83. ^{1}H NMR (400 MHz, $CDCl_3$) Spectra for compound 3aa

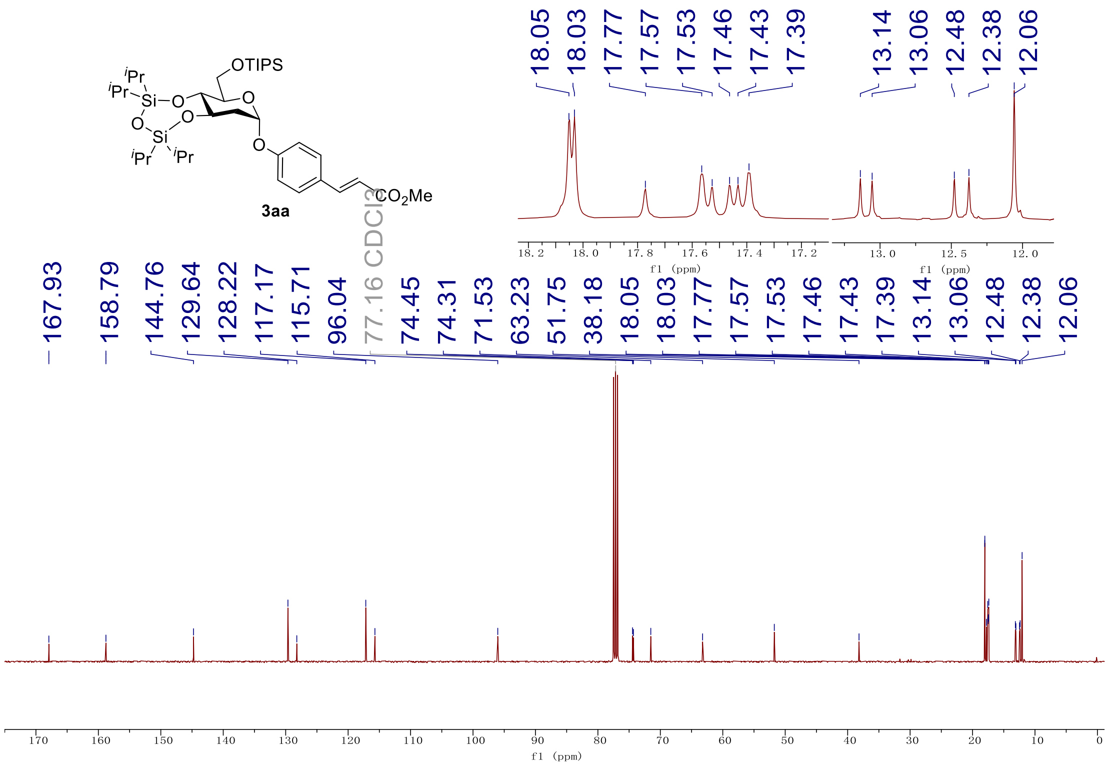

Figure S84. ^{13}C **NMR (101 MHz, $CDCl_3$) Spectra for compound 3aa**

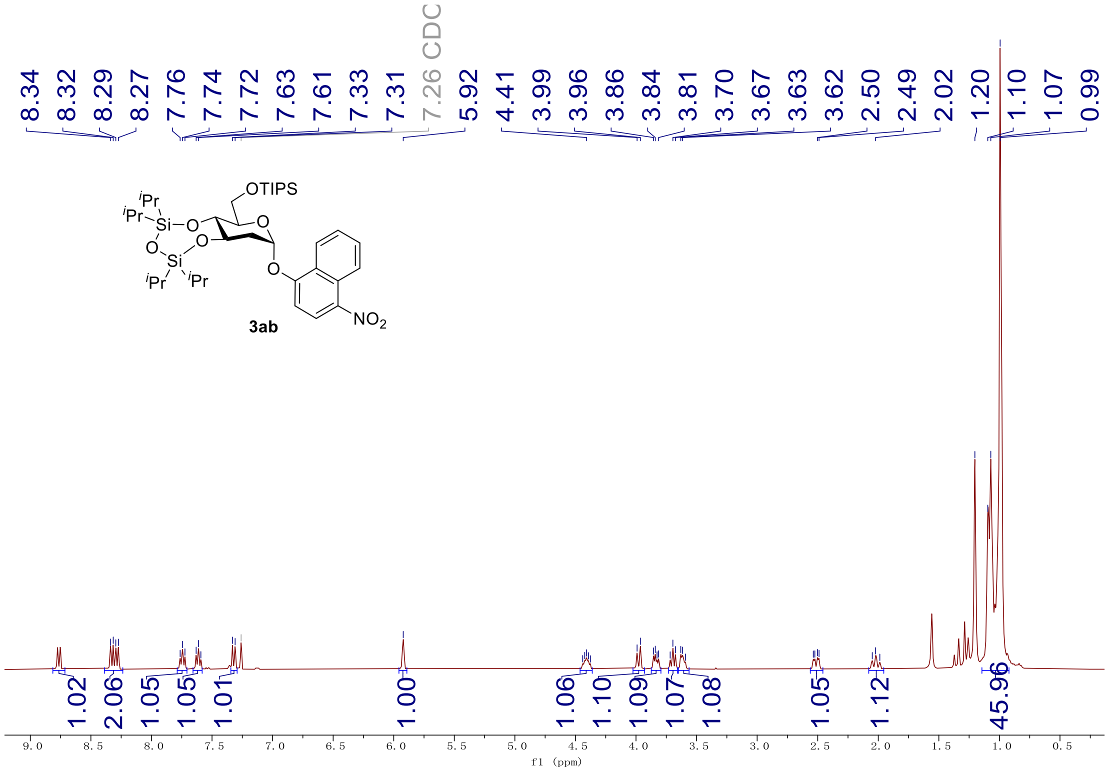

Figure S85. ^{1}H **NMR (400 MHz, $CDCl_3$) Spectra for compound 3ab**

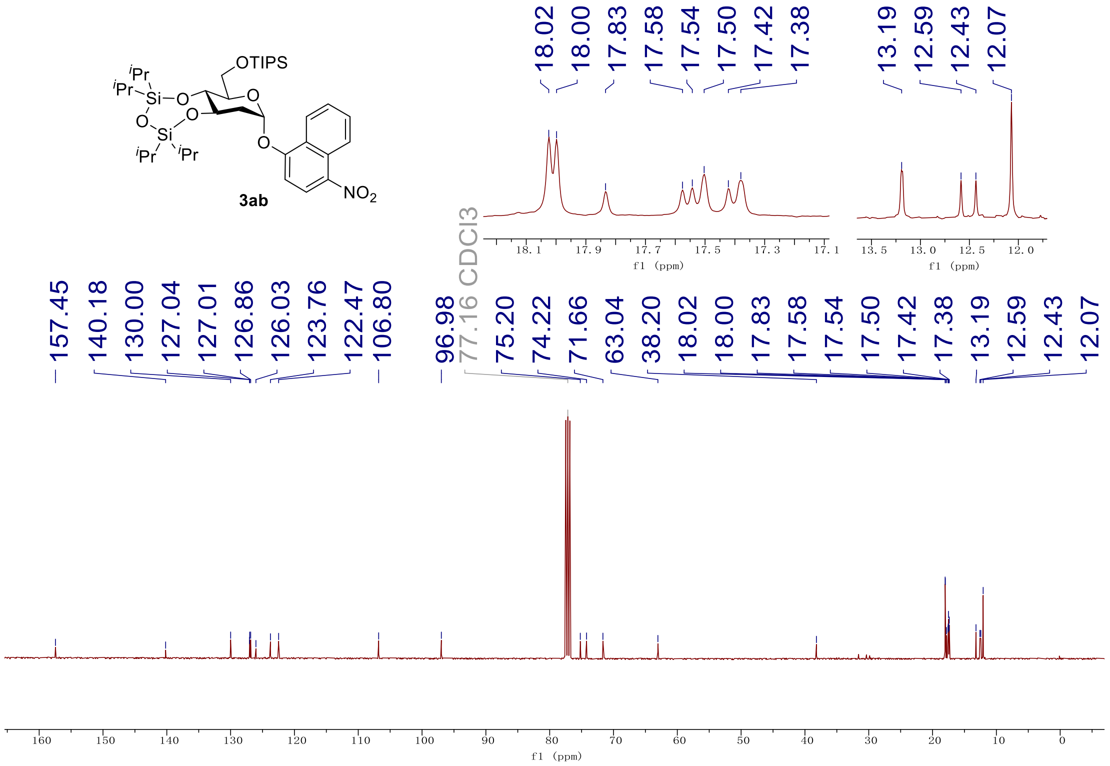

Figure S86. ^{13}**C NMR (101 MHz, CDCl$_3$) Spectra for compound 3ab**

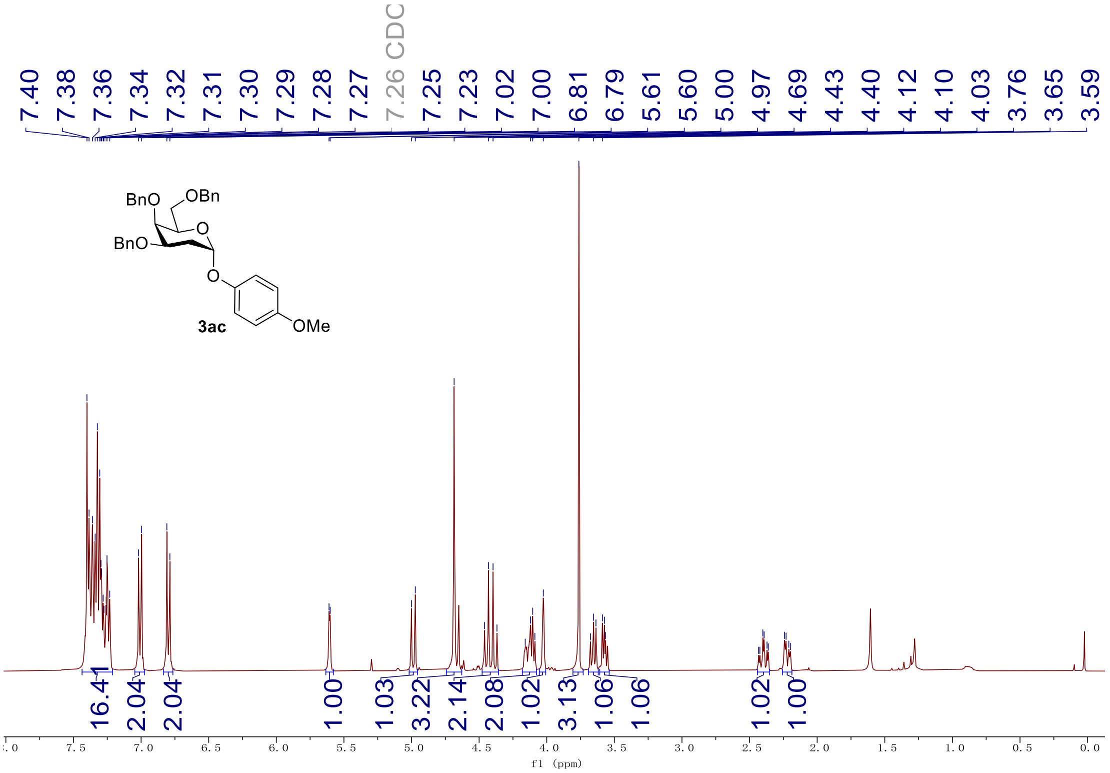

Figure S87. ^{1}**H NMR (400 MHz, CDCl$_3$) Spectra for compound 3ac**

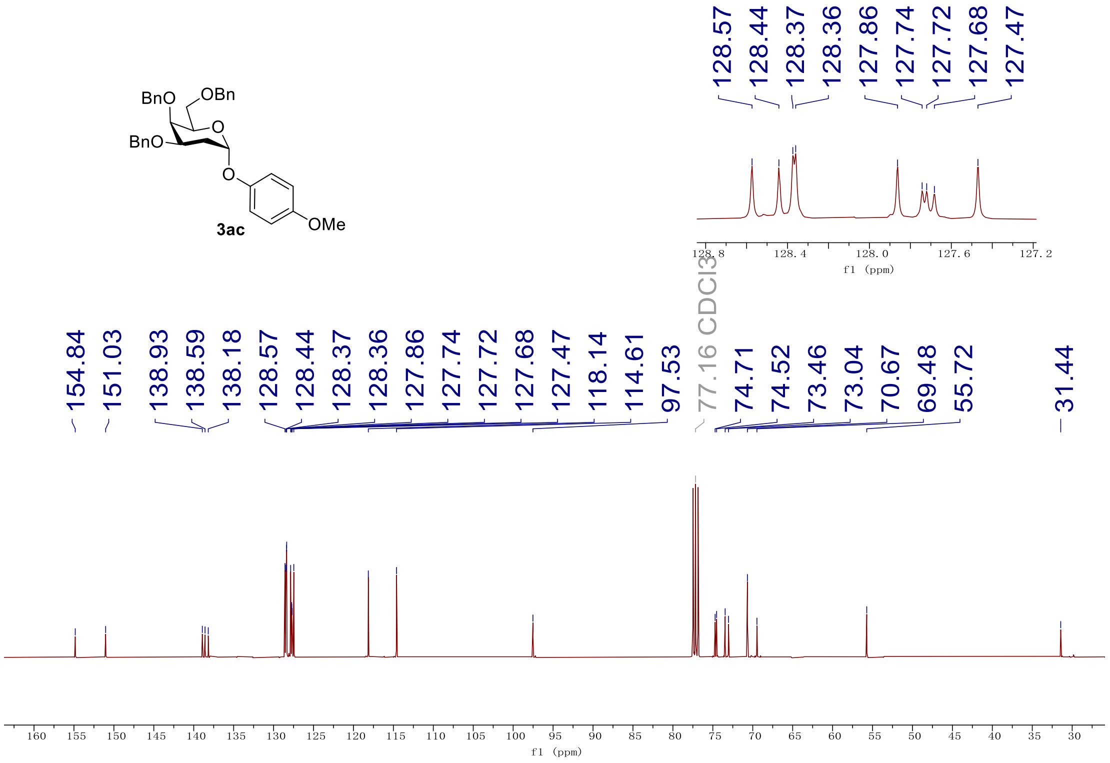

Figure S88. ^{13}C NMR (101 MHz, CDCl$_3$) Spectra for compound 3ac

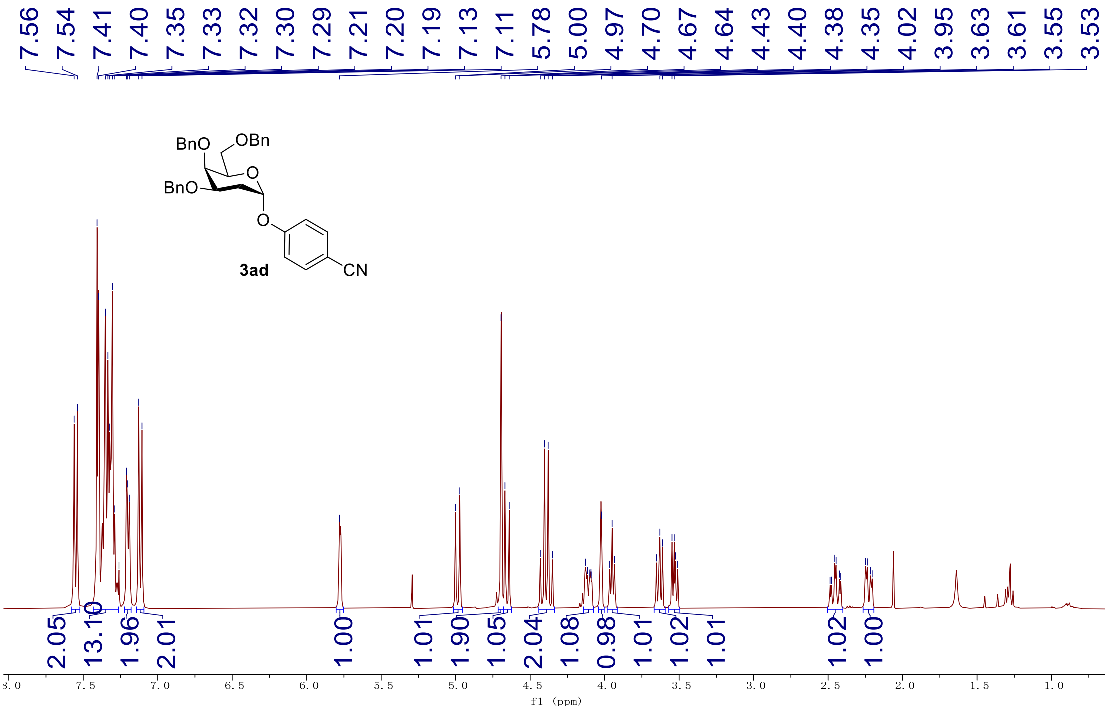

Figure S89. ^{1}H NMR (400 MHz, CDCl$_3$) Spectra for compound 3ad

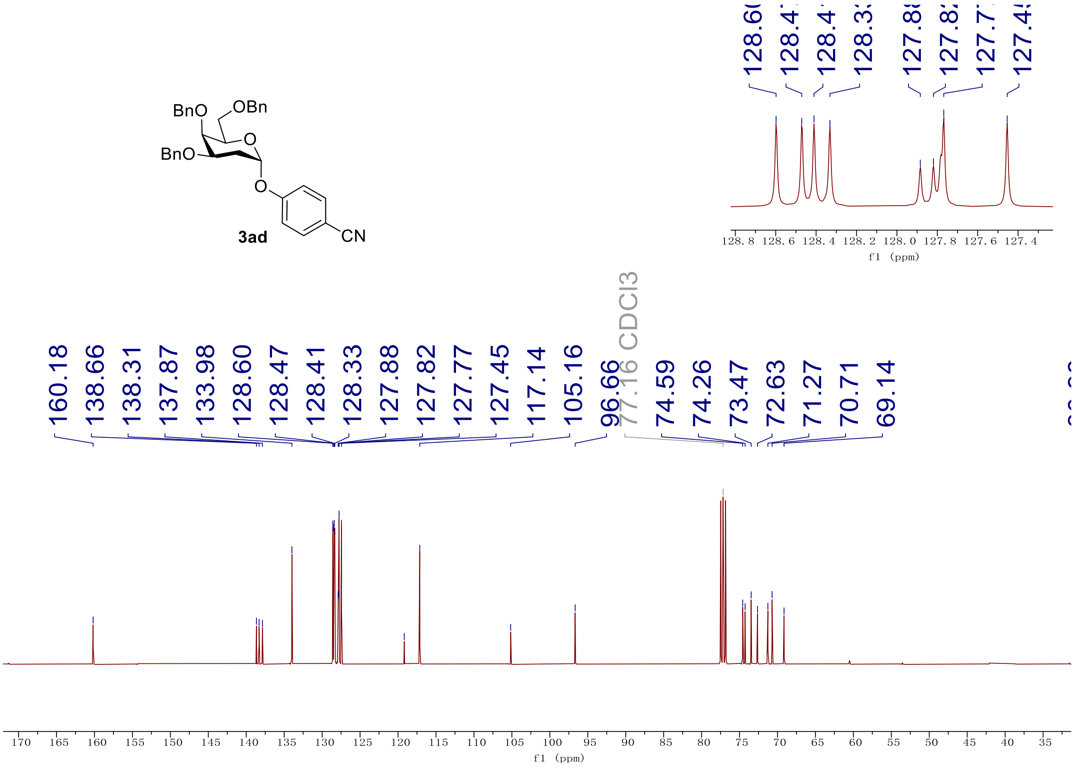

Figure S90. ^{13}C NMR (101 MHz, $CDCl_3$) Spectra for compound 3ad

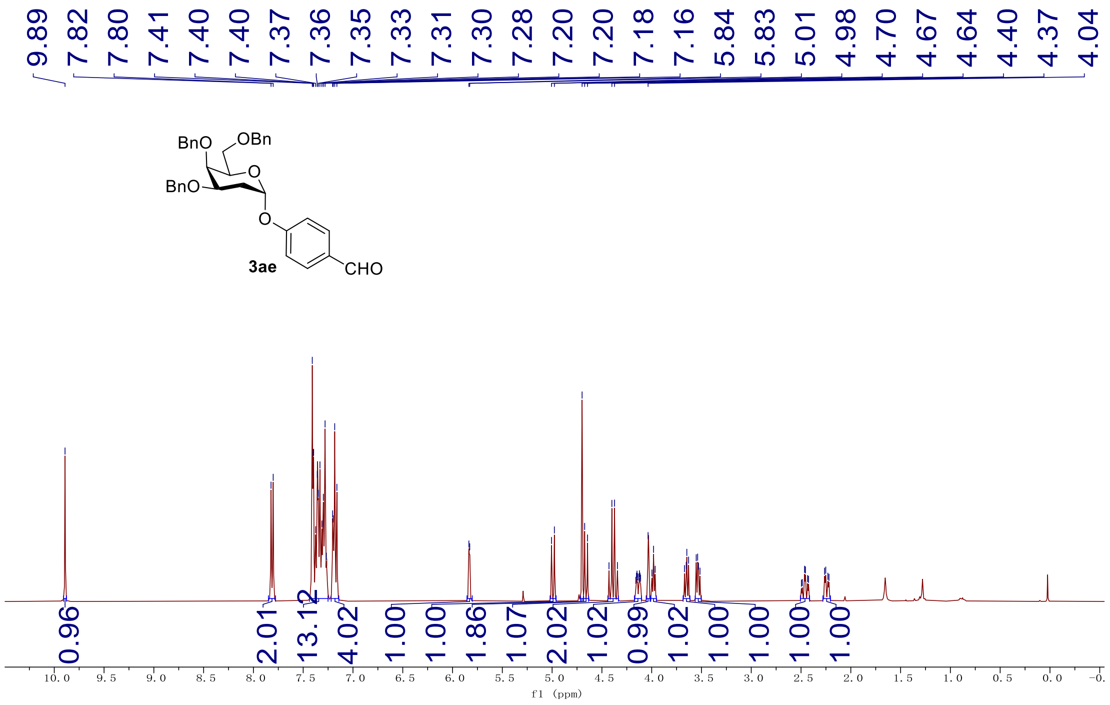

Figure S91. ^{1}H NMR (400 MHz, $CDCl_3$) Spectra for compound 3ae

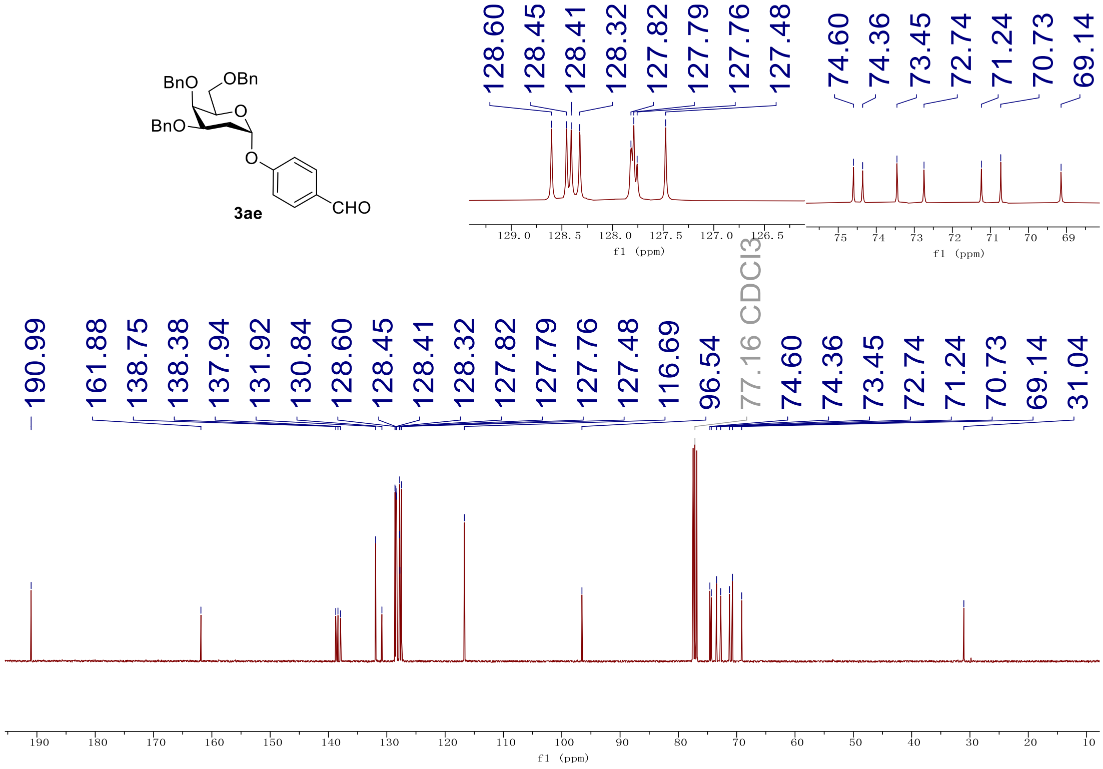

Figure S92. ^{13}C NMR (101 MHz, $CDCl_3$) Spectra for compound 3ae

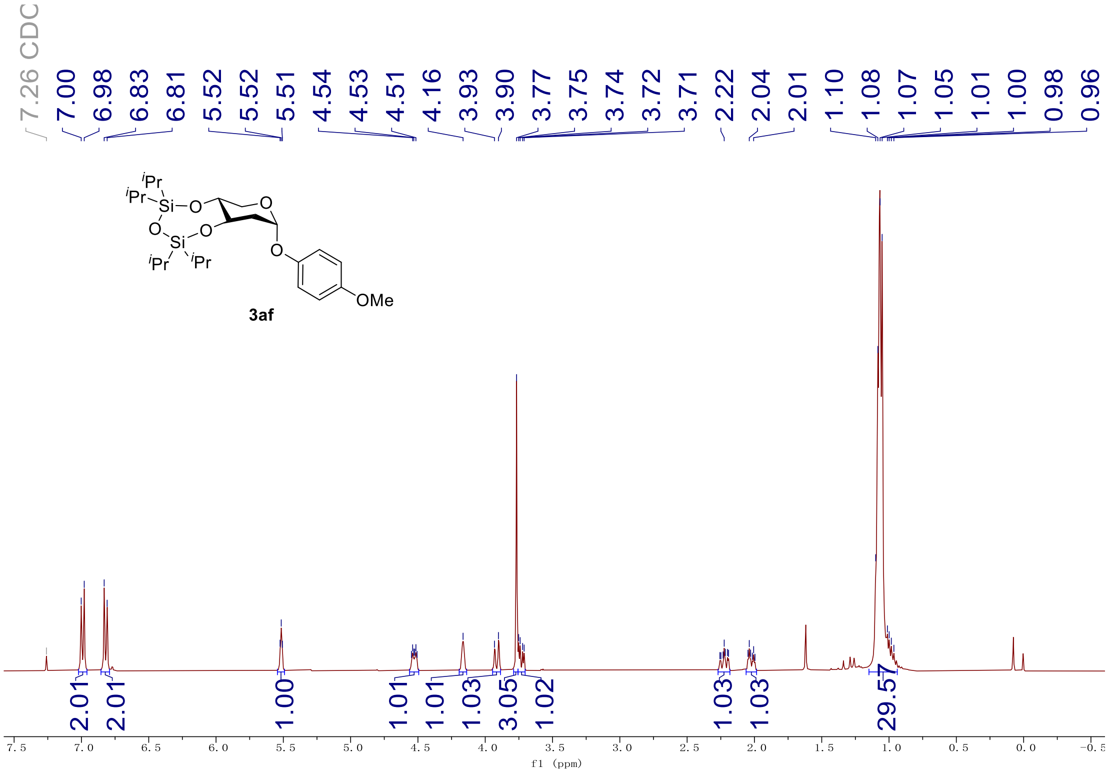

Figure S93. ^{1}H NMR (400 MHz, $CDCl_3$) Spectra for compound 3af

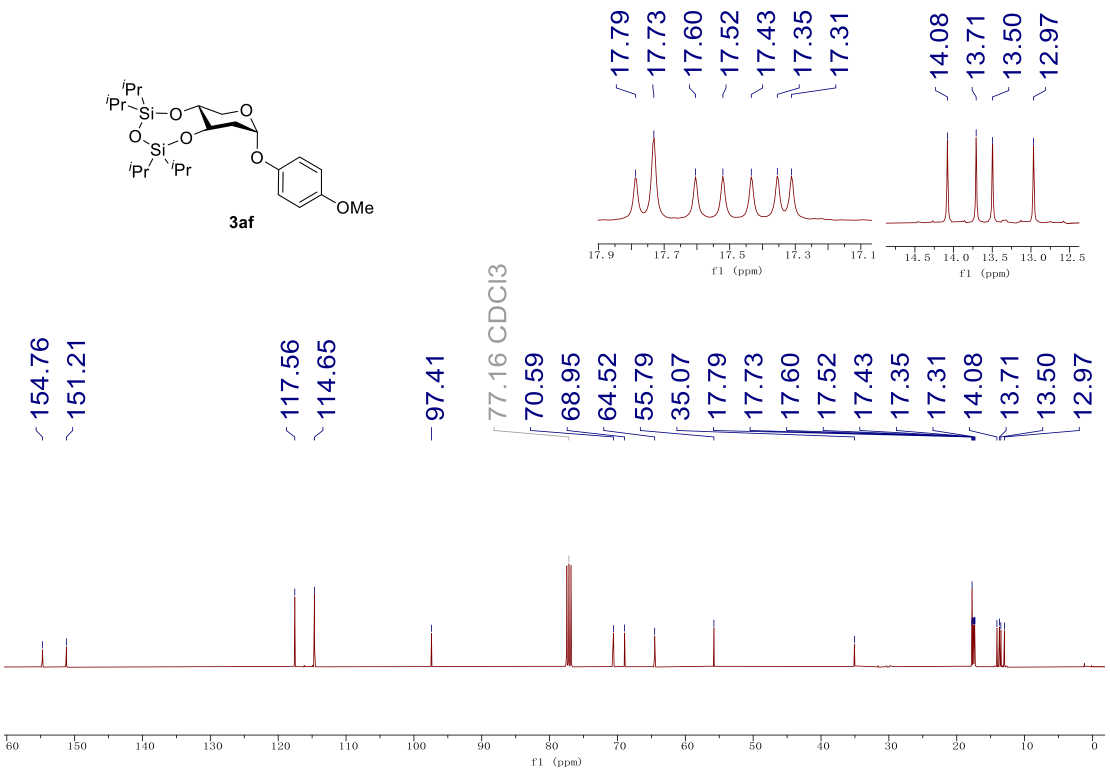

Figure S94. ^{13}C NMR (101 MHz, $CDCl_3$) Spectra for compound 3af

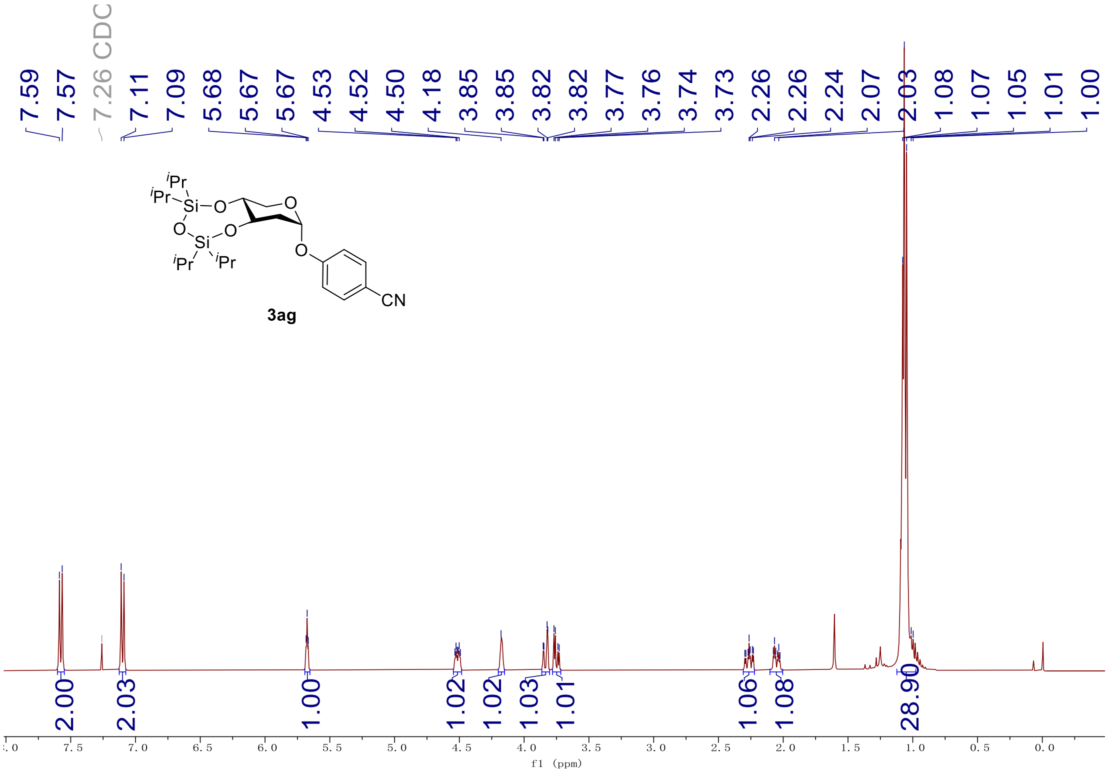

Figure S95. ^{1}H NMR (400 MHz, $CDCl_3$) Spectra for compound 3ag

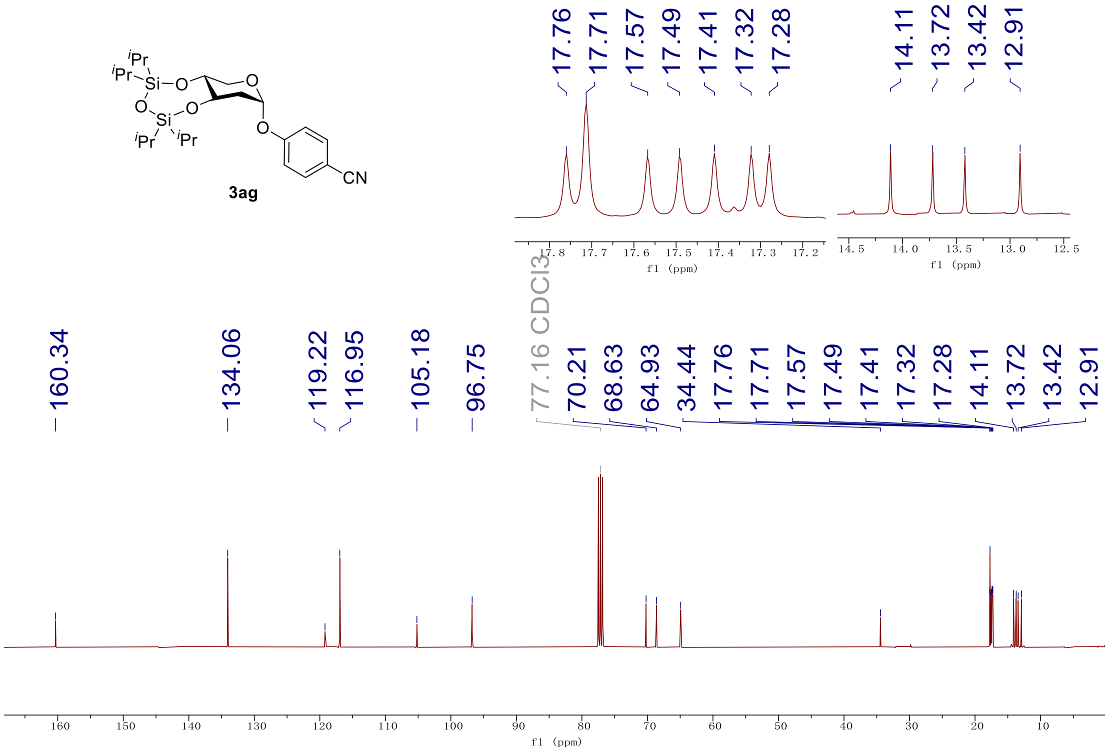

Figure S96. ^{13}C NMR (101 MHz, CDCl$_3$) Spectra for compound 3ag

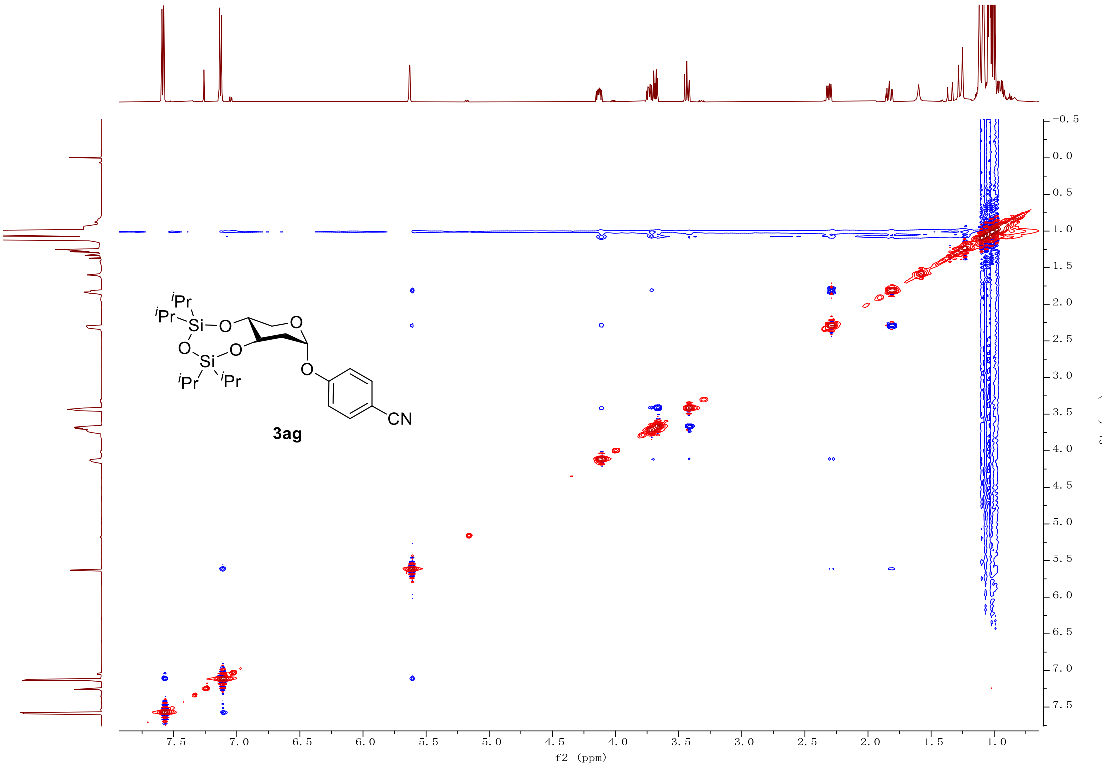

Figure S97: NOESY Spectra for 3ag

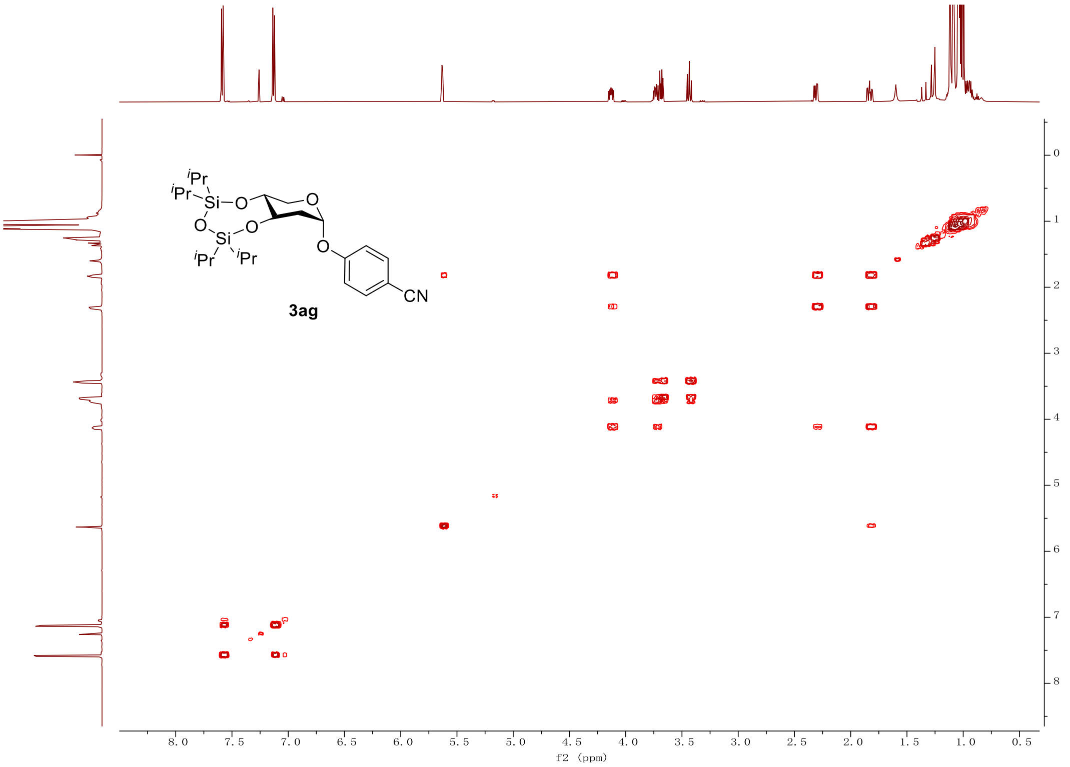

Figure S98: COSY Spectra for 3ag

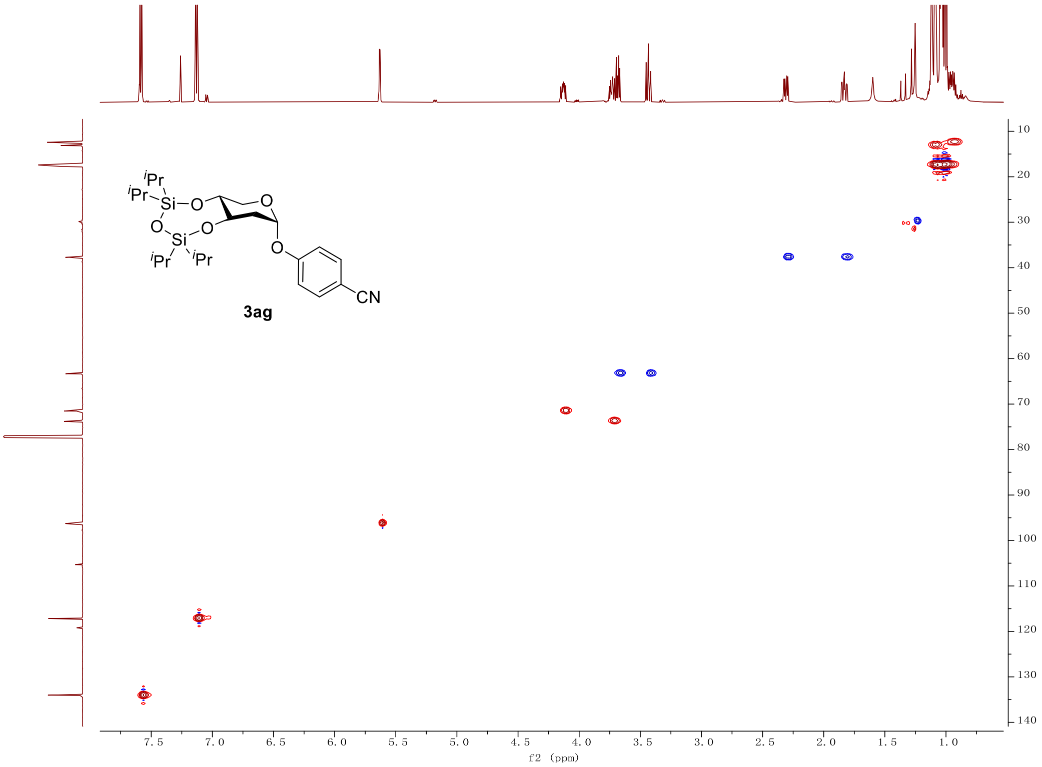

Figure S99: HSQC Spectra for 3ag

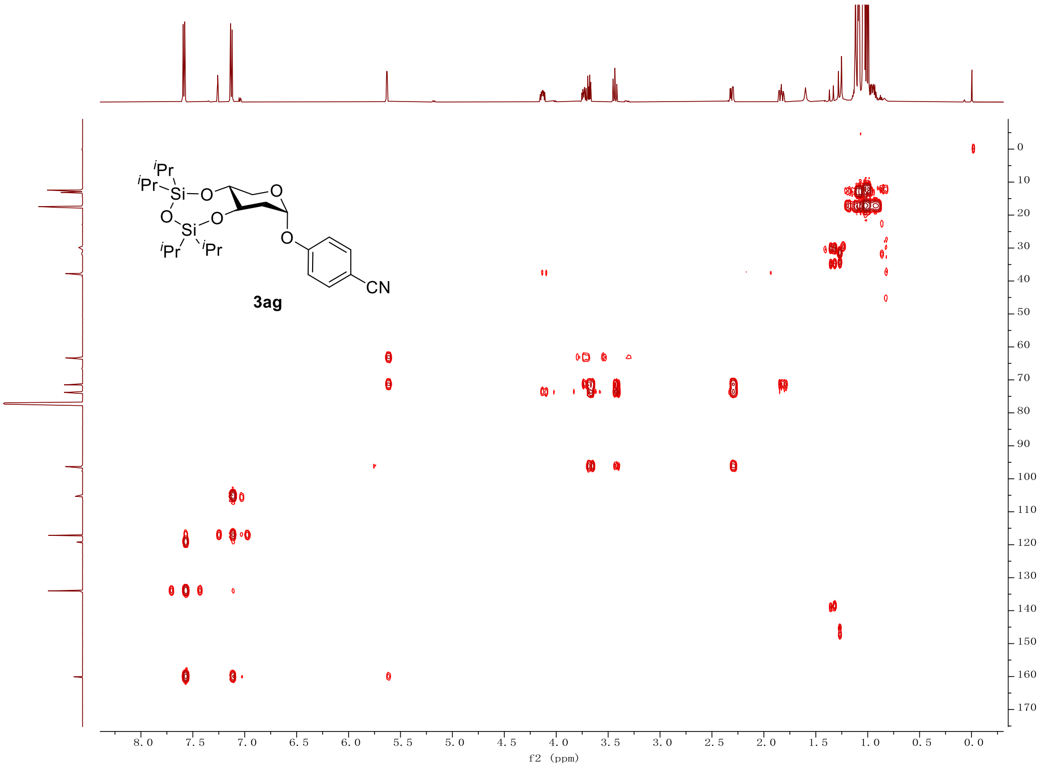

Figure S100: HMBC Spectra for 3ag

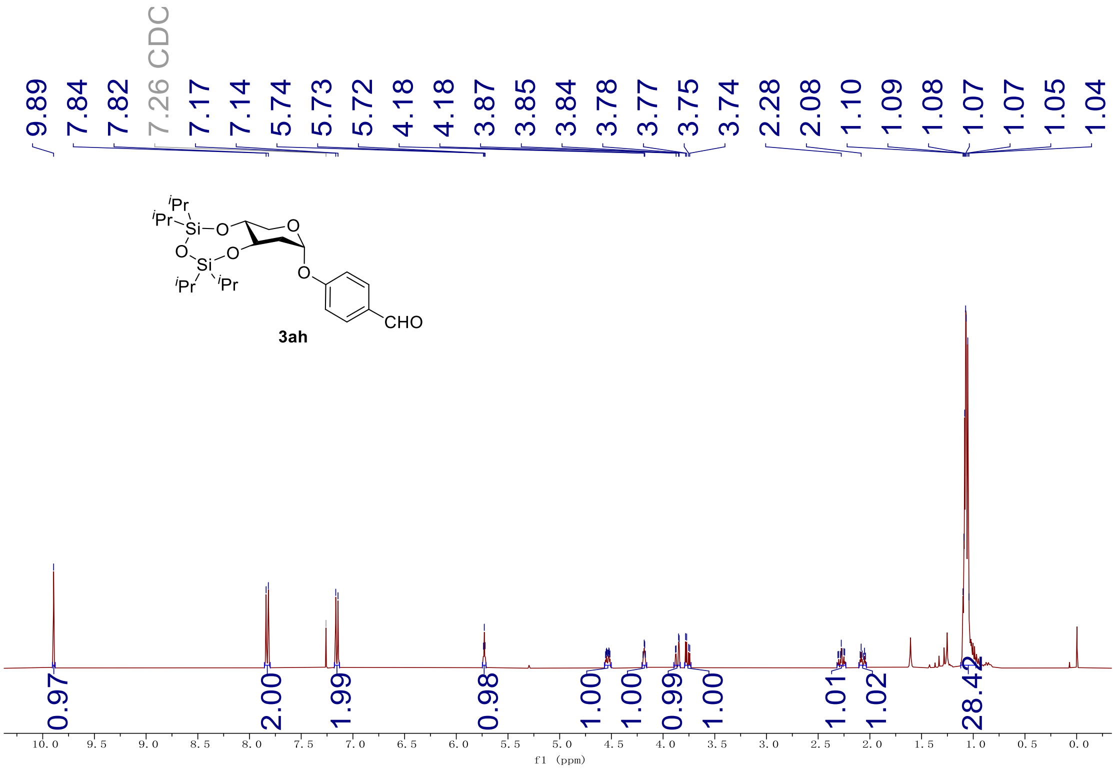

Figure S101. ^{1}H NMR (400 MHz, $CDCl_3$) Spectra for compound 3ah

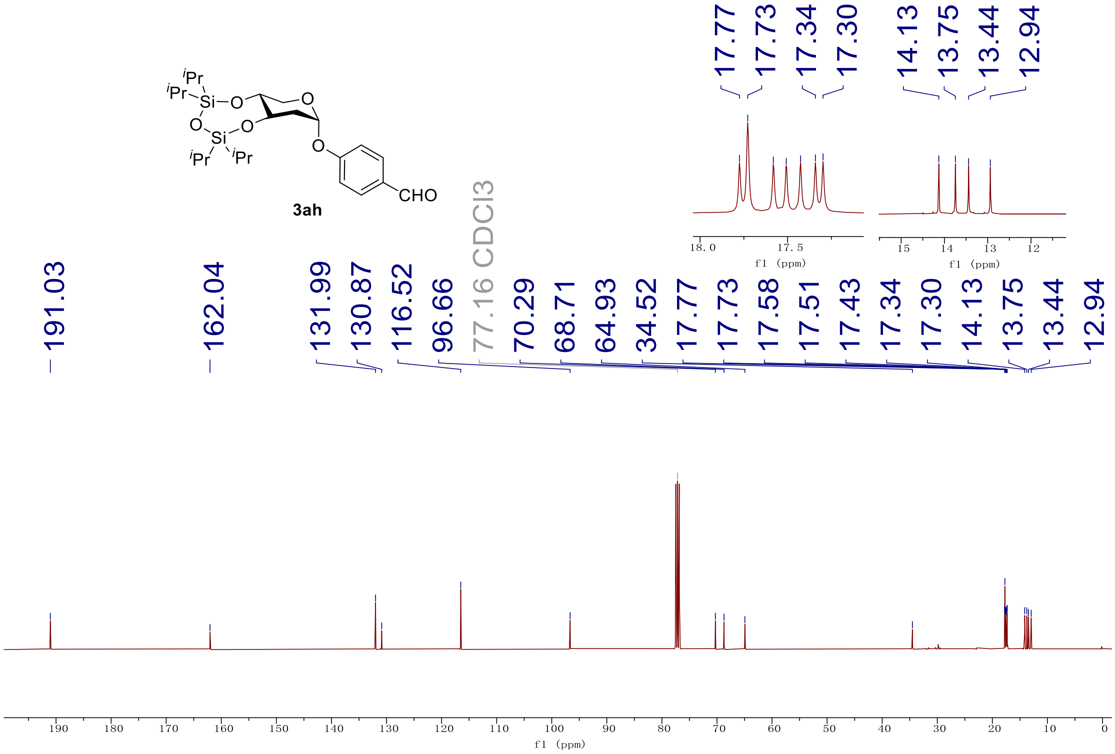

Figure S102. ^{13}C NMR (101 MHz, $CDCl_3$) Spectra for compound 3ah

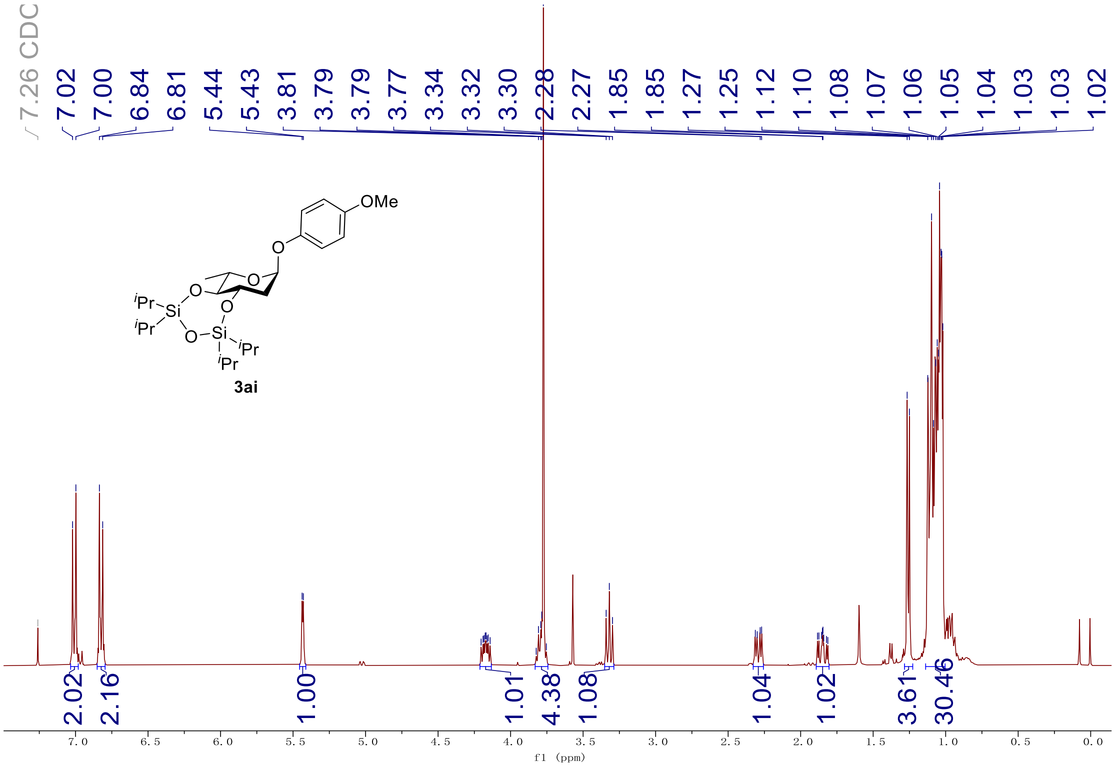

Figure S103. ^{1}H NMR (400 MHz, $CDCl_3$) Spectra for compound 3ai

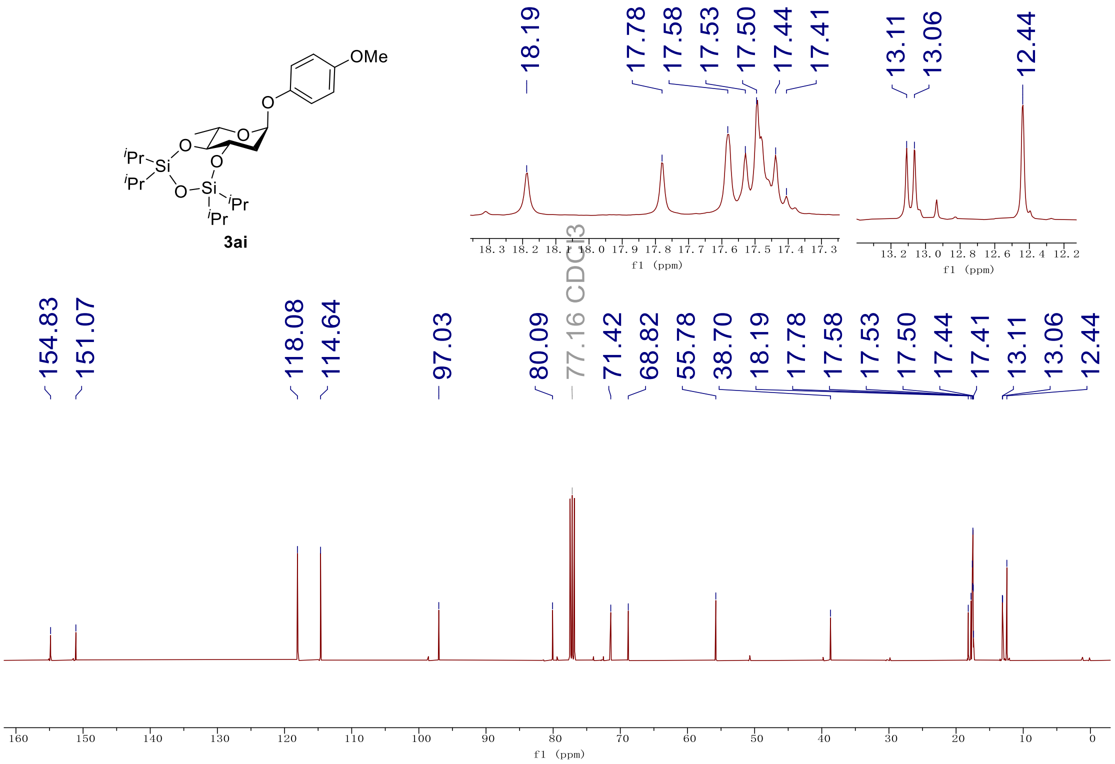

Figure S104. ^{13}C NMR (101 MHz, CDCl$_3$) Spectra for compound 3ai

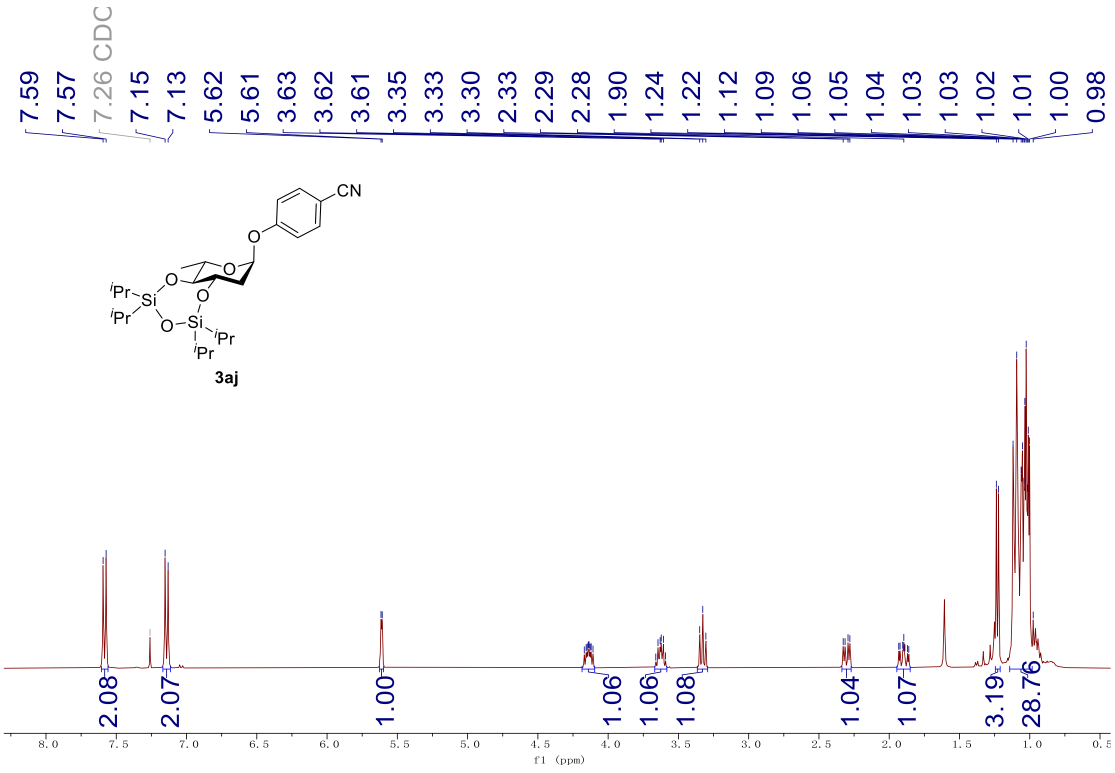

Figure S105. ^{1}H NMR (400 MHz, CDCl$_3$) Spectra for compound 3aj

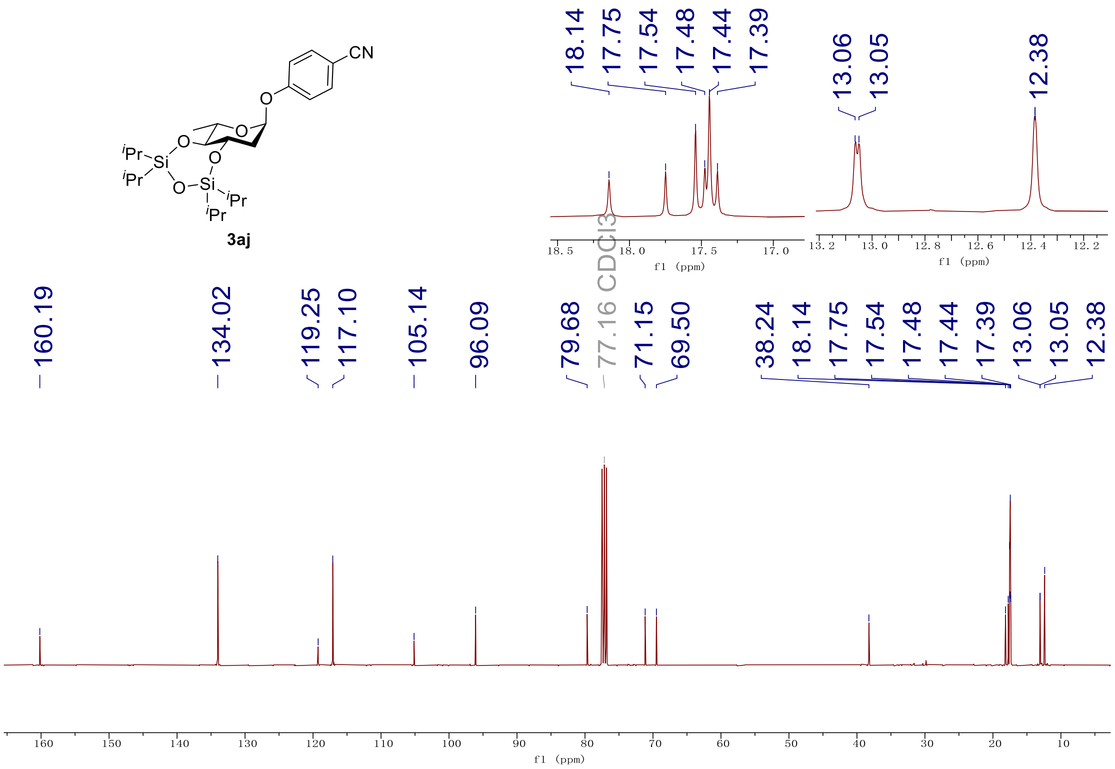

Figure S106. ^{13}C NMR (101 MHz, $CDCl_3$) Spectra for compound 3aj

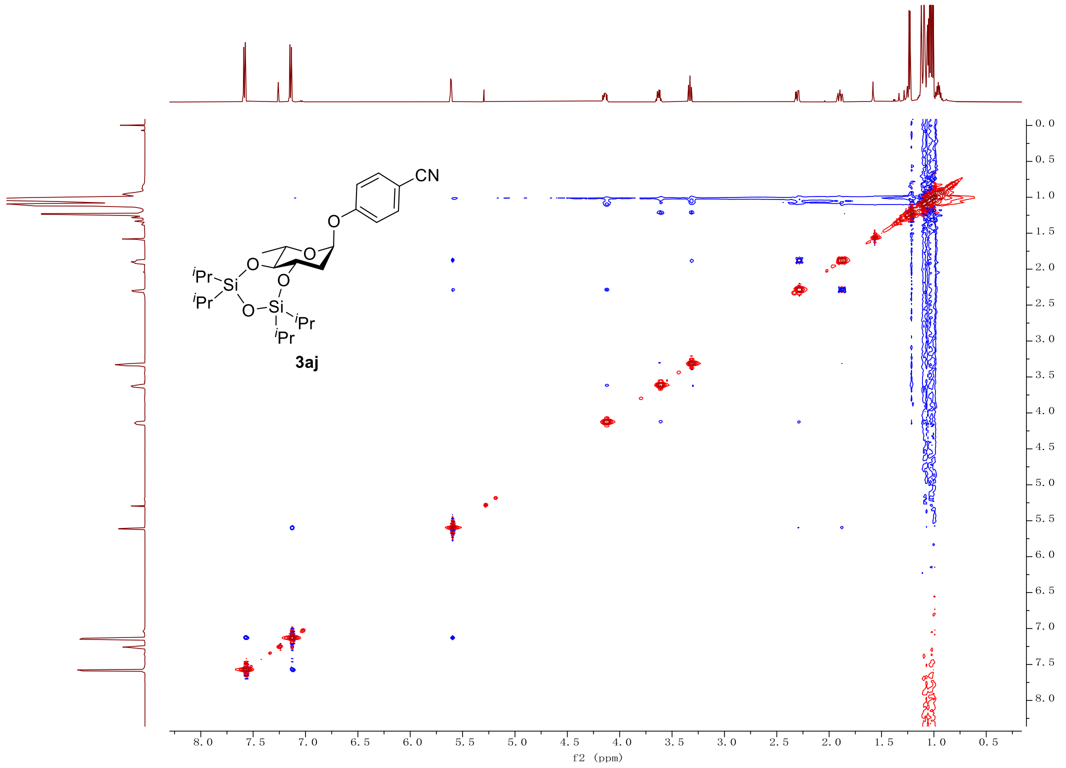

Figure S107: NOESY Spectra for 3aj

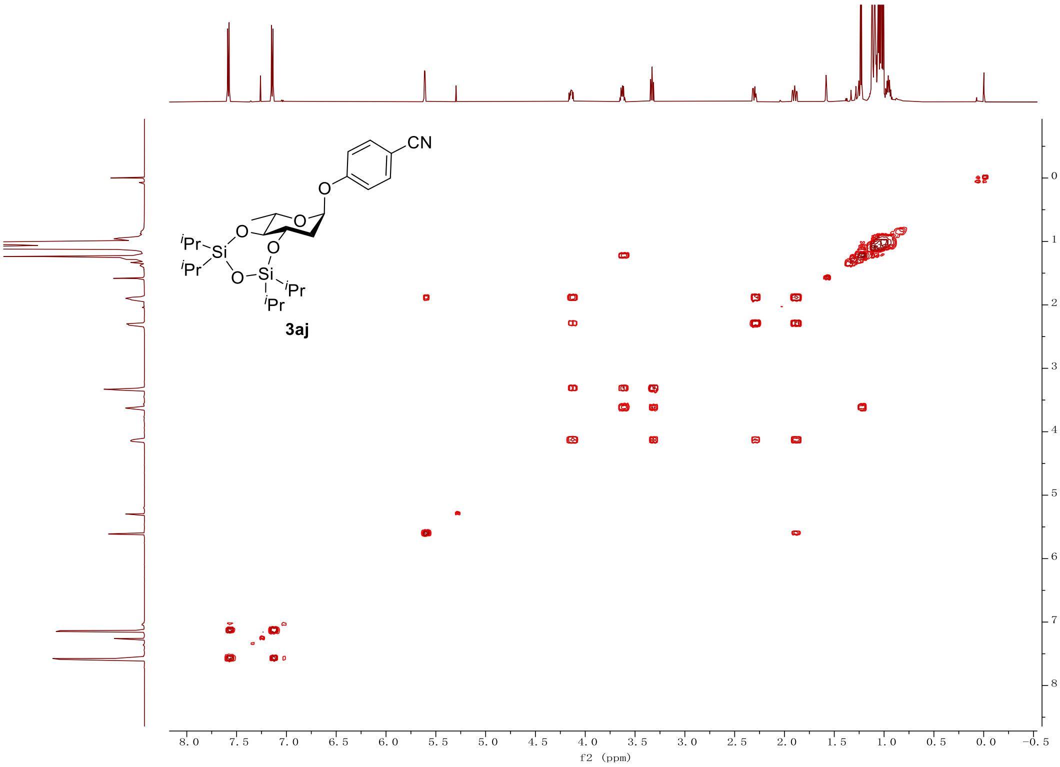

Figure S108: COSY Spectra for 3aj

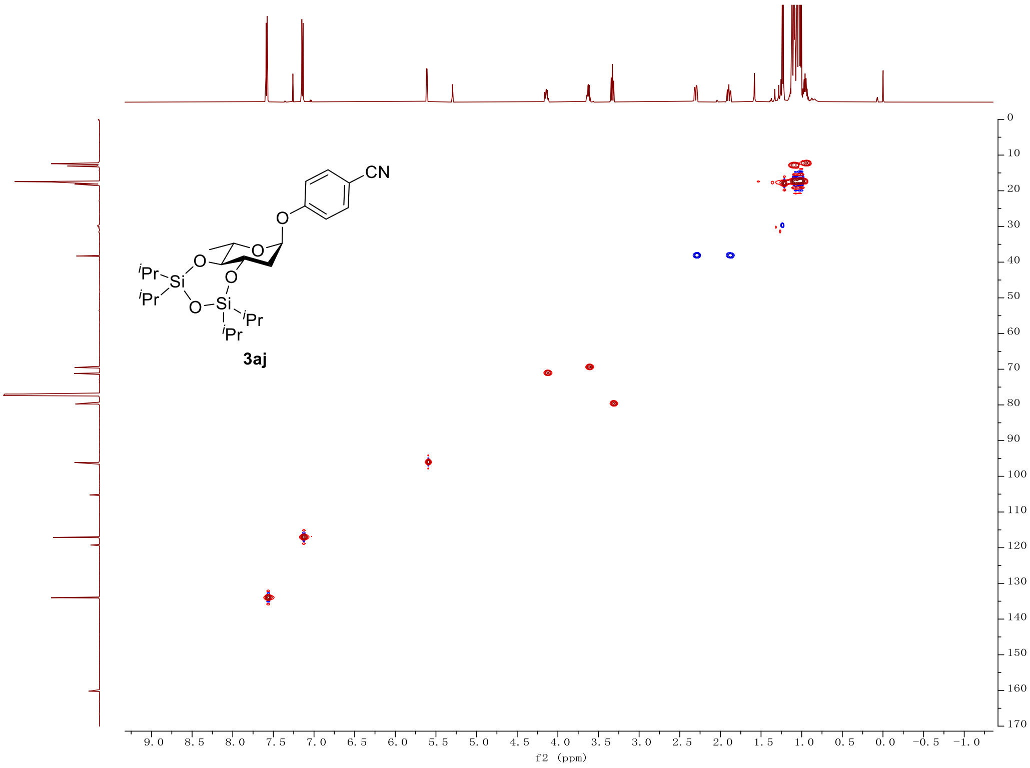

Figure S109: HSQC Spectra for 3aj

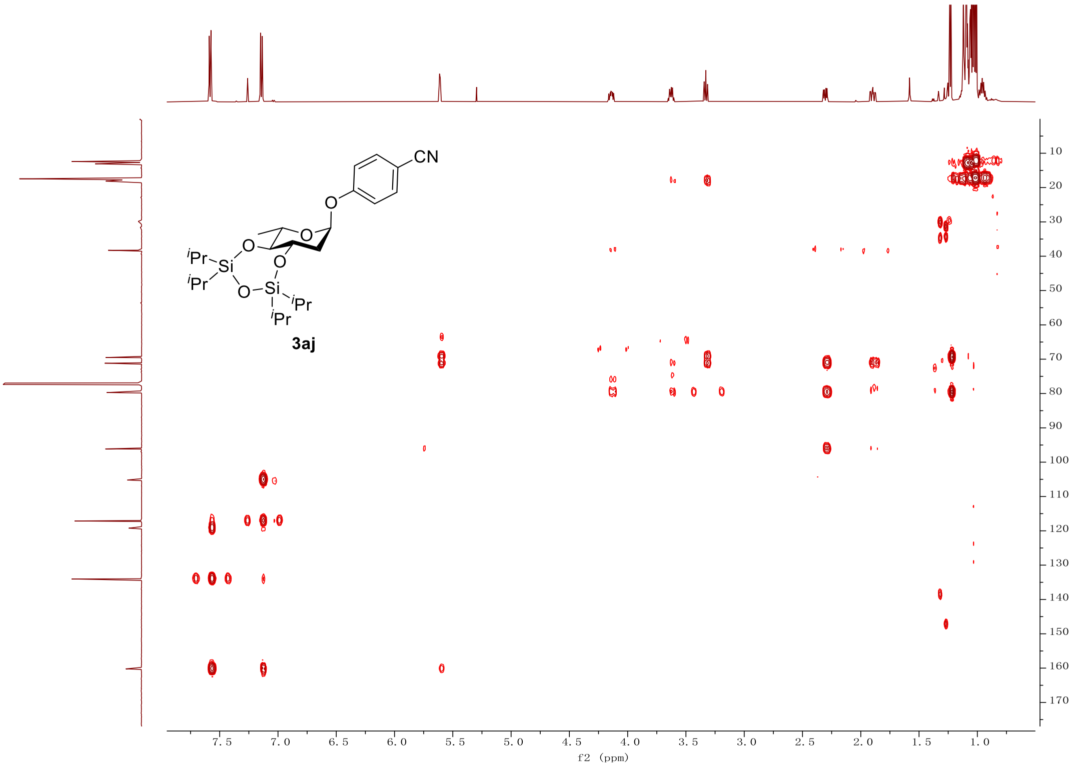

Figure S110: HMBC Spectra for 3aj

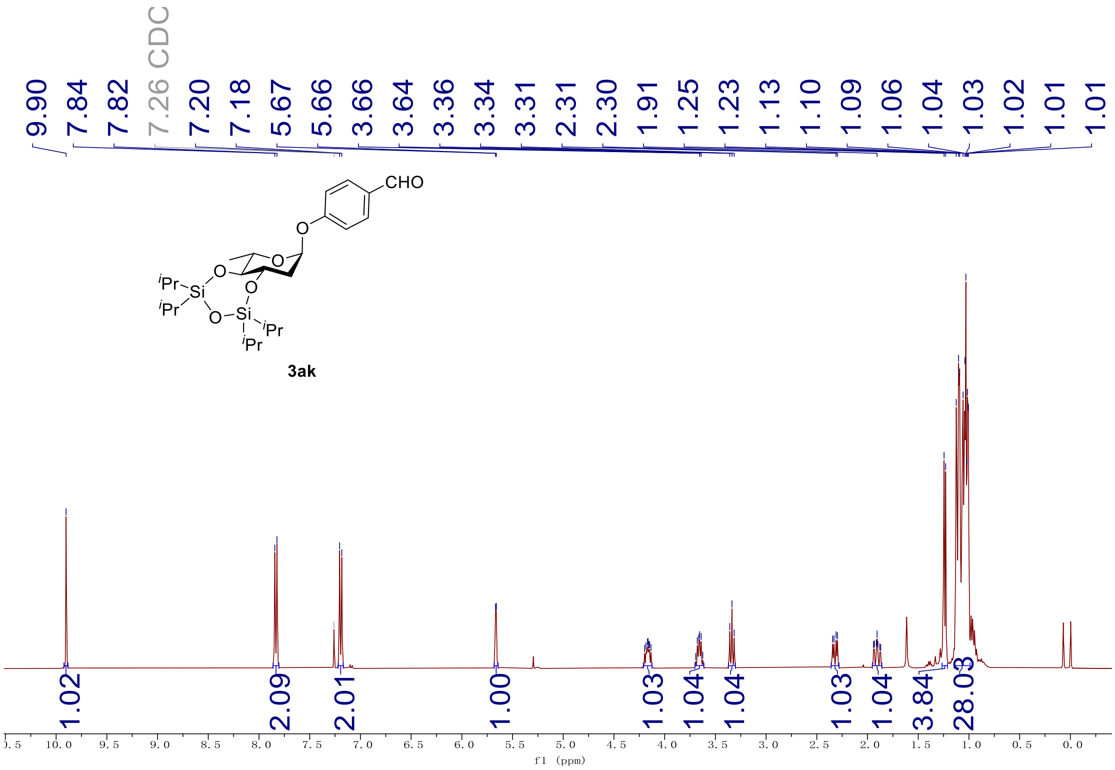

Figure S111. ^{1}H NMR (400 MHz, CDCl3) Spectra for compound 3ak

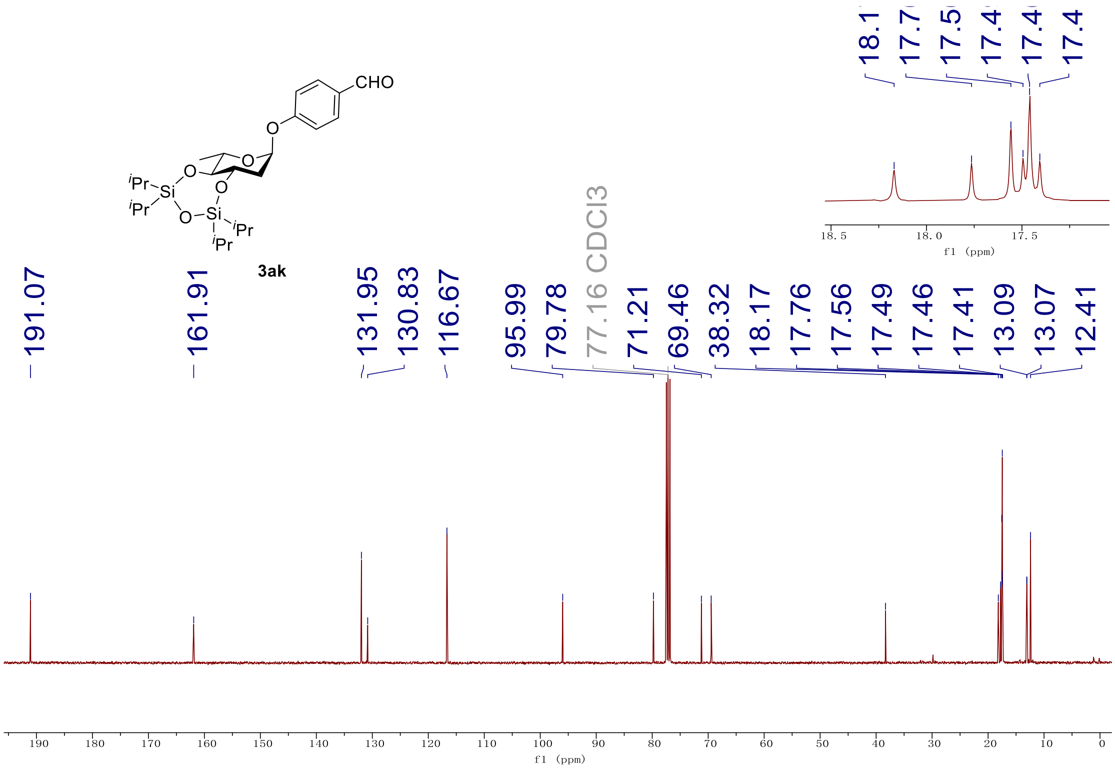

Figure S112. ^{13}**C NMR (101 MHz,** $CDCl_3$**) Spectra for compound 3ak**

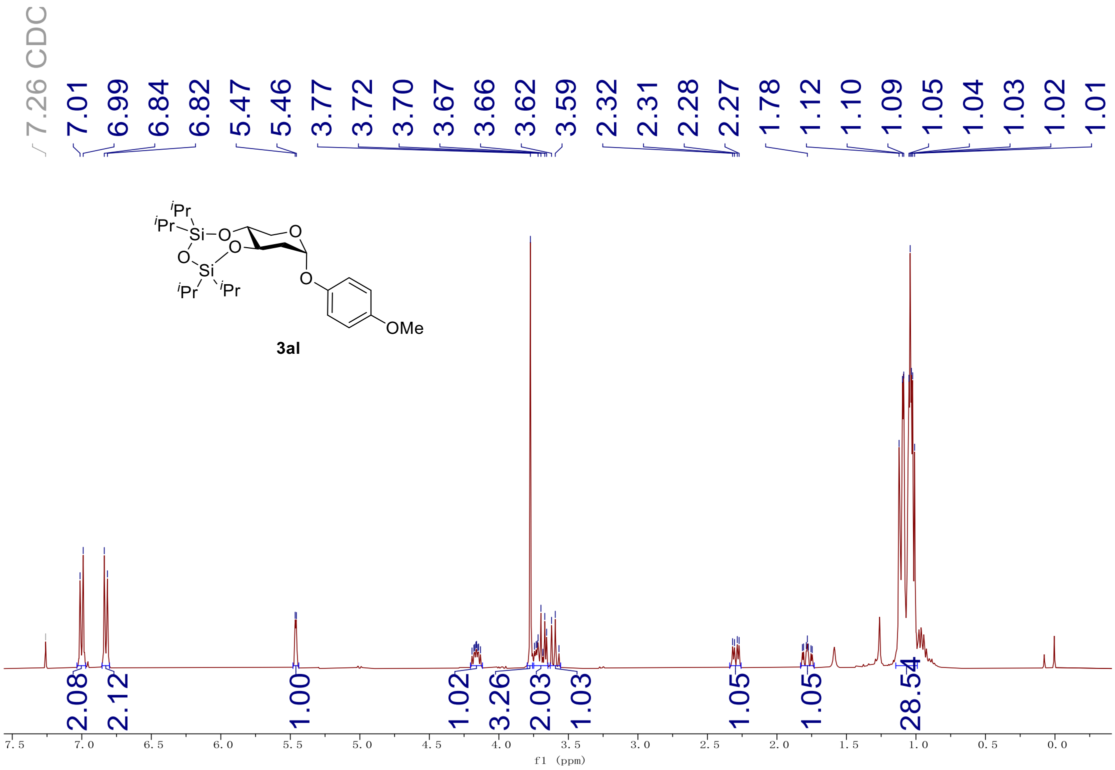

Figure S113. ^{1}**H NMR (400 MHz,** $CDCl_3$**) Spectra for compound 3al**

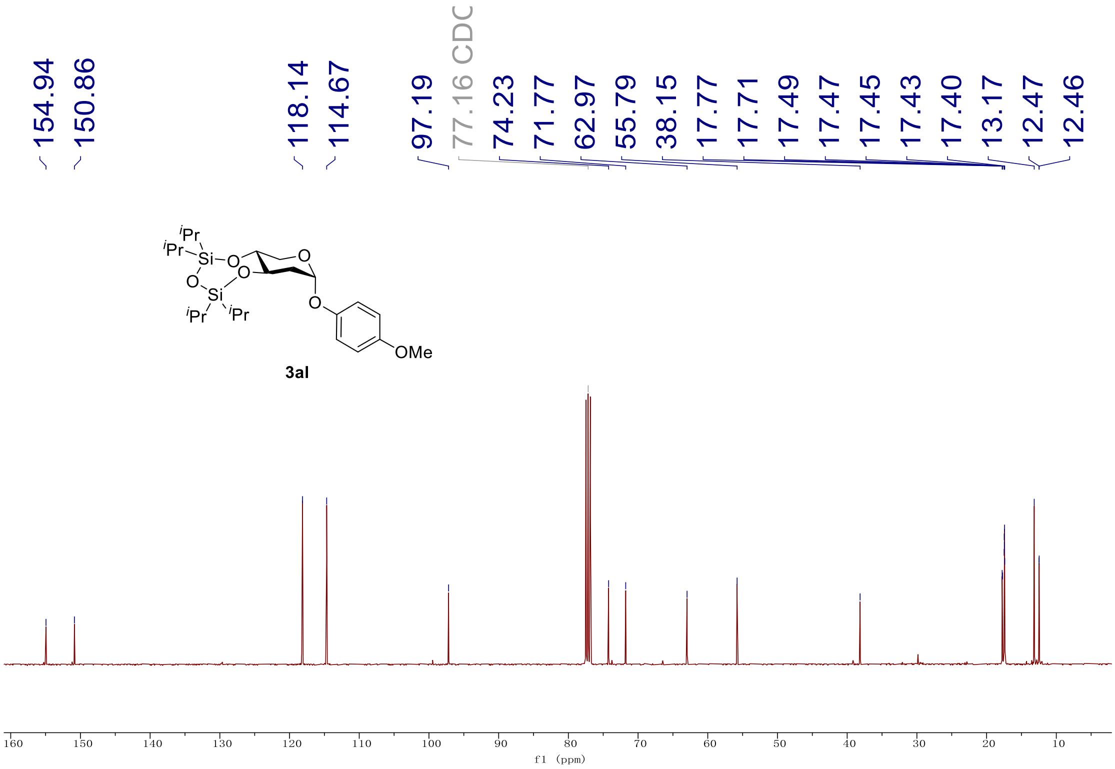

Figure S114. ^{13}C **NMR (101 MHz,** $CDCl_3$**) Spectra for compound 3al**

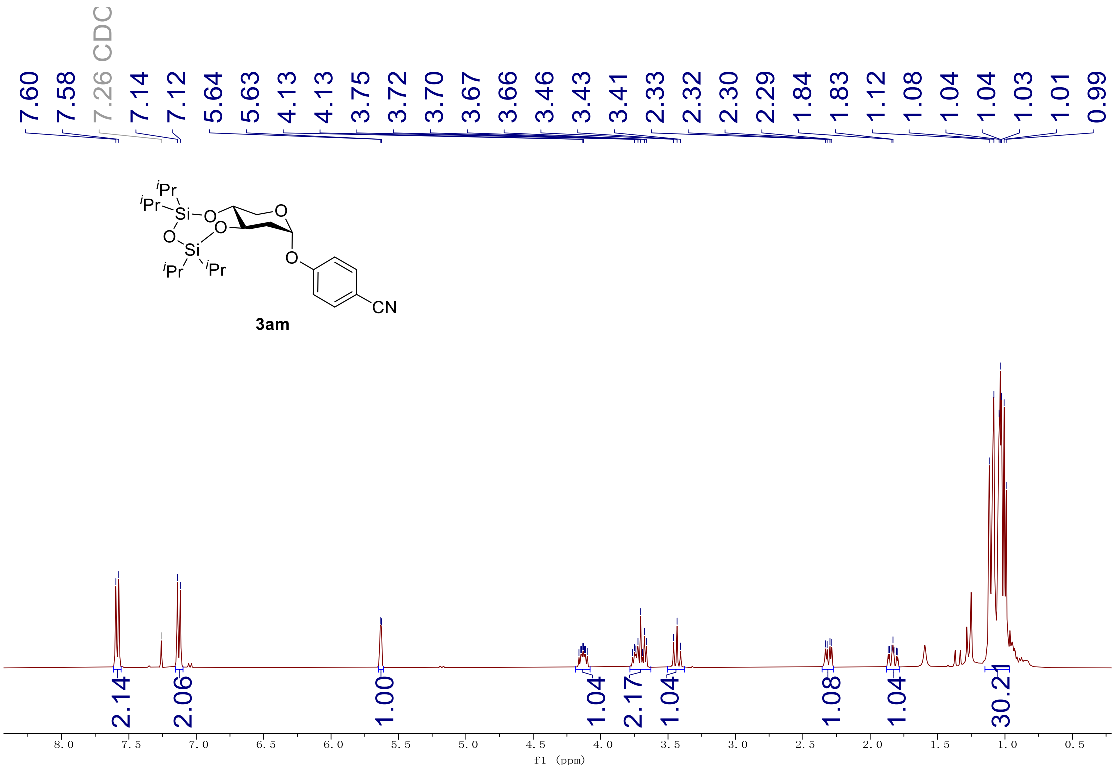

Figure S115. ^{1}H **NMR (400 MHz,** $CDCl_3$**) Spectra for compound 3am**

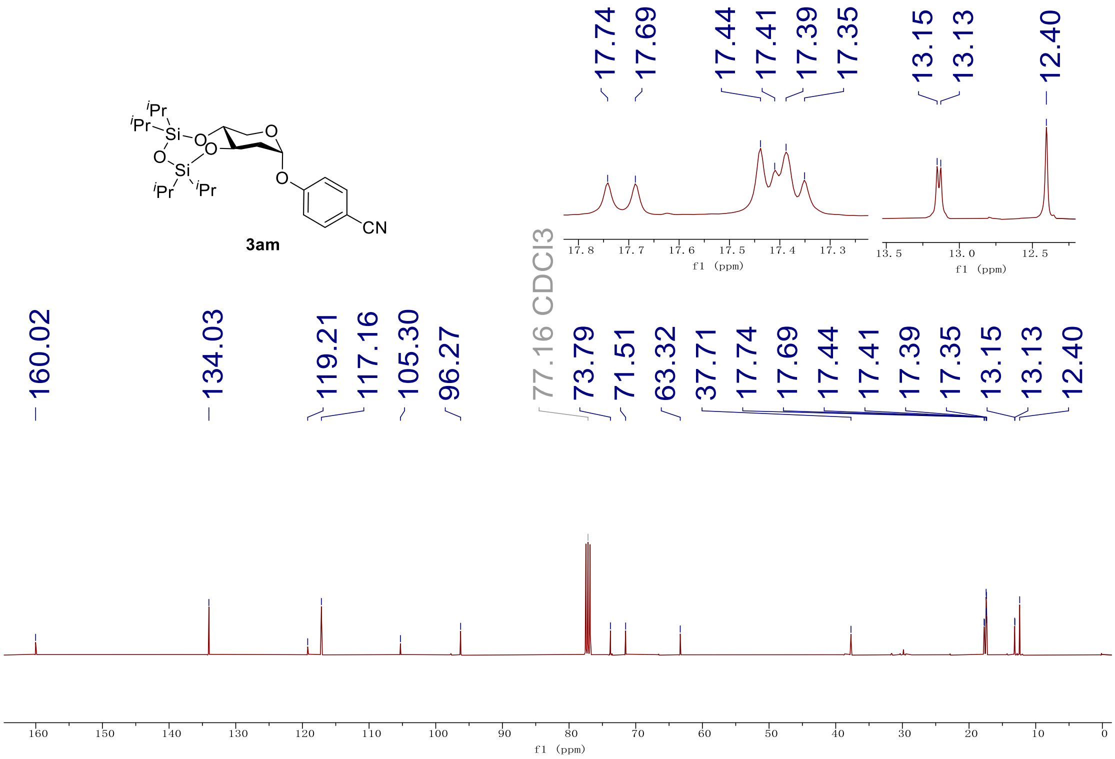

Figure S116. ^{13}C NMR (101 MHz, $CDCl_3$) Spectra for compound 3am

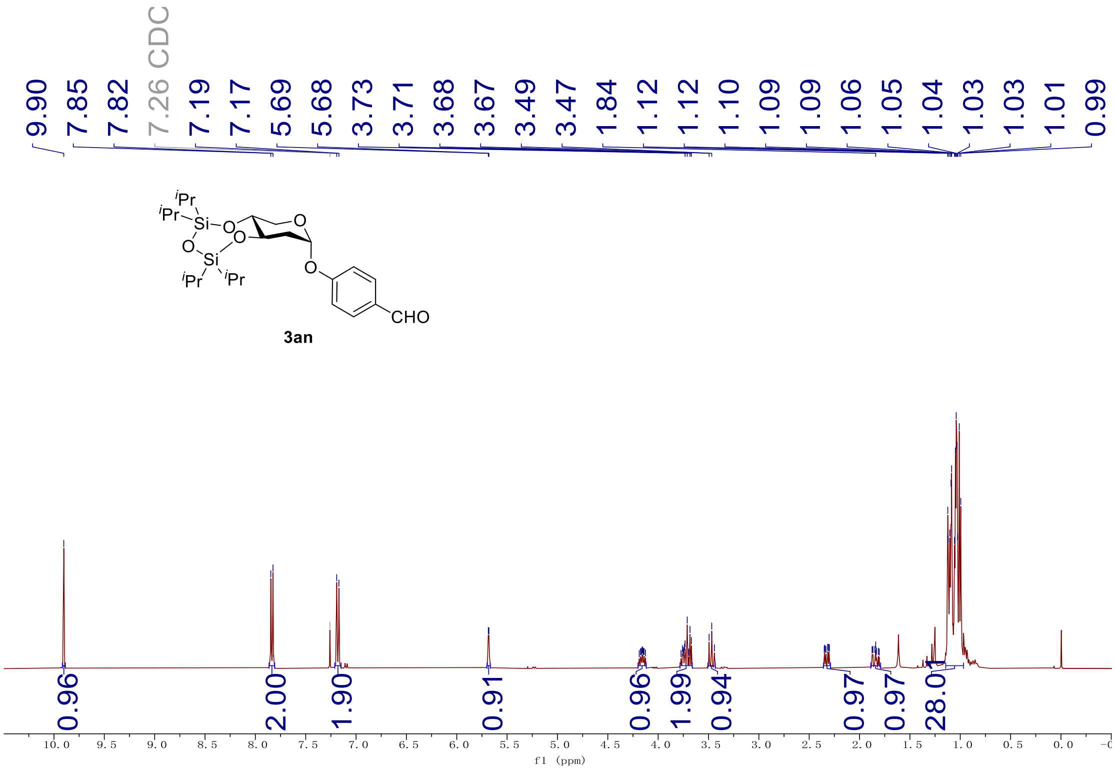

Figure S117. ^{1}H NMR (400 MHz, $CDCl_3$) Spectra for compound 3an

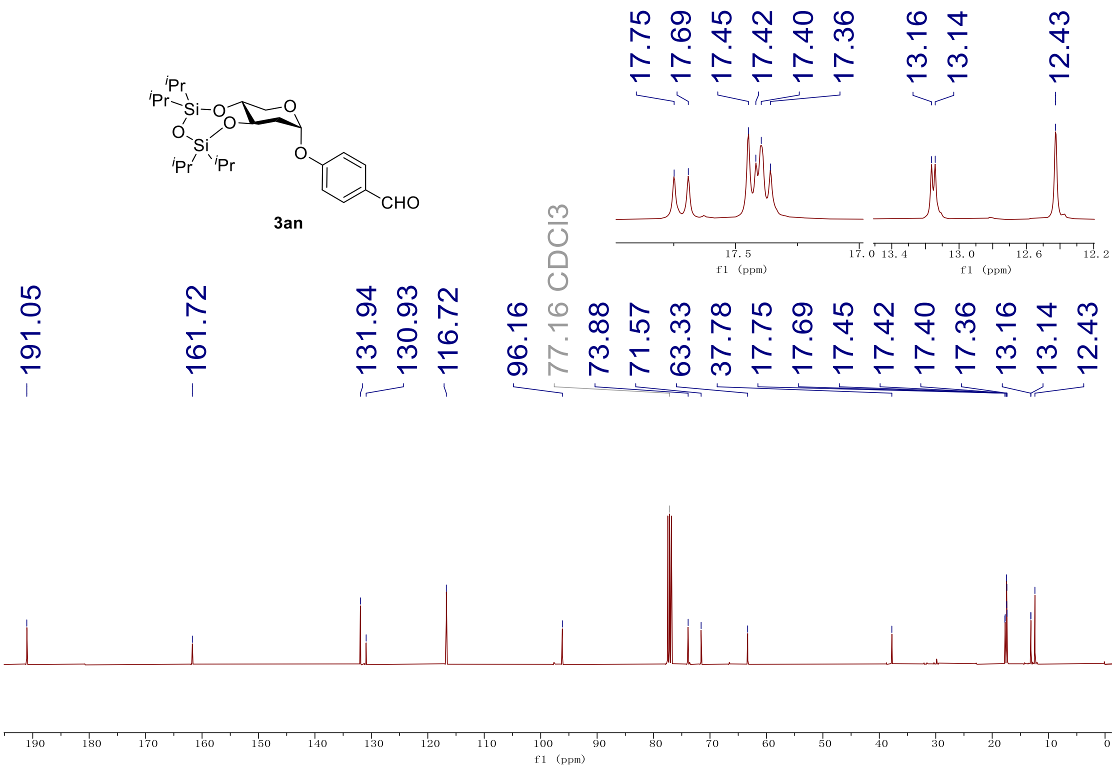

Figure S118. ^{13}C NMR (101 MHz, CDCl$_3$) Spectra for compound 3an

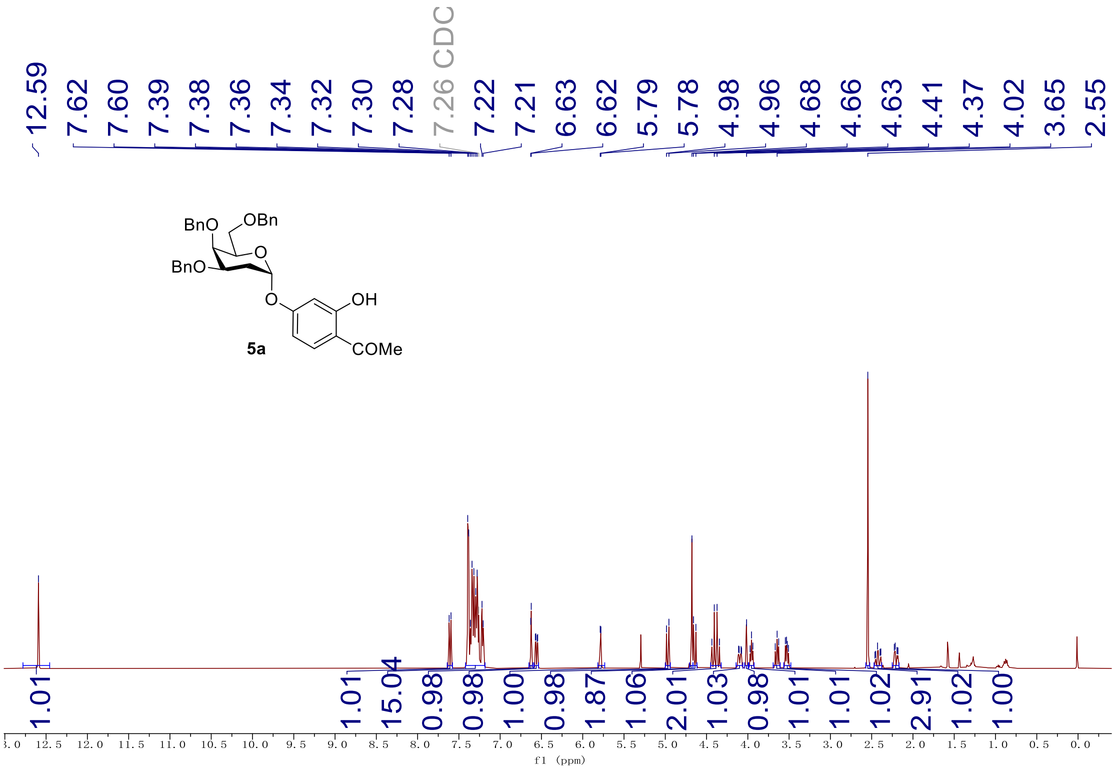

Figure S119. ^{1}H NMR (400 MHz, CDCl$_3$) Spectra for compound 5a

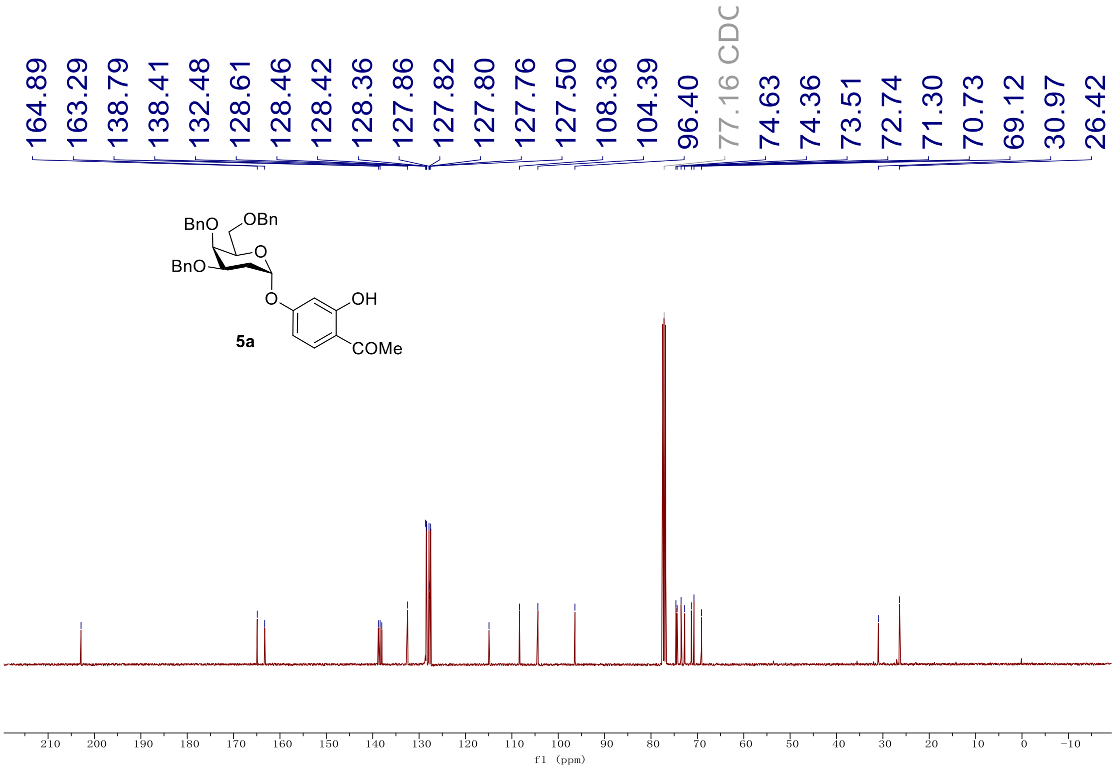

Figure S120. ^{13}C NMR (101 MHz, $CDCl_3$) Spectra for compound 5a

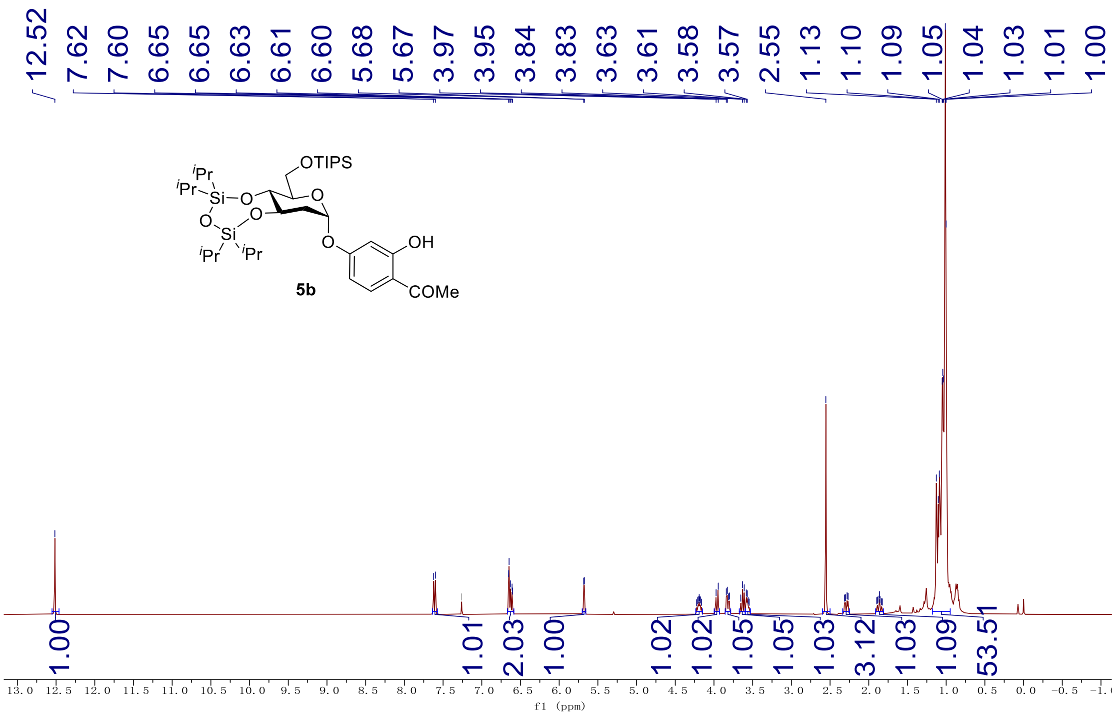

Figure S121. ^{1}H NMR (400 MHz, $CDCl_3$) Spectra for compound 5b

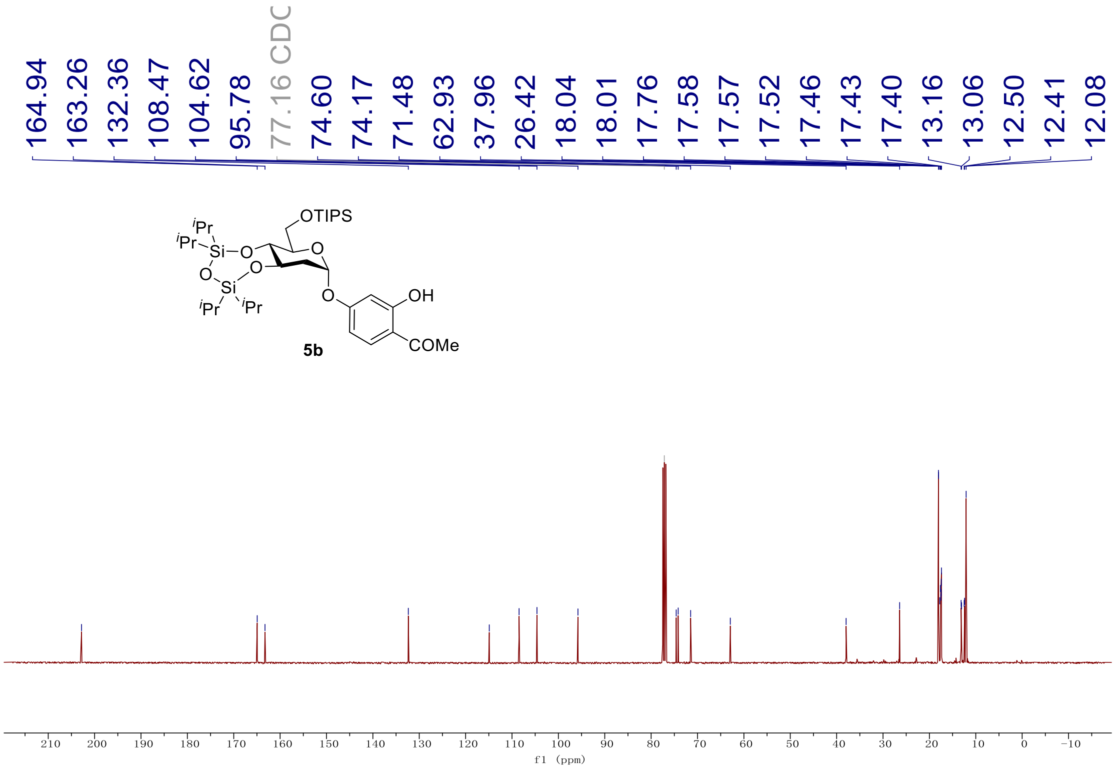

Figure S122. ^{13}C NMR (101 MHz, CDCl$_3$) Spectra for compound 5b

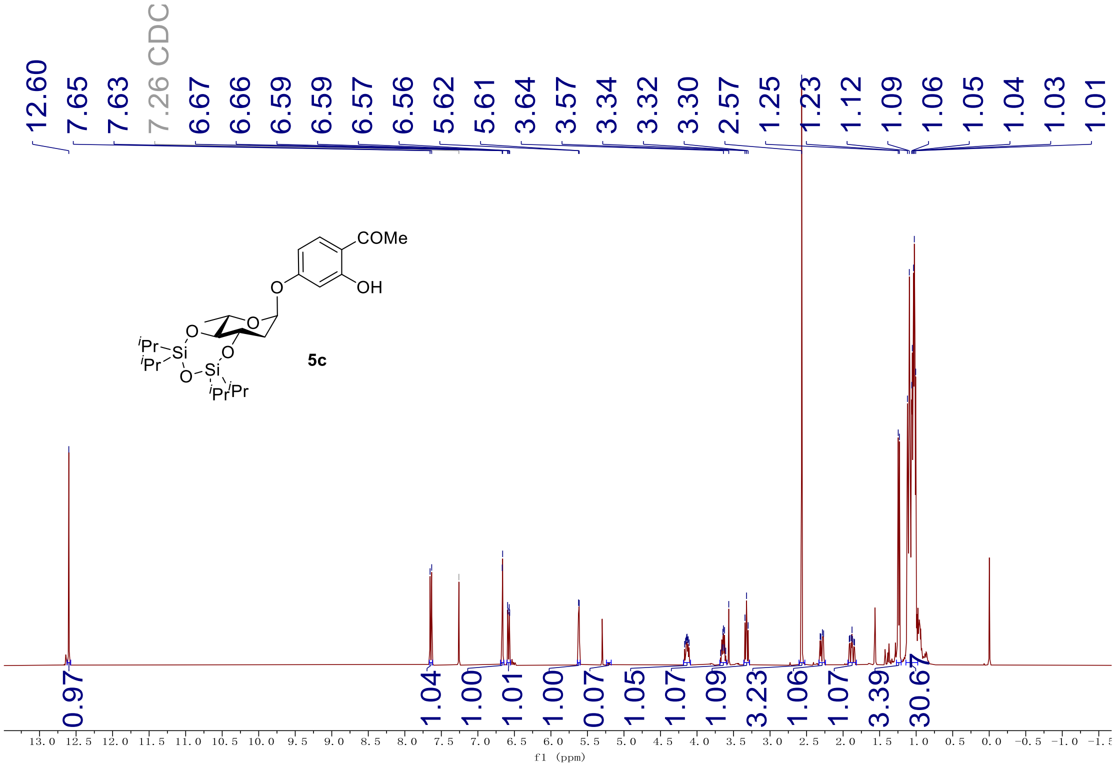

Figure S123. ^{1}H NMR (400 MHz, CDCl$_3$) Spectra for compound 5c

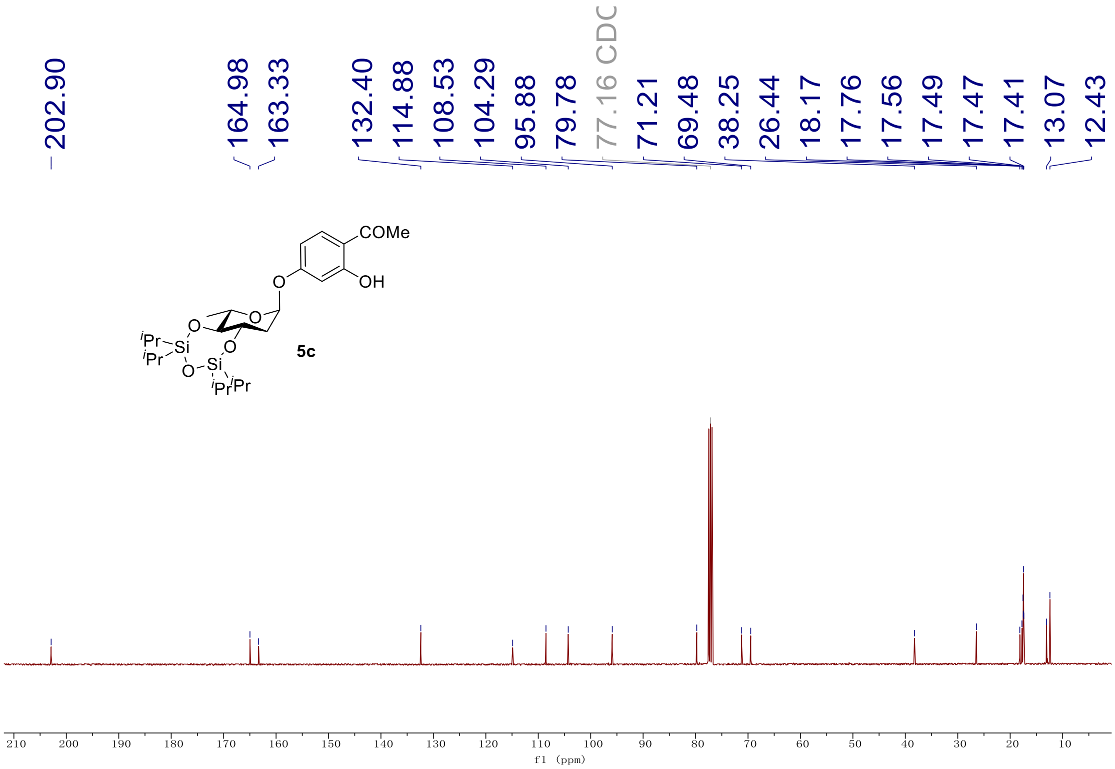

Figure S124. ^{13}C NMR (101 MHz, CDCl$_3$) Spectra for compound 5c

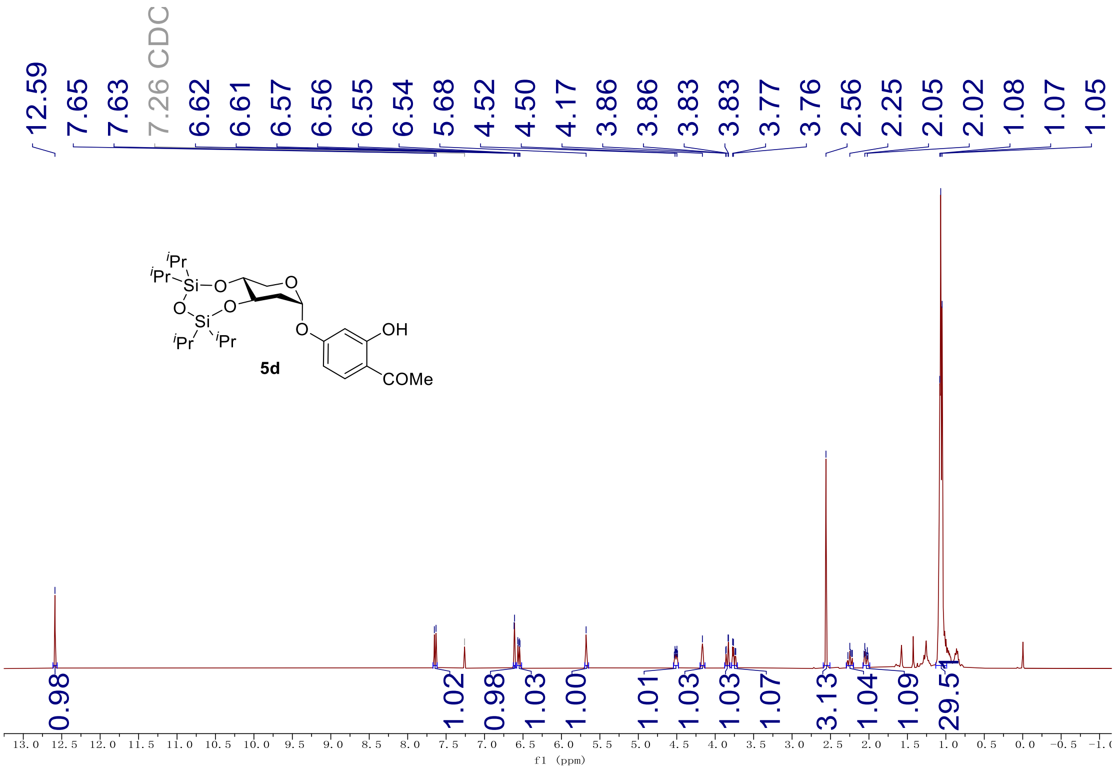

Figure S125. ^{1}H NMR (400 MHz, CDCl$_3$) Spectra for compound 5d

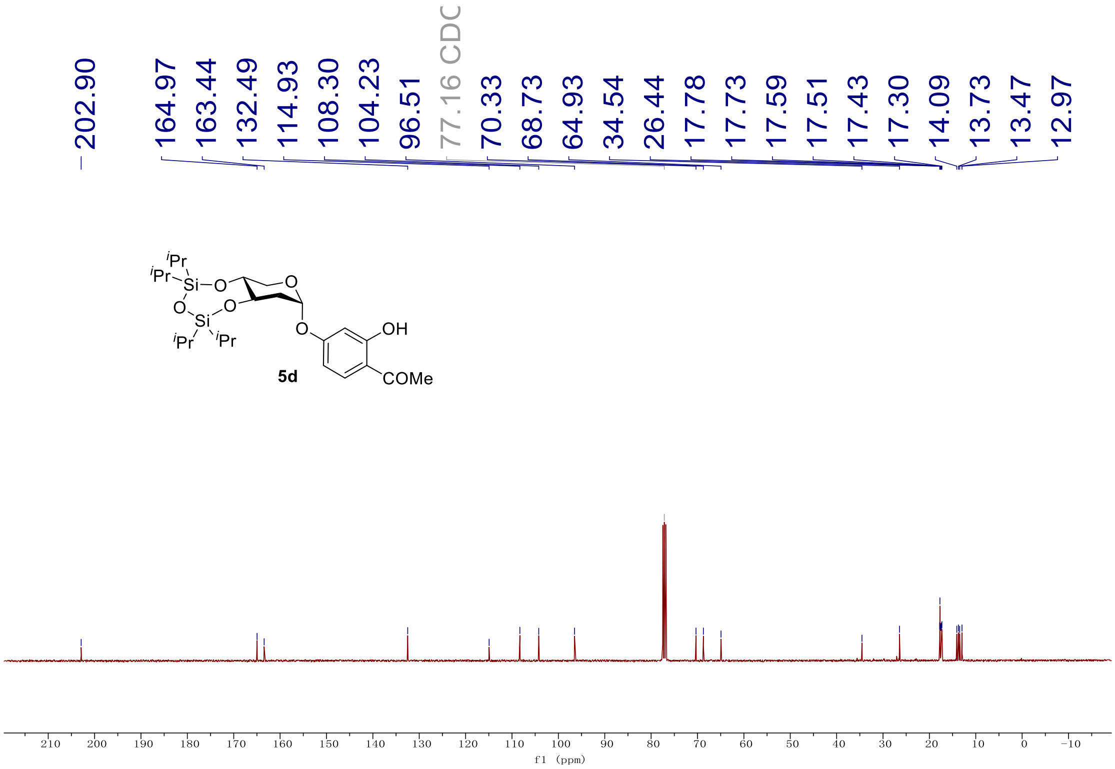

Figure S126. ^{13}C NMR (101 MHz, $CDCl_3$) Spectra for compound 5d

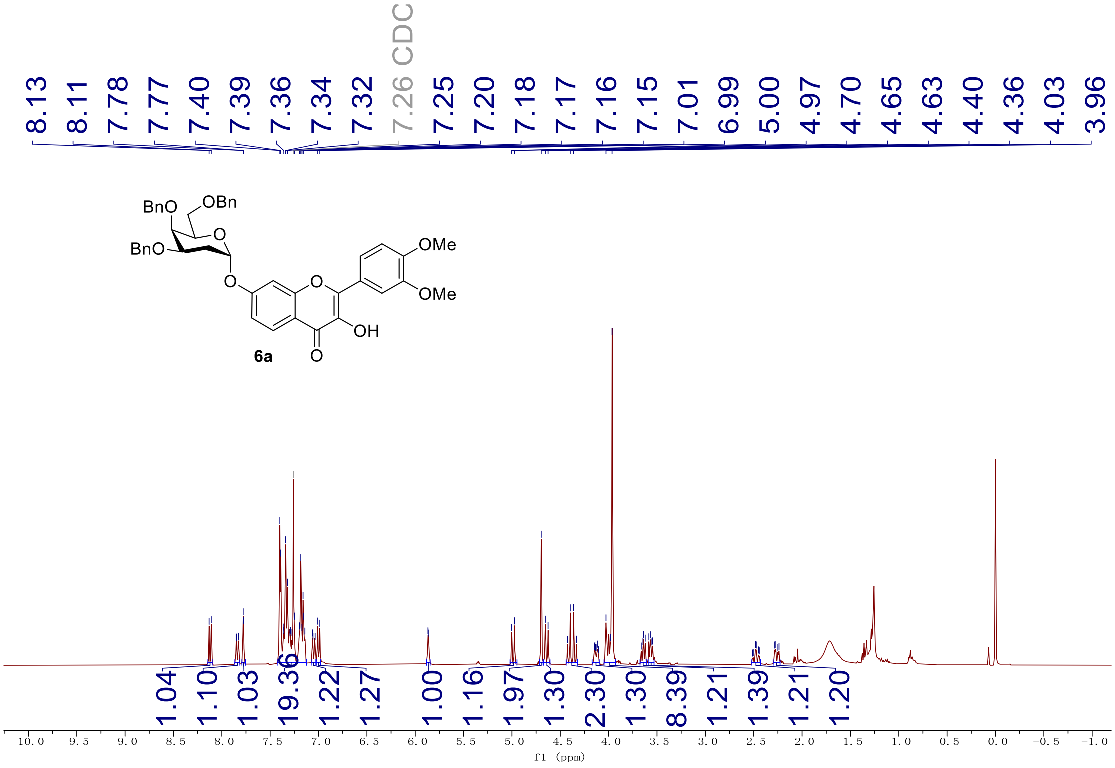

Figure S127. ^{1}H NMR (400 MHz, $CDCl_3$) Spectra for compound 6a

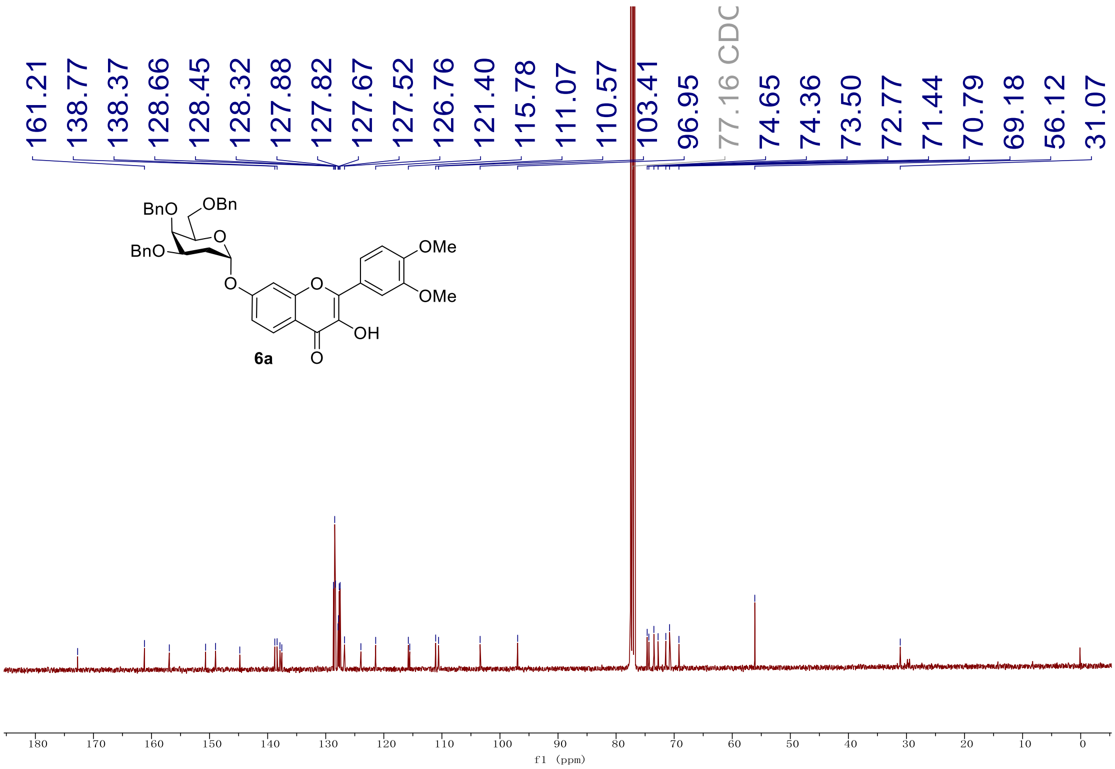

Figure S128. ^{13}C NMR (101 MHz, $CDCl_3$) Spectra for compound 6a

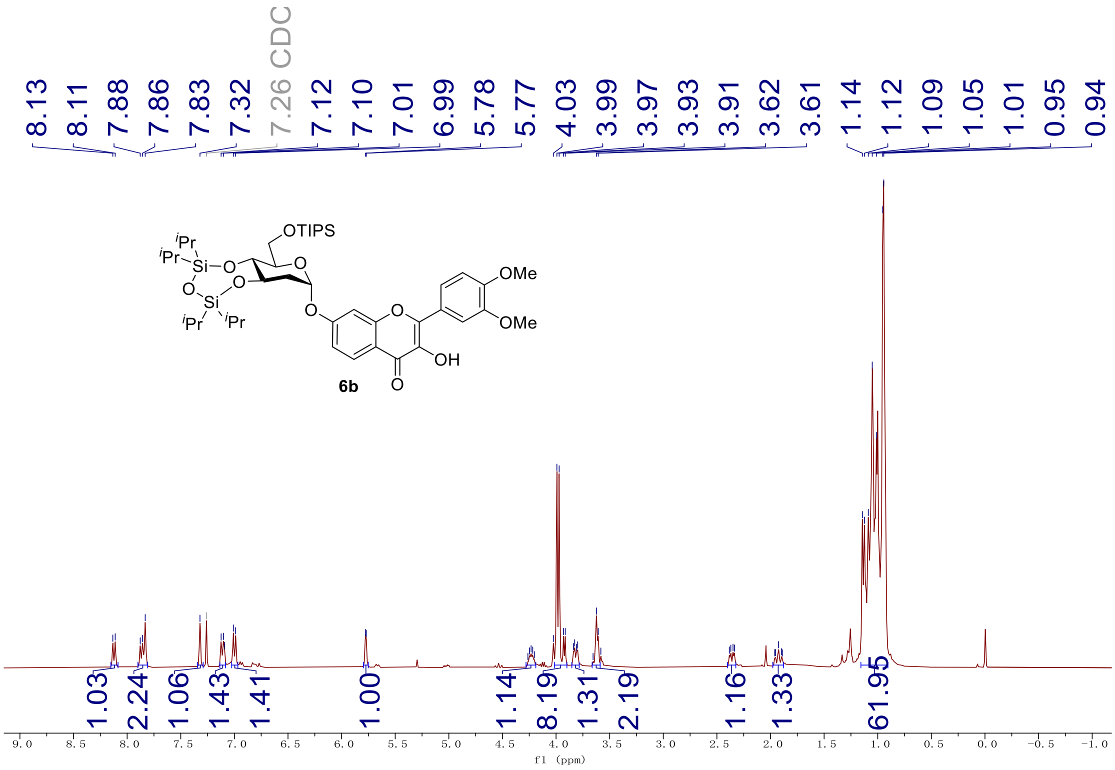

Figure S129. ^{1}H NMR (400 MHz, $CDCl_3$) Spectra for compound 6b

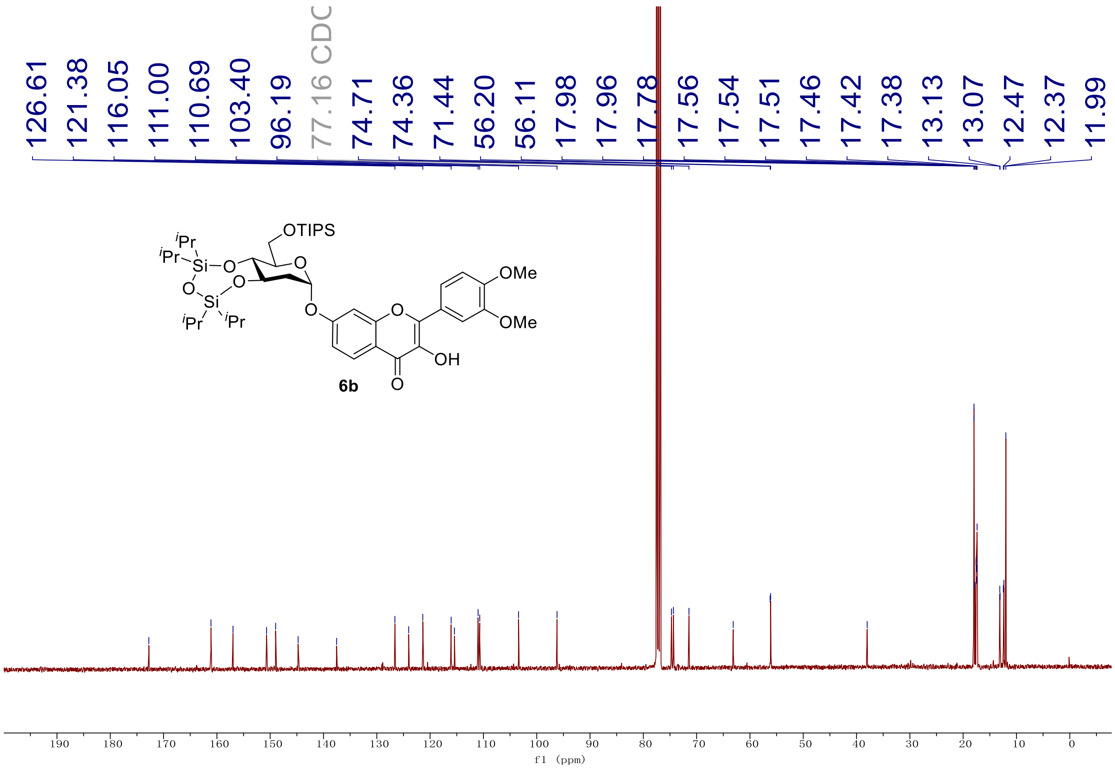

Figure S130. ^{13}**C NMR (101 MHz, CDCl**$_3$**) Spectra for compound 6b**

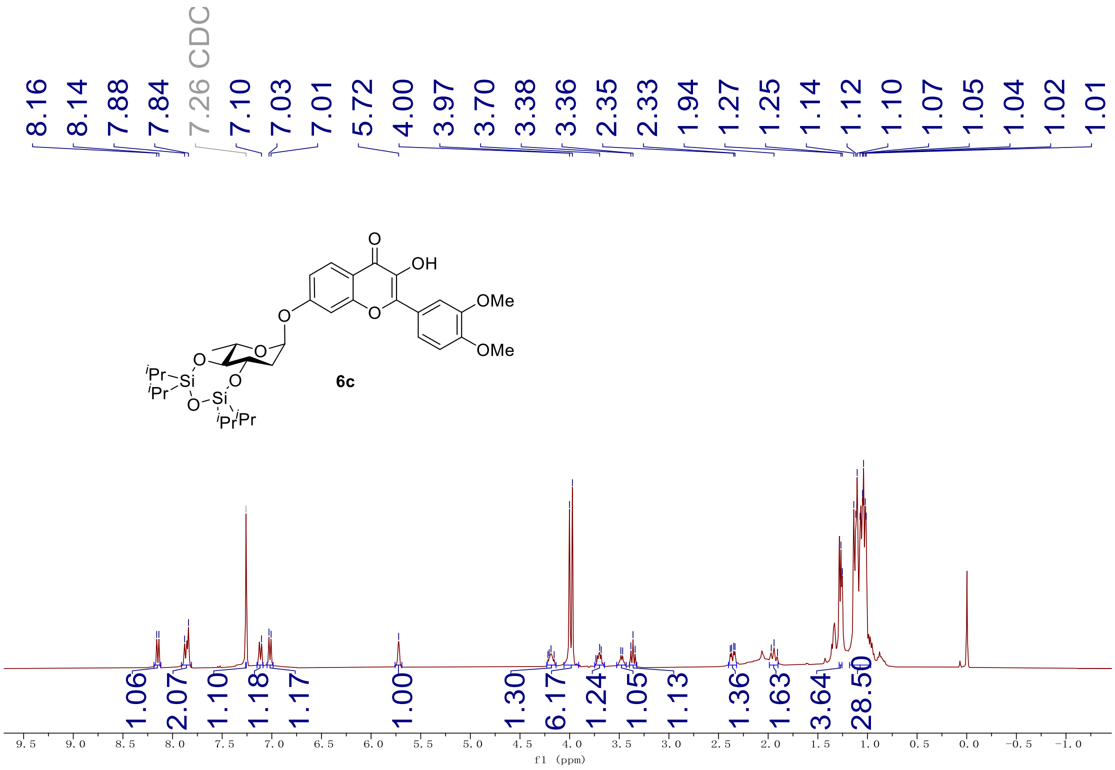

Figure S131. ^{1}**H NMR (400 MHz, CDCl**$_3$**) Spectra for compound 6c**

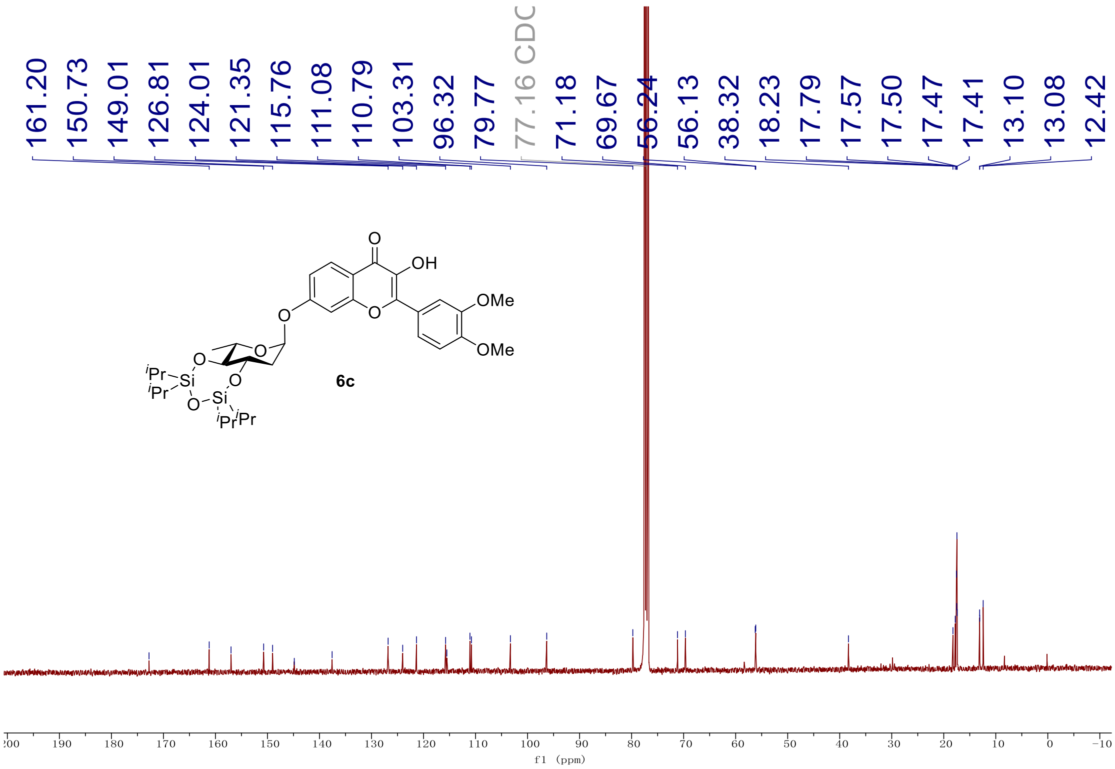

Figure S132. ^{13}C NMR (101 MHz, CDCl$_3$) Spectra for compound 6c

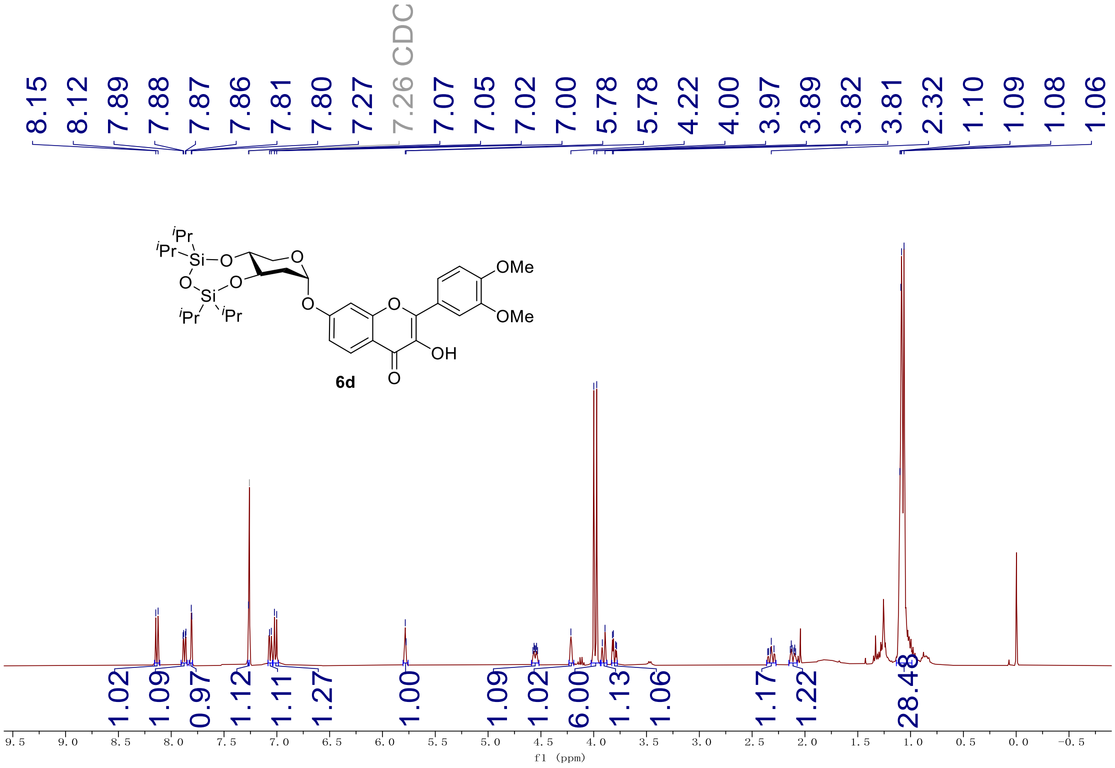

Figure S133. ^{1}H NMR (400 MHz, CDCl$_3$) Spectra for compound 6d

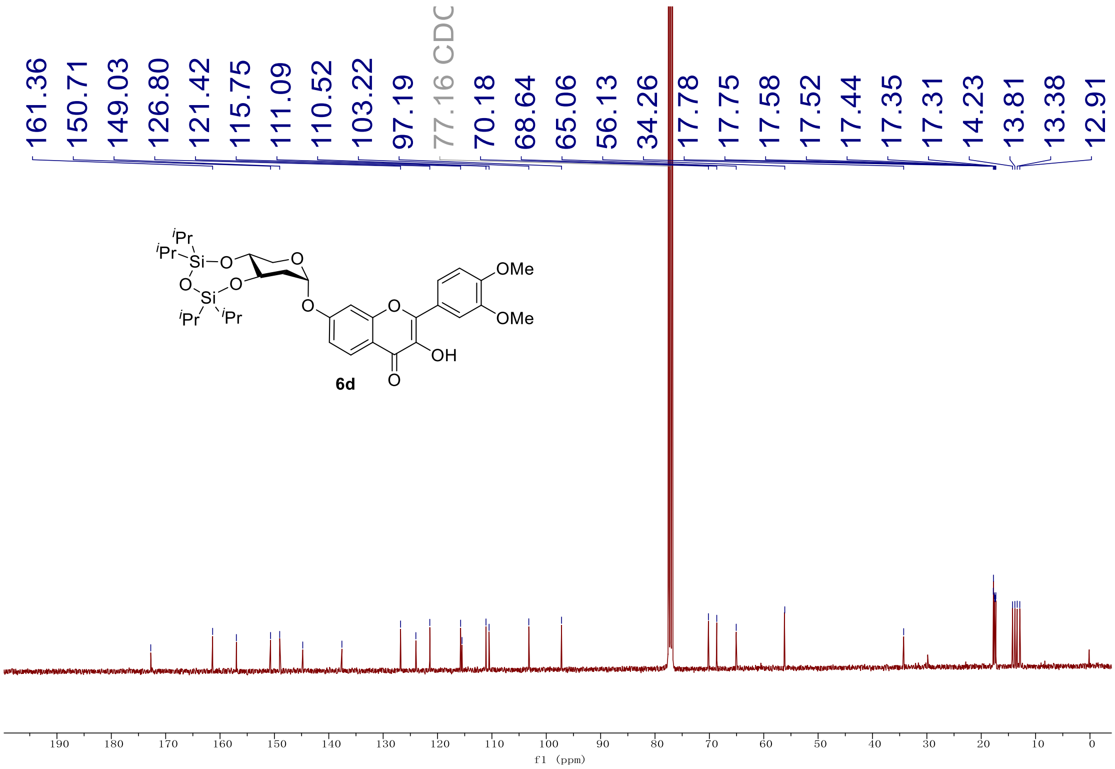

Figure S134. ^{13}C NMR (101 MHz, $CDCl_3$) Spectra for compound 6d

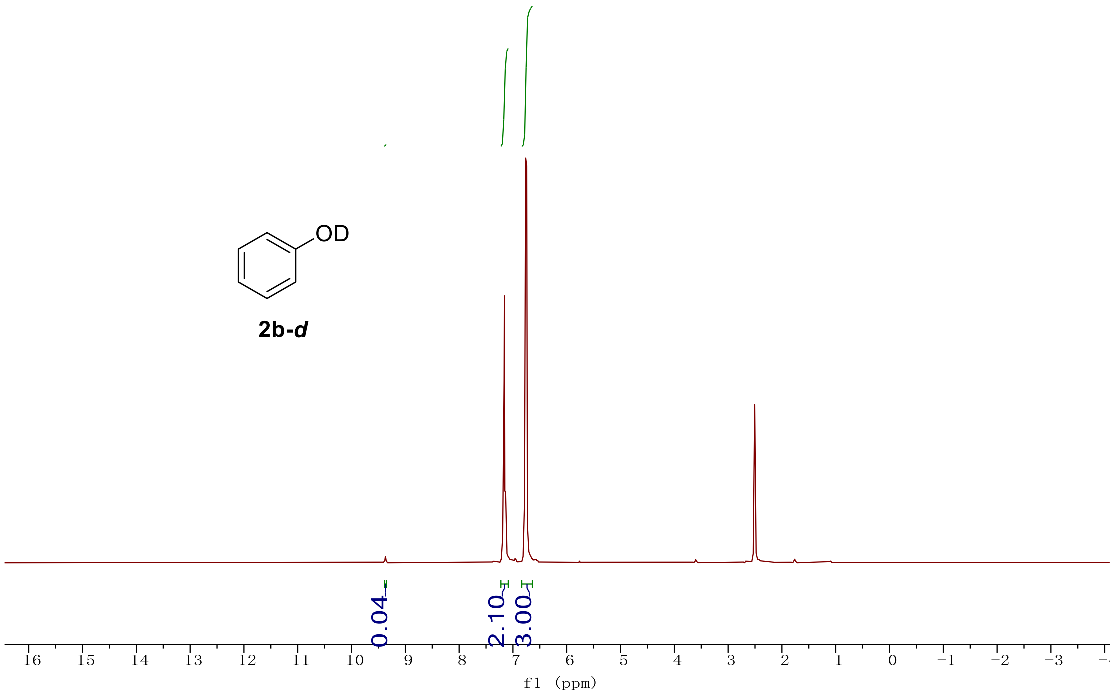

Figure S135. ^{1}H-NMR (400 MHz, DMSO-d_6) spectrum of compound 2b-*d*

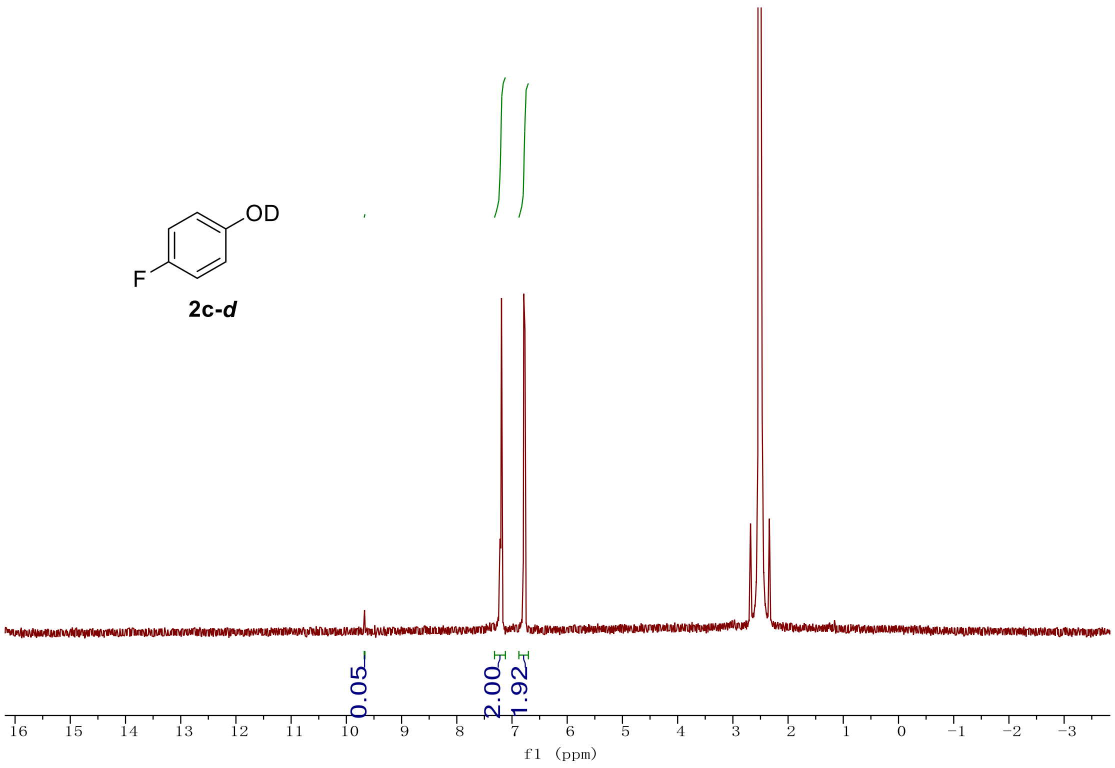

Figure S136. **^{1}H-NMR (400 MHz, DMSO-d$_6$) spectrum of compound 2c-*d***

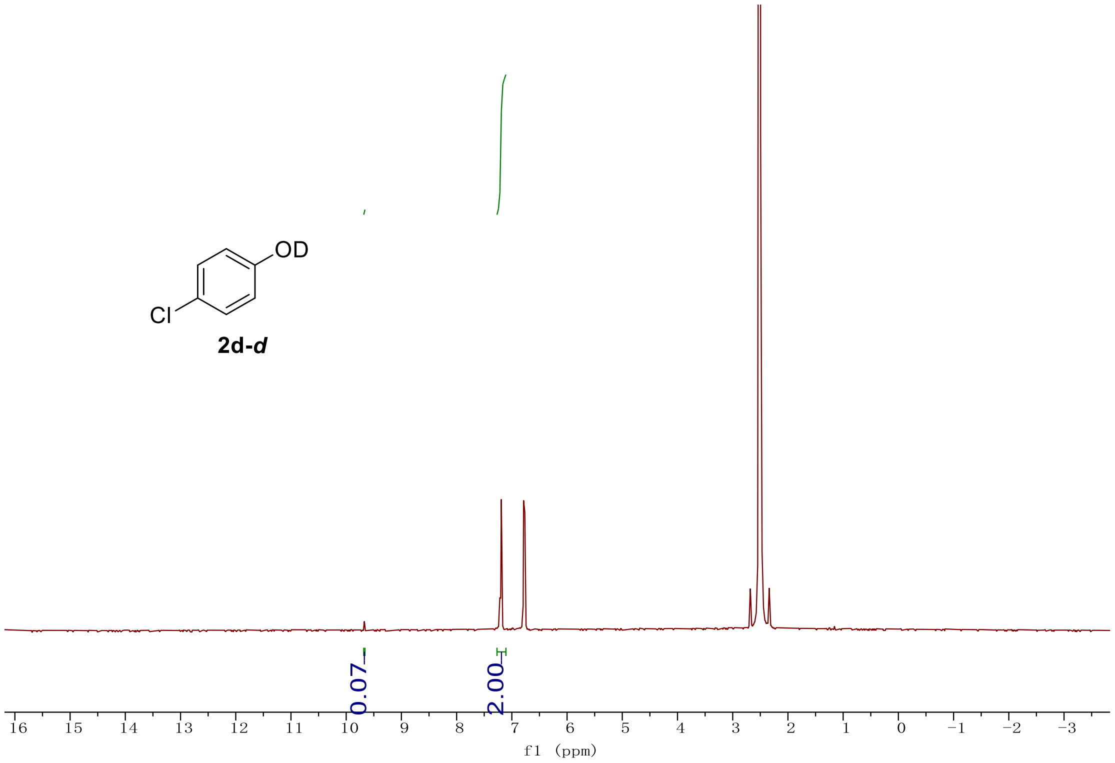

Figure S137. **^{1}H-NMR (400 MHz, DMSO-d$_6$) spectrum of compound 2d-*d***

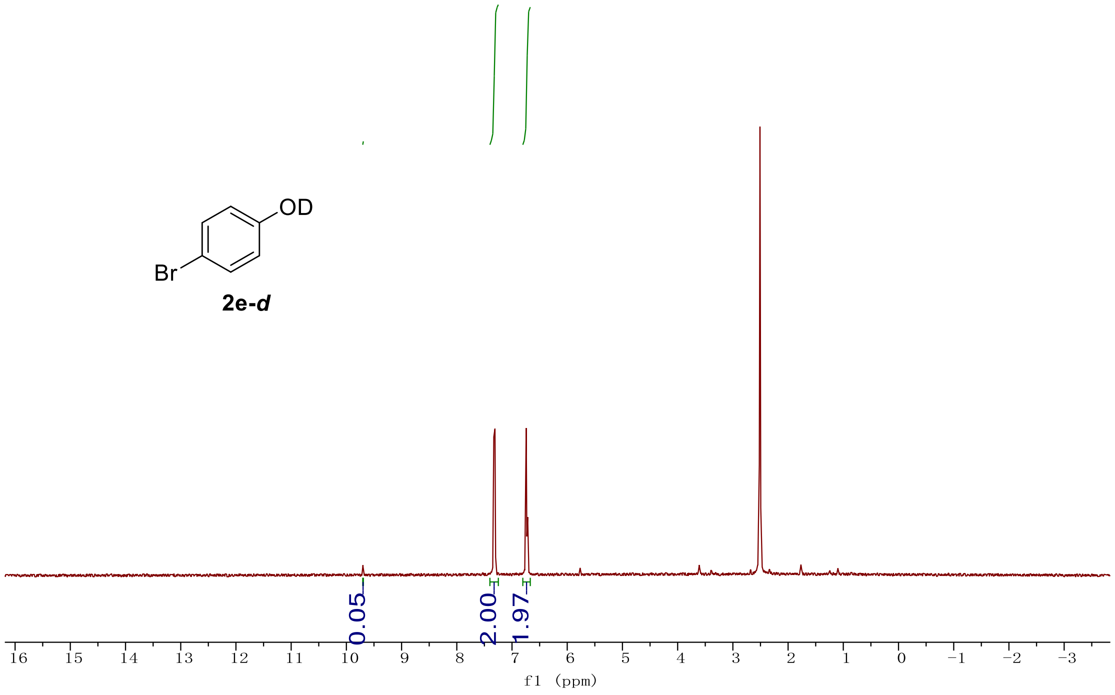

Figure S138. **^{1}H-NMR (400 MHz, DMSO-d$_6$) spectrum of compound 2e-*d***

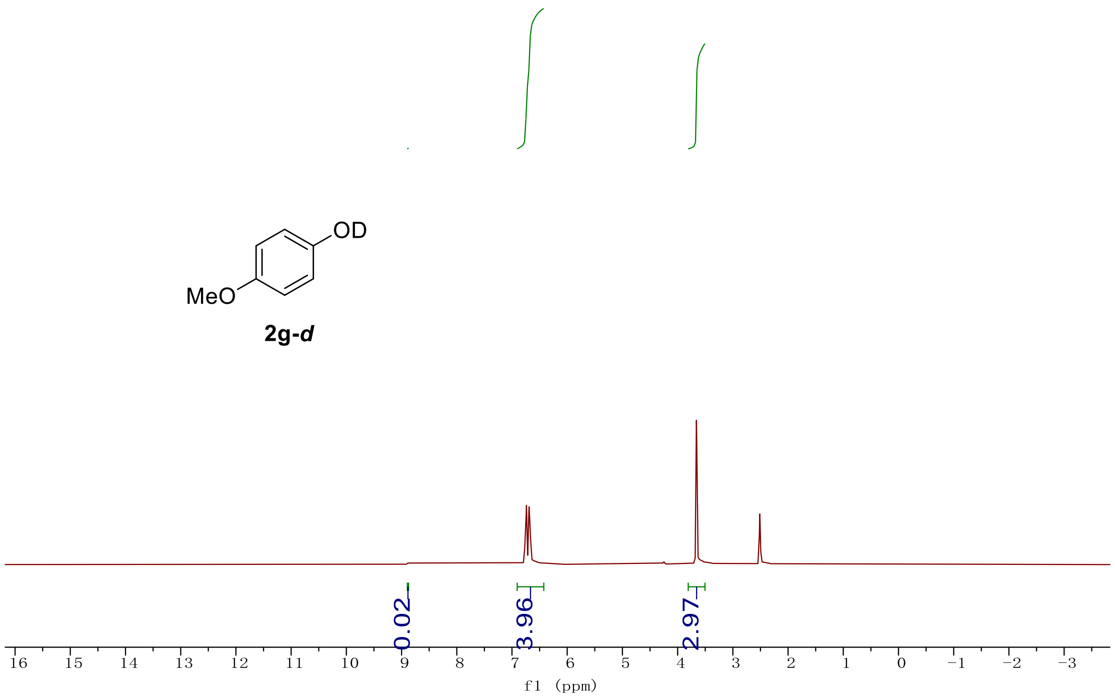

Figure S139. **^{1}H-NMR (400 MHz, DMSO-d$_6$) spectrum of compound 2g-*d***

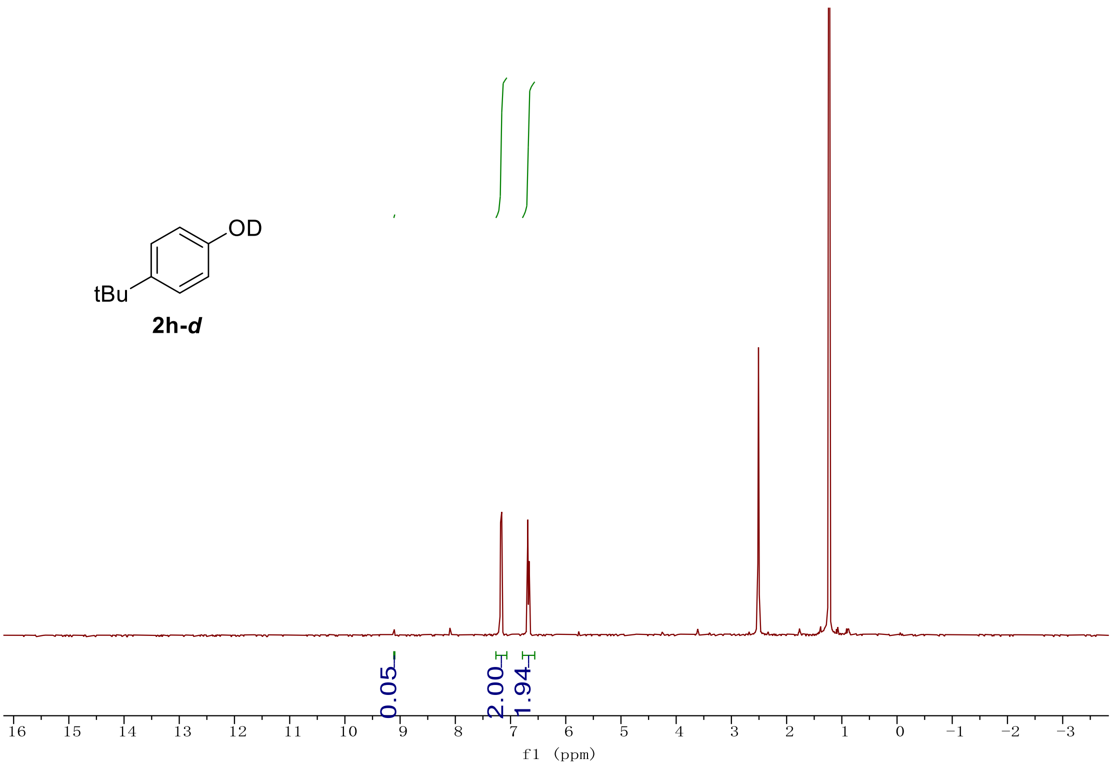

Figure S140. ^{1}H-NMR (400 MHz, DMSO-d6) spectrum of compound 2h-*d*

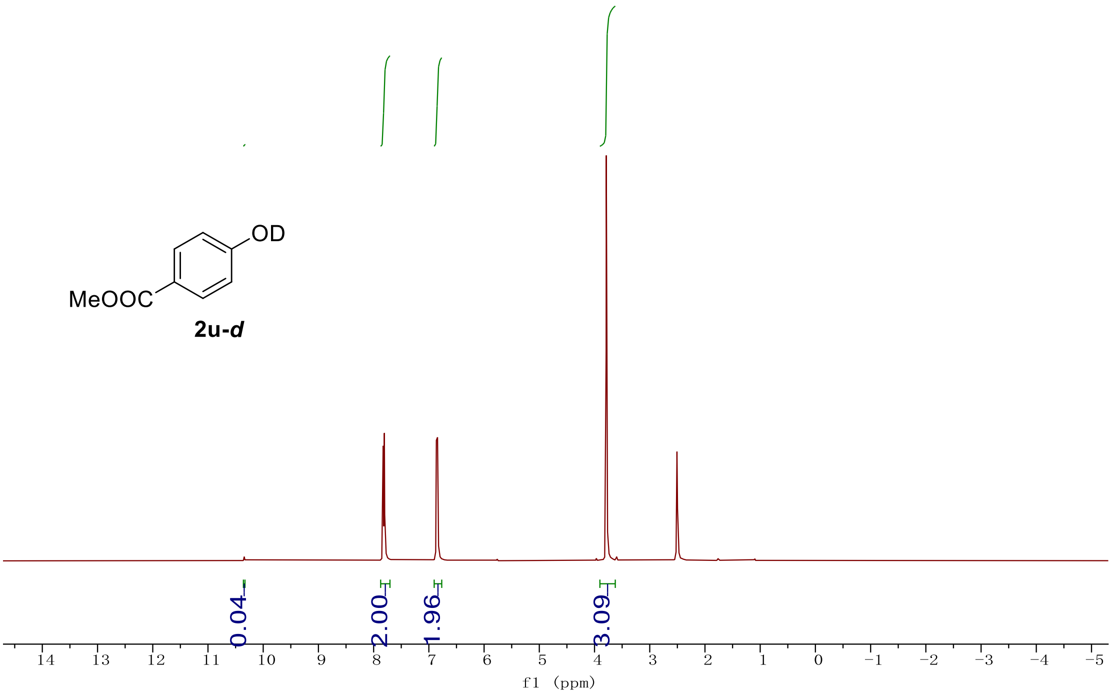

Figure S141. ^{1}H-NMR (400 MHz, DMSO-d6) spectrum of compound 2u-*d*

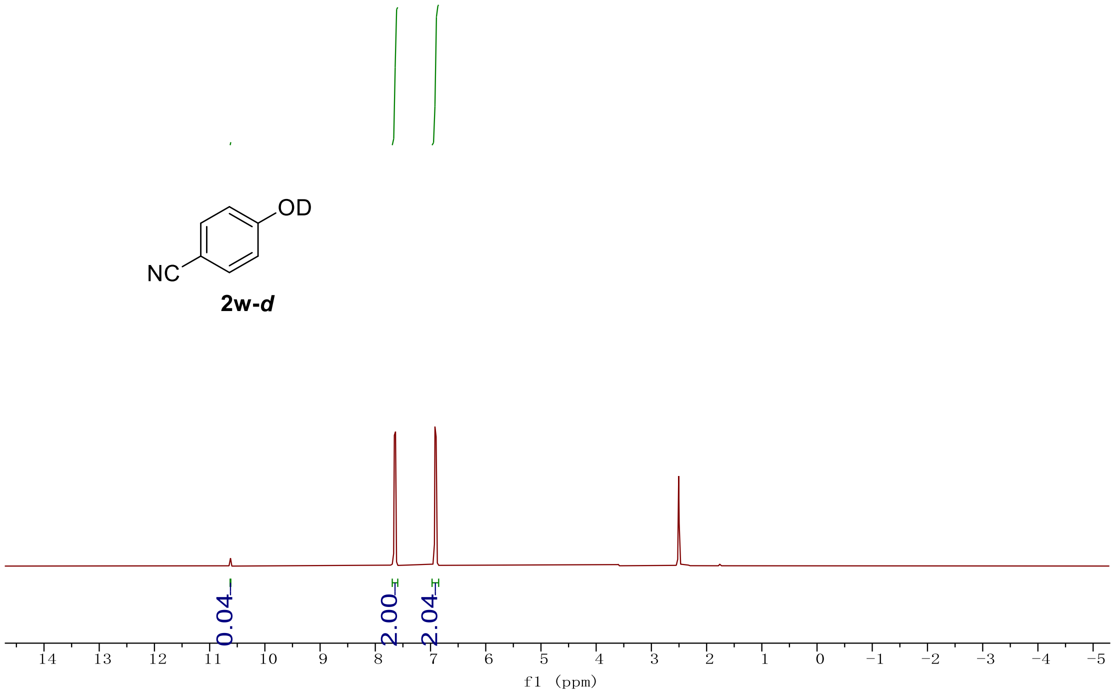

Figure S142. **^{1}H-NMR (400 MHz, DMSO-d$_6$) spectrum of compound 2w-*d***
